# Supplementary figures and images for: Body size estimation from isolated fossil bones reveals deep time evolutionary trends in North American lizards
Source: PLoS One. 2024 Jan 5;19(1):e0296318. doi: 10.1371/journal.pone.0296318 (PMC10769094; doi:10.1371/journal.pone.0296318)

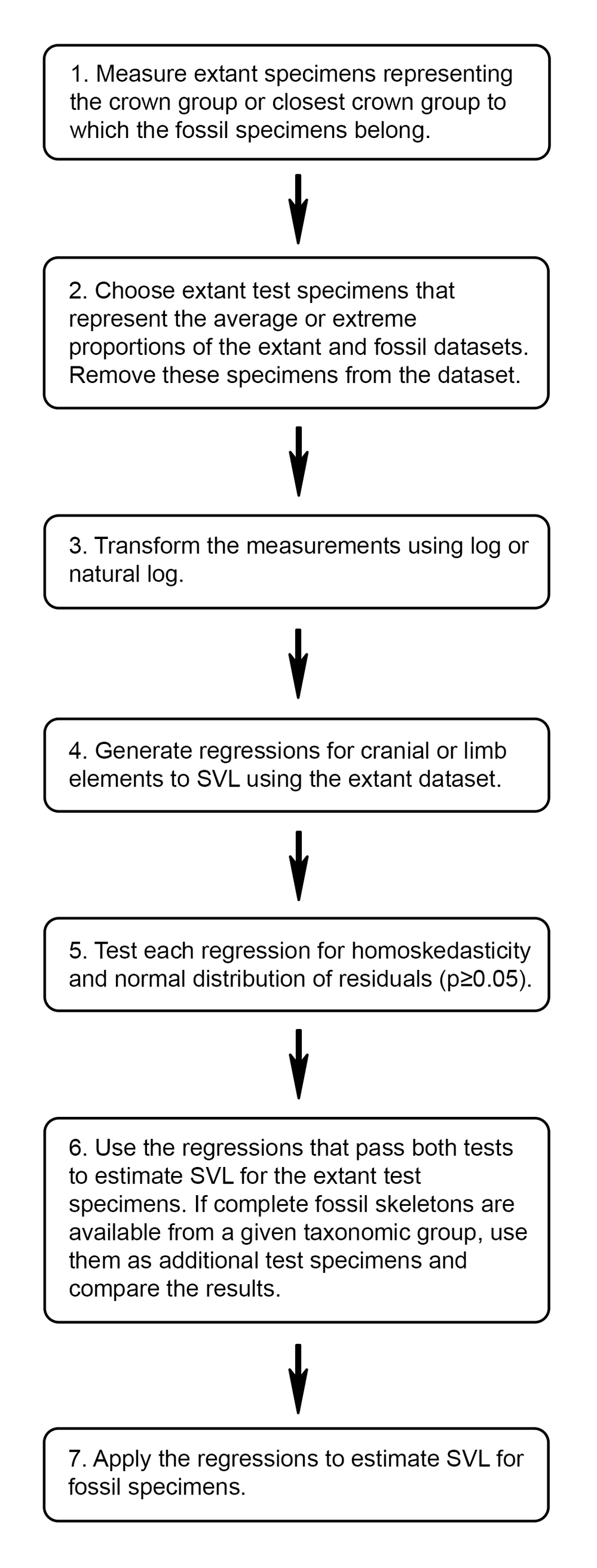

Supplement: S1 Fig — (TIF) [file pone.0296318.s001.tif]

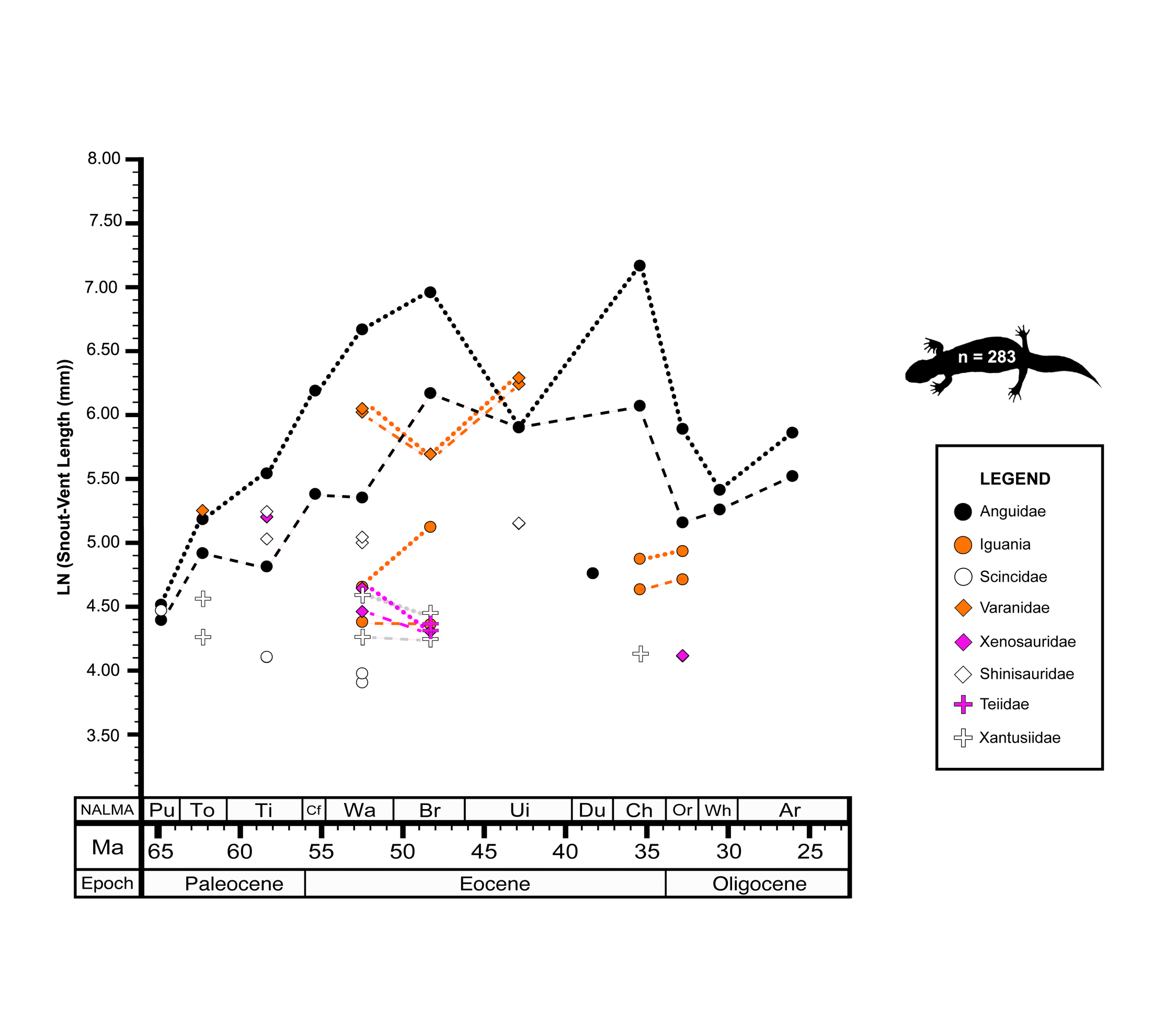

Supplement: S2 Fig — Body length is measured as snout-vent length (SVL) in mm. All measurements were transformed using natural log (LN). Dotted line = maximum. Dashed line = mean. Lines do not connect with the single Duchesnean datapoint since it likely does not accurately represent a maximum or mean value for that NALMA. North American Land Mammal Age (NALMA) abbreviations: Pu = Puercan, To = Torrejonian, Ti = Tiffanian, Cf = Clarkforkian, Wa = Wasatchian, Br = Bridgerian, Ui = Uintan, Du = Duchesnean, Ch = Chadronian, Or = Orellan, Wh = Whitneyan, Ar = Arikareean. (TIF) [file pone.0296318.s002.tif]

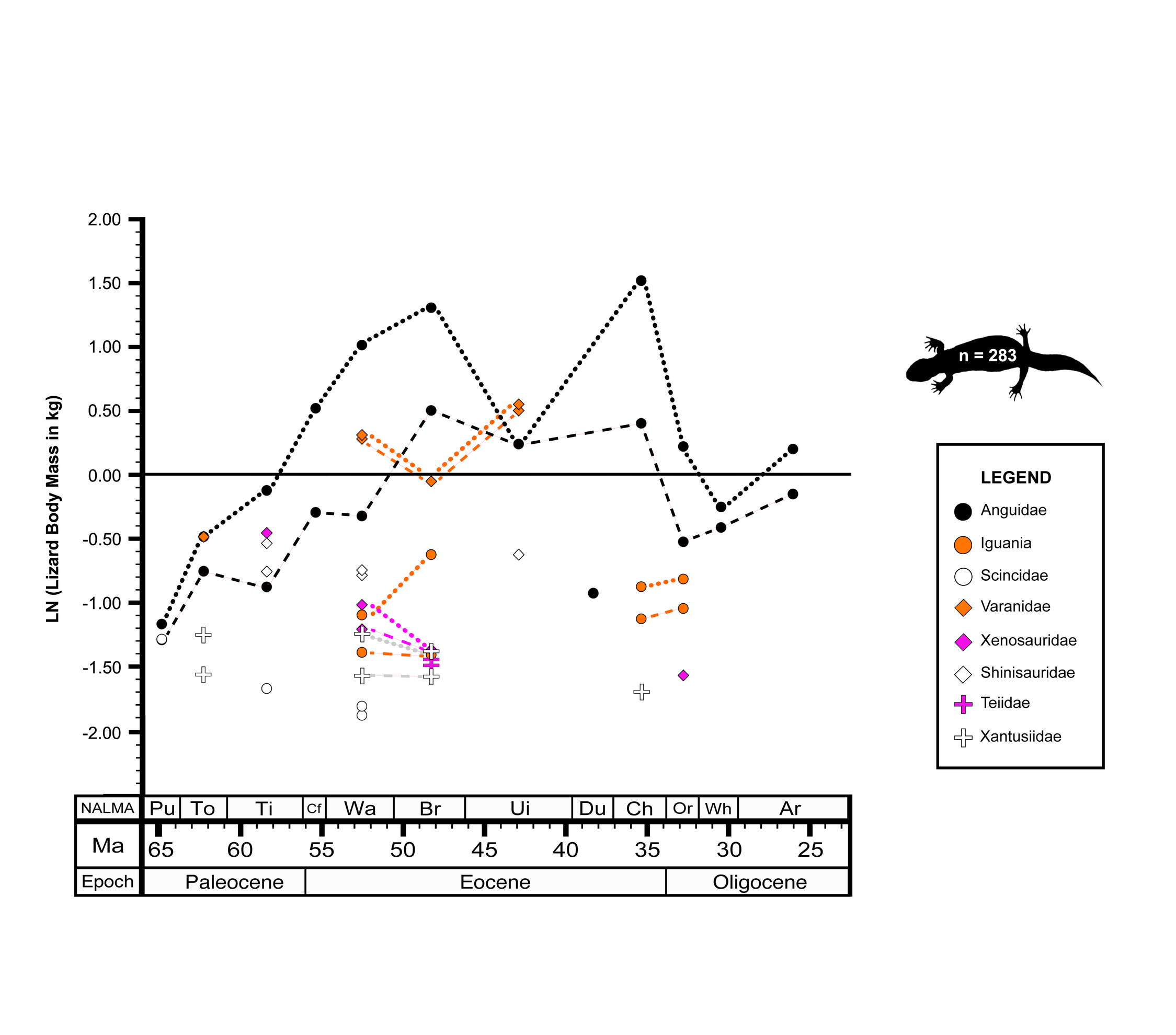

Supplement: S3 Fig — Mass estimates were calculated from snout-vent length (SVL) using the published equations listed in S8 Table. All measurements (in kg) were transformed using natural log (LN). Dotted line = maximum. Dashed line = mean. Solid black line indicates 1 kg threshold (LN(1) = 0.00). Lines do not connect with the single Duchesnean datapoint since it likely does not accurately represent a maximum or mean value for that NALMA. North American Land Mammal Age (NALMA) abbreviations: Pu = Puercan, To = Torrejonian, Ti = Tiffanian, Cf = Clarkforkian, Wa = Wasatchian, Br = Bridgerian, Ui = Uintan, Du = Duchesnean, Ch = Chadronian, Or = Orellan, Wh = Whitneyan, Ar = Arikareean. (TIF) [file pone.0296318.s003.tif]

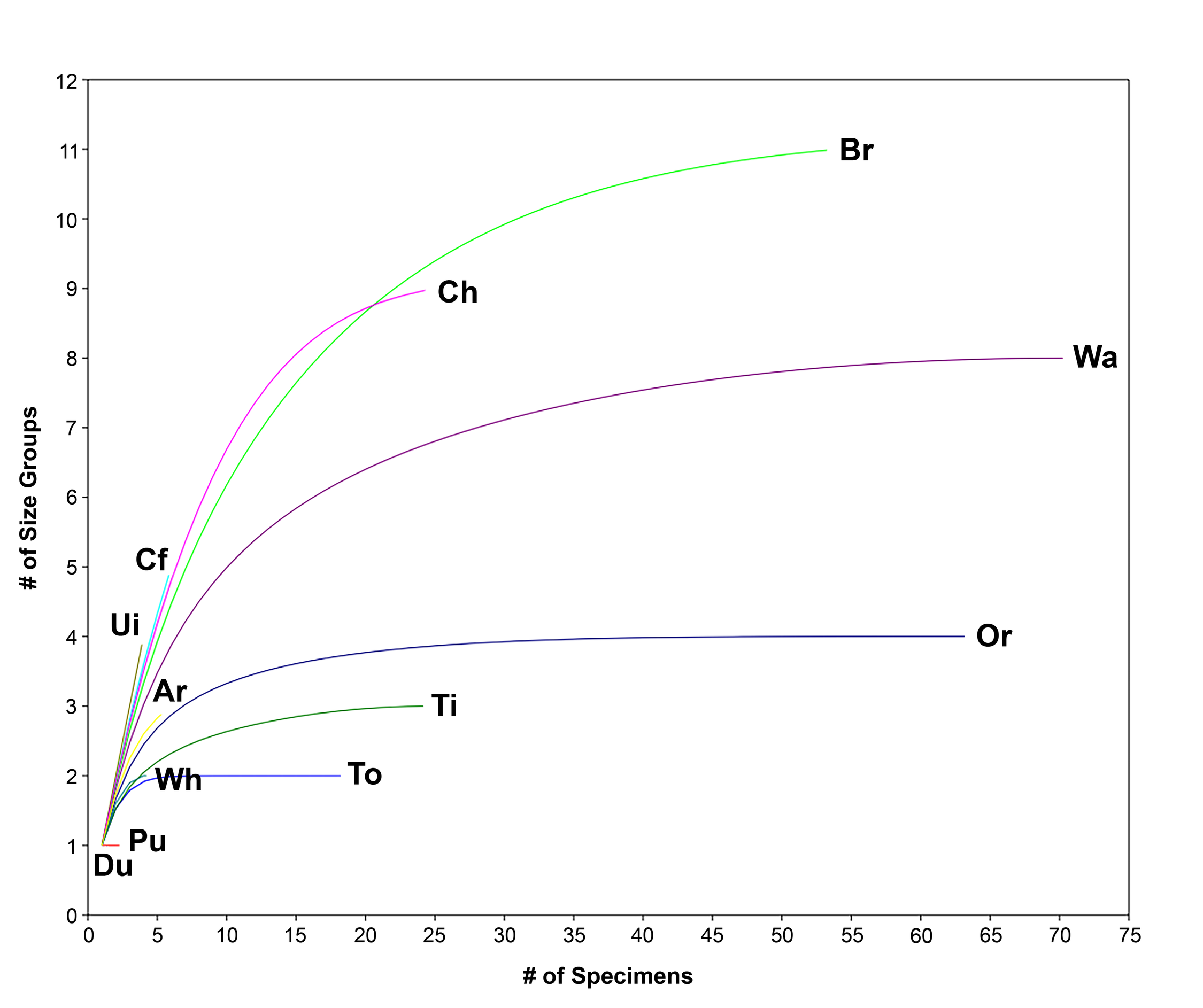

Supplement: S4 Fig — To conduct this analysis, each fossil specimen in S1 Dataset was assigned to one of 11 size groups, each group representing a bin of 100 mm (e.g., 1–99 mm, 100–199 mm, 200–299 mm, etc.; the last group was ≥ 1 m). S1 Dataset was subsampled here by North American Land Mammal Age (NALMA) interval. The y-axis in this graph shows the total number of size groups recovered in the sample for each NALMA interval. NALMA abbreviations: Pu = Puercan, To = Torrejonian, Ti = Tiffanian, Cf = Clarkforkian, Wa = Wasatchian, Br = Bridgerian, Ui = Uintan, Du = Duchesnean, Ch = Chadronian, Or = Orellan, Wh = Whitneyan, Ar = Arikareean. Individual rarefaction analysis was performed using PAST v4.03 [72]. (TIF) [file pone.0296318.s004.tif]

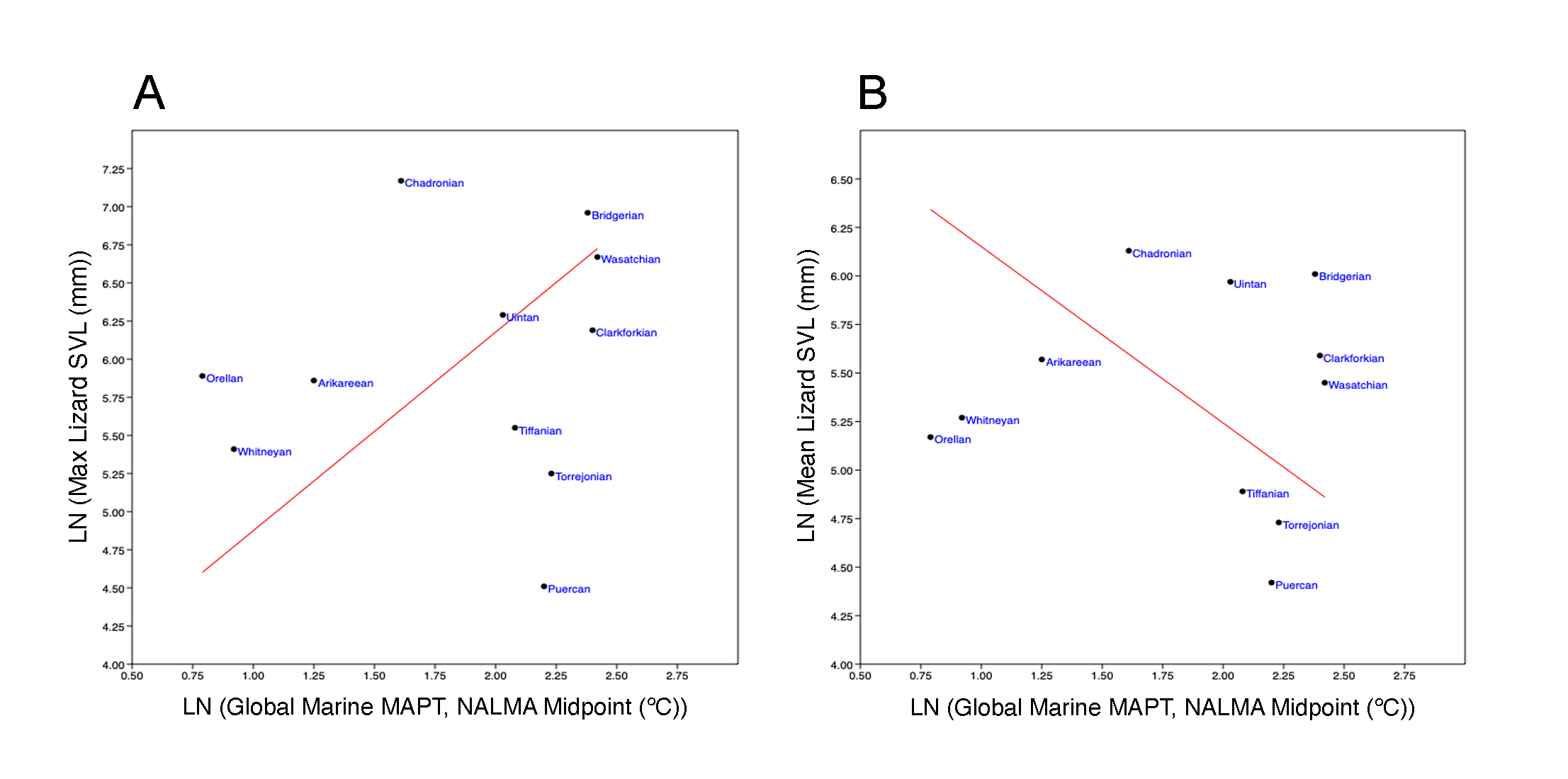

Supplement: S5 Fig — A) Global marine mean annual paleotemperature (MAPT) vs. maximum lizard SVL as a proxy of maximum body size. Function: LN(Max Lizard SVL) = ((1.30±0.431)*LN(Global Marine MAPT)) + (3.57±0.855), R2 = 0.02, p(uncorr.) = 0.71, SEE = 1.10. B) Global marine MAPT vs. mean lizard SVL. LN(Mean Lizard SVL) = ((-0.910±0.303)*LN(Global Marine MAPT)) + (7.06±0.606), R2 = 0.0004, p(uncorr.) = 0.95, SEE = 0.812. The graphs for correlations of global temperature with maximum and mean mass had the same spread of data. Temperature data are from Zachos et al. [45] and were taken from the NALMA midpoint in°C. All data were transformed using natural log. Regression analysis was performed using PAST v4.03 [72]. (TIF) [file pone.0296318.s005.tif]
