# Supplementary material for: Body size estimation from isolated fossil bones reveals deep time evolutionary trends in North American lizards
Source: PLoS One. 2024 Jan 5;19(1):e0296318. doi: 10.1371/journal.pone.0296318 (PMC10769094; doi:10.1371/journal.pone.0296318)
Supplement: S1 File — Additional references cited only in Supporting Information. (DOCX) [file pone.0296318.s019.docx]

**Supporting Information References**

Clark J, Beerbower JR, Kietzke KK. Oligocene sedimentation, stratigraphy, paleoecology and paleoclimatology in the big Badlands of South Dakota. Fieldiana. 1967;5: 21–74.

Estes R. The fossil record and early distribution of lizards. Adv Herpetol Evol Biol. 1983; 365–398.

Gutiérrez-Rodríguez J, Zaldívar-Riverón A, Solano-Zavaleta I, Campbell JA, Meza-Lázaro RN, Flores-Villela O, et al. Phylogenomics of the Mesoamerican alligator-lizard genera Abronia and Mesaspis (Anguidae: Gerrhonotinae) reveals multiple independent clades of arboreal and terrestrial species. Mol Phylogenet Evol. 2021;154: 106963. doi:10.1016/j.ympev.2020.106963

Jepsen, GL. Paleocene faunas of the Polecat Bench Formation, Park County, Wyoming: Part I. Proc Amer Phil Soc. 1940;217-340.

Nydam RL, Fitzpatrick BM. The occurrence of contogenys-like lizards in the late cretaceous and early tertiary of the western interior of the U.S.A. J Vertebr Paleontol. 2009;29: 677–701. doi:10.1671/039.029.0331

Schools M, Hedges SB. Phylogenetics, classification, and biogeography of the Neotropical forest lizards (Squamata, Diploglossidae). 2021;4974: 201–257.

Sullivan RM. Revision of the Paleogene genus *Glyptosaurus* (Reptilia, Anguidae). Bull Am Museum Nat Hist. 1979;163.

Sullivan RM. Parophisaurus pawneensis (Gilmore, 1928) New Genus of Anguid Lizard from the Middle Oligocene of North America. J Herpetol. 1987;21: 115–133. doi:10.2307/1564472

Sullivan RM, Lucas SG. Fossil Squamata from the San José Formation, early Eocene, San Juan Basin, New Mexico. J Paleontol. 1988;631-9.

Terry DO, LaGarry HE, Hunt RM, editors. Depositional environments, lithostratigraphy, and biostratigraphy of the White River and Arikaree groups (late Eocene to early Miocene, North America). Geol Soc Am. 1998; 65.
