# Supplementary material for: Body size estimation from isolated fossil bones reveals deep time evolutionary trends in North American lizards
Source: PLoS One. 2024 Jan 5;19(1):e0296318. doi: 10.1371/journal.pone.0296318 (PMC10769094; doi:10.1371/journal.pone.0296318)
Supplement: Attachment — Submitted filename: PONE-D-22-04244_R2_MeloroComments.pdf [file pone.0296318.s027.pdf]

PLOS ONE

Body size estimation from isolated fossil bones reveals deep time evolutionary trends for lizards

--Manuscript Draft--

**Manuscript Number:**	PONE-D-22-04244R2
**Article Type:**	Research Article
**Full Title:**	Body size estimation from isolated fossil bones reveals deep time evolutionary trends for lizards
**Short Title:**	Body size estimation from isolated fossil bones reveals deep time evolutionary trends for lizards
**Corresponding Author:**	Sara J ElShafie, PhD<br>UC Berkeley: University of California Berkeley<br>Berkeley, CA UNITED STATES
**Keywords:**	Body size; Reptiles; lizards; vertebrate paleobiology; size regressions; fossils
**Abstract:**	Lizards play vital roles in extant ecosystems. However, their roles in extinct ecosystems are poorly understood because the fossil record of lizards consists mostly of isolated bones. This makes it difficult to document changes in lizard morphology and body size over time, which is essential for studies of lizard paleoecology and evolution. It is also difficult to compare available fossil lizard data with existing extant lizard datasets because extant studies rarely measure individual bones. Furthermore, no previous study has regressed measurements of individual bones to body length across crown lizard groups, nor tested those regressions on fossil skeletons. An extensive dataset of extant lizard measurements across crown taxonomic groups is provided here, including individual bones, from which methods for estimating lizard body size from isolated fossil elements were derived. These methods were applied to a comparably large dataset of fossil lizard specimens from the robust Paleogene record (66-23 Mya) of the Western Interior of North America. This study tests the hypothesis that anatomical proportions have been conserved within higher-level crown lizard groups since the Paleogene and can therefore be used to reconstruct snout-vent length (SVL) for fossil specimens referred to the same groups.<br>Individual bones were regressed to SVL in each extant lizard group and the regressions were tested using measurements from complete extant specimens as well as some rare fossil skeletons. The regressions predicted actual SVL for both the extant and fossil test specimens with similar accuracy. The resulting body size estimates for the entire fossil dataset revealed that lizards reached greatest maximum body size for this study period in the middle Paleogene, with the largest size class dominated by anguid lizards that exceeded 1 meter in SVL and 1 kg in body mass. Maximum body size decreased to under 400 mm in the late Paleogene. This is the first study to investigate body size evolution across lizard clades over a deep time interval and for a large geographic region. The proposed methods can provide estimates of SVL for fossil lizards referred to any crown group.
**Order of Authors:**	Sara J ElShafie, PhD
**Opposed Reviewers:**	
**Response to Reviewers:**	February 28, 2023<br><br>Dear PLoS One Editorial Board,<br><br>Thank you for the opportunity to make these additional revisions to my manuscript. Below is a list of the new changes that I made.<br><br>Overall Changes Made:<br><br>•I revised the whole manuscript to be in third-person passive voice to make it sound more impersonal.

	•I separated the Results and Discussion sections and substantially revised both sections based on the Editor and Reviewer comments, including omitting the section on Competition from the Discussion.<br><br>Body Mass Estimations and Equations<br><br>•I agree that adding equations for estimating mass of fossil specimens from individual bone measurements is useful. However, I cannot generate novel equations for body mass from individual lengths here because almost none of the extant lizard specimens that I measured included measurements of body mass. Unfortunately, lizard specimens that are dry skeletonized almost never have the original weight recorded before they are processed. Furthermore, measurements of individual cranial and limb bones are not provided in Feldman et al. (2016) or Meiri (2010), both of which were recommended by Reviewer 2 as sources for extant lizard measurements and equations for lizard body mass from SVL. Therefore, I do not have the data necessary to generate novel equations for estimating body mass from individual cranial or limb bones.<br>oInstead, I translated the original equations that I provided here for estimating SVL from individual bones (Tables S1-S7) into the equations for estimating mass from SVL provided in Feldman et al. (2016; see S8 Table). The resulting equations have been added to Tables S1-S7. These translated equations can now be used to estimate lizard body mass from individual cranial and limb bones.<br>oThe mass estimates are the same whether you calculate mass from SVL using the equations from Feldman et al. (2016) or the new equations for body mass from individual elements provided here.<br>•I am still including the original equations I generated for estimating SVL from individual bones because SVL is also an important variable for paleontologists and zoologists.<br>•Calculating body mass equations for individual fossil species would not be practical because fossil specimens usually cannot be identified to individual species. In addition, even genus-level assignments often change for fossil specimens upon later reexamination.<br>oIt is safer and more practical to develop family-level equations - that way, the body mass estimate becomes resilient to any specific taxonomic identification changes. Furthermore, family-level equations are based on a larger sample size. This approach is consistent with the rest of the paper, and with the sources used (Meiri 2010; Meiri et al. 2013; Feldman et al. 2016), which focus on family-level analyses.<br><br>New Figures and Tables<br><br>•I added the following figures:<br>oFig 7. Body mass distribution by taxonomic group for fossil lizards in the Western Interior of North America through the Paleogene, including a line for the overall mean lizard body mass per NALMA.<br>oS6 Fig. Mean and maximum snout-vent length in fossil lizards by taxonomic group per NALMA.<br>oS7 Fig. Mean and maximum body mass in fossil lizards by taxonomic group per NALMA.<br>I added commentary about the latter two new figures in the revised Discussion as well.<br>•I also added a line for the overall mean lizard snout-vent length per NALMA to the SVL distribution graph in Fig 6.<br>•I added the following tables:<br>oS8 Table. Published equations for estimating lizard body mass from snout-vent length.<br>oS11 Table. Mean and maximum fossil lizard snout-vent length and body mass by taxonomic group per NALMA.<br>•The numbers of all other figures and tables were also updated accordingly.<br><br><br>I made all changes suggested by the Editor and Reviewers, with the following notes…<br><br>Additional Editor Comments:<br><br>•Table 2 is a bit broad and I think it would be better a figure showing specimens and /or species sampled by group / time period in comparison with diversity expected as by

Powered by Editorial Manager® and ProduXion Manager® from Aries Systems Corporation

	the palaeodb database.<br>oI still prefer these data presented as a table, and I think it's helpful to have the NALMAs listed here in table format with their age ranges. But I took the suggestion of adding the number of genera and species sampled per taxonomic group per NALMA compared to the numbers expected based on the Paleobiology Database (PBDB). These data have been added to the table. I think the result is more a reflection of how much is missing from the PBDB, but the data are now provided for comparison.<br>•Table 3 should be simplified showing slope as parameter (b), confidence interval and intercept + R2 and SEE.<br>oI made these changes, but for the sake of space and simplicity, I listed the standard error value rather than listing the full confidence interval (e.g., - X.XXX to X.XXX).<br>•Line 264-269: ok but perhaps this might be due to some outliers or some groups whose body bauplan differ from the "general" lizard one. What if you try exclusion of some groups?<br>oI did try excluding some groups, and the attempted general regressions still did not pass the tests for homoskedasticity and normal distribution of residuals. In any case, now that body mass estimation equations specific to taxonomic group are used in this study, I think a generalized lizard SVL equation is unnecessary.<br>•Reduce extensively also the part on sex (line 936-948). You did not have sex to test so all you are saying is quite speculative.<br>oI think the comment made here is an important point to make for paleontological studies, and Reviewer 1 agreed. However, I did shorten this paragraph.<br><br>Reviewer 2 Comments:<br>•I made a mistake recommending Meiri 2010 in my last review for body mass estimates. I meant Feldman 2016 (which Meiri is on) supplementary appendix 2 (see below). It has all of the regression equations needed for your study including the families missing at the moment. There is an equation for legged anuids and legless anguids. If I'm not mistaken there are a few species that you are not sure if they had limbs or not? In which case you might want to use the Anguimorpha equation, or simply assume that they had limbs if there are no known limbless glyptosaurs. In any case you definitely need to change this.<br>oI used updated equations from Feldman et al. (2016). The equations listed in this reference were only different for Anguidae, Xenosauridae, Iguania, and Anguimorpha; the rest were taken from Meiri (2010), so I didn't need to change those. I used the Anguimorpha equation for Shinisauridae since an equation for Shinisauridae was not provided and Shinisauridae falls within Anguimorpha. I used the equation for Fully Legged Anguidae for the fossil anguid lizards because limb material has been found with many fossil anguid specimens – none have been interpreted as limbless.<br>•27 – you say "large dataset" of fossil lizards but I think that you should be specific in saying what percent of Midwest species you studied. You can check this on the paleobiology database I think.<br>oI honestly don't know what Reviewer 2 meant by this comment. Only some of the fossil lizards in my dataset were collected in the Midwest, and I don't know why that matters in the context of this study. If they meant that I should specify how many of the taxa in the fossil dataset still occur in the Western Interior today, I point that out in the Results section (see "Taxonomic Turnover"). I don't think the Introduction is the place for that detail.<br>•414-421 – really interesting analysis, you should use family as an additional variable in addition to doing it across all species. Also, how did you try to correlate them? Linear regression model? ANOVA based on each NALMA? What was the predictor and what was the response? This remains very unclear in the text. You should provide a quantitative analysis, perhaps similar to Smith 2018. Or at the very least, a very in-depth qualitative analysis/discussion. Quantitative is always better, so I would recommend that. I would personally recommend using linear regressions with estimated body size of species as the response, and temperature per million years as the predictor (check where smith got the data, I think Zachos something). You can also use family as an additional covariate.<br>oI revised the text to clarify how I set up this regression. However, the additional analyses suggested here were not feasible because the predictor variable (lizard body size) could not be binned per million years due to the way in which the specimens were documented in museum collections (the majority of the specimens are only referred to a NALMA interval and cannot be referred to a more precise time bin). Furthermore,

	only two lizard families (Anguidae and Varanidae) represent the maximum lizard body size for any given NALMA, and the record of Varanidae is not continuous enough through the Paleogene to set up a correlation (see S6-S7 Fig). The correlation is already mostly a correlation between maximum anguid body size and varanid body size.<br><br>Thank you again for the considerable time you, the Editor, and the Reviewers have contributed to reviewing this paper, and for your patience as I incorporated the suggestions into the manuscript. I look forward to hearing back from you.<br><br>Best regards,<br><br>Sara J. ElShafie, Ph.D.<br>Alumnus, Department of Integrative Biology and Museum of Paleontology<br>University of California, Berkeley<br>selshafie@berkeley.edu
**Additional Information:**	
**Question**	**Response**
**Financial Disclosure**<br><br>Enter a financial disclosure statement that describes the sources of funding for the work included in this submission. Review the submission guidelines for detailed requirements. View published research articles from *PLOS ONE* for specific examples.<br><br>This statement is required for submission and **will appear in the published article** if the submission is accepted. Please make sure it is accurate.	This work was supported by the Anthony Barnosky Graduate Student Research Fund (UCMP – ucmp.berkeley.edu), the Doris O. and Samuel P. Welles Research Fund (UCMP), the Department of Integrative Biology at the University of California Berkeley (ib.berkeley.edu), the Mary R. Dawson Award and the Albert E. Wood Award (Society for Vertebrate Paleontology – vertpaleo.org), the Charles A. and June R.P. Ross Research Award (Geological Society of America – www.geosociety.org), the Evolving Earth Foundation Graduate Student Research Grant (www.evolvingearth.org), the Burke Museum Vertebrate Paleontology Collections Study Grant (www.burkemuseum.org), the Sigma Xi Grant in Aid of Research (www.sigmaxi.org), the Friends of the Nebraska State Museum (friendsofthemuseum.org), the Nebraska Geological Society (www.nebraskageologicalsociety.org), the Department of Earth and Atmospheric Sciences at the University of Nebraska Lincoln (eas.unl.edu), the Sakana Foundation, and the Uplands Foundation. The funders had no role in study design, data collection and analysis, decision to publish, or preparation of the manuscript.

Powered by Editorial Manager® and ProduXion Manager® from Aries Systems Corporation

**Unfunded studies**<br>Enter: *The author(s) received no specific funding for this work.*<br><br>**Funded studies**<br>Enter a statement with the following details:<br>• Initials of the authors who received each award<br>• Grant numbers awarded to each author<br>• The full name of each funder<br>• URL of each funder website<br>• Did the sponsors or funders play any role in the study design, data collection and analysis, decision to publish, or preparation of the manuscript?<br>• **NO** - Include this sentence at the end of your statement: *The funders had no role in study design, data collection and analysis, decision to publish, or preparation of the manuscript.*<br>• **YES** - Specify the role(s) played.<br><br>* typeset	
**Competing Interests**<br><br>Use the instructions below to enter a competing interest statement for this submission. On behalf of all authors, disclose any competing interests that could be perceived to bias this work—acknowledging all financial support and any other relevant financial or non-financial competing interests.<br><br>This statement is required for submission and **will appear in the published article** if the submission is accepted. Please make sure it is accurate and that any funding sources listed in your Funding Information later in the submission form are also declared in your Financial Disclosure statement.<br><br>View published research articles from *PLOS ONE* for specific examples.	The author has declared that no competing interests exist.

Powered by Editorial Manager® and ProduXion Manager® from Aries Systems Corporation

**NO authors have competing interests**<br><br>Enter: *The authors have declared that no competing interests exist.*<br><br>**Authors with competing interests**<br><br>Enter competing interest details beginning with this statement:<br><br>*I have read the journal's policy and the authors of this manuscript have the following competing interests: [insert competing interests here]*<br><br>* typeset	
**Ethics Statement**<br><br>Enter an ethics statement for this submission. This statement is required if the study involved:<br><br>• Human participants<br>• Human specimens or tissue<br>• Vertebrate animals or cephalopods<br>• Vertebrate embryos or tissues<br>• Field research<br><br>Write "N/A" if the submission does not require an ethics statement.<br><br>General guidance is provided below. Consult the submission guidelines for detailed instructions. **Make sure that all information entered here is included in the Methods section of the manuscript.**	N/A

**Format for specific study types**<br><br>**Human Subject Research (involving human participants and/or tissue)**<br>• Give the name of the institutional review board or ethics committee that approved the study<br>• Include the approval number and/or a statement indicating approval of this research<br>• Indicate the form of consent obtained (written/oral) or the reason that consent was not obtained (e.g. the data were analyzed anonymously)<br><br>**Animal Research (involving vertebrate animals, embryos or tissues)**<br>• Provide the name of the Institutional Animal Care and Use Committee (IACUC) or other relevant ethics board that reviewed the study protocol, and indicate whether they approved this research or granted a formal waiver of ethical approval<br>• Include an approval number if one was obtained<br>• If the study involved *non-human primates*, add *additional details* about animal welfare and steps taken to ameliorate suffering<br>• If anesthesia, euthanasia, or any kind of animal sacrifice is part of the study, include briefly which substances and/or methods were applied<br><br>**Field Research**<br><br>Include the following details if this study involves the collection of plant, animal, or other materials from a natural setting:<br>• Field permit number<br>• Name of the institution or relevant body that granted permission	
**Data Availability**<br><br>Authors are required to make all data underlying the findings described fully available, without restriction, and from the time of publication. PLOS allows rare exceptions to address legal and ethical concerns. See the PLOS Data Policy and FAQ for detailed information.	Yes - all data are fully available without restriction

A Data Availability Statement describing where the data can be found is required at submission. Your answers to this question constitute the Data Availability Statement and **will be published in the article**, if accepted.<br><br>**Important:** Stating 'data available on request from the author' is not sufficient. If your data are only available upon request, select 'No' for the first question and explain your exceptional situation in the text box.<br><br>Do the authors confirm that all data underlying the findings described in their manuscript are fully available without restriction?	
**Describe where the data may be found in full sentences. If you are copying our sample text, replace any instances of XXX with the appropriate details.**<br><br>• If the data are **held or will be held in a public repository**, include URLs, accession numbers or DOIs. If this information will only be available after acceptance, indicate this by ticking the box below. For example: *All XXX files are available from the XXX database (accession number(s) XXX, XXX.).*<br>• If the data are all contained **within the manuscript and/or Supporting Information files**, enter the following: *All relevant data are within the manuscript and its Supporting Information files.*<br>• If neither of these applies but you are able to provide **details of access elsewhere**, with or without limitations, please do so. For example:<br><br>*Data cannot be shared publicly because of [XXX]. Data are available from the XXX Institutional Data Access / Ethics Committee (contact via XXX) for researchers who meet the criteria for access to confidential data.*<br><br>*The data underlying the results presented in the study are available from (include the name of the third party*	All relevant data are within the manuscript and its Supporting Information files.

Powered by Editorial Manager® and ProduXion Manager® from Aries Systems Corporation

and contact information or URL).<br>• This text is appropriate if the data are owned by a third party and authors do not have permission to share the data.<br><br>* typeset	
Additional data availability information:	

UNIVERSITY OF CALIFORNIA, BERKELEY

BERKELEY • DAVIS • IRVINE • LOS ANGELES • MERCED • RIVERSIDE • SAN DIEGO • SAN FRANCISCO SANTA BARBARA • SANTA CRUZ

DEPARTMENT OF INTEGRATIVE BIOLOGY
MUSEUM OF PALEONTOLOGY
1101 VALLEY LIFE SCIENCES BUILDING
BERKELEY, CA 94720-3140
(510) 642-1821

PLOS One
Editorial Board

February 10, 2022

Dear Editorial Board,

I am pleased to submit this original article entitled "Body size estimation from isolated fossil bones reveals deep time evolutionary trends for lizards" for consideration for publication in the peer-reviewed journal *PLOS One*.

This manuscript investigates patterns of deep time body size evolution in lizards. Body size is essential to understanding ecology, and lizards play vital ecological roles in extant ecosystems today. Yet lizard paleoecology and body size evolution have not been previously assessed on large taxonomic or geographic scales over geologic time intervals because the lizard fossil record consists mostly of isolated bones. Estimating body size from such specimens would require a large dataset of corresponding measurements from extant lizards. However, existing surveys of extant lizard diversity do not include measurements of individual bones.

I address this need by providing an extensive dataset of extant lizard measurements (n = 333), including individual cranial and limb bone lengths, from a range of crown groups. I generated anatomical regressions from these extant data and found that they accurately predict body length for available fossil lizard skeletons. These results indicate that anatomical proportions are conserved within lizard crown groups over geologic time scales.

I further applied these regressions to a comparably large dataset of incomplete fossil lizard specimens (n = 283) sampled from across the Western Interior of the United States through the Paleogene record (66-23 Mya). The results indicate that lizard body size in this region peaked in the early-mid Paleogene, with the largest size class dominated by varanids and heavily armored anguid lizards as large as 1 meter in snout-vent length. In an extensive discussion of their paleoecological context, I explore evidence suggesting that these lizards may have achieved large body size through mixed carnivorous, omnivorous, or durophagous diets. This is the first study to reconstruct body size evolution patterns for multiple lizard clades over a deep time interval and across a large geographic region.

I consider this manuscript appropriate for publication by *PLOS One* because it addresses far-reaching questions and offers integrative methods that will be of broad interest and application to larger scientific communities, especially those interested in vertebrate paleontology, paleoecology, reptile biology, evolutionary biology, functional morphology, and comparative anatomy.

This manuscript has not been published in any other peer-reviewed journal and is not under consideration for publication elsewhere. The submitted draft of the manuscript contains 14,976 words, nine figures, and three tables, in addition to two supplementary figures, ten supplementary tables, and two supplementary datasets.

I have no conflicts of interest to disclose. I have not had any prior discussions about this manuscript with a *PLOS One* Editorial Board Member.

If considered appropriate for *PLOS One*, I suggest the following individuals to review my submission:

- Melissa Kemp, Assistant Professor, University of Texas, Austin (mkemp@austin.utexas.edu) – expertise in lizard paleoecology and evolution.
- Hank Woolley, Doctoral Candidate, University of Southern California (chwoolle@usc.edu) – expertise in lizard morphology, paleobiology and systematics, including broad-scale taphonomic analyses of lizard fossil records.
- Simon Scarpetta, Postdoctoral Fellow, Museum of Vertebrate Zoology, University of California, Berkeley (scas100@berkeley.edu) – expertise in lizard paleobiology and systematics.
- Raymond Huey, Professor Emeritus, University of Washington (hueyrb@uw.edu) – expertise in lizard ecology and physiology.
- Donald Miles, Professor, Ohio University (milesd@ohio.edu) – expertise in lizard ecology and physiology.

Thank you very much for your consideration. I look forward to hearing from you.

Sincerely,

Sara J. ElShafie
Doctoral Candidate

Department of Integrative Biology and
Museum of Paleontology
University of California, Berkeley
1101 Valley Life Sciences Building
Berkeley, CA 94720-4780
(510) 642-1821
selshafie@berkeley.edu

UNIVERSITY OF CALIFORNIA, BERKELEY

BERKELEY • DAVIS • IRVINE • LOS ANGELES • MERCED • RIVERSIDE • SAN DIEGO • SAN FRANCISCO SANTA BARBARA • SANTA CRUZ

DEPARTMENT OF INTEGRATIVE BIOLOGY
MUSEUM OF PALEONTOLOGY
1101 VALLEY LIFE SCIENCES BUILDING
BERKELEY, CA 94720-3140
(510) 642-1821

PLOS One
Editorial Board

February 28, 2023

Dear Editorial Board,

I am pleased to resubmit this original article entitled "Body size estimation from isolated fossil bones reveals deep time evolutionary trends for lizards" for consideration for publication in the peer-reviewed journal *PLOS One*.

This manuscript investigates patterns of deep time body size evolution in lizards. Body size is essential to understanding ecology, and lizards play vital ecological roles in extant ecosystems today. Yet lizard paleoecology and body size evolution have not been previously assessed on large taxonomic or geographic scales over geologic time intervals because the lizard fossil record consists mostly of isolated bones. Estimating body size from such specimens would require a large dataset of corresponding measurements from extant lizards. However, existing surveys of extant lizard diversity do not include measurements of individual bones.

I address this need by providing an extensive dataset of extant lizard measurements (n = 266), including individual cranial and limb bone lengths, from a range of crown groups. I generated anatomical regressions from these extant data and found that they accurately predict body length for available fossil lizard skeletons. These results indicate that anatomical proportions are conserved within lizard crown groups over geologic time scales.

I further applied these regressions to a comparably large dataset of incomplete fossil lizard specimens (n = 283) sampled from across the Western Interior of the United States through the Paleogene record (66-23 Mya). The results indicate that lizard body size in this region peaked in the early-mid Paleogene, with the largest size class dominated by varanids and anguid lizards as large as 1 meter in snout-vent length. I discuss potential climatic drivers of gigantism in these lizards. This is the first study to reconstruct body size evolution patterns for multiple lizard clades over a deep time interval and across a large geographic region.

I consider this manuscript appropriate for publication by *PLOS One* because it addresses far-reaching questions and offers integrative methods that will be of broad interest and application to larger scientific communities, especially those interested in vertebrate paleontology,

paleoecology, reptile biology, evolutionary biology, functional morphology, and comparative anatomy.

This manuscript has not been published in any other peer-reviewed journal and is not under consideration for publication elsewhere. The submitted draft of the manuscript contains 12,871 words, seven figures, and four tables, in addition to seven supplementary figures, eleven supplementary tables, and two supplementary datasets.

I have no conflicts of interest to disclose. I have not had any prior discussions about this manuscript with a *PLOS One* Editorial Board Member.

Please see my "Response to Reviewers" letter for details on the changes I have made in this revised version of the manuscript.

Thank you very much for your consideration. I look forward to hearing from you.

Sincerely,

Sara J. ElShafie, Ph.D.
Alumnus, Department of Integrative Biology and Museum of Paleontology
University of California, Berkeley
selshafie@berkeley.edu

Click here to access/download;Manuscript;Manuscript.docx

Body size estimation from isolated fossil bones reveals deep time evolutionary trends ~~for~~ lizards

Sara J. ElShafie

Department of Integrative Biology and Museum of Paleontology, University of California, Berkeley, California, United States of America

E-mail: selshafie@berkeley.edu

Abstract

Lizards play vital roles in extant ecosystems. However, their roles in extinct ecosystems are poorly understood because the fossil record of lizards consists mostly of isolated bones. This makes it difficult to document changes in lizard morphology and body size over time, which is essential for studies of lizard paleoecology and evolution. It is also difficult to compare available fossil lizard data with existing extant lizard datasets because extant studies rarely measure individual bones. Furthermore, no previous study has regressed measurements of individual bones to body length across crown lizard groups, nor tested those regressions on fossil skeletons. An extensive dataset of extant lizard measurements across crown taxonomic groups is provided here, including individual bones, from which methods for estimating lizard body size from isolated fossil elements were derived. These methods were applied to a comparably large dataset of fossil lizard specimens from the robust Paleogene record (66-23 Mya) of the Western Interior of North America. This study tests the hypothesis that anatomical proportions have been conserved within higher-level crown lizard groups since the Paleogene and can therefore be used to reconstruct snout-vent length (SVL) for fossil specimens referred to the same groups.

Individual bones were regressed to SVL in each extant lizard group and the regressions were tested using measurements from complete extant specimens as well as some rare fossil skeletons. The regressions predicted actual SVL for both the extant and fossil test specimens with similar accuracy. The resulting body size estimates for the entire fossil dataset revealed that lizards reached greatest maximum body size ~~for this study period~~ in the middle Paleogene, with the largest size class dominated by anguid lizards that exceeded 1 meter in SVL and 1 kg in body mass. Maximum body size decreased to under 400 mm in the late Paleogene. This is the first study to investigate body size evolution across lizard clades over a deep time interval and for a

large geographic region. The proposed methods can provide estimates of SVL for fossil lizards referred to any crown group.

Introduction

Body size influences every aspect of a vertebrate's physiology and life history [1–4]. It is therefore essential to estimate body size for fossil vertebrate taxa in order to understand the ecological and evolutionary context of any vertebrate group [5–10]. Lizards are a particularly important group of vertebrates to investigate in both extant and extinct ecosystems because they exhibit high taxonomic and morphological diversity and occupy ~~myriad~~ ecological roles [4,11–14]. For example, lizards make up the majority of tetrapods in some harsh environments, especially deserts; can maintain high population densities in small areas; and have high dispersal abilities [15–19]. Lizards also regulate populations of insects, rodents, and other prey on which they feed, and they are an important food source for many animals [4,12,13,20].

The lizard fossil record consists mostly of isolated elements that can be difficult to identify to the level of genus or even family. Therefore, body size for fossil lizards must be inferred primarily from isolated bones [21]. To further complicate matters, anatomical datasets of extant lizards rarely include measurements of individual cranial or limb bones for comparison. Most relevant previous studies only recorded measurements of head length and SVL across extant lizard groups [3,4,11,12,19,22–26]. Hence, no prior study has reconstructed body size in lizards on evolutionary time scales across higher-level taxonomic groups. ~~This study addresses this need by presenting~~ an extensive reference dataset of individual anatomical element measurements taken across crown group lizard lineages and ~~detailing~~ methods for using those

data to estimate body size in fossil lizards. These methods are used to reconstruct body size in lizard lineages over a deep time interval and across a continental interior.

It is not unambiguously clear which variable is the best proxy for body size in fossil vertebrates. Some previous studies estimated mass as a proxy for body size in fossil crocodyliforms using scaling relationships in extant taxa between body mass and femoral length and circumference [27] and head width [28], since these anatomical measurements are commonly available in the crocodyliform fossil record. Body mass has also been estimated for dinosaurs [7] and fossil mammals [6,29,30] using similar methods.

However, body mass is only weakly correlated with most individual bones in many fossil vertebrates; thus, such methods can be difficult to apply across a fossil record of varied isolated bones [6]. For fossil reptiles, body length is easier to estimate from individual bones than body mass because length correlates tightly with other anatomical measurements. Reptile body length has been shown to correlate with specific cranial bones (e.g., $R^2 = 0.89$ for dentary length in acrodontan lizards [9]; $R^2 \geq 0.78$ for various cranial elements in the lizard genera *Anolis* and *Pholidoscelis* [31]), head length (e.g., $R^2 \geq 0.97$ for extant crocodylians [32]), head width (e.g., $R^2 \geq 0.85$ for extant crocodylians [28]), femur length (e.g., $R^2 \geq 0.99$ for extant crocodylians [27]), and vertebral element width (e.g., $R^2 \geq 0.81$ for extant boine snakes [33]). Mandible length has also been used as a direct proxy for body size in fossil lizards [21,34].

Snout-vent length (SVL), measured from the tip of the snout to the cloacal opening at the base of the tail (Fig 1), is often the preferred measure of body length in extant lizard studies [22,31]. This is because many lizards can autotomize and even regenerate their tails [35–39], thus lizard tail measurements are highly variable. Total body length is also difficult to reconstruct in lizards because the tail rarely possesses its original length in any extant specimen

and is often disarticulated in dry skeletonized specimens. Measurements of SVL are more feasible to obtain from wet preserved or dry skeletonized extant lizard specimens. Maximum SVL strongly correlates with mean adult SVL and SVL at sexual maturity in extant lizards [3,40], so maximum SVL is a good metric of mean adult body size for a population of lizards sampled from the fossil record. Furthermore, once SVL is estimated, lizard body mass can be calculated from family-specific SVL-to-mass equations [24,41,42].

Fig 1. Measurement of snout-vent length (SVL). SVL was measured from the tip of the snout at the premaxilla to the posterior edge of the centrum on the second caudal vertebra, which is the approximate position of the middle of the cloaca (vent). SVL measurement is indicated by the blue arrow. Scale bar = 1 cm. Specimen pictured is *Gerrhonotus infernalis* (Anguidae; FMNH 22452).

For this study, lizards were sampled from the Paleogene record (23-66 Mya) in the Western Interior of North America, specifically, from localities concentrated in the United States (Fig 2). The Paleogene is an interesting period to study because it spans significant warming and cooling events [43,44] and chronicles the radiation of mammals [45–55] and later squamates [56–60] following the End-Cretaceous Mass Extinction. This timeframe was used here as a study system because it has a prolonged and robust fossil record in the Western Interior, preserving many diverse and ecologically important extant lizard clades that still occur in the area. These include anguids and pleurodontan iguanians (e.g., iguanids and dactyloids), as well as clades that were extirpated from the region during the Paleogene, such as varanids and shinisaurids. Natural

history museum collections in the United States include many Paleogene lizard specimens identified to the level of family or even genus.

Fig 2. Map showing all localities for fossil lizard data from across the Western Interior of North America through the Paleogene. Featured area is highlighted in inset map. Data points represent only specimens for which the locality could be georeferenced. Some taxonomic groups occurring with others in a particular locality may not be visible. Base maps were made with Natural Earth and are reprinted from Natural Earth under a CC Public Domain license, original copyright 2009. All other data contained were collected by the author. Maps were generated using QGIS-LTR 3.22.6 (download.qgis.org). The figure created is under the CC-BY 4.0 license, as determined by the authors of QGIS.

This study tested the hypothesis that proportions of individual bone lengths to SVL are conserved within each lizard lineage and that these proportions can therefore be used to predict SVLs within crown squamate groups (exclusive of snakes) for fossil lizard specimens identified to those same groups. This hypothesis was tested using eight taxonomic lizard groups (seven families + Iguania – the latter encompassing the iguanian families, not all of which have clear taxonomic relationships [61,62]) that are represented in the Paleogene fossil record and still exist today. For each crown group represented, regressions of individual cranial and limb bones to SVL were generated. Both extant and fossil lizard skeletons were used as test specimens to validate the regressions. The equations were then used to reconstruct body size for crown group lizards sampled from across the U.S. Western Interior through the Paleogene. This is the first study to reconstruct lizard body size evolution in different crown groups over a prolonged

geologic interval and across a continental interior. ~~The results have broad implications for modes of body size evolution and resource zone partitioning in lizards. In addition to applications for fossil research, the extensive dataset and body size estimation methods presented here can be of great use to functional morphologists and herpetologists.~~

Institutional abbreviations

AMNH, American Museum of Natural History, New York, NY; **CAS**, California Academy of Sciences, San Francisco, CA; **CJB**, Christopher J. Bell, University of Texas at Austin, Austin, TX; **CM**, Carnegie Museum of Natural History, Pittsburgh, PA; **DMNH**, Denver Museum of Natural History (Denver Museum of Nature and Science), Denver, CO; **FLMNH**, Florida Museum of Natural History, Gainesville, FL; **FMNH**, Field Museum of Natural History, Chicago, IL; **FOBU**, Fossil Butte National Monument, Kemmerer, WY; **GCVP**, Georgia College Vertebrate Paleontology, Milledgeville, GA; **LACM**, Natural History Museum of Los Angeles County, Los Angeles, CA; **MCZ**, Museum of Comparative Zoology, Cambridge, MA; **MSU**, Michigan State University, Lansing, MI; **MVZ**, Museum of Vertebrate Zoology, Berkeley, CA; **NMMNH**, New Mexico Museum of Natural History, Albuquerque, NM; **PTRM**, Pioneer Trails Regional Museum, Bowman, ND; **RAM**, Raymond M. Alfe Museum of Paleontology, Claremont, CA; **SMM**, Science Museum of Minnesota, Saint Paul, MN; **TxVP**, Texas Vertebrate Paleontology Collection, The University of Texas, Austin, TX; **UCM**, University of Colorado Museum, Boulder, CO; **UCMP**, University of California Museum of Paleontology, Berkeley, CA; **UMMP**, University of Michigan Museum of Paleontology, Ann Arbor, MI; **UNSM**, University of Nebraska State Museum, Lincoln, NE; **USNM**, United States National Museum (Smithsonian National Museum of Natural History), Washington, DC; **UW**,

University of Wyoming Geological Museum, Laramie, WY; **UWBM**, University of Washington Burke Museum, Seattle, WA; **YPM**, Yale Peabody Museum, New Haven, CT.

Materials & methods

Data collection

Anatomical measurements were collected from 283 fossil lizard specimens, including 44 recognized genera and at least 33 recognized species, from the Paleogene record of the U.S. Western Interior (Table 1, S1 Dataset). Not all specimens that were attributed to a genus could be attributed to a specific species. Only specimens that included complete cranial or limb elements (e.g., Fig 3) were sampled. A few specimens included complete skulls (Fig 3A) or even skeletons (Figs 4 and 5). The fossil specimens were grouped by North American Land Mammal Age (NALMA), which compose a relative chronology used to divide the Paleogene based on taxonomic turnover in the mammal fossil record [63]. Age ranges for these intervals are listed in Table 2 based on Barnosky et al. [64].

Table 1. Summary of datasets. The extant species totals include subspecies. Fossil species totals do not include specimens that could not be identified to a specific genus or species (e.g., Anguidae indet., *Glyptosaurus sp.*). For example, all three fossil teiid specimens sampled were identified as only "Teiidae indet."

Taxonomic group	# Extant specimens sampled	# Extant genera sampled	# Extant species sampled	# Fossil specimens sampled	# Fossil genera sampled	# Fossil species sampled

**Anguidae**	66	4	17	218	16	17
**Varanidae**	47	1	17	6	1	1
**Teiidae**	33	5	11	3	0	0
**Scincidae**	28	8	19	4	3	1
**Xantusiidae**	20	1	3	19	2	2
**Xenosauridae**	17	1	3	7	4	3
**Iguania**	55	9	16	19	8	6
**Shinisauridae**	N/A	N/A	N/A	7	3	3
**Total**	**266**	**29**	**86**	**283**	**37**	**33**

Fig 3. Paleogene anguimorph lizards from the Western Interior of North America and extant morphological analogues. (A) *Peltosaurus granulosus*† (Anguidae) skull in right lateral view, and B) right humerus in medial view (AMNH 42913), indicated by arrow, Orellan. (C) *Gerrhonotus infernalis* (Anguidae) skull and right mandible in right lateral view and (D) left humerus in medial view (FMNH 22452), extant. A-D are oriented with anterior to the right. (E) *Heloderma suspectum* (Helodermatidae) skull and left mandible in left lateral view, and (F) right humerus in medial view (UCMP 117512), extant. (G) *Helodermoides tuberculatus*† skull, mandibles, and cervical osteoderms in right lateral view, reflected for continuity (USNM V 13869), Chadronian. (H) *Helodermoides tuberculatus*† (Anguidae) left mandible in lateral view, and (I) right humerus in medial view, reflected for continuity (UNSM 4511), Chadronian. E-I are oriented with anterior to the left. Scale bar = 1 cm. USNM V 13869 (G) image courtesy of the Smithsonian Institution.

Fig 4. Paleogene varanids from the Western Interior of North America. (A) *Saniwa ensidens*† skeleton (FMNH PR 2378), Wasatchian. Scale bar = 10 cm. (B) *Saniwa sp.*† dentaries, vertebrae, and fragments (DMNH EPV.34588), Bridgerian. Scale bar = 3 cm.

Fig 5. Other complete Paleogene lizard skeletons from the Western Interior of North America. (A) *Bahndwivici ammoskius*† (Shinisauridae) skeleton (FMNH PR 2260), Wasatchian. Scale bar = 3 cm. (B) *Afairiguana avius*† (Iguania; Polychrotidae) skeleton (FMNH PR 2379), Wasatchian. Scale bar = 3 cm.

Table 2. Lizard crown group diversity by NALMA interval. Expected diversity based on the Paleobiology Database (PBDB; www.paleobiodb.org) is provided for comparison but should be taken as a coarse estimate because there may be some identification error in PBDB entries. Entries that were deemed inaccurate based on literature review were disregarded. Some data are also missing from the PBDB (e.g., no specific varanid genera or species are listed for the Paleogene within the United States). Iguanian specimens are identified by specific family rather than as "Iguania" in the PBDB, so comparison for that group was not possible. This table does not represent the full taxonomic diversity of Paleogene lizards represented in the U.S. Western Interior fossil record; it only represents groups for which complete fossil cranial or limb bones were measured. Fossil genera and species totals per family per NALMA do not include specimens that could not be identified to a specific genus or species, respectively (e.g., Anguidae indet.; *Glyptosaurus* sp.). Hence, some genera totals are zero. Specimens tentatively referred to a specific genus or species (e.g., *c.f. Glyptosaurus*) are counted toward that genus and/or species. NALMA age ranges are from Barnosky et al. [64].

**Epoch**	**NALMA (Mya)**	**Lizard crown groups sampled**	**# of recognized genera sampled / expected from PBDB**	**# of recognized species sampled / expected from PBDB**
Paleocene	Puercan (66.0 – 63.8)	Anguidae	1 / 2	1 / 2
		Scincidae	1 / 0	1 / 0
	Torrejonian (63.8 – 60.9)	Anguidae	2 / 4	2 / 5
		Varanidae	1 / 0	0 / 0
		Xantusiidae	1 / 1	1 / 1
	Tiffanian (60.9 – 56.2)	Anguidae	3 / 4	3 / 5
		Scincidae	1 / 0	0 / 0
		Shinisauridae	1 / 0	1 / 0
		Xenosauridae	1 / 0	1 / 0
Paleocene / Eocene	Clarkforkian (56.2 – 54.9)	Anguidae	3 / 0	3 / 0
Eocene	Wasatchian (54.9 – 50.5)	Anguidae	10 / 10	8 / 14
		Iguania	4 / 0	3 / 0
		Scincidae	1 / 0	0 / 0
		Shinisauridae	2 / 1	2 / 1
		Varanidae	1 / 0	1 / 0
		Xantusiidae	1 / 2	0 / 4
		Xenosauridae	2 / 0	1 / 0
	Bridgerian (50.5 - 46.2)	Anguidae	4 / 8	2 / 7
		Iguania	1 / 0	0 / 0

		Teiidae	0 / 0	0 / 0
		Varanidae	1 / 0	0 / 0
		Xantusiidae	2 / 1	1 / 1
		Xenosauridae	1 / 0	0 / 0
	Uintan (46.2 - 39.7)	Anguidae	1 / 4	0 / 6
		Shinisauridae	1 / 0	0 / 0
		Varanidae	1 / 0	1 / 0
	Duchesnean (39.7 - 37.0)	Anguidae	0 / 0	0 / 0
	Chadronian (37.0 - 33.9)	Anguidae	3 / 2	3 / 2
		Iguania	2 / 0	2 / 0
		Xantusiidae	1 / 0	1 / 0
Oligocene	Orellan (33.9 - 31.8)	Anguidae	2 / 1	2 / 1
		Iguania	1 / 0	1 / 0
		Xenosauridae	1 / 1	1 / 1
	Whitneyan (31.8 - 29.5)	Anguidae	1 / 0	1 / 0
	Arikareean (29.5 - 23.0)	Anguidae	1 / 1	1 / 1

To compare the fossil lizard specimens with extant congeners, 266 extant specimens were measured (S2 Dataset), comprising 30 genera and 86 species (Table 1) from the same crown group lineages that were represented in the fossil dataset. These included seven extant families:

Anguidae (e.g., alligator lizards and galliwasps), Scincidae (skinks), Shinisauridae (crocodile lizards), Teiidae (e.g., whip-tail lizards), Varanidae (monitor lizards), Xantusiidae (night lizards), and Xenosauridae (knob-scaled lizards). There were also several pleurodontan iguanian families represented in the fossil dataset (e.g., Iguanidae and Polychrotidae), so these were treated as a collective group, "Iguania," in both datasets. Phylogenetic relationships among iguanian families are poorly resolved, but the group "Iguania" is supported by both morphological and molecular analyses [61,62,65,66]. Extant data collection in this study focused on dry skeletonized specimens to obtain measurements of individual bones as well as SVL from the same specimens. Juvenile specimens were excluded from the extant dataset. Further details on data collection procedures and considerations are provided in Supporting Information.

No permits were required for the described study, which complied with all relevant regulations.

Generating and testing regressions

~~Researchers commonly use bivariate linear regressions to estimate SVL for fossil vertebrates using anatomical measurements from specimens of the most inclusive phylogenetic group [67].~~ This study regressed anatomical elements – including skull length, individual cranial bone length (dentary, mandible, maxilla, frontal, parietal), and limb bone length (humerus, femur, tibia) – to SVL for the extant lizard groups in the dataset presented (Table 3, S1-S2 Fig, S1-S7 Tables). Reduced major axis (RMA) linear regressions were used because RMA accommodates error in both the dependent and independent variable measurements [68]. All measurements were transformed using natural log, which is commonly applied in analyses of fossil vertebrate body size [9,29,49,52,53,69]. Tests of natural log compared to base 10 log

regressions also produced the same SVL prediction for large test specimens in this study, so natural log was used in all regressions for consistency. All regression analyses were performed using PAST v.4.03 [68].

Table 3. Regression functions and associated statistics for SVL estimation from individual bones. All functions were transformed using natural log (LN). Standard error for slope and intercept terms are listed after the slope and intercept in the equations. SEE = Standard error of the estimate. Functions are listed in order of increasing SEE for each group. P(uncorrelated) < 0.001 for all functions.

**Group**	**Predictor**	***n***	**Slope *b***	**Standard error of *b***	**Intercept**	**Standard error of intercept**	R^2	**SEE**
Anguidae	Skull Length	46	0.946	0.0417	1.71	0.129	0.91	0.0740
	Femur Length	43	0.888	0.0410	2.46	0.101	0.91	0.0742
	Mandible Length	43	0.921	0.0424	1.82	0.129	0.91	0.0760
	Humerus Length	41	0.889	0.0451	2.57	0.106	0.90	0.0815
	Parietal Length	24	1.03	0.0658	2.29	0.148	0.91	0.0853
	Maxilla Length	22	1.02	0.0803	2.40	0.170	0.88	0.0992
	Frontal Length	22	1.27	0.111	2.13	0.216	0.85	0.120
	Dentary Length	24	0.898	0.0816	2.43	0.195	0.82	0.121
Varanidae	Tibia Length	27	0.953	0.0282	2.43	0.103	0.98	0.0695
	Maxilla Length	25	1.13	0.0423	2.04	0.145	0.97	0.0837
	Dentary Length	23	1.13	0.0845	1.86	0.305	0.88	0.157
Xenosauridae	Dentary Length	12	0.36	0.164	0.921	0.450	0.85	0.0530
	Parietal Length	12	1.03	0.154	2.17	0.369	0.78	0.0661
	Frontal Length	12	1.05	0.185	2.16	0.435	0.69	0.0787

Teiidae	Dentary Length	26	0.677	0.0686	2.70	0.165	0.75	0.0995
Scincidae	Maxilla Length	27	1.06	0.0352	2.20	0.0850	0.97	0.115
	Dentary Length	27	1.12	0.0426	1.74	0.115	0.96	0.133
Xantusiidae	Maxilla Length	20	0.698	0.0659	2.82	0.0944	0.88	0.119
	Dentary Length	20	0.787	0.0869	2.58	0.134	0.89	0.124
Iguania	Skull Length	53	1.15	0.0248	1.06	0.0838	0.98	0.0997
	Maxilla Length	55	1.07	0.0303	2.07	0.0824	0.96	0.133
	Dentary Length	55	1.22	0.0402	1.38	0.118	0.94	0.155

To test the assumption that anatomical proportions are conserved between extant and extinct members of the lizard lineages represented in this study, the ~~regressions were tested~~ using complete skeletons of both extant and extinct taxa. The fossil test specimens included three of the few available complete fossil lizard skeletons that were measured: a large-bodied varanid (*Saniwa ensidens*, FMNH PR 2378, SVL 425 mm which is smaller than the largest extant varanid measured in this study (*V. exanthematicus*, 770 mm); Fig 4A), a small-bodied anguid (*Parophisaurus pawneensis*, YPM VP 060609, SVL 138 mm; ~~not yet published at the time of this publication and thus not pictured here),~~ and a very small-bodied polychrotid iguanian (*Afairiguana avius*, FMNH PR 2379, SVL 46 mm; Fig 5B). The only known complete skeleton of a fossil shinisaurid, *Bahndwivici ammoskius* (FMNH PR 2260, SVL 154 mm; Fig 5A) was also measured and used to estimate SVL for the other fossil shinisaurid specimens based on anatomical ratios because complete dry skeletonized extant specimens for Shinisauridae were not available. See Supporting Information for more details on materials and methods.

Estimating body mass for fossils

Body mass scales linearly with SVL in extant lizards with limbs [15,24]. Other researchers developed family-level SVL-to-mass allometric equations for extant lizards (S8 Table) [24,41,42] as well as general SVL-to-mass equations for all lizards (RMA: Body Mass in kg = (((3.088±0.067)*SVL) – (4.852±0.128))/1000, R^2 = 0.946) [24]. Body mass for each fossil specimen in the dataset presented here (S1 Dataset) was calculated using both the family-level (S8 Table) and general RMA equations from these published studies for comparison.

New equations were also generated for estimating lizard body mass directly from individual bones (S1-S7 Tables). This was done by combining the equations generated in this study for estimating SVL from individual bones (S1-S7 Tables) with the equations published elsewhere for estimating lizard body mass from SVL (S8 Table). ~~The resulting equations produced the same body mass estimates for the fossil lizards as the estimates obtained from the previously published equations (S1 Dataset).~~

Results

Estimating body size of fossil lizards

Skull and mandible length consistently provided reliable regressions for estimating SVL (SEE ≤ 0.0997) across relevant taxonomic groups represented in this study (Table 3, S1-S7 Tables). These measurements are known to correlate with SVL widely among extant lizards [12,21,22]. Regressions based on limb measurements (femur, humerus, or tibia length) were also consistently accurate across groups (SEE ≤ 0.0815). Regressions based on dentary, maxilla, frontal, and parietal length had the greatest variation in rankings among lizard groups (SEE

0.0530 – 0.1571). Cranial element morphology often varies between closely related lizard groups, more so than proportions of head or limb length to SVL [70]. This may be the reason why attempts to calculate general regression equations for this study proved unsuccessful: none of the general equations passed tests for both homoskedasticity and normal distribution of residuals (S9 Table). Meiri's [24] general equation for predicting mass from SVL for lizards (see S1 Dataset) was more viable likely because the relationship between SVL and mass appears to be more consistent than the relationship between head length or any individual element to SVL across lizard taxonomic groups.

The group-level regressions generated in this study predicted actual SVL with comparable accuracy between the extant and fossil test specimens. The actual SVL for each extant and fossil test specimen fell within or close to the 95% confidence interval for the estimated SVL (S1-S7 Tables). The percentage difference between predicted and actual SVL of test specimens ranged from 1% (varanid tibia length vs. SVL, SEE = 0.0695; S2 Table) to 36% (anguid frontal length vs. SVL, SEE = 0.120; S1 Table). Clearly, some anatomical elements provide more accurate estimates than others for different lizard groups.

Taxonomic turnover

Anguids are known to be the most abundant lizards in the Paleogene fossil record of the Western Interior [71–73] and that was reflected in this study by their unrivaled taxonomic richness, which included at least 15 genera (Figs 6-7, Table 2, S1 Dataset). The results presented here also indicated that taxonomic diversity was greatest in the early Eocene and decreased in the early Oligocene (Figs 6-7, Table 2). Rarefaction analysis indicated that the number of crown groups recovered within a given NALMA peaked around a sample size of 50 specimens (S3

Fig). The Orellan sample included 64 specimens (Fig 6, S1 Dataset) and yet recovered only three crown groups (Table 2, S3 Fig), supporting the interpretation of a decline in diversity in the late Paleogene.

Fig 6. Snout-vent length distribution by taxonomic group for fossil lizards in the Western Interior of North America through the Paleogene. Sample sizes are per North American Land Mammal Age (NALMA). Data points are arbitrarily spread within each NALMA for visibility. NALMA abbreviations: Pu = Puercan, To = Torrejonian, Ti = Tiffanian, Cf = Clarkforkian, Wa = Wasatchian, Br = Bridgerian, Ui = Uintan, Du = Duchesnean, Ch = Chadronian, Or = Orellan, Wh = Whitneyan, Ar = Arikareean. Data points marked with an asterisk (*) were measured from complete fossil skeletons. All others were estimated from individual cranial or limb elements using regressions (see S1-S7 Tables). All measurements are transformed using natural log.

Fig 7. Body mass distribution by taxonomic group for fossil lizards in the Western Interior of North America through the Paleogene. Sample sizes are per North American Land Mammal Age (NALMA). Data points are arbitrarily spread within each NALMA for visibility. NALMA abbreviations: Pu = Puercan, To = Torrejonian, Ti = Tiffanian, Cf = Clarkforkian, Wa = Wasatchian, Br = Bridgerian, Ui = Uintan, Du = Duchesnean, Ch = Chadronian, Or = Orellan, Wh = Whitneyan, Ar = Arikareean. Masses were calculated with snout-vent length using the published equations listed in S8 Table. Data points marked with an asterisk (*) used SVLs measured from complete fossil skeletons. All others used SVLs estimated from individual cranial or limb element lengths using regressions (see S1-S7 Tables). All measurements are transformed using natural log.

The observed patterns may reflect some taphonomic or preservation bias (especially with respect to smaller bones), gaps in the rock record, and selective sampling by fossil collection crews. However, rarefaction analysis (S3 Fig) indicated that this study adequately sampled the upper range of body size diversity represented in this fossil record, especially from thoroughly documented areas like the Bighorn and Green River Basins (S1 Dataset; for further discussion, see the censuses of microvertebrate localities in this system by Smith [56–60]). Preservation bias toward complete elements from adult individuals may have mitigated other sources of bias when reconstructing maximum body size through geologic intervals.

The observed late Paleogene reductions in maximum body size represent the extinction of larger taxa [74,75]. Even in extant lizards, extinction risk increases with body size [3]. Among the eight lizard groups documented in this study from the Paleogene record of this region, only Anguidae, Scincidae, Teiidae, and Xantusiidae still occur in the area. Xenosaurids and the iguanians included in this study that occurred in the Western Interior during the Paleogene have since been extirpated from the region; varanids and shinisaurids no longer occur anywhere in North America [4,11,13,76]. For further discussion, see Supporting Information.

Body size evolution in fossil lizards

Maximum lizard SVL remained below 300 mm through the early and middle Paleocene (Puercan – Tiffanian, Fig 6, Table 4, S1 Dataset). This upper SVL limit increased to almost 500 mm and maximum body mass exceeded 1 kg around the Paleocene-Eocene transition (Clarkforkian; Fig 7). By the Wasatchian, maximum SVL reached 750 mm, and surpassed 1 m by the Bridgerian (Table 4).

Table 4. Maximum lizard body size estimates per NALMA interval. NALMA age ranges are from Barnosky et al. [64].

NALMA	Age Range (Mya)	Crown Group	Binomial	Specimen	Max SVL Estimate (mm)	Mass Estimate (kg)
Puercan	66.0-63.8	Anguidae	*cf. Odaxosaurus piger*	UCM 34991	91	0.311
Torrejonian	63.8-60.9	Varanidae	cf. Varanidae indet.	UCMP 2671	191	0.615
Tiffanian	60.9-56.2	Anguidae	*Melanosaurus sp.*	UCM 98615	256	0.885
Clarkforkian	56.2-54.9	Anguidae	*Melanosaurus maximus*	UMMP 74618	487	1.69
Wasatchian	54.9-50.5	Anguidae	*Melanosaurus sp.*	UCMP 154536	788	2.74
Bridgerian	50.5-46.2	Anguidae	*Glyptosaurus sylvestris*	USNM 12590	1056	3.67
Uintan	46.2-39.7	Varanidae	*Saniwa ensidens*	FMNH UC 1719	540	1.74
Duchesnean	39.7-37.0	Anguidae	Anguidae indet.	CM 42469	116	0.399
Chadronian	37.0-33.9	Anguidae	*Helodermoides sp.*	UW 11057	1296	4.51
Orellan	33.9-31.8	Anguidae	*Peltosaurus sp.*	UCM 20877	360	1.25
Whitneyan	31.8-29.5	Anguidae	*Peltosaurus sp.*	SMM P81.8.71	224	0.775

Arikareean	29.5-23.0	Anguidae	*Peltosaurus sp.*	UNSM 81001	351	1.22

~~The pattern for the middle Eocene (Uintan – Duchesnean) was dubious.~~ The observed maximum body size for the middle Eocene (540 mm, 1.74 kg, *Saniwa ensidens*, Figs 6-7, Table 4) was much lower than the early or late Eocene. Fewer outcrops are available from this interval in the U.S. Western Interior, so this result was likely an artifact of poor sampling (n = 4 for the Uintan, n = 1 for the Duchesnean, Fig 6). Rarefaction analysis (S4 Fig) demonstrated that the number of expected size groups recovered in a sample from this fossil dataset again peaked and leveled off around n = 50, supporting the assumption that the NALMAs with small sample sizes in this study did not capture the full range of lizard body size that was likely present during those intervals.

The sample size was higher for the late Eocene (n = 24 for the Chadronian, Fig 6). The Chadronian record included several large individuals, including the largest body size estimated in this study (1.3 m, 4.5 kg, *Helodermoides sp.*, Table 4). By the early Oligocene (Orellan), maximum SVL dropped to 360 mm (*Peltosaurus sp.*) and remained below that limit through the late Paleogene. The Orellan was very well sampled (n = 64) compared to other NALMAs, and consequently, sample size did not correlate strongly with maximum estimated SVL across the dataset (R^2 = 0.23, p(uncorr.) = 0.11). ~~Together with rarefaction analysis (S4 Fig), these results indicate that the observed decrease in maximum lizard SVL in the late Paleogene is likely valid.~~

Discussion

Climate as a potential driver of Paleogene lizard body size evolution

Environmental temperature can influence body size evolution in reptiles. Metabolic rate increases with temperature and scales with body size in all poikilothermic vertebrates [1,77,78], including lizards [15,79]. Changes in environmental temperature are known to correlate globally with changes in maximum body size in extant lizards (exclusive of snakes [25]). Increased temperatures around the Paleocene-Eocene Thermal Maximum (PETM, 56 Mya; [44,80]) could have elevated metabolism and released a physiological constraint on maximum size for some lizard taxa. Gigantism has been documented in other fossil reptiles in conjunction with elevated temperatures, including a boid snake (*Titanoboa*, total length 13 m, 60-58 Mya, Colombia [33]), a caimanine crocodyliform (*Purussaurus*, total length 12.5 m, 8 Mya, northern South America [81]), and a varanid lizard (*Varanus priscus*, total length up to 5.7 m, around 50 kya, Australia [82,83]).

To investigate this possibility, RMA linear regression was applied to test whether maximum and mean fossil lizard SVL predicted global temperature per NALMA (S5 Fig, S10 Table). Global temperature data were obtained from Zachos et al. [43,44], a widely used dataset of Cenozoic paleotemperatures derived from marine oxygen isotopes (e.g., [10,49]). Here, fossil lizard body size showed no relationship to global temperature through the Paleogene. It is possible that the relationship observed today is not detectable across scales of millions of years. It might also be more pertinent to compare these data with terrestrial paleotemperature proxies from the same geotemporal system sampled in this study. This could be a fruitful area for continued research to build on the data presented here.

Alternatively, body size evolution patterns observed here could reflect indirect effects of climate [84]. Temperatures were warmer during both the summer and winter months around the PETM, and most precipitation came during the summer [85–87]. This would have allowed

lizards to be active and growing during a longer portion of the year [84,88] and could also have led to greater food availability from increased productivity, especially in the summer months [89–91]. Previous studies found elevated growth rates in extant tropical lizards occupying areas that received greater precipitation [92]. Attaining large body size in a closed tropical forest system with an equable climate, like that present in the Western Interior in the early Eocene [89,90,93,94], would also have allowed lizards to spend less time and energy on active thermoregulation [1,95]. Large body size sometimes corresponds with thermoconforming behavior and nocturnal activity in extant lizards [3,12,88], and these strategies could have reduced competition with diurnal organisms.

Herbivory can also lead to large body size in lizards [3,4,79]. This has been inferred for at least one giant fossil lizard [9]. However, the dentition of the largest lizards in this study were more indicative of omnivorous or insectivorous (Anguidae, Fig 3A) and carnivorous diets (Varanidae, Fig 4B; see [21,96]). It may be impossible to determine the specific mechanisms of gigantism in lizards in this system, but the results presented here elucidate clear patterns of body size evolution.

Body size evolution among taxonomic groups

Despite increased environmental temperatures in the early Eocene, most of the lizards in this geotemporal system did not experience an increase in body size range. Small-bodied lizards dominated the North American Paleogene lizard record. The maximum and mean body sizes for most lizard groups did not exceed 200 mm in SVL (LN(SVL) = 5.30, S6 Fig, S11 Table) or 1 kg in body mass (LN(Mass) = 0.00, S7 Fig, S11 Table). Maximum body size in extant lizards can stay comparatively low in warm climates due to behavioral thermoregulation [95], competition,

predation, or specializations related to resource zones of small taxa [97,98]. The results presented here may reflect an ecological hierarchy in the Paleogene communities of the Western Interior similar to extant ecosystems in which large-bodied lizards are less abundant [3].

Among the eight lizard crown groups sampled, only two reached SVLs over 200 mm and masses over 1kg: Anguidae and Varanidae (Figs 6-7, S6-S7 Figs, Table 4). Interestingly, the largest extant limbed anguid today, *Diploglossus millepunctatus* (maximum SVL 280 mm), does not approach the size of the largest anguids from the Eocene, nor do the largest lizards occurring in North America today (*Heloderma horridum*, maximum SVL 520 mm [99], Fig 3E; *Iguana iguana*, maximum SVL 580 mm [4]). Conversely, Varanidae includes both the largest extant lizard (*Varanus komodoensis*, which exceeds the adult size of the largest Eocene anguid at around 1.5 m SVL [4,76]) and the largest known terrestrial lizard in the fossil record (*Varanus priscus*, known from Pleistocene deposits in Australia, with SVL at least 3 m [82,83]), but varanids are now restricted to the Eastern Hemisphere [13].

Conclusions

This study offers the first survey and reconstruction of body size across crown lizard groups through a prolonged geologic interval and across a large continental interior region. The findings indicate that lizard body size peaked in the early Eocene. Only two lizard groups, Anguidae and Varanidae, reached large body sizes exceeding 1 m in SVL and 1 kg in mass. Body size range did not change considerably for other groups. Both maximum body size and taxonomic diversity decreased for lizards across the Western Interior in the early Oligocene. The methods presented here can be applied to other lizard clades to generate reasonably accurate

body size estimates for fossil taxa and to study patterns of body size evolution across higher taxonomic groups.

Acknowledgments

The author wishes to thank K. Padian, J. McGuire, A. Barnosky, and I. Wang for their mentorship and helpful comments on this work, as well as J. Head, who mentored the research that started this project. The following individuals provided assistance and access to museum collections: M. Norell, C. Mehling, J. Meng, D. Kizirian, L. Vonnahme, M. Arnold (AMNH); J. Vindum, L. Scheinberg, E. Ely (CAS); M. Lamanna, J. Padial, A. Henrici, S. Rogers (CM); T. Lyson, L. Ivy, K. MacKenzie (DMNH); J. Bloch, R. Hulbert, J. Bourque (FLMNH); P. Makovicky, L. Grande, A. Resetar, W. Simpson, A. Stroupe, J. Mata (FMNH); A. Aase (FOBU); N. Smith, S. McLeod, V. Rhue (LACM); J.Rosado (MCZ); J. McGuire, C. Spencer (MVZ); T. Williamson (NMMNH); A. Farke, G. Santos (RAM); A. Hastings, E. Whiting (SMM); C. Bell, M. Brown (TMM); T. Culver, N. Ridgwell (UCM); P. Holroyd, C. Marshall (UCMP); J. Wilson, A. Rountrey (UMMP); R. Hunt, Jr., P. Freeman, G. Corner, T. Labedz (UNSM); H. Dieter-Sues, M. Carrano, M. Brett-Surman, A. Millhouse (USNM); L. Vietti (UW); C. Sidor, R. Eng, M. Rivin (UWBM); J. Gauthier, C. Norris, D. Brinkman (YPM). The author thanks the following for helpful discussions: C. Badgley, C. Bell, B-A. Bhullar, J. Bloch, W. Clemens[†], C. Crumly, J. Gauthier, W. Gearty, P. Gingerich, L. Grande, A. Gunderson, P. Holroyd, E. Holt, R. Huey, R. Hunt, Jr., M. Kemp, D. Meyer, D. Miles, J. McGuire, J. Parham, H. Petermann, R. Secord, M. Stocker, R. Sullivan, D. Watkins, L. Weaver, S. Werning, E. Whiting, C. Williams, the MVZ Herp Group, the entire UCMP community. C. Bell also provided access to obscure literature. D. Meyer provided measurements of an unpublished specimen. G. Brown (UNSM) prepared a new *Peltosaurus* specimen (UNSM 12102) containing critical limb material. F. Vasconcellos provided the photo in Fig 3E. B. Chelemedos and C. Wong assisted with data collection. E. Whiting provided instruction on using QGIS to make maps. J. Lipps helped to design a visually

accessible color palette for Fig 2. The author thanks C. Meloro, S. Scarpetta, and an anonymous reviewer for their constructive comments which greatly helped to improve the manuscript.

References

1. Peters RH. The Ecological Implications of Body Size. 1st ed. Beck E, Birks HJB, Connor EF, editors. New York, NY: Cambridge University Press; 1983.
2. Schmidt-Nielsen K. Scaling: Why is Animal Size So Important? Cambridge: Cambridge University Press; 1984.
3. Meiri S. Evolution and ecology of lizard body sizes. Glob Ecol Biogeogr. 2008;17: 724–734. doi:10.1111/j.1466-8238.2008.00414.x
4. Meiri S. Traits of lizards of the world: Variation around a successful evolutionary design. Glob Ecol Biogeogr. 2018;27: 1168–1172. doi:10.1111/geb.12773
5. Damuth J. Problems in estimating body masses of archaic ungulates using dental measurements. Body Size Mamm Paleobiol Estim Biol Implic. 1990;229: tables 16.9-16.10.
6. Figueirido B, Pérez-Claros JA, Hunt RM, Palmqvist P. Body mass estimation in amphicyonid carnivoran mammals: A multiple regression approach from the skull and skeleton. Acta Palaeontol Pol. 2011;56: 225–246. doi:10.4202/app.2010.0005
7. Campione NE, Evans DC. A universal scaling relationship between body mass and proximal limb bone dimensions in quadrupedal terrestrial tetrapods. BMC Biol. 2012;10. doi:10.1186/1741-7007-10-60
8. Thomas Goodwin H, Bullock KM. Estimates of body mass for fossil giant ground squirrels, genus *Paenemarmota*. J Mammal. 2012;93: 1169–1177. doi:10.1644/11-MAMM-A-312.3
9. Head JJ, Gunnell GF, Holroyd PA, Howard Hutchison J, Ciochon RL. Giant lizards occupied herbivorous mammalian ecospace during the Paleogene greenhouse in Southeast

Asia. Proc R Soc B Biol Sci. 2013;280. doi:10.1098/rspb.2013.0665
10. Godoy PL, Benson RBJ, Bronzati M, Butler RJ. The multi-peak adaptive landscape of crocodylomorph body size evolution. BMC Evol Biol. 2019;19: 1–29. doi:10.1186/s12862-019-1466-4
11. Vidan E, Novosolov M, Bauer AM, Herrera FC, Chirio L, Campos Nogueira C, et al. The global biogeography of lizard functional groups. J Biogeogr. 2019;00: 1–12. doi:10.1111/jbi.13667
12. Pianka ER, Vitt LJ, Pelegrin N, Fitzgerald DB, Winemiller KO. Toward a Periodic Table of Niches, or Exploring the Lizard Niche Hypervolume. Am Nat. 2017;190: 000–000. doi:10.1086/693781
13. Pianka ER, Vitt LJ. Lizards: Windows to the Evolution of Diversity. Berkeley: University of California Press; 2003.
14. Vitt LJ, Pianka ER, Cooper WE, Schwenk K. History and the global ecology of squamate reptiles. Am Nat. 2003;162: 44–60. doi:10.1086/375172
15. Pough FH. The advantages of ectothermy for tetrapods. Am Nat. 1980;115: 92–112. doi:10.1086/283547
16. Muñoz MM, Bodensteiner BL. Janzen's Hypothesis meets the Bogert Effect: Connecting climate variation, thermoregulatory behavior, and rates of physiological evolution. Integr Org Biol. 2019;1: 1–12. doi:10.1093/iob/oby002
17. Muñoz MM, Losos JB. Thermoregulatory behavior simultaneously promotes and forestalls evolution in a tropical lizard. Am Nat. 2018;191: E15–E26. doi:10.1086/694779
18. Muñoz MM, Stimola MA, Algar AC, Conover A, Rodriguez AJ, Landestoy MA, et al. Evolutionary stasis and lability in thermal physiology in a group of tropical lizards. Proc R

Soc B Biol Sci. 2014;281: 20132433. doi:10.1098/rspb.2013.2433

19. Roll U, Feldman A, Novosolov M, Allison A, Bauer AM, Bernard R, et al. The global distribution of tetrapods reveals a need for targeted reptile conservation. Nat Ecol Evol. 2017;1: 1677–1682. doi:10.1038/s41559-017-0332-2

20. Vitt E.R. Pianka, W.E. Cooper, Jr., and K. Schwenk LJ. History and the global ecology of squamate reptiles. Am Nat. 2003;162: 44–60.

21. Herrera-Flores JA, Stubbs TL, Benton MJ. Ecomorphological diversification of squamates in the Cretaceous. R Soc Open Sci. 2021;8: 1–11. doi:10.1098/rsos.201961

22. Metzger KA, Herrel A. Correlations between lizard cranial shape and diet: A quantitative, phylogenetically informed analysis. Biol J Linn Soc. 2005;86: 433–466. doi:10.1111/j.1095-8312.2005.00546.x

23. Olalla-Tárraga MÁ, Rodríguez MÁ, Hawkins BA. Broad-scale patterns of body size in squamate reptiles of Europe and North America. J Biogeogr. 2006;33: 781–793. doi:10.1111/j.1365-2699.2006.01435.x

24. Meiri S. Length-weight allometries in lizards. J Zool. 2010;281: 218–226. doi:10.1111/j.1469-7998.2010.00696.x

25. Slavenko A, Feldman A, Allison A, Bauer AM, Böhm M, Chirio L, et al. Global patterns of body size evolution in squamate reptiles are not driven by climate. Glob Ecol Biogeogr. 2019;28: 471–483. doi:10.1111/geb.12868

26. Villa A, Delfino M. A comparative atlas of the skull osteology of European lizards (Reptilia: Squamata). Zool J Linn Soc. 2019; 1–100. doi:10.1093/zoolinnean/zlz035

27. Farlow JO, Hurlburt GR, Elsey RM, Britton ARC, Langston W. Femoral dimensions and body size of *Alligator mississippiensis*: Estimating the size of extinct mesoeucrocodylians.

J Vertebr Paleontol. 2005;25: 354–369. doi:10.1671/0272-4634(2005)025[0354:FDABSO]2.0.CO;2

28. O'Brien HD, Lynch LM, Vliet KA, Brueggen J, Erickson GM, Gignac PM. Crocodylian Head Width Allometry and Phylogenetic Prediction of Body Size in Extinct Crocodyliforms. Integr Org Biol. 2019;1. doi:10.1093/iob/obz006

29. Egi N. Body mass estimates in extinct mammals from limb bone dimensions: The case of North American Hyaenodontids. Palaeontology. 2001;44: 497–528. doi:10.1111/1475-4983.00189

30. Valkenburgh B Van. Skeletal and dental predictors of body mass in carnivores. Body size in Mammalian Paleobiology: Estimation and biological implications. 1990. pp. 181–205.

31. Bochaton C, Kemp ME. Reconstructing the body sizes of Quaternary lizards using *Pholidoscelis Fitzinger*, 1843, and Anolis Daudin, 1802, as case studies. J Vertebr Paleontol. 2017;37: 9 pp. doi:10.1080/02724634.2017.1239626

32. Sereno PC, Larsson HCE, Sidor CA, Gado B. The giant crocodyliform *Sarcosuchus* from the cretaceous of Africa. Science (80-). 2001;294: 1516–1519. doi:10.1126/science.1066521

33. Head JJ, Bloch JI, Hastings AK, Bourque JR, Cadena EA, Herrera FA, et al. Giant boid snake from the Palaeocene neotropics reveals hotter past equatorial temperatures. Nature. 2009;457: 715–718. doi:10.1038/nature07671

34. Herrera-Flores JA, Elsler A, Stubbs TL, Benton MJ. Slow and fast evolutionary rates in the history of lepidosaurs. Palaeontology. 2022;65: 1–13. doi:10.1111/pala.12579

35. Arnold EN. Evolutionary aspects of tail shedding in lizards and their relatives. J Nat Hist. 1984;18: 127–169. doi:10.1080/00222938400770131

36. Bateman PW, Fleming PA. To cut a long tail short: A review of lizard caudal autotomy studies carried out over the last 20 years. J Zool. 2009;277: 1–14. doi:10.1111/j.1469-7998.2008.00484.x

37. Gilbert EAB, Payne SL, Vickaryous MK. The anatomy and histology of caudal autotomy and regeneration in lizards. Physiol Biochem Zool. 2013;86: 631–644. doi:10.1086/673889

38. Sanggaard KW, Danielsen CC, Wogensen L, Vinding MS, Rydtoft LM, Mortensen MB, et al. Unique Structural Features Facilitate Lizard Tail Autotomy. PLoS One. 2012;7. doi:10.1371/journal.pone.0051803

39. Zani PA. Patterns of caudal-anatomy evolution in lizards. J Zool. 1996;240: 201–230.

40. Greer AE. Distribution of Maximum Snout-Vent Length among Species of Scincid Lizards. J Herpetol. 2001;35: 383–395.

41. Meiri S, Bauer AM, Chirio L, Colli GR, Das I, Doan TM, et al. Are lizards feeling the heat?: a tale of ecology and evolution under two temperatures. Glob Ecol Biogeogr. 2013;22: 834–845.

42. Feldman A, Sabath N, Pyron RA, Mayrose I, Meiri S. Body sizes and diversification rates of lizards, snakes, amphisbaenians and the tuatara. Glob Ecol Biogeogr. 2016;25: 187–197. doi:10.1111/geb.12398

43. Zachos J, Pagani M, Sloan L, Thomas E, Billups K. Trends, rhythms and aberrations in global climate 65 Ma to present. Science (80-). 2001;292: 686–693.

44. Zachos JC, Dickens GR, Zeebe RE. An early Cenozoic perspective on greenhouse warming and carbon-cycle dynamics. Nature. 2008;451: 279–283. doi:10.1038/nature06588

45. Woodburne MO, Gunnell GF, Stucky RK. Climate directly influences Eocene mammal faunal dynamics in North America. Proc Natl Acad Sci. 2009;106: 13399–13403. doi:10.1073/pnas.0906802106

46. Gunnell GF, Zonneveld JP, Bartels WS. Stratigraphy, mammalian paleontology, paleoecology, and age correlation of the Wasatch Formation, Fossil Butte National Monument, Wyoming. J Paleontol. 2016;90: 981–1011. doi:10.1017/jpa.2016.100

47. Saarinen JJ, Boyer AG, Brown JH, Costa DP, Ernest M, Evans AR, et al. Patterns of maximum body size evolution in Cenozoic land mammals: eco-evolutionary processes and abiotic forcing. Proc R Soc B Biol Sci. 2014;281: 1–10.

48. Rose KD. Composition and Species Diversity in Paleocene and Eocene Mammal Assemblages: An Empirical Study. J Vertebr Paleontol. 1981;1: 367–388.

49. Lovegrove BG, Mowoe MO. The evolution of mammal body sizes: Responses to Cenozoic climate change in North American mammals. J Evol Biol. 2013;26: 1317–1329. doi:10.1111/jeb.12138

50. Lyson TR, Miller IM, Bercovici AD, Weissenburger K, Fuentes AJ, Clyde WC, et al. Exceptional continental record of biotic recovery after the Cretaceous−Paleogene mass extinction. Science (80-). 2019;366: 977–983. doi:10.1126/science.aay2268

51. Alroy J. Constant extinction, constrained diversification, and uncoordinated stasis in North American mammals. Palaeogeogr Palaeoclimatol Palaeoecol. 1996;127: 285–311.

52. Alroy J. Cope's Rule and the Dynamics of Body Mass Evolution in North American Fossil Mammals. Science (80-). 1998;280: 731–734.

53. Alroy J. New Methods for Quantifying Macroevolutionary Patterns and Processes. Paleobiology. 2000;26: 707–733.

54. Alroy J. Speciation and extinction in the fossil record of North American mammals. In: Butlin RK, Brindle JR, Schluter D, editors. Speciation and Patterns of Diversity. Cambridge University Press; 2009. pp. 301–323.

55. Huang S, Eronen JT, Janis CM, Saarinen JJ, Silvestro D, Fritz SA. Mammal body size evolution in North America and Europe over 20 Myr: Similar trends generated by different processes. Proc R Soc B Biol Sci. 2017;284: 1–9. doi:10.1098/rspb.2016.2361

56. Smith KT, Gauthier JA. Early Eocene lizards of the Wasatch Formation near Bitter Creek, Wyoming: Diversity and paleoenvironment during an interval of global warming. Bull Peabody Museum Nat Hist. 2013;54: 135–230. doi:10.3374/014.054.0205

57. Smith KT. A diverse new assemblage of Late Eocene squamates (Reptilia) from the Chadron Formation of North Dakota, U.S.A. Palaeontol Electron. 2006;9: 1–44.

58. Smith KT. A new lizard assemblage from the earliest Eocene (zone Wa0) of the Bighorn Basin, Wyoming, USA: Biogeography during the warmest interval of the Cenozoic. Journal of Systematic Palaeontology. 2009. doi:10.1017/S1477201909002752

59. Smith KT. The evolution of mid-latitude faunas during the Eocene: Late Eocene lizards of the Medicine Pole Hills reconsidered. Bull Peabody Museum Nat Hist. 2011;52: 3–105.

60. Smith KT. The long-term history of dispersal among lizards in the early Eocene: New evidence from a microvertebrate assemblage in the Bighorn Basin of Wyoming, USA. Palaeontology. 2011;54: 1243–1270. doi:10.1111/j.1475-4983.2011.01107.x

61. Gauthier JA, Kearney M, Maisano JA, Rieppel O, Behlke ADB. Assembling the Squamate Tree of Life : Perspectives from the Phenotype and the Fossil Record Assembling the Squamate Tree of Life : Perspectives from the Phenotype and the Fossil Record. Bull Peabody Museum Nat Hist. 2012;53: 3–308. doi:10.3374/014.053.0101

62. Wiens JJ, Hutter CR, Mulcahy DG, Noonan BP, Townsend TM, Sites JW, et al. Resolving the phylogeny of lizards and snakes (Squamata) with extensive sampling of genes and species. Biol Lett Lett. 2012;8: 1043–1046. doi:10.5061/dryad.g1gd8

63. Woodburne MO, editor. Late Cretaceous and Cenozoic Mammals of North America: Biostratigraphy and Geochronology. New York: Columbia University Press; 2004.

64. Barnosky AD, Holmes M, Kirchholtes R, Lindsey E, Maguire KC, Poust AW, et al. Prelude to the Anthropocene: Two new North American Land Mammal Ages (NALMAs). Anthr Rev. 2014;1: 225–242. doi:10.1177/2053019614547433

65. Estes R, De Queiroz K, Gauthier J. Phylogenetic Relationships within Squamata. In: Estes R, Pregill G, editors. Phylogenetic Relationships of the Lizard Families. Palo Alto, CA: Stanford University Press; 1988. pp. 119–281.

66. Frost DR, Etheridge R. A phylogenetic analysis and taxonomy of iguanian lizards. Univ Kansas Museum Nat Hist Misc Publ. 1989;81: 1–65.

67. Smith RJ. Estimation of body mass in paleontology. J Hum Evol. 2002;43: 271–287. doi:10.1006/jhev.2002.0573

68. Hammer Ø, Harper DAT, Ryan PD. PAST: Palaeontological Statistics software package for education and data analysis. Palaeontol Electron. 2001;4: 1–9.

69. Gingerich PD. Arithmetic or geometric normality of biological variation: An empirical test of theory. J Theor Biol. 2000;204: 201–221. doi:10.1006/jtbi.2000.2008

70. Evans SE. The Skull of Lizards and Tuatara. Volume 20,. In: Gans C, Gaunt AS, Adler K, editors. The Skull of Lepidosauria. Volume 20,. Ithaca, NY: Society for the Study of Amphibians and Reptiles; 2008. pp. 1–348.

71. ElShafie SJ. Body size and species richness changes in Glyptosaurinae (Squamata:

Anguidae) through climatic transitions of the North American Cenozoic. Master's Thesis. Department of Earth & Atmospheric Sciences: University of Nebraska, Lincoln. University of Nebraska, Lincoln. 2014.

72. Meszoely CAM. North American fossil anguid lizards. Bull Museum Comp Zool. 1970;139: 87–150.

73. Gilmore CW. Fossil Lizards of North America. Mem Natl Acad Sci. 1928;22: v–201.

74. Sullivan RM. The taxonomy, chronostratigraphy and paleobiogeography of glyptosaurine lizards (Glyptosaurinae, Anguidae). Comptes Rendus Palevol. 2019;18: 747–763. doi:10.1016/j.crpv.2019.05.006

75. Scarpetta SG. *Peltosaurus granulosus* (Squamata, Anguidae) from the middle Oligocene of Sharps Corner, South Dakota, and the youngest known chronostratigraphic occurrence of Glyptosaurinae. J Vertebr Paleontol. 2019;39: e1622129. doi:10.1080/02724634.2019.1622129

76. Pianka ER. Evolution of body size: Varanid lizards as a model system. Am Nat. 1995;146: 398–414.

77. Makarieva AM, Gorshkov VG, Li BL. Gigantism, temperature and metabolic rate in terrestrial poikilotherms. Proc R Soc B Biol Sci. 2005;272: 2325–2328. doi:10.1098/rspb.2005.3223

78. Makarieva AM, Gorshkov VG, Li BL. Temperature-associated upper limits to body size in terrestrial poikilotherms. Oikos. 2005;111: 425–436. doi:10.1111/j.1600-0706.2005.14095.x

79. Pough FH. Lizard Energetics and Diet. Ecology. 1973;54: 837–844. doi:10.2307/1935678

80. Wing SL, Harrington GJ, Smith FA, Bloch JI, Boyer DM, Freeman KH. Transient floral

change and rapid global warming at the Paleocene-Eocene boundary. Science (80-). 2005;310: 993–996. doi:10.1126/science.1116913

81. Aureliano T, Ghilardi AM, Guilherme E, Souza-Filho JP, Cavalcanti M, Riff D. Morphometry, bite-force, and paleobiology of the Late Miocene caiman *Purussaurus brasiliensis*. PLoS One. 2015;10: 1–14. doi:10.1371/journal.pone.0117944

82. Molnar RE. Dragons in the dust: the paleobiology of the giant monitor lizard *Megalania*. Indiana University Press; 2004.

83. Conrad JL, Balcarcel AM, Mehling CM. Earliest Example of a Giant Monitor Lizard (*Varanus*, Varanidae, Squamata). PLoS One. 2012;7: e41767. doi:10.1371/journal.pone.0041767

84. Sinervo B, Méndez-de-la-Cruz F, Miles DB, Heulin B, Bastiaans E, Cruz MVS, et al. Erosion of lizard diversity by climate change and altered thermal niches. Science (80-). 2010;328: 894–899. doi:10.1126/science.1184695

85. Gregory KM, McIntosh WC. Paleoclimate and paleoelevation of the Oligocene Pitch-Pinnacle flora, Sawatch Range, Colorado. Geol Soc Am Bull. 1996;108: 545. doi:10.1130/0016-7606(1996)108<0545:PAPOTO>2.3.CO;2

86. Snell KE. Paleoclimate and paleoelevation of the western Cordillera in the United States. University of California, Santa Cruz. 2011.

87. Snell KE, Thrasher BL, Eiler JM, Koch PL, Sloan LC, Tabor NJ. Hot summers in the Bighorn Basin during the early Paleogene. Geology. 2013;41: 55–58. doi:10.1130/G33567.1

88. Huey RB, Slatkin M. Cost and benefits of lizard thermoregulation. Q Rev Biol. 1976;51: 363–384.

89. Wing SL. Eocene and Oligocene floras and vegetation of the Rocky Mountains. Ann Missouri Bot Gard. 1987;74: 748. doi:10.2307/2399449

90. Wing SL, Bown TM, Obradovich JD. Early Eocene biotic and climatic change in interior western North America. Geology. 1991;19: 1189–1192. doi:10.1130/0091-7613(1991)019<1189:EEBACC>2.3.CO;2

91. Stucky RK. Mammalian faunas in North America of Bridgerian to Early Arikareean “Ages” (Eocene and Oligocene). In: Prothero DR, Berggren WA, editors. Eocene-Oligocene Climatic and Biotic Evolution. Princeton, NJ: Princeton University Press; 1992. pp. 464–493.

92. Stamps JA. Rainfall, Moisture and Dry Season Growth Rates in *Anolis aeneus*. Copeia. 1977;1977: 415–419.

93. Wing SL, Greenwood DR. Fossils and fossil climate: The case for equable continental interiors in the Eocene. Philos Trans - R Soc London, B. 1993;341: 243–252. doi:10.1098/rstb.1993.0109

94. Fricke HC, Wing SL. Oxygen isotope and paleobotanical estimates of temperature and d18O-latitude gradients over North America during the early Eocene. Am J Sci. 2004;304: 612–635.

95. Huey RB. Behavioral thermoregulation in lizards: Importance of associated costs. Science (80-). 1974;184: 1001–1003.

96. Lafuma F, Corfe IJ, Clavel J, Di-Poï N. Multiple evolutionary origins and losses of tooth complexity in squamates. Nat Commun. 2021;12: 1–13. doi:10.1038/s41467-021-26285-w

97. Angilletta MJ, Steury TD, Sears MW. Temperature, growth rate, and body size in

ectotherms: Fitting pieces of a life-history puzzle. Integr Comp Biol. 2004;44: 498–509.

98. Kingsolver JG, Huey RB. Size, temperature, and fitness: Three rules. Evol Ecol Res. 2008;10: 251–268.

99. Beck DD. Biology of Gila Monsters and Beaded Lizards. 9th ed. Berkeley: University of California Press; 2005.

100. Angilletta, Jr. MJ, Niewiarowski PH, Dunham AE, Leaché AD, Porter WP. Bergmann's Clines in Ectotherms: Illustrating a Life- History Perspective with Sceloporine Lizards. Am Nat. 2004;164: E168–E183. doi:10.1086/425222

101. Woolley CH, Thompson JR, Wu Y-H (Beckey), Bottjer DJ, Smith ND. Can a fragmented past be trusted? Assessing bias and phylogenetic signal in the squamate fossil record. J Vertebr Paleontol Progr Abstr. 2020; 351–352.

102. Woolley CH, Thompson JR, Wu YH, Bottjer DJ, Smith ND. A biased fossil record can preserve reliable phylogenetic signal. Paleobiology. 2022; 1–16. doi:10.1017/pab.2021.45

103. Bell CJ, Gauthier JA, Bever GS. Covert biases, circularity, and apomorphies: A critical look at the North American Quaternary Herpetofaunal Stability Hypothesis. Quat Int. 2010;217: 30–36. doi:10.1016/j.quaint.2009.08.009

104. Ledesma DT, Scarpetta SG, Bell CJ. Variation in the skulls of *Elgaria* and *Gerrhonotus* (Anguidae, Gerrhonotinae) and implications for phylogenetics and fossil identification. PeerJ. 2021;9: 1–80. doi:10.7717/peerj.11602

105. Scarpetta SG, Ledesma DT, Bell CJ. A new extinct species of alligator lizard (Squamata: *Elgaria*) and an expanded perspective on the osteology and phylogeny of Gerrhonotinae. BMC Ecol Evol. 2021;21: 1–58. doi:10.1186/s12862-021-01912-8

106. The Paleobiology Database. Available: www.paleobiodb.org

107. Estes R. The fossil record and early distribution of lizards. Adv Herpetol Evol Biol. 1983; 365–398.

108. Nydam RL, Fitzpatrick BM. The occurrence of contogenys-like lizards in the late cretaceous and early tertiary of the western interior of the U.S.A. J Vertebr Paleontol. 2009;29: 677–701. doi:10.1671/039.029.0331

109. Estes R. A scincoid lizard from the Cretaceous and Paleocene of Montana. Cambridge, MA; 1969.

110. Bryant LJ. Non-dinosaurian lower vertebrates across the Cretaceous-Tertiary boundary in northeastern Montana. Vol. 134. Berkeley: University of California Press; 1989.

111. Conrad JL. An Eocene shinisaurid (Reptilia, Squamata) from Wyoming, U.S.A. J Vertebr Paleontol. 2006;26: 113–126. doi:10.1671/0272-4634(2006)26[113:AESRSF]2.0.CO;2

112. Tschopp E, Napoli JG, Wencker LCM, Delfino M, Upchurch P. How to Render Species Comparable Taxonomic Units Through Deep Time: A Case Study on Intraspecific Osteological Variability in Extant and Extinct Lacertid Lizards. Syst Biol. 2022;71: 875–900. doi:10.1093/sysbio/syab078

Supporting information

Materials & methods

Data collection

Almost all the 283 fossil lizard specimens listed in S1 Dataset were measured in person in museum collections. A few measurements were obtained from literature or specimen photos provided by researchers (see “Measurements” below). The fossil dataset was likely biased toward adult individuals because complete anatomical elements are more likely to be preserved from adults. Juveniles are less likely to get preserved as fossils because their bones are more delicate and juveniles are frequently consumed by predators [100]. This preservation bias would not adversely affect the results in this study since it focuses on maximum body sizes.

The 266 extant specimens measured in this study (S2 Dataset) include 86 extant species total and 1-19 individuals sampled per species. This dataset only includes extant species for which reasonably complete dry skeletonized specimens were available. Legless anguids and scincids were omitted because proportions of cranial elements to SVL differ between limbed taxa and serpentiform taxa in extant squamate groups [21] and because all the extinct taxa in the fossil dataset presented here are inferred to have had limbs based on associated limb material or assignment to extant groups with limbs. These data for this study were amassed from 26 different natural history museum collections across the United States (see “Institutional Abbreviations” in the main text).

When collecting extant data, the target sample size was at least n = 20 for each taxonomic group. The sample size was only smaller for Xenosauridae (n = 12; S3 Table), so results for this group should be considered with caution.

Most described diagnostic characters for lizards are found in cranial bones [101,102]. Cranial bones account for most of the curated and published fossil record of lizards. This record is biased toward dentaries and maxillae, which are easy to identify as belonging to a lizard based on dentition and often provide at least coarse taxonomic identifications (family or higher) [102]. Therefore, we can be reasonably confident in taxonomic assignments for fossil lizard cranial bones based on extant phylogenetic morphological characters (although caution should be taken with historical identifications that may lack fidelity, especially at the genus or species level [103–105]).

Fossil specimens were referred to crown groups (family-level or higher) based on specimen label identifications as well as taxonomic information from the Paleobiology Database [106] and current literature. For extinct taxa whose exact placement within or around an extant lizard group remains unresolved, the taxon was assigned to the closest crown group for the purpose of this study. This was the case for three extinct taxa: *Palaeoscincosaurus* and *Contogenys*, both referred to Scincidae [107–110], and *Provaranosaurus*, referred to Shinisauridae. The latter was based on Smith and Gauthier [56], who asserted that the extinct genus *Provaranosaurus* is closely related to extant *Shinisaurus* and placed it within a group called "*Pan-Shinisaurus*." Any such considerations for specific taxa are indicated in the "Notes" column in S1 Dataset.

Measurements

All measurements were taken with digital calipers (Mitutoyo 150 mm) to the nearest 0.1 mm for individual bones and the nearest 1 mm for SVL. For specimens with an SVL measuring > 150 mm, or for specimens that were preserved in a curved position, a tape measure was used to measure SVL to the nearest 1 mm. Only extant specimens for which SVL could be measured

were included, i.e., dry skeletonized specimens with a complete articulated vertebral series. Two xenosaurid specimens with wet-preserved bodies and dry skeletonized skulls (S2 Dataset) were also included because fewer xenosaurid specimens were available and these two specimens still provided the measurements needed. SVL was measured on skeletons as the length from the tip of the snout to the posterior centrum of the second caudal vertebra, which is the approximate position of the middle of the cloaca (vent; see Fig 1). The position of the cloaca on skeletonized specimens was determined by aligning the pelvis of a wet specimen with that of a dry skeletonized congener specimen of comparable size. Whenever a dried skin was included with a skeletonized specimen, it was used to verify the SVL measurement from the vertebral series.

Skull length was measured as the length from the tip of the snout to the most posterior point of the skull (either the occipital condyle or the posterior tip of the supratemporal process). If a cranial bone had bilateral asymmetry (e.g., if on a parietal, one supratemporal process was longer than the other), the longer side was taken to represent the maximum measurement. Each cranial bone was measured from the most anterior to the most posterior point (e.g., on the dentary, from the anterior tip of the ramus to the posterior tip of the retroarticular process). For long bones, only complete bones with epiphyses included were measured.

When it was not possible to access a fossil specimen in person (which was the case for the UMMP specimens and a few specimens measured from figures in literature), measurements were taken from digital photographs with a scale bar in standard orientation using the open access software ImageJ (available online at: https://imagej.nih.gov/ij/download.html). Measurements taken from photographs are indicated in the "Notes" column in S1 Dataset. Measurements taken both in person and from photographs were compared for several extant

specimens to ensure that the measurements taken from photographs were accurate: all measurements proved to be within a margin of error of 5% or less (1-3 mm).

Generating and testing regressions

Regressions were only generated for the anatomical elements represented in the fossil dataset for each specific lizard group (S1-S7 Tables). For example, no isolated fossil varanid femora were measured, so a regression for varanid femur length to SVL was not included (S2 Table).

Each regression was tested for homoskedasticity and for normal distribution of residuals (p(homosked) and p(norm) ≥ 0.05) to validate the assumptions of the regression. These tests were performed using PAST v.4.03 [68].

For each extant lizard group, one specimen with a head length and SVL close to the average in the extant dataset was selected for that group and remove from the dataset prior to calculating the regressions. That removed specimen was then used to test the accuracy of each regression generated from the remaining sample of that extant lizard group (S1-S7 Tables). The samples of extant specimens encompassed the range of body size for most of the crown groups sampled except for Anguidae and Varanidae. The largest taxa in those families were exceptionally large outliers (*Diploglossus millepunctatus* (Anguidae); *Varanus komodoensis* (Varanidae)). Those outliers were therefore omitted from the samples used to generate regressions. However, specimens of those outliers were applied to their respective family regressions as an additional test. The resulting SVL differences were comparable (see Varanidae, S2 Table) or lower (see Anguidae, S1 Table) than the first test specimen, indicating that those regressions can reliably predict SVL for large outliers in those families.

A large specimen of *Heloderma horridum* (Helodermatidae), the skull of which greatly resembles those of the largest anguid fossils in this dataset (Fig 3E), was used as an additional test specimen for the anguid regressions (S1 Table). Helodermatidae is placed close to Anguidae within the group Anguimorpha in both morphological and molecular phylogenetic analyses [61,62,65,66], so it is reasonable to consider Helodermatidae as an extant morphological and close phylogenetic analogue to large extinct anguid lizards. The anguid regressions proved comparably accurate in predicting the SVL of the largest helodermatid test specimen compared to the smaller extant and extinct anguid test specimens.

If a regression for a needed element failed the test for homoskedasticity or normal distribution of residuals, outliers were removed from the dataset for that extant lizard group [67]. The outliers were no larger or smaller than any other taxa included, but their anatomical proportions differed from their congeners. SVL was then estimated for the test specimens with and without the outliers removed. If the predicted values for SVL barely changed, regressions were generated for that group with the outliers removed. This was the case for groups such as Anguidae and Iguania. However, if the new SVL estimate was very different, or if removing clear outliers still did not result in either regression passing the tests for both homoskedasticity and normal distribution of residuals, then the regression was removed completely and fossil specimens that would have relied on that regression were omitted from the study. This resulted in the removal of a handful of fossil specimens known from a single element for which a suitable regression could not be generated. For example, a few fossil varanid specimens known only from a frontal or parietal were omitted.

Minimum and maximum estimates of SVL (95% confidence interval) were calculated for each specimen by subtracting or adding the standard error, respectively, for the slope and interval of the regression used (S1 Dataset, S1-S7 Tables).

If a particular fossil specimen included more than one complete element, the regression that offered the lowest standard error of the estimate (SEE) was used. The regression equations in Table 3 and S1-S7 Tables are ranked in this order. For example, one fossil varanid specimen, FMNH PR 2380, included a complete dentary, maxilla, frontal, and parietal (S1 Dataset). The extant varanid dataset did not yield viable regressions for SVL based on frontal or parietal length, neither of those elements could be used to estimate SVL for that specimen. The varanid regression for maxilla length to SVL yielded a lower SEE than that for dentary length to SVL (S2 Table), so the maxilla length regression was used to estimate SVL for that specimen (S1 Dataset).

Separate regressions for males and females were not generated because most extant specimens measured did not have a sex indicated. Although female lizards often have shorter SVLs than males [4], there is no reliable way to tell the sex of an individual fossil lizard specimen based on isolated bones, so any fossil lizard dataset must be treated as an averaged sample across sexes. The same is largely true for ontogenetic stages represented in a fossil dataset. Adult members of an extinct taxon can be reasonably identified if a large sample of referred individuals that reach a consistent maximum size range is present (e.g., large fossil anguids, S1 Dataset) or if some of those specimens include long bones with fused epiphyses, but some subadult or juvenile specimens may also be present.

An attempt was made to combine measurements from different lizard groups to create "generic" lizard regressions to generate SVL estimates for unidentified lizard elements.

However, none of the resulting regressions passed the necessary tests (see the failed generic equations in S9 Table). Anatomical proportions of the extant data sampled here varied too much between crown groups to produce successful generic regressions.

Special considerations for Iguania

Within extant Iguania, a representative range of the body size, morphology, and taxonomic diversity within pleurodontans was sampled because all the fossil iguanian specimens measured had pleurodont dentition but not all were identified to a specific pleurodontan family. *Amblyrhynchus* and *Crotaphytus* were not included because they were outliers with respect to head length : SVL ratio compared to the rest of the extant iguanian dataset (0.14 for *Amblyrhynchus* and 0.26 for *Crotaphytus* compared to an average of 0.21 for the other genera).

Special considerations for Shinisauridae

No complete dry skeletonized specimens of extant *Shinisaurus* were available to measure. Only one dry skull and four wet preserved specimens of this monotypic family were found. However, the holotype and only known complete skeleton of an extinct taxon referred to Shinisauridae, *Bahndwivici ammoskius* (FMNH PR 2260, Fig 5A) [111] was available. The *B. ammoskius* holotype skeleton had the same head length : SVL ratio as the average calculated for extant *Shinisaurus* based on wet preserved specimens (0.23 vs. 0.22, n = 4). Therefore, ratios of individual cranial elements to SVL from the *Bahndwivici* skeleton were used to estimate SVL for the handful of fossil specimens referred to Shinisauridae (S1 Dataset).

Estimating body mass

The equation provided by Feldman et al. **[42]** was used for Shinisauridae because an equation for Shinisauridae was not provided and Shinisauridae falls within Anguimorpha. The equation for Fully Legged Anguidae was used from the

same reference for the fossil anguid lizards because limb material has been found with many fossil anguid specimens and none have been interpreted as limbless.

Discussion

Taxonomic richness

No more than two distinct species were documented for any given genus in the fossil dataset (S1 Dataset). Many fossil specimens could not be identified with certainty past the level of genus (e.g., *Melanosaurus sp*.). There is no way to know exactly how many different species might be represented by these specimens, even if only one diagnostic species is recognized within a given extinct genus (e.g., *Melanosaurus maximus*). Many of the extinct genera in this study that included only one diagnostic species almost certainly included other unidentified species as well. For example, the extinct anguid genus *Melanosaurus* had greater variation in SVL than any individual extant anguid species (standard deviation = 222 vs. 34 (*Gerrhonotus liocephalus*); S1 and S2 Datasets). In general, it is much more difficult to discern diagnostic species within a genus than it is to parse genera within a clade based on fossils [112]. In this fossil dataset, 88% of specimens were identified to the level of genus but only 42% were referred to a species. This may account for some of the discrepancies between the data reported here and the data available in the Paleobiology Database (Table 2).

However, this is unlikely to affect the patterns of taxonomic richness observed here. Even in extant lizards, elevated richness usually reflects a high number of genera with a low number of species per genus, possibly because lizard genera within families are more ecologically distinct from each other than are species within genera [3]. Thus, in lieu of trying to estimate the actual number of extinct species sampled, tracking richness at the level of genera and higher taxonomic

groups (S3 Fig) is likely to capture a sufficient representation of the taxonomic diversity in this geotemporal system.

Supporting Captions

S1 Fig. Protocol for estimating snout-vent length of fossil lizards.

S2 Fig. Regressions of dentary length to snout-vent length for all extant lizard groups sampled. Shinisauridae is not included here because no regressions were generated for that group (see "Special Considerations for Shinisauridae" in Supporting Information). See Table 3 for regression equations and statistics. All regressions are transformed using natural log.

S3 Fig. Rarefaction curve showing the number of expected crown taxonomic groups for NALMA sample sizes within the fossil lizard dataset. S1 Dataset was subsampled here by North American Land Mammal Age (NALMA) interval. NALMA abbreviations: Pu = Puercan, To = Torrejonian, Ti = Tiffanian, Cf = Clarkforkian, Wa = Wasatchian, Br = Bridgerian, Ui = Uintan, Du = Duchesnean, Ch = Chadronian, Or = Orellan, Wh = Whitneyan, Ar = Arikareean. Individual rarefaction analysis was performed using PAST v4.03 [68].

S4 Fig. Rarefaction curve showing the number of expected size groups for NALMA sample sizes within the fossil lizard dataset. To conduct this analysis, each fossil specimen in S1 Dataset was assigned to one of 11 size groups, each group representing a bin of 100 mm (e.g., 1-99 mm, 100-199 mm, 200-299 mm, etc.; the last group was ≥ 1 m). S1 Dataset was subsampled here by North American Land Mammal Age (NALMA) interval. The y-axis in this graph shows

the total number of size groups recovered in the sample for each NALMA interval. NALMA abbreviations: Pu = Puercan, To = Torrejonian, Ti = Tiffanian, Cf = Clarkforkian, Wa = Wasatchian, Br = Bridgerian, Ui = Uintan, Du = Duchesnean, Ch = Chadronian, Or = Orellan, Wh = Whitneyan, Ar = Arikareean. Individual rarefaction analysis was performed using PAST v4.03 [68].

S5 Fig. Global temperature vs. lizard body size by NALMA. A) Global marine mean annual paleotemperature (MAPT) vs. maximum lizard SVL. Function: LN(Global Marine MAPT) = ((1.23±0.401)*LN(Max Lizard SVL)) + (3.70±0.792), R^2 = 0.04, p(uncorr.) = 0.56, SEE = 0.991. **B)** Global marine MAPT vs. mean lizard SVL. LN(Global Marine MAPT) = ((-0.910±0.303)*LN(Mean Lizard SVL)) + (7.06±0.606), R^2 = 0.0004, p(uncorr.) = 0.95, SEE = 0.812. Temperature data are from Zachos et al. [44] and were taken from the NALMA midpoint in ºC. All data were transformed using natural log. Regression analysis was performed using PAST v4.03 [68].

S6 Fig. Mean and maximum snout-vent length in fossil lizards by taxonomic group per NALMA. Dotted line = maximum. Dashed line = mean. Lines do not connect with the single Duchesnean datapoint since it likely does not accurately represent a maximum or mean value for that NALMA. North American Land Mammal Age (NALMA) abbreviations: Pu = Puercan, To = Torrejonian, Ti = Tiffanian, Cf = Clarkforkian, Wa = Wasatchian, Br = Bridgerian, Ui = Uintan, Du = Duchesnean, Ch = Chadronian, Or = Orellan, Wh = Whitneyan, Ar = Arikareean. All measurements were transformed using natural log.

S7 Fig. Mean and maximum body mass in fossil lizards by taxonomic group per NALMA. Dotted line = maximum. Dashed line = mean. Lines do not connect with the single Duchesnean datapoint since it likely does not accurately represent a maximum or mean value for that NALMA. North American Land Mammal Age (NALMA) abbreviations: Pu = Puercan, To = Torrejonian, Ti = Tiffanian, Cf = Clarkforkian, Wa = Wasatchian, Br = Bridgerian, Ui = Uintan, Du = Duchesnean, Ch = Chadronian, Or = Orellan, Wh = Whitneyan, Ar = Arikareean. All measurements were transformed using natural log.

S1 Table. Anguidae regression equations and tests. Sample sizes indicate number of extant anguid specimens that included the element used in the given regression. Regression equations are ranked in order of increasing standard error of the estimate (SEE). Minimum and maximum SVL estimates for test specimens are based on the standard errors for the slope and intercept of the given regression. Equations for body mass from individual bones are based on the relevant equation listed in S8 Table.

S2 Table. Varanidae regression equations and tests. Sample sizes indicate number of extant varanid specimens that included the element used in the given regression. Regression equations are ranked in order of increasing standard error of the estimate (SEE). Minimum and maximum SVL estimates for test specimens are based on the standard errors for the slope and intercept of the given regression. Equations for body mass from individual bones are based on the relevant equation listed in S8 Table.

S3 Table. Xenosauridae regression equations and tests. Sample sizes indicate number of extant xenosaurid specimens that included the element used in the given regression. Regression equations are ranked in order of increasing standard error of the estimate (SEE). Minimum and maximum SVL estimates for test specimens are based on the standard errors for the slope and intercept of the given regression. Equations for body mass from individual bones are based on the relevant equation listed in S8 Table.

S4 Table. Teiidae regression equations and tests. Sample sizes indicate number of extant teiid specimens that included the element used in the given regression. Regression equations are ranked in order of increasing standard error of the estimate (SEE). Minimum and maximum SVL estimates for test specimens are based on the standard errors for the slope and intercept of the given regression. Equations for body mass from individual bones are based on the relevant equation listed in S8 Table.

S5 Table. Scincidae regression equations and tests. Sample sizes indicate number of extant scincid specimens that included the element used in the given regression. Regression equations are ranked in order of increasing standard error of the estimate (SEE). Minimum and maximum SVL estimates for test specimens are based on the standard errors for the slope and intercept of the given regression. Equations for body mass from individual bones are based on the relevant equation listed in S8 Table.

S6 Table. Xantusiidae regression equations and tests. Sample sizes indicate number of extant xantusiid specimens that included the element used in the given regression. Regression equations

are ranked in order of increasing standard error of the estimate (SEE). Minimum and maximum SVL estimates for test specimens are based on the standard errors for the slope and intercept of the given regression. Equations for body mass from individual bones are based on the relevant equation listed in S8 Table.

S7 Table. Iguania regression equations and tests. Sample sizes indicate number of extant iguanian specimens that included the element used in the given regression. Regression equations are ranked in order of increasing standard error of the estimate (SEE). Minimum and maximum SVL estimates for test specimens are based on the standard errors for the slope and intercept of the given regression. Equations for body mass from individual bones are based on the relevant equation listed in S8 Table.

S8 Table. Published equations for estimating lizard body mass from snout-vent length. These equations were used to estimate mass for fossil lizards as listed in S1 Dataset and to generate equations for estimating body mass from individual bones (S1-S7 Tables).

S9 Table. Tests of general regression equations. Sample sizes indicate number of extant specimens that included the element used in the given regression. Regression equations are ranked in order of increasing standard error of the estimate (SEE). None of these equations passed tests for both homoskedasticity and normal distribution of residuals, thus, none of these equations were used in this study.

S10 Table. Data for correlating SVL with temperature. Temperature data came from Zachos et al. [44] and were taken from the NALMA midpoint in ºC. The Duchesnean was not included because the maximum SVL estimate for that NALMA was exceptionally low and based on a single data point.

S11 Table. Mean and maximum fossil lizard snout-vent length and body mass by taxonomic group per NALMA. These natural log-transformed data were used to generate the graphs in S6 Fig and S7 Fig.

S1 Dataset. Fossil lizard data. Measurements, locality information, and body size estimates for all fossil lizard specimens sampled. Family-specific mass was calculated from equations listed in S8 Table [24,41,42]. General mass estimate (across all lizard groups) was calculated using the embedded equation from Meiri [24].

S2 Dataset. Extant lizard data. Data for all extant lizard specimens measured. Measurements taken from photographs using ImageJ are indicated in bold font.

Figure 1

Click here to access/download;Figure;Fig_1.tif

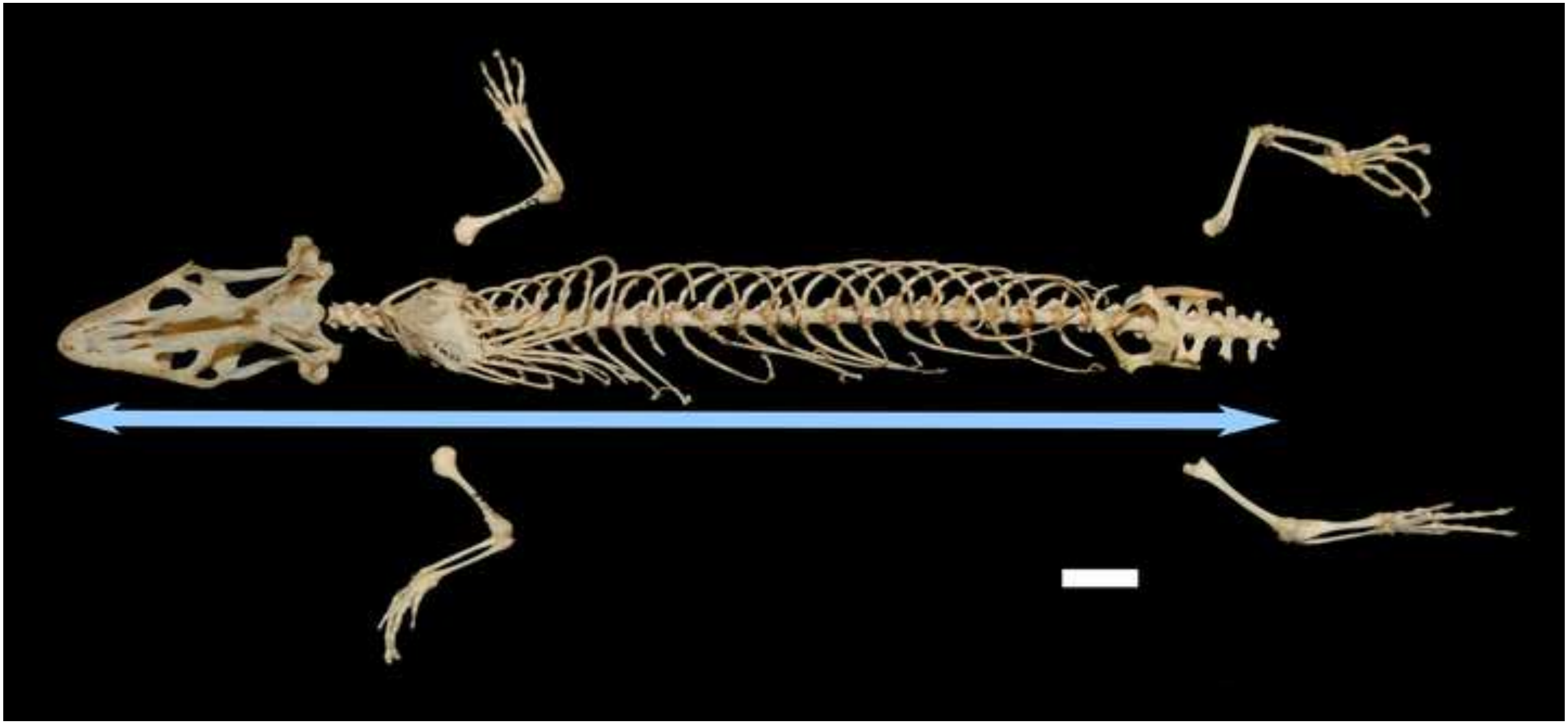

Figure 2

Click here to access/download;Figure;Fig_2.tif

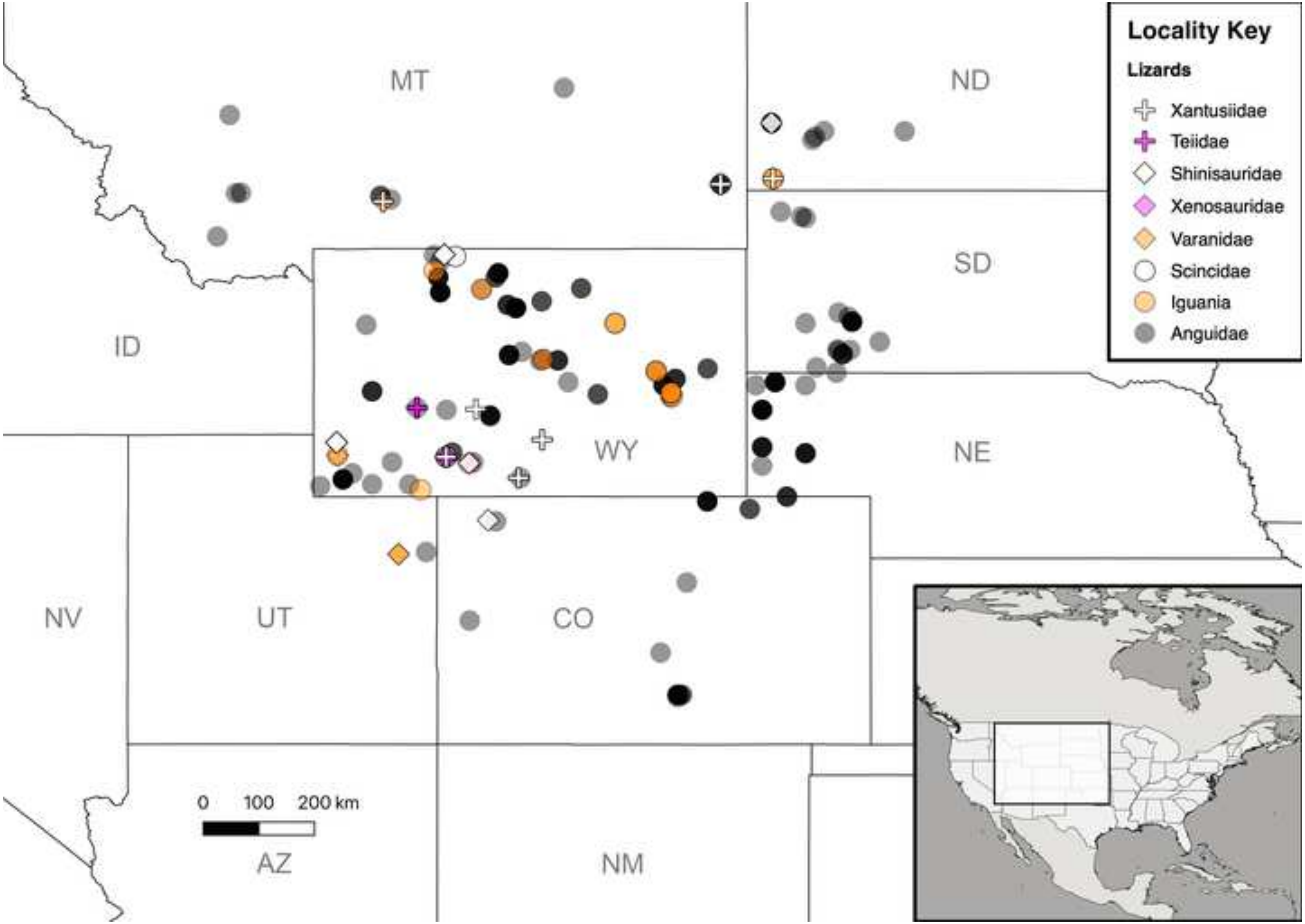

Figure 3

Click here to access/download;Figure;Fig_3.tif

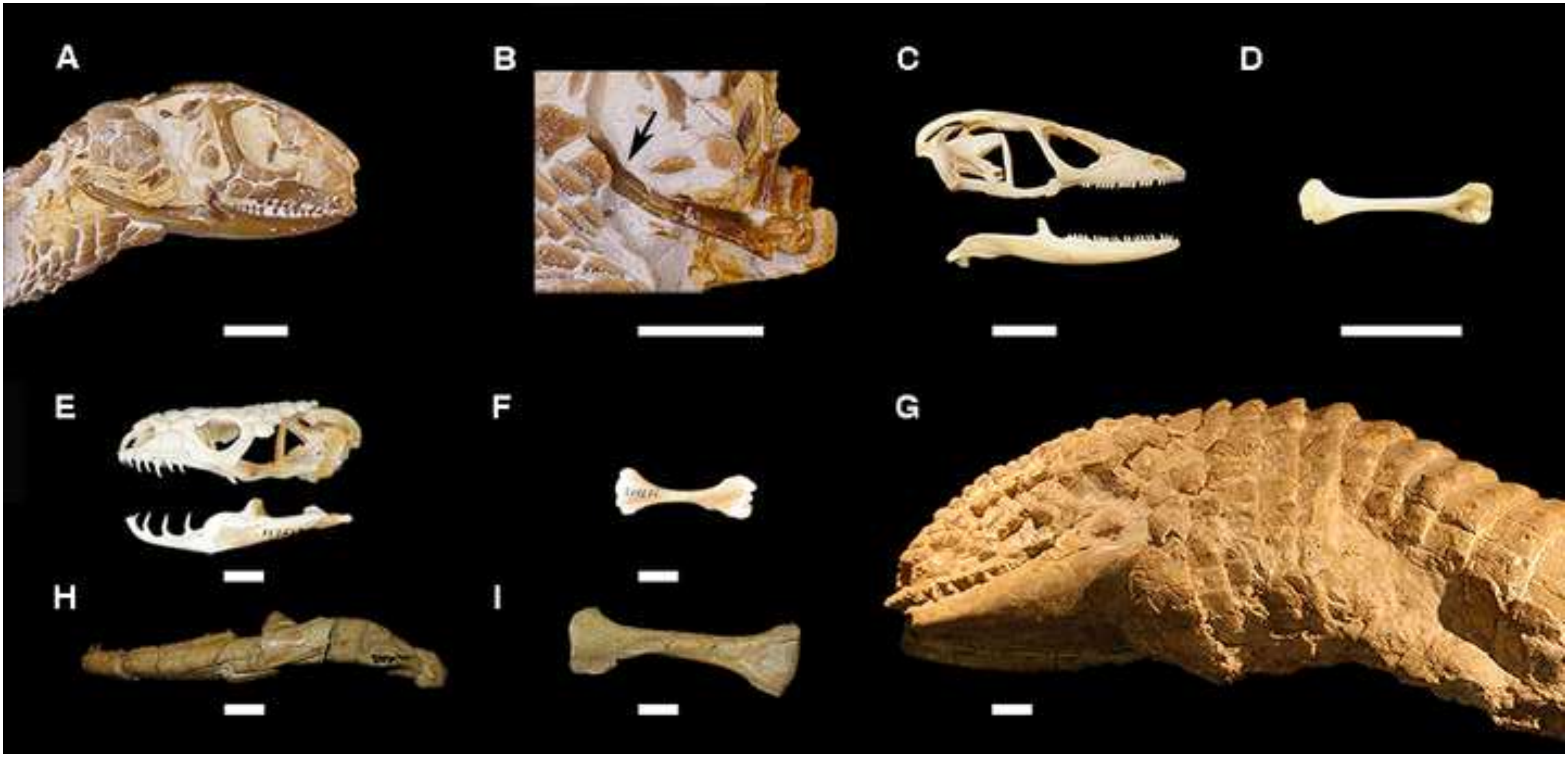

Click here to access/download;Figure;Fig_4.tif

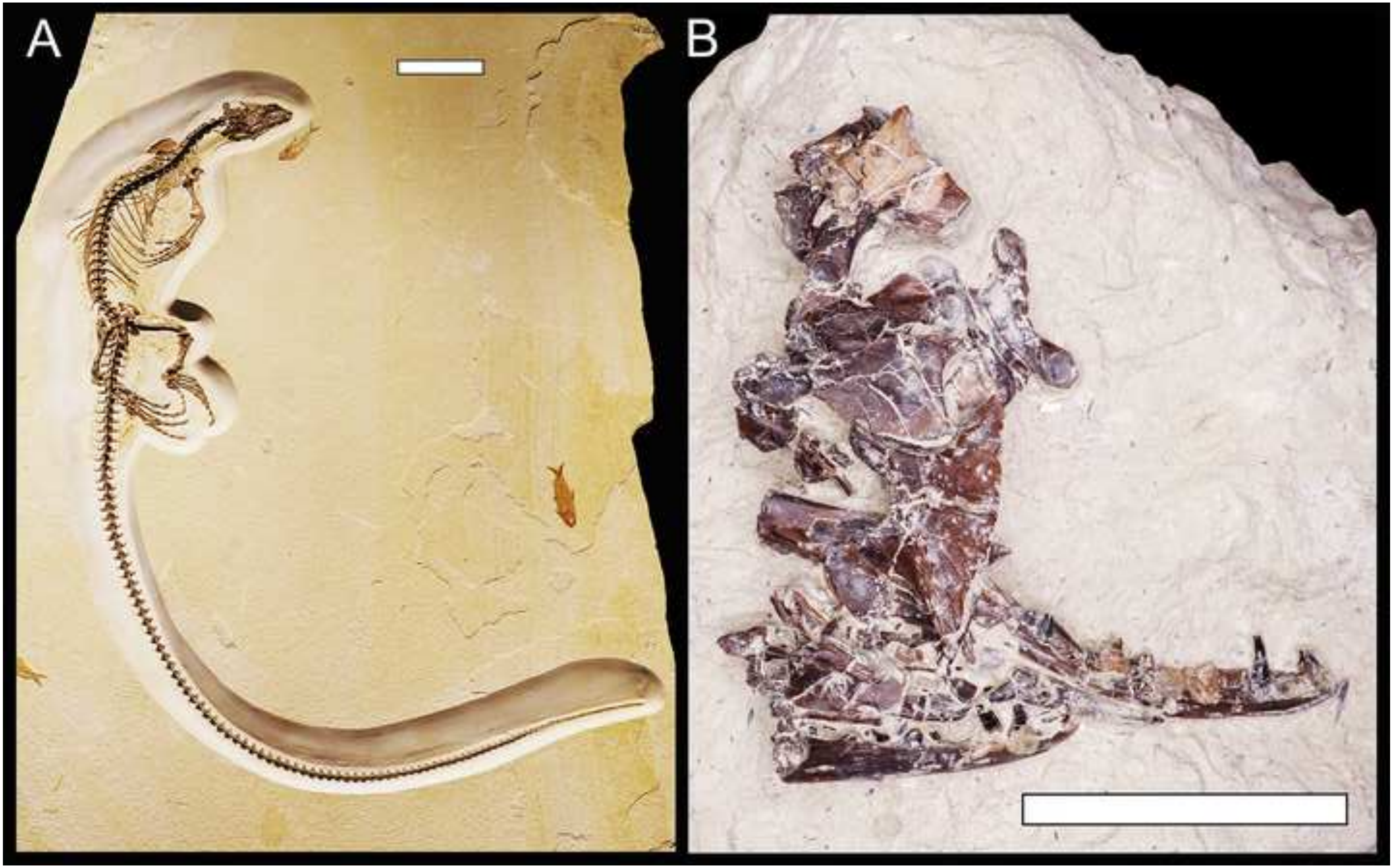

Figure 5

Click here to access/download;Figure;Fig_5.tif

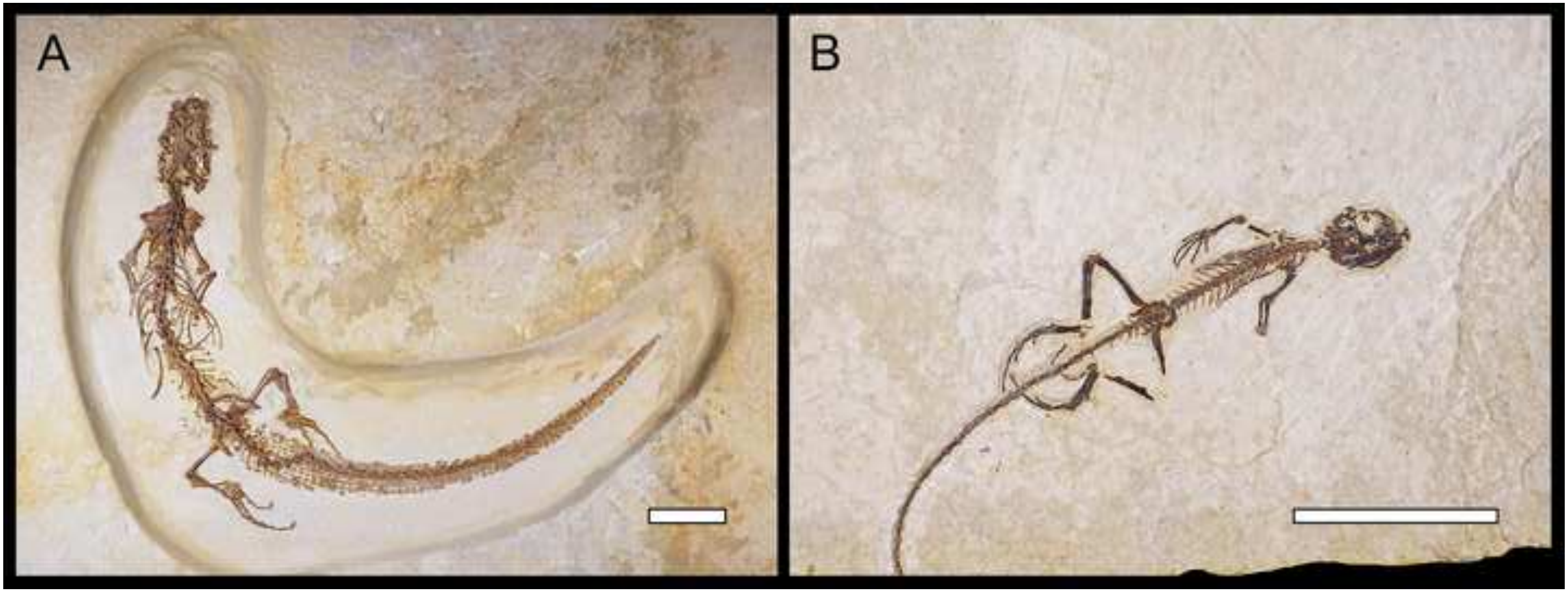

Figure 6

Click here to access/download;Figure;Fig_6.tif

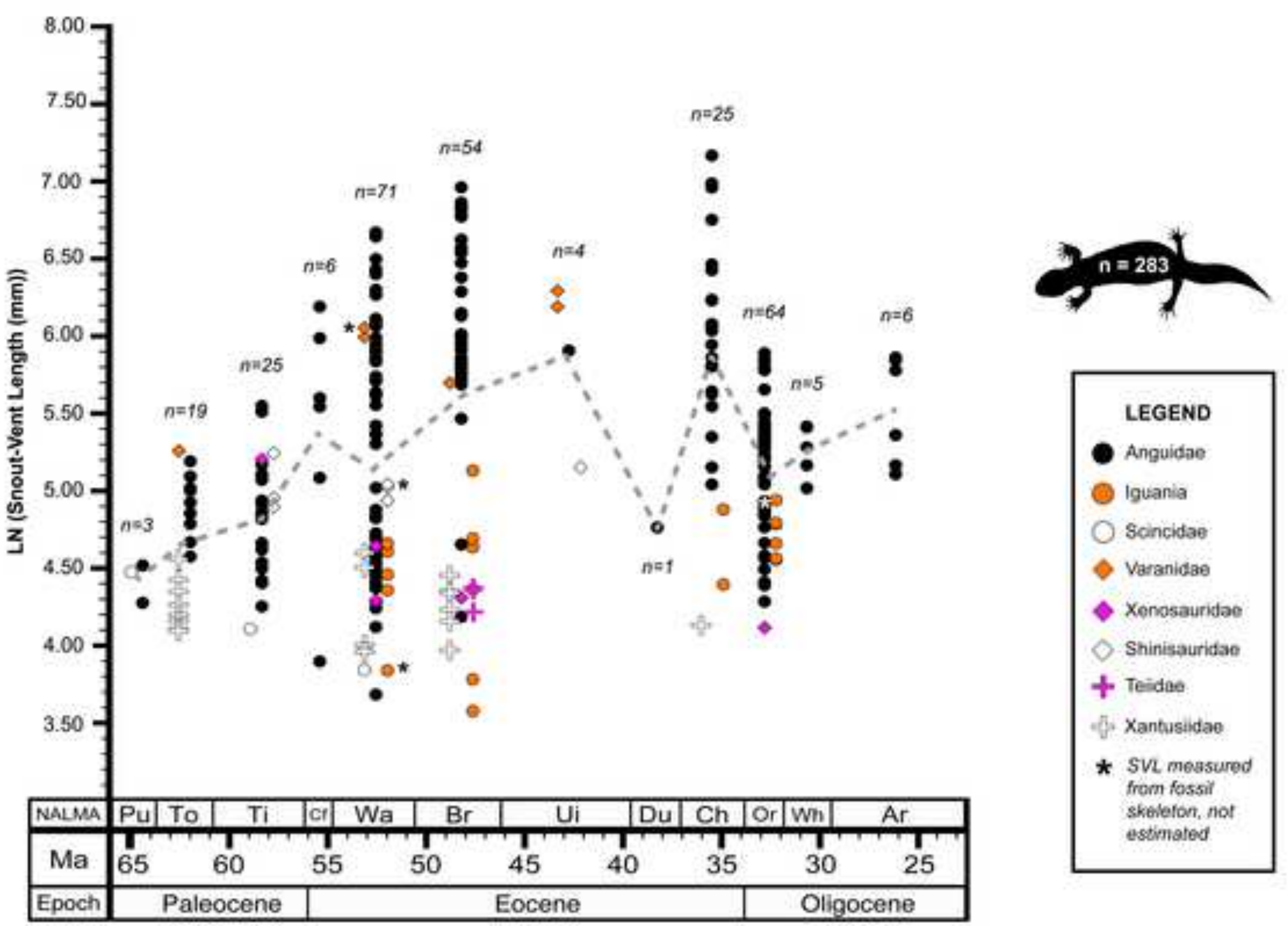

Figure 7

Click here to access/download;Figure;Fig_7.tif

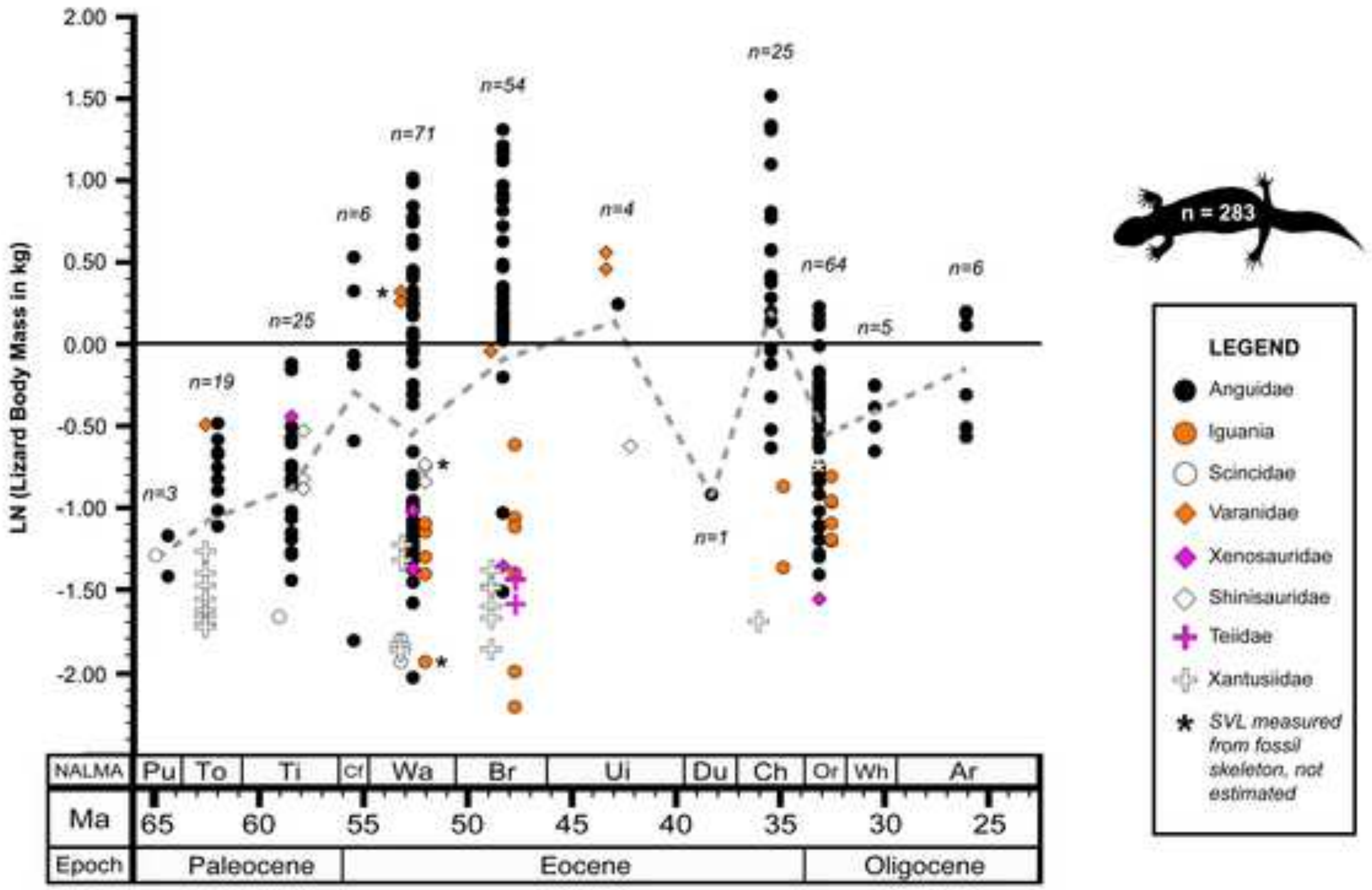

S1 Figure

Click here to access/download
Supporting Information
S1_Fig.tif

S2 Figure

Click here to access/download
Supporting Information
S2_Fig.tif

S3 Figure

Click here to access/download
Supporting Information
S3_Fig.tif

S4 Figure

Click here to access/download
Supporting Information
S4_Fig.tif

S5 Figure

Click here to access/download
Supporting Information
S5_Fig.tif

S6 Figure

Click here to access/download
Supporting Information
S6_Fig.tif

S1-S2 Datasets

Click here to access/download
Supporting Information
S1-S2_Datasets.xlsx

Click here to access/download
Supporting Information
S1-S11_Tables.xlsx

Body size estimation from isolated fossil bones reveals deep time evolutionary trends for lizards

Sara J. ElShafie

Department of Integrative Biology and Museum of Paleontology, University of California, Berkeley, California, United States of America

E-mail: selshafie@berkeley.edu

Abstract

Lizards play vital roles in extant ecosystems. However, their roles in extinct ecosystems are poorly understood because the fossil record of lizards consists mostly of isolated bones. This makes it difficult to document changes in lizard morphology and body size over time, which is essential for studies of lizard paleoecology and evolution. It is also difficult to compare available fossil lizard data with existing extant lizard datasets because extant studies rarely measure individual bones. Furthermore, no previous study has regressed measurements of individual bones to body length across crown lizard groups, nor tested those regressions on fossil skeletons. ~~Here I provide~~A ~~a~~n extensive dataset of extant lizard measurements across crown taxonomic groups is provided here, including individual bones, ~~across crown taxonomic groups, and derive~~from which methods for estimating lizard body size from isolated fossil elements were derived. ~~I apply~~T ~~t~~hese methods were applied to a comparably large dataset of fossil lizard specimens from the robust Paleogene record (66-23 Mya) of the Western Interior of North America. This study~~I~~ tests the hypothesis that anatomical proportions have been conserved within higher-level crown lizard groups since the Paleogene and can therefore be used to reconstruct snout-vent length (SVL) for fossil specimens referred to the same groups.

I ~~regressed i~~ndividual bones were regressed to SVL in each extant lizard group and ~~tested~~ the regressions were tested using measurements from complete extant specimens as well as ~~a handful of~~some rare fossil skeletons. The regressions predicted actual SVL for both the extant and fossil test specimens with ~~comparable~~ similar accuracy. The resulting body size estimates for the entire fossil dataset revealed that lizards reached greatest maximum body size for this study period in the middle Paleogene, with the largest size class dominated by ~~heavily armored~~ anguid lizards that exceeded 1 meter in SVL and 1 kg in body mass. Maximum body size decreased to

under 400 mm in the late Paleogene. This is the first study to investigate body size evolution across lizard clades over a deep time interval and ~~across~~ for a large geographic region. The proposed methods can provide ~~reasonable~~ estimates of SVL for fossil lizards referred to any crown group.

Introduction

Body size influences every aspect of a vertebrate’s physiology and life history [1–4]. It is therefore essential to estimate body size for fossil vertebrate taxa in order to understand the ecological and evolutionary context of any vertebrate group [5–10]. Lizards are a particularly important group of vertebrates to ~~understand~~ investigate in both extant and extinct ecosystems because they exhibit high taxonomic and morphological diversity and occupy myriad ecological roles [4,11–14]. For example, lizards make up the majority of tetrapods in some harsh environments, especially deserts;~~lizards occupy a wide range of habitats and environments including harsh conditions,~~ can maintain high population densities in small areas;~~,~~ and have high dispersal abilities [15–19]. Lizards also regulate populations of insects, rodents, and other prey on which they feed, and they are an important food source for many animals [4,12,13,20].

~~Unfortunately, complete fossil lizard skeletons are rare; most commonly found are~~The lizard fossil record consists mostly of isolated elements that can be difficult to identify to the level of genus or even family. Therefore, body size for fossil lizards must be inferred primarily from isolated bones [21]. To further complicate matters, anatomical datasets of extant lizards rarely include measurements of individual cranial or limb bones for comparison. Most relevant previous studies only recorded measurements of head length and SVL across extant lizard groups [3,4,11,12,19,22–26]. Hence, no prior study has reconstructed body size in lizards on

evolutionary time scales across higher-level taxonomic groups. This study~~I~~ addresses this need by presenting an extensive reference dataset of individual anatomical element measurements taken across crown group lizard lineages and detailing~~ed~~ methods for using those data to estimate body size in fossil lizards. ~~I use those~~These methods are used to reconstruct body size in lizard lineages over a deep time interval and across a continental interior.

~~The first question to consider here is which variable would be best to use as a measure of body size~~It is not unambiguously clear which variable is the best proxy for body size in fossil vertebrates. Some previous studies ~~of fossil crocodyliforms~~ estimated mass as a proxy for body size in fossil crocodyliforms using scaling relationships in extant taxa between body mass and ~~measurements~~ femoral length and circumference [27] and head width [28], since these anatomical measurements are commonly available in the crocodyliform fossil record~~of individual bones and body mass in extant taxa [27,28]~~. Body mass has also been estimated for dinosaurs [7] and fossil mammals [6,29,30] using similar methods.

However, ~~body mass can be difficult to estimate in this way for fossil vertebrates because~~ body mass is only weakly correlated with most individual bones in many fossil vertebrates; thus, such methods can be difficult to apply across a fossil record of varied isolated bones [6]. For fossil reptiles, b~~B~~ody length is ~~often more feasible~~easier ~~to estimate~~ to estimate from individual bones than body mass ~~for fossil reptiles~~ because ~~body length~~length correlates tightly with other anatomical measurements. Reptile body length has been shown to correlate with specific ~~including head length (e.g., $R^2 \geq 0.97$ for extant crocodylians [31]), head width (e.g., $R^2 \geq 0.85$ for extant crocodylians [28]), lengths of individual~~ cranial ~~elements~~ bones (e.g., ~~-~~R^2 = 0.89 for dentary length in acrodontan lizards [9]; $R^2 \geq 0.78$ for various cranial elements in the lizard genera *Anolis* and *Pholidoscelis* [31]), head length (e.g., $R^2 \geq 0.97$ for extant crocodylians [32]),

head width (e.g., $R^2 \geq 0.85$ for extant crocodylians [28]), femur length (e.g., $R^2 \geq 0.99$ for extant crocodylians [27]), ~~or~~ and vertebral element width (e.g., $R^2 \geq 0.81$ for extant boine snakes [33]). Mandible length has also been used as a direct proxy for body size in fossil lizards [21,34].

Snout-vent length (SVL), measured from the tip of the snout to the cloacal opening at the base of the tail (Fig 1), is often the preferred measure of body length in extant lizard studies [22,31]. This is because many lizards can autotomize and even regenerate their tails [35–39], thus lizard tail measurements are highly variable. Total body length is also difficult to reconstruct in lizards because the tail rarely possesses its original length in any extant specimen and is often disarticulated in dry skeletonized specimens. Measurements of SVL are more feasible to obtain from ~~live,~~ wet preserved~~,~~ or dry skeletonized extant lizard specimens. Maximum SVL strongly correlates with mean adult SVL and SVL at sexual maturity in extant lizards [3,40], so maximum SVL is a good metric of mean adult body size for a population of lizards sampled from the fossil record. Furthermore, once SVL is estimated, lizard body mass can be calculated from family-specific SVL-to-mass equations [24,41,42].

Fig 1. Measurement of snout-vent length (SVL). SVL was measured from the tip of the snout at the premaxilla to the posterior edge of the centrum on the second caudal vertebra, which is the approximate position of the middle of the cloaca (vent). SVL measurement is indicated by the blue arrow. Scale bar = 1 cm. Specimen pictured is *Gerrhonotus infernalis* (Anguidae; FMNH 22452).

For this study, ~~I sample~~ lizards were sampled from the Paleogene record (23-66 Mya) in the Western Interior of North America, specifically, from localities concentrated in the United

States (Fig 2). The Paleogene is an interesting period to study because it spans significant warming and cooling events [43,44] and chronicles the radiation of mammals [45–55] and later squamates [56–60] following the End-Cretaceous Mass Extinction. T~~I use t~~his ~~geotemporal system~~timeframe was used here -as a study system ~~as a paleontological framework~~ because it has a prolonged and robust fossil record in the Western Interior, preserving many diverse and ecologically important extant lizard clades that still occur in the ~~Western Interior~~area.~~,~~ ~~such as~~These include anguids and pleurodontan iguanians (e.g., iguanids and ~~polychrotids~~dactyloids), as well as clades that were extirpated from the region during the Paleogene, such as varanids and shinisaurids. Natural history museum collections in the United States include many Paleogene lizard specimens identified to the level of family or even genus.

Fig 2. Map showing all localities for fossil lizard data from across the Western Interior of North America through the Paleogene. Featured area is highlighted in inset map. Data points represent only specimens for which the locality could be georeferenced. Some taxonomic groups occurring with others in a particular locality may not be visible. Base maps were made with Natural Earth and are reprinted from Natural Earth under a CC Public Domain license, original copyright 2009. All other data contained were collected by the author. Maps were generated using QGIS-LTR 3.22.6 (download.qgis.org). The figure created is under the CC-BY 4.0 license, as determined by th~~e~~e authors of QGIS.

This study~~I~~ tested the hypothesis that proportions of individual bone lengths to SVL are conserved within each lizard lineage and that these proportions can therefore be used to predict SVLs within crown squamate groups (exclusive of snakes) for fossil lizard specimens identified

to those same groups. T~~I test t~~his hypothesis was tested using eight taxonomic lizard groups (seven families + Iguania —~~,~~ the latter encompassing the ~~polytomy of~~ iguanian families, not all of which have clear taxonomic relationships [61,62]) that are represented in the Paleogene fossil record and still exist today. For each crown group represented, ~~I generate~~ regressions of individual cranial and limb bones to SVL were generated. ~~and validate these regressions using~~ B~~b~~oth extant and fossil lizard skeletons were used as test specimens to validate the regressions. ~~I then use these regressions~~The equations were then used to reconstruct body size for crown group lizards sampled from across the U.S. Western Interior through the Paleogene. This is the first study to reconstruct lizard body size evolution in different crown groups over a prolonged geologic interval and across a continental interior. The results have broad implications for modes of body size evolution and resource zone partitioning in lizards. In addition to applications for fossil research, the extensive dataset and body size estimation methods presented here can be of great use to functional morphologists and herpetologists.

Institutional abbreviations

AMNH, American Museum of Natural History, New York, NY; **CAS**, California Academy of Sciences, San Francisco, CA; **CJB**, Christopher J. Bell, University of Texas at Austin, Austin, TX; **CM**, Carnegie Museum of Natural History, Pittsburgh, PA; **DMNH**, Denver Museum of Natural History (Denver Museum of Nature and Science), Denver, CO; **FLMNH**, Florida Museum of Natural History, Gainesville, FL; **FMNH**, Field Museum of Natural History, Chicago, IL; **FOBU**, Fossil Butte National Monument, Kemmerer, WY; **GCVP**, Georgia College Vertebrate Paleontology, Milledgeville, GA; **LACM**, Natural History Museum of Los Angeles County, Los Angeles, CA; **MCZ**, Museum of Comparative Zoology, Cambridge, MA;

MSU, Michigan State University, Lansing, MI; **MVZ**, Museum of Vertebrate Zoology, Berkeley, CA; **NMMNH**, New Mexico Museum of Natural History, Albuquerque, NM; **PTRM**, Pioneer Trails Regional Museum, Bowman, ND; **RAM**, Raymond M. Alfe Museum of Paleontology, Claremont, CA; **SMM**, Science Museum of Minnesota, Saint Paul, MN; ~~**TMM**~~**TxVP**, Texas ~~Memorial Museum~~Vertebrate Paleontology Collection, The University of Texas, Austin, TX; **UCM**, University of Colorado Museum, Boulder, CO; **UCMP**, University of California Museum of Paleontology, Berkeley, CA; **UMMP**, University of Michigan Museum of Paleontology, Ann Arbor, MI; **UNSM**, University of Nebraska State Museum, Lincoln, NE; **USNM**, United States National Museum (Smithsonian National Museum of Natural History), Washington, DC; **UW**, University of Wyoming Geological Museum, Laramie, WY; **UWBM**, University of Washington Burke Museum, Seattle, WA; **YPM**, Yale Peabody Museum, New Haven, CT.

Materials & methods

Data collection

~~I collected~~A ~~a~~natomical measurements were collected from 283 fossil lizard specimens, including 44 recognized genera and at least 33 recognized species, from the Paleogene record of the U.S. Western Interior (Table 1, S1 Dataset). Not all specimens that were attributed to a genus could be attributed to a specific species. ~~I only sampled~~Only specimens that included complete cranial or limb elements (e.g., Fig 3) were sampled. A few specimens included complete skulls (Fig 3A) or even skeletons (Figs 4 and 5). ~~I grouped~~ T~~t~~he fossil specimens were grouped by North American Land Mammal Age (NALMA), which compose a relative chronology used to

divide the Paleogene based on taxonomic turnover in the mammal fossil record [63]. Age ranges for these intervals are listed in Table 2 based on Barnosky et al. [64].

Table 1. Summary of datasets. The extant species totals include subspecies. Fossil species totals do not include specimens that could not be identified ~~past the level of genus~~to a specific genus or species (e.g., Anguidae indet., *Glyptosaurus sp.*~~, Anguidae indet.~~). For example, all three fossil teiid specimens sampled were identified as only "Teiidae indet."

Taxonomic group	# Extant specimens sampled	# Extant genera sampled	# Extant species sampled	# Fossil specimens sampled	# Fossil genera sampled	# Fossil species sampled
**Anguidae**	66	4	17	218	16	17
**Varanidae**	47	1	17	6	1	1
**Teiidae**	33	5	11	3	0	0
**Scincidae**	28	8	19	4	3	1
**Xantusiidae**	20	1	3	19	2	2
**Xenosauridae**	17	1	3	7	4	3
**Iguania**	55	9	16	19	8	6
**Shinisauridae**	N/A	N/A	N/A	7	3	3
**Total**	**266**	**29**	**86**	**283**	**37**	**33**

Formatted Table

Fig 3. Paleogene angui~~morphd~~ lizards from the Western Interior of North America and extant morphological analogues. (A) *Peltosaurus granulosus*† (~~Glyptosaurinae;~~ Anguidae) skull in right lateral view, and B) right humerus in medial view (AMNH 42913), indicated by

arrow, Orellan. (C) *Gerrhonotus infernalis* (Anguidae) skull and right mandible in right lateral view and (D) left humerus in medial view (FMNH 22452), extant. A-D are oriented with anterior to the right. (E) *Heloderma suspectum* (Helodermatidae) skull and left mandible in left lateral view, and (F) right humerus in medial view (UCMP 117512), extant. (G) *Helodermoides tuberculatus*† skull, mandibles, and cervical osteoderms in right lateral view, reflected for continuity (USNM V 13869), Chadronian. (H) *Helodermoides tuberculatus*† (~~Glyptosaurinae;~~ Anguidae) left mandible in lateral view, and (I) right humerus in medial view, reflected for continuity (UNSM 4511), Chadronian. E-I are oriented with anterior to the left. Scale bar = 1 cm. USNM V 13869 (G) image courtesy of the Smithsonian Institution.

Fig 4. Paleogene varanids from the Western Interior of North America. (A) *Saniwa ensidens*† ~~(Varanidae)~~ skeleton (FMNH PR 2378), Wasatchian. Scale bar = 10 cm. (B) *Saniwa sp.*† dentaries, vertebrae, and fragments (DMNH EPV.34588), Bridgerian. Scale bar = 3 cm.

Fig 5. Other complete Paleogene lizard skeletons from the Western Interior of North America. (A) *Bahndwivici ammoskius*† (Shinisauridae) skeleton (FMNH PR 2260), Wasatchian. Scale bar = 3 cm. (B) *Afairiguana avius*† (Iguania~~dae~~; Polychrotidae) skeleton (FMNH PR 2379), Wasatchian. Scale bar = 3 cm.

Table 2. Lizard crown group diversity by NALMA interval. ~~This table does not represent the full taxonomic diversity of Paleogene lizards represented in the U.S. Western Interior fossil record; it only represents groups for which I found complete fossil cranial or limb bones.~~ Expected diversity based on the Paleobiology Database (PBDB; www.paleobiodb.org) is

provided for comparison but should be taken as a coarse estimate because there may be some identification error in PBDB entries. Entries that were deemed inaccurate based on literature review were disregarded. Some data are also missing from the PBDB (e.g., no specific varanid genera or species are listed for the Paleogene within the United States). Iguanian specimens are identified by specific family rather than as “Iguania” in the PBDB, so comparison for that group was not possible. This table does not represent the full taxonomic diversity of Paleogene lizards represented in the U.S. Western Interior fossil record; it only represents groups for which complete fossil cranial or limb bones were measured. Fossil genera and species totals per family per NALMA do not include specimens that could not be identified to a specific genus or species, respectively (e.g., Anguidae indet.; *Glyptosaurus* sp.). Hence, some genera totals are zero. Specimens tentatively referred to a specific genus or species (e.g., *c.f. Glyptosaurus*) are counted toward that genus and/or species. NALMA age ranges are from Barnosky et al. [64].

Epoch	NALMA (Mya)	Lizard crown groups sampled	# of recognized genera sampled / expected from PBDB	# of recognized species sampled / expected from PBDB
Paleocene	Puercan (66.0 –– 63.8)	AnguidaeAnguidae Scincidae	1 / 2	1 / 2
		Scincidae	1 / 0	1 / 0
	Torrejonian (63.8 –– 60.9)	AnguidaeAnguidae Varanidae Xantusiidae	2 / 4	2 / 5
		Varanidae	1 / 0	0 / 0
		Xantusiidae	1 / 1	1 / 1

Formatted Table

Formatted: Centered, Indent: Left: 0.08", Right: 0.08"

	Tiffanian (60.9 – 56.2)	Anguidae~~Anguidae~~ ~~Scincidae~~ ~~Shinisauridae~~ ~~Xenosauridae~~	3 / 4	3 / 5
		Scincidae	1 / 0	0 / 0
		Shinisauridae	1 / 0	1 / 0
		Xenosauridae	1 / 0	1 / 0
	Clarkforkian (56.2 – 54.9)	Anguidae	3 / 0	3 / 0
Eocene	Wasatchian (54.9 – 50.5)	Anguidae~~Anguidae~~ ~~Iguania~~ ~~Scincidae~~ ~~Shinisauridae~~ ~~Varanidae~~ ~~Xantusiidae~~ ~~Xenosauridae~~	10 / 10	8 / 14
		Iguania	4 / 0	3 / 0
		Scincidae	1 / 0	0 / 0
		Shinisauridae	2 / 1	2 / 1
		Varanidae	1 / 0	1 / 0
		Xantusiidae	1 / 2	0 / 4
		Xenosauridae	2 / 0	1 / 0
	Bridgerian	Anguidae~~Anguidae~~	4 / 8	2 / 7

Formatted: Centered, Indent: Left: 0.08", Right: 0.08"

	(50.5 - 46.2)	~~Iguania~~<br>~~Teiidae~~<br>~~Varanidae~~<br>~~Xantusiidae~~<br>~~Xenosauridae~~		
		Iguania	1 / 0	0 / 0
		Teiidae	0 / 0	0 / 0
		Varanidae	1 / 0	0 / 0
		Xantusiidae	2 / 1	1 / 1
		Xenosauridae	1 / 0	0 / 0
	Uintan<br>(46.2 - 39.7)	Anguidae~~Anguidae~~<br>~~Shinisauridae~~<br>~~Varanidae~~	1 / 4	0 / 6
		Shinisauridae	1 / 0	0 / 0
		Varanidae	1 / 0	1 / 0
	Duchesnean<br>(39.7 - 37.0)	Anguidae	0 / 0	0 / 0
	Chadronian<br>(37.0 - 33.9)	Anguidae~~Anguidae~~<br>~~Iguania~~<br>~~Xantusiidae~~	3 / 2	3 / 2
		Iguania	2 / 0	2 / 0
		Xantusiidae	1 / 0	1 / 0
Oli go	Orellan	Anguidae~~Anguidae~~	2 / 1	2 / 1

Formatted: Centered, Indent: Left: 0.08", Right: 0.08"

	(33.9 - 31.8)	~~Iguania~~ ~~Xenosauridae~~		
		Iguania	1 / 0	1 / 0
		Xenosauridae	1 / 1	1 / 1
	Whitneyan (31.8 - 29.5)	Anguidae	1 / 0	1 / 0
	Arikareean (29.5 - 23.0)	Anguidae	1 / 1	1 / 1

To compare the fossil lizard specimens with extant congeners, ~~I measured~~ 266 extant specimens were measured (S2 Dataset), comprising 30 genera and 86 species (Table 1) from the same crown group lineages that were represented in the fossil dataset. These included seven extant families: Anguidae (e.g., alligator lizards and galliwasps), Scincidae (skinks), Shinisauridae (crocodile lizards), Teiidae (e.g., whip-tail lizards), Varanidae (monitor lizards), Xantusiidae (night lizards), and Xenosauridae (knob-scaled lizards). There were also several pleurodontan iguanian families represented in the fossil dataset (e.g., Iguanidae and Polychrotidae), so these were ~~I~~ treated ~~these~~ as a collective group, "Iguania," in both datasets. Phylogenetic relationships among iguanian families are poorly resolved, but the group "Iguania" is supported by both morphological and molecular analyses [61,62,65,66]. ~~I focused the~~ Eextant data collection in this study focused on dry skeletonized specimens to obtain measurements of individual bones as well as SVL from the same specimens. Juvenile specimens were excluded from the extant dataset. Further details on data collection procedures and considerations are provided in ~~Supplementary Information~~Supporting Information.

No permits were required for the described study, which complied with all relevant regulations.

Generating and testing regressions

Researchers commonly use bivariate linear regressions to estimate SVL for fossil vertebrates using anatomical measurements from specimens of the most inclusive phylogenetic group [67]. This study~~I~~ regressed anatomical elements – including skull length, individual cranial bone length (dentary, mandible, maxilla, frontal, parietal), and limb bone length (humerus, femur, tibia) – to SVL for the extant lizard groups in the~~his~~ dataset presented (Table 3, S1-~~1 Fig~~S2 Fig, S1-S7 Tables). ~~I used~~ R~~r~~educed major axis (RMA) linear regressions were used because RMA accommodates error in both the dependent and independent variable measurements [68]. All measurements were transformed using natural log, which ~~. Natural log~~ is commonly ~~used~~ applied in analyses of fossil vertebrate body size [9,29,49,52,53,69]. Tests of natural log compared to base 10 log regressions also produced the same SVL prediction for large test specimens in this study, so ~~I used~~ natural log was used in all regressions for consistency. All regression analyses were performed using PAST v.4.03 [68].

Table 3. Regression functions and associated statistics for SVL estimation from ~~anatomical elements~~individual bones. All functions were transformed using natural log (LN~~n~~). Standard error for slope and intercept terms are listed after the slope and intercept in the equations. SEE = Standard error of the estimate. Functions are listed in order of increasing SEE for each group. P(uncorrelated)~~ =~~ < 0.001 for all functions.

Group	~~Element~~Predictor	*n*	Slope *b*	Standard error of *b*	Intercept	Standard error of intercept	R^2	SEE
Anguidae	Skull Length	46	0.946	0.0417	1.71	0.129	0.91	0.0740
	Femur Length	43	0.888	0.0410	2.46	0.101	0.91	0.0742
	Mandible Length	43	0.921	0.0424	1.82	0.129	0.91	0.0760
	Humerus Length	41	0.889	0.0451	2.57	0.106	0.90	0.0815
	Parietal Length	24	1.03	0.0658	2.29	0.148	0.91	0.0853
	Maxilla Length	22	1.02	0.0803	2.40	0.170	0.88	0.0992
	Frontal Length	22	1.27	0.111	2.13	0.216	0.85	0.120
	Dentary Length	24	0.898	0.0816	2.43	0.195	0.82	0.121
Varanidae	Tibia Length	27	0.953	0.0282	2.43	0.103	0.98	0.0695
	Maxilla Length	25	1.13	0.0423	2.04	0.145	0.97	0.0837
	Dentary Length	23	1.13	0.0845	1.86	0.305	0.88	0.157
Xenosauridae	Dentary Length	12	0.36	0.164	0.921	0.450	0.85	0.0530
	Parietal Length	12	1.03	0.154	2.17	0.369	0.78	0.0661
	Frontal Length	12	1.05	0.185	2.16	0.435	0.69	0.0787
Teiidae	Dentary Length	26	0.677	0.0686	2.70	0.165	0.75	0.0995
Scincidae	Maxilla Length	27	1.06	0.0352	2.20	0.0850	0.97	0.115
	Dentary Length	27	1.12	0.0426	1.74	0.115	0.96	0.133
Xantusiidae	Maxilla Length	20	0.698	0.0659	2.82	0.0944	0.88	0.119
	Dentary Length	20	0.787	0.0869	2.58	0.134	0.89	0.124
Iguania	Skull Length	53	1.15	0.0248	1.06	0.0838	0.98	0.0997
	Maxilla Length	55	1.07	0.0303	2.07	0.0824	0.96	0.133
	Dentary Length	55	1.22	0.0402	1.38	0.118	0.94	0.155

Formatted: Font: Not Italic

Formatted Table

Formatted: Font: Not Italic

Formatted: Font: Not Italic

To test the assumption that anatomical proportions are conserved between extant and extinct members of the lizard lineages represented in this study, ~~I tested~~ the regressions were tested using complete skeletons ~~specimens~~ of both extant and extinct taxa. The fossil test specimens included three of the few available complete fossil lizard skeletons that were~~I~~ measured: a large-bodied varanid (*Saniwa ensidens*, FMNH PR 2378, ~~Fig 4A~~SVL 425 mm which is smaller than the largest extant varanid measured in this study (*V. exanthematicus*, 770 mm); Fig 4A), a small-bodied anguid (*Parophisaurus pawneensis*, YPM VP 060609, ~~which was~~SVL 138 mm; not yet published at the time of this publication and thus not pictured here), and a very small-bodied polychrotid iguanian (*Afairiguana avius*, FMNH PR 2379, SVL 46 mm;~~,~~ Fig 5B). ~~I also measured the~~The only known complete skeleton of a fossil shinisaurid, *Bahndwivici ammoskius* (FMNH PR 2260, SVL 154 mm; Fig 5A) was also measured and ~~, and~~ used ~~it~~ to estimate SVL for the other fossil shinisaurid specimens based on anatomical ratios because complete dry skeletonized extant specimens for Shinisauridae were not available. See ~~Supplementary Information~~Supporting Information for more details on materials and methods.

Estimating body mass for fossils

Body mass scales linearly with SVL in extant lizards with limbs [15,24]. Other researchers developed family-level ~~Meiri developed general lizard~~ SVL-to-mass allometric equations for extant lizards (S8 Table) [24,41,42] ~~(RMA: Body Mass in kg = (((3.088±0.067)*SVL) – (4.852±0.128))/1000, R^2 = 0.946)~~ as well as ~~family-level equations based on measurements of extant taxa~~general SVL-to-mass equations for all lizards (RMA: Body Mass in kg = (((3.088±0.067)*SVL) – (4.852±0.128))/1000, $R^2 = 0.946$) [24]. Body mass for

each fossil specimen in the dataset presented here (S1 Dataset) was calculated using b~~I applied~~ ~~b~~oth the family-level (S8 Table) and general RMA equations from these published studies for comparison~~to the SVL estimates from this study to calculate body mass for each fossil specimen in this dataset (S1 Dataset)~~. ~~If a family-level equation was not provided for a given family represented in this study, I used the general equation. The equation for Anguidae was less reliable, so the range between that estimate and the general estimate is listed for anguid specimens in Table 3.~~

New equations were also generated for estimating lizard body mass directly from individual bones (S1-S7 Tables). This was done by combining the equations generated in this study for estimating SVL from individual bones (S1-S7 Tables) with the equations published elsewhere for estimating lizard body mass from SVL (S8 Table). The resulting equations produced the same body mass estimates for the fossil lizards as the estimates obtained from the previously published equations (S1 Dataset).

Results ~~& discussion~~

Estimating body size of fossil lizards

~~The regressions provided here can generate SVL estimates for any fossil lizard specimen referred to the crown groups represented in this dataset if the fossil includes a complete cranial or limb bone. These methods (S2 Fig) can also be used to generate regressions for any extant lizard group not included in this study.~~

Skull and mandible length consistently provided reliable regressions for estimating SVL (SEE $\leq$ 0.0997) across relevant taxonomic groups represented in this study (Table 3, S1-S7 Tables). These measurements are known to correlate with SVL widely among extant lizards

[12,21,22]. Regressions based on limb measurements (femur, humerus, or tibia length) were also ~~strong~~ consistently accurate across groups (SEE ≤ 0.0815). Regressions based on dentary, maxilla, frontal, and parietal length had the greatest variation in rankings among lizard groups (SEE 0.0530 – 0.1571). Cranial element morphology often varies between closely related lizard groups, more so than proportions of head or limb length to SVL [70]. This may be the reason why attempts to calculate general regression equations for this study proved unsuccessful: none of the general equations passed tests for both homoskedasticity and normal distribution of residuals (S9~~8~~ Table). Meiri's [24] general equation for predicting mass from SVL for lizards (see S1 Dataset) was more viable likely because the relationship between SVL and mass appears to be more consistent than the relationship between head length or any individual element to SVL across lizard taxonomic groups.

Formatted: Not Highlight

The group-level regressions generated in this study predicted actual SVL with comparable accuracy between the extant and fossil test specimens. The actual SVL for each extant and fossil test specimen fell within or close to the 95% confidence interval for the estimated SVL (S1-S7 Tables). The percentage difference between predicted and actual SVL of test specimens ranged from 1% (varanid tibia length vs. SVL, SEE = 0.0695; S2 Table) to 36% (anguid frontal length vs. SVL, SEE = 0.120; S1 Table). Clearly, some anatomical elements provide more accurate estimates than others for different lizard groups.

Taxonomic turnover

Anguids are known to be the most abundant lizards in the Paleogene fossil record of the Western Interior [71–73] and that was reflected in this study by their unrivaled taxonomic richness, which included at least 15 genera (Figs 6-7, Table 2, S1 Dataset). The results presented

here also indicated that taxonomic diversity was greatest in the early Eocene and decreased in the early Oligocene (Figs 6-7, Table 2). Rarefaction analysis indicated that the number of crown groups recovered within a given NALMA peaked around a sample size of 50 specimens (S3 Fig). The Orellan sample included 64 specimens (Fig 6, S1 Dataset) and yet recovered only three crown groups (Table 2, S3 Fig), supporting the interpretation of a decline in diversity in the late Paleogene.

Fig 6. Snout-vent length distribution by taxonomic group for fossil lizards in the Western Interior of North America through the Paleogene. Sample sizes are per North American Land Mammal Age (NALMA). Data points are arbitrarily spread within each NALMA for visibility. NALMA abbreviations: Pu = Puercan, To = Torrejonian, Ti = Tiffanian, Cf = Clarkforkian, Wa = Wasatchian, Br = Bridgerian, Ui = Uintan, Du = Duchesnean, Ch = Chadronian, Or = Orellan, Wh = Whitneyan, Ar = Arikareean. Data points marked with an asterisk (*) were measured from complete fossil skeletons. All others were estimated from individual cranial or limb elements using regressions (see S1-S7 Tables). All measurements are transformed using natural log.

Fig 7. Body mass distribution by taxonomic group for fossil lizards in the Western Interior of North America through the Paleogene. Sample sizes are per North American Land Mammal Age (NALMA). Data points are arbitrarily spread within each NALMA for visibility. NALMA abbreviations: Pu = Puercan, To = Torrejonian, Ti = Tiffanian, Cf = Clarkforkian, Wa = Wasatchian, Br = Bridgerian, Ui = Uintan, Du = Duchesnean, Ch = Chadronian, Or = Orellan, Wh = Whitneyan, Ar = Arikareean. Masses were calculated with snout-vent length using the published equations listed in S8 Table. Data points marked with an asterisk (*) used SVLs

measured from complete fossil skeletons. All others used SVLs estimated from individual cranial or limb element lengths using regressions (see S1-S7 Tables). All measurements are transformed using natural log.

The observed patterns may reflect some taphonomic or preservation bias (especially with respect to smaller bones), gaps in the rock record, and selective sampling by fossil collection crews. However, rarefaction analysis (S3 Fig) indicated that this study adequately sampled the upper range of body size diversity represented in this fossil record, especially from thoroughly documented areas like the Bighorn and Green River Basins (S1 Dataset; for further discussion, see the censuses of microvertebrate localities in this system by Smith [56–60]). Preservation bias toward complete elements from adult individuals may have mitigated other sources of bias when reconstructing maximum body size through geologic intervals.

Formatted: Indent: First line: 0.5"

The observed late Paleogene reductions in maximum body size represent the extinction of larger taxa [74,75]. Even in extant lizards, extinction risk increases with body size [3]. Among the eight lizard groups documented in this study from the Paleogene record of this region, only Anguidae, Scincidae, Teiidae, and Xantusiidae still occur in the area. Xenosaurids and the iguanians included in this study that occurred in the Western Interior during the Paleogene have since been extirpated from the region; varanids and shinisaurids no longer occur anywhere in North America [4,11,13,76]. For further discussion, see Supporting Information.

Body size evolution in fossil lizards

Maximum lizard SVL remained below 300 mm through the early and middle Paleocene (Puercan – Tiffanian, Fig 6, Table 4, S1 Dataset). This upper SVL limit increased to almost 500

mm and maximum body mass exceeded 1 kg around the Paleocene-Eocene transition (Clarkforkian; Fig 7). By the Wasatchian, maximum SVL reached 750 mm~~ and maximum body mass exceeded 1 kg~~, and ~~. Maximum SVL~~ surpassed 1 m by the Bridgerian (Table 4).

~~**Fig 6. Snout-vent length distribution by taxonomic group for fossil lizards in the Western Interior of North America through the Paleogene.** Sample sizes are per North American Land Mammal Age (NALMA). Data points are arbitrarily spread within each NALMA for visibility. NALMA abbreviations: Pu = Puercan, To = Torrejonian, Ti = Tiffanian, Cf = Clarkforkian, Wa = Wasatchian, Br = Bridgerian, Ui = Uintan, Du = Duchesnean, Ch = Chadronian, Or = Orellan, Wh = Whitneyan, Ar = Arikareean. Data points marked with an asterisk (*) were measured from complete fossil skeletons. All others were estimated from individual cranial or limb elements using regressions (see S1-S7 Tables). All measurements are transformed using natural log.~~

Table 4. Maximum lizard body size estimates per NALMA interval. NALMA age ranges are from Barnosky et al. [64]. ~~Note that the single specimen from the Duchesnean is substantially lower than the maxima listed for preceding and successive intervals and likely does not represent the accurate maximum for that time bin. Mass estimates are based on Meiri [24]: for anguids, both family-specific (lower) and general estimates (higher) are listed. Otherwise, only family-specific estimates are given.~~

NALMA	Age Range (Mya)	Crown Group	Binomial	Specimen	Max SVL Estimate (mm)	Mass Estimate (kg)

Puercan	66.0-63.8	Anguidae	*cf. Odaxosaurus piger*	UCM 34991	91	0.311~~0.141-0.276~~
Torrejonian	63.8-60.9	Varanidae	cf. Varanidae indet.	UCMP 2671	191	0~~.612~~.615
Tiffanian	60.9-56.2	Anguidae	*Melanosaurus sp.*	UCM 98615	256	0.~~401-0.785~~885
Clarkforkian	56.2-54.9	Anguidae	*Melanosaurus maximus*	UMMP 74618	487	1.69~~0.765-1.50~~
Wasatchian	54.9-50.5	Anguidae	*Melanosaurus sp.*	UCMP 154536	788	~~1.24-2.43~~2.74
Bridgerian	50.5-46.2	Anguidae	*Glyptosaurus sylvestris*	USNM 12590	1056	~~1.66-3.26~~3.67
Uintan	46.2-39.7	Varanidae	*Saniwa ensidens*	FMNH UC 1719	540	~~1.74~~1.74
Duchesnean	39.7-37.0	Anguidae	Anguidae indet.	CM 42469	116	~~0.181-0.354~~0.399
Chadronian	37.0-33.9	Anguidae	*Helodermoides sp.*	UW 11057	1296	~~2.04-4.00~~4.51
Orellan	33.9-31.8	Anguidae	*Peltosaurus sp.*	UCM 20877	360	~~0.565-1.11~~1.25
Whitneyan	31.8-29.5	Anguidae	*Peltosaurus sp.*	SMM P81.8.71	224	~~0.352-0.688~~0.775
Arikareean	29.5-23.0	Anguidae	*Peltosaurus sp.*	UNSM 81001	351	~~0.511-1.08~~1.22

Formatted: Not Highlight

Formatted: Not Highlight

Formatted: Not Highlight

Formatted: Not Highlight

Formatted: Not Highlight

Formatted: Not Highlight

Formatted: Not Highlight

Formatted: Not Highlight

Formatted: Not Highlight

Formatted: Not Highlight

Formatted: Not Highlight

Formatted: Not Highlight

The pattern for the middle Eocene (Uintan – Duchesnean) was dubious. The observed maximum ~~SVL~~ body size for the middle Eocene (540 mm, 1.74 kg, *Saniwa ensidens*, Figs 6-7,

Table 4) was much lower than the early or late Eocene ~~(540 mm, *Saniwa ensidens*, Fig 6, Table 4)~~. Fewer outcrops are available from this interval in the U.S. Western Interior, so this result was likely an artifact of poor sampling (n = 4 for the Uintan, n = 1 for the Duchesnean, Fig 6). R~~The r~~arefaction analysis (S4~~3~~ Fig) demonstrated~~s~~ that the number of expected size groups recovered in a sample from this fossil dataset again peaked and leveled off around n = 50, supporting the assumption that the NALMAs with small sample sizes in this study did not capture the full range of lizard body size that was likely present during those intervals.

The sample size was higher for the late Eocene (n = 24 for the Chadronian, Fig 6). The Chadronian record included several large individuals, including the largest ~~maximum SVL~~ body size estimated in this study (1.3 m, 4.5 kg, *Helodermoides sp.*, Table 4). By the early Oligocene (Orellan), maximum SVL dropped to 360 mm (*Peltosaurus sp.*) and remained below that limit through the late Paleogene. The Orellan was very well sampled (n = 64) compared to other NALMAs, and consequently~~.~~, sample size did not correlate strongly with maximum estimated SVL across the dataset (R^2 = 0.23, p(uncorr.) = 0.11). Together with -r~~R~~arefaction analysis (S4~~3~~ Fig), these results indicate~~d~~ that the~~is~~ observed decrease in maximum lizard SVL in the late Paleogene i~~wa~~s likely valid.

~~The most common lizard clade in the Western Interior through the Paleogene was an extinct subgroup of anguid lizards, Glyptosaurinae [71–77]. Glyptosaurinae has an exceptionally robust fossil record in terms of known taxa and number of specimens collected and published [75]. Glyptosaurinae has strong support for placement within crown Anguidae [63,71,72,78]. The largest Paleogene lizards documented in this study are referred Glyptosaurinae (up to 1.3 m and at least 2 kg (*Helodermoides*, Fig 3G); Fig 6, Table 4, S1 Dataset). Several glyptosaurine~~

taxa exceeded 1 kg in body weight during the Eocene (S1 Dataset, Table 4), which makes their body masses comparable to those of medium-sized mammals (> 1 kg and < 50 kg [45]). The largest glyptosaurines seemed to have consistent maximum sizes within genera. For instance, eight frontals of *Glyptosaurus* were recovered from two different NALMA intervals (Wasatchian and Bridgerian, Early Eocene), all measuring in the upper range of 38-45 mm, and two frontals of *Paraglyptosaurus* measuring 40-45 mm came from the same NALMAs (S1 Dataset).

Among the eight lizard crown groups sampled, only two reached SVLs over 200 mm: Anguidae and Varanidae (Fig 6). Interestingly, body size in the largest extant limbed anguid, *Diploglossus millepunctatus* (maximum SVL 280 mm, mass 0.439-0.860 based on Meiri [4,24]), does not approach the upper limit exhibited by large Eocene anguids, nor do the largest lizards occurring in North America today (*Heloderma horridum*, maximum SVL 520 mm [79], Fig 3E; *Iguana iguana*, maximum SVL 580 mm [4]). Conversely, Varanidae includes the largest extant lizard, *Varanus komodoensis*, which exceeds the adult size of the largest Eocene anguid at around 1.5 m [4,80], but this clade is now restricted to the Eastern Hemisphere [13].

Taxonomic turnover

The results of this study indicated that taxonomic diversity was greatest at the family level for the clades sampled in the early Eocene and then decreased in the early Oligocene (Fig 6, Table 2). Rarefaction analysis indicated that the number of crown groups recovered within a given NALMA peaked above a sample size of 50 specimens (S4 Fig). I sampled 64 specimens from the Orellan (Fig 6, S1 Dataset) and yet recovered only three crown groups (Table 2, S4 Fig), supporting the interpretation of a decline in diversity in the late Paleogene.

Anguids are known to be the most abundant lizards in the Paleogene fossil record of the Western Interior [73,76,77] and that was reflected in this study by their unrivaled taxonomic richness, which included at least 15 genera (Fig 6, Table 2, S1 Dataset). Glyptosaurinae alone accounted for 11 genera, with taxonomic identifications dominated by *Glyptosaurus* and *Melanosaurus*. By the late Eocene (Chadronian), only one large glyptosaurine genus was present, *Helodermoides* (Fig 3G, S1 Dataset).

Most of the lizards in this geotemporal system did not experience an increase in body size range. Small-bodied lizards, including smaller anguid taxa, dominate the North American Paleogene lizard record. For example, 75% of the most common isolated bone in this dataset, the dentary, are < 26 mm in length with an estimated SVL of < 200 mm (S1 Dataset). This may reflect an ecological hierarchy in these paleocommunities. Large-bodied lizards are less abundant than smaller ones in extant ecosystems [3].

Additional bias in the observed patterns may result from taphonomic or preservation bias, gaps in the rock record, and selective sampling by fossil collection crews. For further discussion see the censuses of microvertebrate localities in this system by Smith [54–58]. However, the rarefaction analysis in S4 Fig would indicate that this study adequately sampled the diversity represented in this fossil record, especially from thoroughly documented areas like the Bighorn and Green River Basins (S1 Dataset). It is always possible that there were larger individuals present in this system. For Anguidae, at least, the number of individuals sampled indicates that the upper body size range observed here is as accurate as we can possibly know for that group (see S4 Fig). In any case, the preservation bias toward complete elements from adult individuals may help to mitigate other sources of bias when reconstructing maximum body size through geologic intervals.

The only diagnostic record of large glyptosaurines occurring outside the U.S. Western Interior was a Uintan skeleton from San Diego County, California, that was referred to *Glyptosaurus* [81]. Some small osteoderms from the Clarkforkian of South Carolina were also referred to Glyptosaurinae [82], but it was impossible to estimate the size of the animal that produced them. The largest Paleogene varanid from the Western Interior, *Saniwa ensidens* (Fig 4A), was also previously reported from Eocene deposits in California [83]. Thus, it appears that these large Eocene lizards were restricted to the Western half of the present-day United States.

The late Paleogene reductions in maximum body size represent the extinction of larger taxa [84,85]. Even in extant lizards, extinction risk increases with body size [3]. Among the eight lizard groups documented in this study from the Paleogene record of this region, only Anguidae, Scincidae, Teiidae, and Xantusiidae still occur in the area. Xenosaurids and the iguanians included in this study have since been extirpated from the Western Interior; varanids and shinisaurids no longer occur anywhere in North America [4,11,13,80]. For further discussion, see Supplementary Information.

Discussion

Formatted: PLOS One Level 1 Heading, Line spacing: single

Potential Climate as a potential drivers of Paleogene lizard body size evolution and diversity

Competition

Biotic interactions should at least partially drive body sizes in reptiles [9,86,87]. Early in the Paleogene, lizards may have been constrained to small body sizes due to competition, predation, or specializations related to resource zones of small taxa [88,89]. Changes in faunal

composition between the late Eocene and early Oligocene could similarly explain the loss of large lizards in the Western Interior and the eventual extinction of glyptosaurines.

The Eocene varanids did not reach the size of their extant congener *Varanus komodoensis*, the largest living lizard [13,80]. However, the largest glyptosaurines did reach the minimum length of *V. komodoensis*, over 1 m in SVL (Table 4; [4,80]). *Varanus komodoensis* does not have any natural predators [4,80], a factor which can release size constraints on extant lizards [3]. This was not the case for the large Eocene lizards in the Western Interior, which lived with many contemporaneous medium- and large-sized mammalian carnivores that could have preyed on lizards [45,90–94]. But unlike extant *V. komodoensis*, large glyptosaurines were heavily armored (Fig 3G; *V. komodoensis* has osteoderms in its skin as well but they are less heavily ossified [95]). It is possible that gigantism together with robust body armor protected large glyptosaurines from predation (see [96]).

The observed body size evolution patterns may also reflect some competition between lizard groups. The available data indicate that maximum body size in Anguidae, the most abundant group in this study that included large-bodied individuals, negatively correlated with maximum body size in Xantusiidae and Xenosauridae, two of the most common small-bodied groups (S5 Fig). These results indicate that as anguids evolved larger body sizes, maximum body size in the other two groups decreased. It is possible that this was an outcome of competition for resources between these groups.

Climate

Another factor that Environmental temperature can influence body size evolution in reptiless is climate change. Metabolic rate increases with temperature and scales with body size in all poikilothermic vertebrates [1,77,78], including lizards [15,79]. Changes in environmental

temperature are known to correlate globally with changes in maximum body size in extant lizards (exclusive of snakes [25]). Increased temperatures around the Paleocene-Eocene Thermal Maximum (PETM, 56 Mya; [44,80]) could have elevated metabolism and released a physiological constraint on maximum size for some lizard taxa. ~~[9,34].~~ Gigantism has been documented in other fossil reptiles in conjunction with elevated temperatures, including a boid snake (*Titanoboa*, total length 13 m, 60-58 Mya, Colombia [33]), a caimanine crocodyliform (*Purussaurus*, total length 12.5 m, 8 Mya, northern South America [81]), and a varanid lizard (*Varanus priscus*, total length up to 5.7 m, around 50 kya, Australia [82,83]).

To investigate this possibility, ~~I~~ RMA linear regression was applied to test whether ~~correlated~~ maximum and mean fossil lizard ~~body size~~SVL ~~with~~ predicted global temperature per NALMA (S5 Fig, S10 Table). G~~obtained~~lobal temperature data were obtained from Zachos et al. [43,44], a widely used dataset of Cenozoic paleotemperatures derived from marine oxygen isotopes (e.g., [10,49]). Here, fossil lizard body size showed no relationship to global temperature through the Paleogene ~~(S6 Fig, S9 Table)~~. It is possible that the relationship observed today is not detectable ~~on~~ across scales of millions of years. It might also be more pertinent to compare these data with terrestrial paleotemperature proxies from the same geotemporal system sampled in this study. This could be a fruitful area for~~of~~ continued research to build on the data presented here.

Alternatively, body size evolution patterns observed here could reflect indirect effects of climate [84]. Temperatures were warmer during both the summer and winter months around the PETM, and most precipitation came during the summer [85–87]. This would have allowed lizards to be active and growing during a longer portion of the year [84,88] and could also have led to greater food availability from increased productivity, especially in the summer months

Formatted: Not Highlight
Formatted: Not Highlight
Formatted: Not Highlight
Formatted: Not Highlight
Formatted: Not Highlight
Formatted: Not Highlight
Formatted: Not Highlight
Formatted: Not Highlight
Formatted: Not Highlight
Formatted: Not Highlight

[89–91]. Previous studies found elevated growth rates in extant tropical lizards occupying areas that received greater precipitation [92]. Attaining large body size in a closed tropical forest system with an equable climate, like that present in the Western Interior in the early Eocene [89,90,93,94], would also have allowed lizards to spend less time and energy on active thermoregulation [1,95]. Large body size sometimes corresponds with thermoconforming behavior and nocturnal activity in extant lizards [3,12,88], and these strategies could have reduced competition with diurnal organisms.

Herbivory can also lead to large body size in lizards [3,4,79]. This has been inferred for at least one giant fossil lizard [9]. However, the dentition of the largest lizards in this study were more indicative of omnivorous or insectivorous (Anguidae, Fig 3A) and carnivorous diets (Varanidae, Fig 4B; see [21,96]). It may be impossible to determine the specific mechanisms of gigantism in lizards in this system, but the results presented here elucidate clear patterns of body size evolution.

Formatted: Not Highlight

Formatted: Not Highlight

Formatted: Not Highlight

Body size evolution among taxonomic groups

Despite increased environmental temperatures in the early Eocene, most of the lizards in this geotemporal system did not experience an increase in body size range. Small-bodied lizards dominated the North American Paleogene lizard record. The maximum and mean body sizes for most lizard groups did not exceed 200 mm in SVL (LN(SVL) = 5.30, S6 Fig, S11 Table) or 1 kg in body mass (LN(Mass) = 0.00, S7 Fig, S11 Table). Maximum body size in extant lizards can stay comparatively low in warm climates due to behavioral thermoregulation [95], competition, predation, or specializations related to resource zones of small taxa [97,98]. The results presented

here may reflect an ecological hierarchy in the Paleogene communities of the Western Interior similar to extant ecosystems in which large-bodied lizards are less abundant [3].

Among the eight lizard crown groups sampled, only two reached SVLs over 200 mm and masses over 1kg: Anguidae and Varanidae (Figs 6-7, S6-S7 Figs, Table 4). Interestingly, the largest extant limbed anguid today, *Diploglossus millepunctatus* (maximum SVL 280 mm), does not approach the size of the largest anguids from the Eocene, nor do the largest lizards occurring in North America today (*Heloderma horridum*, maximum SVL 520 mm [99], Fig 3E; *Iguana iguana*, maximum SVL 580 mm [4]). Conversely, Varanidae includes both the largest extant lizard (*Varanus komodoensis*, which exceeds the adult size of the largest Eocene anguid at around 1.5 m SVL [4,76]) and the largest known terrestrial lizard in the fossil record (*Varanus priscus*, known from Pleistocene deposits in Australia, with SVL at least 3 m [82,83]), but varanids are now restricted to the Eastern Hemisphere [13].

Conclusions

This study offers the first survey and reconstruction of body size across crown lizard groups through a prolonged geologic interval and across a large continental interior region. The findings indicate that lizard body size peaked in the early Eocene. Only two lizard groups, Anguidae and Varanidae, reached large body sizes exceeding ~~, ~~1 m in SVL and 1 kg in mass. B~~while b~~ody size range ~~barely changed~~did not change considerably for other groups. ~~Most large-bodied lizards in the Eocene were heavily armored glyptosaurine anguids, some of which exceeded 1m in SVL and 1 kg in body mass.~~ Both maximum body size and taxonomic diversity decreased for lizards across the Western Interior in the early Oligocene. The methods presented

here can be applied to other lizard clades to generate reasonably accurate body size estimates for fossil taxa and to study patterns of body size evolution across higher taxonomic groups.

Acknowledgments

The author wishes to thank K. Padian, J. McGuire, A. Barnosky, and I. Wang for their mentorship and helpful comments on this work, as well as J. Head, who mentored the research that started this project. The following individuals provided assistance and access to museum collections: M. Norell, C. Mehling, J. Meng, D. Kizirian, L. Vonnahme, M. Arnold (AMNH); J. Vindum, L. Scheinberg, E. Ely (CAS); M. Lamanna, J. Padial, A. Henrici, S. Rogers (CM); T. Lyson, L. Ivy, K. MacKenzie (DMNH); J. Bloch, R. Hulbert, J. Bourque (FLMNH); P. Makovicky, L. Grande, A. Resetar, W. Simpson, A. Stroupe, J. Mata (FMNH); A. Aase (FOBU); N. Smith, S. McLeod, V. Rhue (LACM); J.Rosado (MCZ); J. McGuire, C. Spencer (MVZ); T. Williamson (NMMNH); A. Farke, G. Santos (RAM); A. Hastings, E. Whiting (SMM); C. Bell, M. Brown (TMM); T. Culver, N. Ridgwell (UCM); P. Holroyd, C. Marshall (UCMP); J. Wilson, A. Rountrey (UMMP); R. Hunt, Jr., P. Freeman, G. Corner, T. Labedz (UNSM); H. Dieter-Sues, M. Carrano, M. Brett-Surman, A. Millhouse (USNM); L. Vietti (UW); C. Sidor, R. Eng, M. Rivin (UWBM); J. Gauthier, C. Norris, D. Brinkman (YPM). The author thanks the following for helpful discussions: C. Badgley, C. Bell, B-A. Bhullar, J. Bloch, W. Clemens[†], C. Crumly, J. Gauthier, W. Gearty, P. Gingerich, L. Grande, A. Gunderson, P. Holroyd, E. Holt, R. Huey, R. Hunt, Jr., M. Kemp, D. Meyer, D. Miles, J. McGuire, J. Parham, H. Petermann, R. Secord, M. Stocker, R. Sullivan, D. Watkins, L. Weaver, S. Werning, E. Whiting, C. Williams, the MVZ Herp Group, the entire UCMP community. C. Bell also provided access to obscure literature. D. Meyer provided measurements of an unpublished specimen. G. Brown (UNSM) prepared a new *Peltosaurus* specimen (UNSM 12102) containing critical limb material. F. Vasconcellos provided the photo in Fig 3E. B. Chelemedos and C. Wong assisted with data collection. E. Whiting provided instruction on ~~how to use~~using QGIS to make maps. J. Lipps helped to design

a visually accessible color palette for Fig 2~~the figures~~. The author thanks ~~Carlo~~ C. Meloro, ~~Simon~~ S. Scarpetta, and an anonymous reviewer for their constructive comments which greatly helped to improve the manuscript.

References

1. Peters RH. The Ecological Implications of Body Size. 1st ed. Beck E, Birks HJB, Connor EF, editors. New York, NY: Cambridge University Press; 1983.

2. Schmidt-Nielsen K. Scaling: Why is Animal Size So Important? Cambridge: Cambridge University Press; 1984.

3. Meiri S. Evolution and ecology of lizard body sizes. Glob Ecol Biogeogr. 2008;17: 724–734. doi:10.1111/j.1466-8238.2008.00414.x

4. Meiri S. Traits of lizards of the world: Variation around a successful evolutionary design. Glob Ecol Biogeogr. 2018;27: 1168–1172. doi:10.1111/geb.12773

5. Damuth J. Problems in estimating body masses of archaic ungulates using dental measurements. Body Size Mamm Paleobiol Estim Biol Implic. 1990;229: tables 16.9-16.10.

6. Figueirido B, Pérez-Claros JA, Hunt RM, Palmqvist P. Body mass estimation in amphicyonid carnivoran mammals: A multiple regression approach from the skull and skeleton. Acta Palaeontol Pol. 2011;56: 225–246. doi:10.4202/app.2010.0005

7. Campione NE, Evans DC. A universal scaling relationship between body mass and proximal limb bone dimensions in quadrupedal terrestrial tetrapods. BMC Biol. 2012;10. doi:10.1186/1741-7007-10-60

8. Thomas Goodwin H, Bullock KM. Estimates of body mass for fossil giant ground squirrels, genus *Paenemarmota*. J Mammal. 2012;93: 1169–1177. doi:10.1644/11-MAMM-A-312.3

9. Head JJ, Gunnell GF, Holroyd PA, Howard Hutchison J, Ciochon RL. Giant lizards occupied herbivorous mammalian ecospace during the Paleogene greenhouse in Southeast

Asia. Proc R Soc B Biol Sci. 2013;280. doi:10.1098/rspb.2013.0665

10. Godoy PL, Benson RBJ, Bronzati M, Butler RJ. The multi-peak adaptive landscape of crocodylomorph body size evolution. BMC Evol Biol. 2019;19: 1–29. doi:10.1186/s12862-019-1466-4

11. Vidan E, Novosolov M, Bauer AM, Herrera FC, Chirio L, Campos Nogueira C, et al. The global biogeography of lizard functional groups. J Biogeogr. 2019;00: 1–12. doi:10.1111/jbi.13667

12. Pianka ER, Vitt LJ, Pelegrin N, Fitzgerald DB, Winemiller KO. Toward a Periodic Table of Niches, or Exploring the Lizard Niche Hypervolume. Am Nat. 2017;190: 000–000. doi:10.1086/693781

13. Pianka ER, Vitt LJ. Lizards: Windows to the Evolution of Diversity. Berkeley: University of California Press; 2003.

14. Vitt LJ, Pianka ER, Cooper WE, Schwenk K. History and the global ecology of squamate reptiles. Am Nat. 2003;162: 44–60. doi:10.1086/375172

15. Pough FH. The advantages of ectothermy for tetrapods. Am Nat. 1980;115: 92–112. doi:10.1086/283547

16. Muñoz MM, Bodensteiner BL. Janzen's Hypothesis meets the Bogert Effect: Connecting climate variation, thermoregulatory behavior, and rates of physiological evolution. Integr Org Biol. 2019;1: 1–12. doi:10.1093/iob/oby002

17. Muñoz MM, Losos JB. Thermoregulatory behavior simultaneously promotes and forestalls evolution in a tropical lizard. Am Nat. 2018;191: E15–E26. doi:10.1086/694779

18. Muñoz MM, Stimola MA, Algar AC, Conover A, Rodriguez AJ, Landestoy MA, et al. Evolutionary stasis and lability in thermal physiology in a group of tropical lizards. Proc R

Soc B Biol Sci. 2014;281: 20132433. doi:10.1098/rspb.2013.2433

19. Roll U, Feldman A, Novosolov M, Allison A, Bauer AM, Bernard R, et al. The global distribution of tetrapods reveals a need for targeted reptile conservation. Nat Ecol Evol. 2017;1: 1677–1682. doi:10.1038/s41559-017-0332-2

20. Vitt E.R. Pianka, W.E. Cooper, Jr., and K. Schwenk LJ. History and the global ecology of squamate reptiles. Am Nat. 2003;162: 44–60.

21. Herrera-Flores JA, Stubbs TL, Benton MJ. Ecomorphological diversification of squamates in the Cretaceous. R Soc Open Sci. 2021;8: 1–11. doi:10.1098/rsos.201961

22. Metzger KA, Herrel A. Correlations between lizard cranial shape and diet: A quantitative, phylogenetically informed analysis. Biol J Linn Soc. 2005;86: 433–466. doi:10.1111/j.1095-8312.2005.00546.x

23. Olalla-Tárraga MÁ, Rodríguez MÁ, Hawkins BA. Broad-scale patterns of body size in squamate reptiles of Europe and North America. J Biogeogr. 2006;33: 781–793. doi:10.1111/j.1365-2699.2006.01435.x

24. Meiri S. Length-weight allometries in lizards. J Zool. 2010;281: 218–226. doi:10.1111/j.1469-7998.2010.00696.x

25. Slavenko A, Feldman A, Allison A, Bauer AM, Böhm M, Chirio L, et al. Global patterns of body size evolution in squamate reptiles are not driven by climate. Glob Ecol Biogeogr. 2019;28: 471–483. doi:10.1111/geb.12868

26. Villa A, Delfino M. A comparative atlas of the skull osteology of European lizards (Reptilia: Squamata). Zool J Linn Soc. 2019; 1–100. doi:10.1093/zoolinnean/zlz035

27. Farlow JO, Hurlburt GR, Elsey RM, Britton ARC, Langston W. Femoral dimensions and body size of *Alligator mississippiensis*: Estimating the size of extinct mesoeucrocodylians.

J Vertebr Paleontol. 2005;25: 354–369. doi:10.1671/0272-4634(2005)025[0354:FDABSO]2.0.CO;2

28. O'Brien HD, Lynch LM, Vliet KA, Brueggen J, Erickson GM, Gignac PM. Crocodylian Head Width Allometry and Phylogenetic Prediction of Body Size in Extinct Crocodyliforms. Integr Org Biol. 2019;1. doi:10.1093/iob/obz006

29. Egi N. Body mass estimates in extinct mammals from limb bone dimensions: The case of North American Hyaenodontids. Palaeontology. 2001;44: 497–528. doi:10.1111/1475-4983.00189

30. Valkenburgh B Van. Skeletal and dental predictors of body mass in carnivores. Body size in Mammalian Paleobiology: Estimation and biological implications. 1990. pp. 181–205.

31. Bochaton C, Kemp ME. Reconstructing the body sizes of Quaternary lizards using *Pholidoscelis Fitzinger*, 1843, and Anolis Daudin, 1802, as case studies. J Vertebr Paleontol. 2017;37: 9 pp. doi:10.1080/02724634.2017.1239626

32. Sereno PC, Larsson HCE, Sidor CA, Gado B. The giant crocodyliform *Sarcosuchus* from the cretaceous of Africa. Science (80-). 2001;294: 1516–1519. doi:10.1126/science.1066521

33. Head JJ, Bloch JI, Hastings AK, Bourque JR, Cadena EA, Herrera FA, et al. Giant boid snake from the Palaeocene neotropics reveals hotter past equatorial temperatures. Nature. 2009;457: 715–718. doi:10.1038/nature07671

34. Herrera-Flores JA, Elsler A, Stubbs TL, Benton MJ. Slow and fast evolutionary rates in the history of lepidosaurs. Palaeontology. 2022;65: 1–13. doi:10.1111/pala.12579

35. Arnold EN. Evolutionary aspects of tail shedding in lizards and their relatives. J Nat Hist. 1984;18: 127–169. doi:10.1080/00222938400770131

36. Bateman PW, Fleming PA. To cut a long tail short: A review of lizard caudal autotomy studies carried out over the last 20 years. J Zool. 2009;277: 1–14. doi:10.1111/j.1469-7998.2008.00484.x

37. Gilbert EAB, Payne SL, Vickaryous MK. The anatomy and histology of caudal autotomy and regeneration in lizards. Physiol Biochem Zool. 2013;86: 631–644. doi:10.1086/673889

38. Sanggaard KW, Danielsen CC, Wogensen L, Vinding MS, Rydtoft LM, Mortensen MB, et al. Unique Structural Features Facilitate Lizard Tail Autotomy. PLoS One. 2012;7. doi:10.1371/journal.pone.0051803

39. Zani PA. Patterns of caudal-anatomy evolution in lizards. J Zool. 1996;240: 201–230.

40. Greer AE. Distribution of Maximum Snout-Vent Length among Species of Scincid Lizards. J Herpetol. 2001;35: 383–395.

41. Meiri S, Bauer AM, Chirio L, Colli GR, Das I, Doan TM, et al. Are lizards feeling the heat?: a tale of ecology and evolution under two temperatures. Glob Ecol Biogeogr. 2013;22: 834–845.

42. Feldman A, Sabath N, Pyron RA, Mayrose I, Meiri S. Body sizes and diversification rates of lizards, snakes, amphisbaenians and the tuatara. Glob Ecol Biogeogr. 2016;25: 187–197. doi:10.1111/geb.12398

43. Zachos J, Pagani M, Sloan L, Thomas E, Billups K. Trends, rhythms and aberrations in global climate 65 Ma to present. Science (80-). 2001;292: 686–693.

44. Zachos JC, Dickens GR, Zeebe RE. An early Cenozoic perspective on greenhouse warming and carbon-cycle dynamics. Nature. 2008;451: 279–283. doi:10.1038/nature06588

45. Woodburne MO, Gunnell GF, Stucky RK. Climate directly influences Eocene mammal faunal dynamics in North America. Proc Natl Acad Sci. 2009;106: 13399–13403. doi:10.1073/pnas.0906802106
46. Gunnell GF, Zonneveld JP, Bartels WS. Stratigraphy, mammalian paleontology, paleoecology, and age correlation of the Wasatch Formation, Fossil Butte National Monument, Wyoming. J Paleontol. 2016;90: 981–1011. doi:10.1017/jpa.2016.100
47. Saarinen JJ, Boyer AG, Brown JH, Costa DP, Ernest M, Evans AR, et al. Patterns of maximum body size evolution in Cenozoic land mammals: eco-evolutionary processes and abiotic forcing. Proc R Soc B Biol Sci. 2014;281: 1–10.
48. Rose KD. Composition and Species Diversity in Paleocene and Eocene Mammal Assemblages: An Empirical Study. J Vertebr Paleontol. 1981;1: 367–388.
49. Lovegrove BG, Mowoe MO. The evolution of mammal body sizes: Responses to Cenozoic climate change in North American mammals. J Evol Biol. 2013;26: 1317–1329. doi:10.1111/jeb.12138
50. Lyson TR, Miller IM, Bercovici AD, Weissenburger K, Fuentes AJ, Clyde WC, et al. Exceptional continental record of biotic recovery after the Cretaceous−Paleogene mass extinction. Science (80-). 2019;366: 977–983. doi:10.1126/science.aay2268
51. Alroy J. Constant extinction, constrained diversification, and uncoordinated stasis in North American mammals. Palaeogeogr Palaeoclimatol Palaeoecol. 1996;127: 285–311.
52. Alroy J. Cope's Rule and the Dynamics of Body Mass Evolution in North American Fossil Mammals. Science (80-). 1998;280: 731–734.
53. Alroy J. New Methods for Quantifying Macroevolutionary Patterns and Processes. Paleobiology. 2000;26: 707–733.

54. Alroy J. Speciation and extinction in the fossil record of North American mammals. In: Butlin RK, Brindle JR, Schluter D, editors. Speciation and Patterns of Diversity. Cambridge University Press; 2009. pp. 301–323.

55. Huang S, Eronen JT, Janis CM, Saarinen JJ, Silvestro D, Fritz SA. Mammal body size evolution in North America and Europe over 20 Myr: Similar trends generated by different processes. Proc R Soc B Biol Sci. 2017;284: 1–9. doi:10.1098/rspb.2016.2361

56. Smith KT, Gauthier JA. Early Eocene lizards of the Wasatch Formation near Bitter Creek, Wyoming: Diversity and paleoenvironment during an interval of global warming. Bull Peabody Museum Nat Hist. 2013;54: 135–230. doi:10.3374/014.054.0205

57. Smith KT. A diverse new assemblage of Late Eocene squamates (Reptilia) from the Chadron Formation of North Dakota, U.S.A. Palaeontol Electron. 2006;9: 1–44.

58. Smith KT. A new lizard assemblage from the earliest Eocene (zone Wa0) of the Bighorn Basin, Wyoming, USA: Biogeography during the warmest interval of the Cenozoic. Journal of Systematic Palaeontology. 2009. doi:10.1017/S1477201909002752

59. Smith KT. The evolution of mid-latitude faunas during the Eocene: Late Eocene lizards of the Medicine Pole Hills reconsidered. Bull Peabody Museum Nat Hist. 2011;52: 3–105.

60. Smith KT. The long-term history of dispersal among lizards in the early Eocene: New evidence from a microvertebrate assemblage in the Bighorn Basin of Wyoming, USA. Palaeontology. 2011;54: 1243–1270. doi:10.1111/j.1475-4983.2011.01107.x

61. Gauthier JA, Kearney M, Maisano JA, Rieppel O, Behlke ADB. Assembling the Squamate Tree of Life : Perspectives from the Phenotype and the Fossil Record Assembling the Squamate Tree of Life : Perspectives from the Phenotype and the Fossil Record. Bull Peabody Museum Nat Hist. 2012;53: 3–308. doi:10.3374/014.053.0101

62. Wiens JJ, Hutter CR, Mulcahy DG, Noonan BP, Townsend TM, Sites JW, et al. Resolving the phylogeny of lizards and snakes (Squamata) with extensive sampling of genes and species. Biol Lett Lett. 2012;8: 1043–1046. doi:10.5061/dryad.g1gd8
63. Woodburne MO, editor. Late Cretaceous and Cenozoic Mammals of North America: Biostratigraphy and Geochronology. New York: Columbia University Press; 2004.
64. Barnosky AD, Holmes M, Kirchholtes R, Lindsey E, Maguire KC, Poust AW, et al. Prelude to the Anthropocene: Two new North American Land Mammal Ages (NALMAs). Anthr Rev. 2014;1: 225–242. doi:10.1177/2053019614547433
65. Estes R, De Queiroz K, Gauthier J. Phylogenetic Relationships within Squamata. In: Estes R, Pregill G, editors. Phylogenetic Relationships of the Lizard Families. Palo Alto, CA: Stanford University Press; 1988. pp. 119–281.
66. Frost DR, Etheridge R. A phylogenetic analysis and taxonomy of iguanian lizards. Univ Kansas Museum Nat Hist Misc Publ. 1989;81: 1–65.
67. Smith RJ. Estimation of body mass in paleontology. J Hum Evol. 2002;43: 271–287. doi:10.1006/jhev.2002.0573
68. Hammer Ø, Harper DAT, Ryan PD. PAST: Palaeontological Statistics software package for education and data analysis. Palaeontol Electron. 2001;4: 1–9.
69. Gingerich PD. Arithmetic or geometric normality of biological variation: An empirical test of theory. J Theor Biol. 2000;204: 201–221. doi:10.1006/jtbi.2000.2008
70. Evans SE. The Skull of Lizards and Tuatara. Volume 20,. In: Gans C, Gaunt AS, Adler K, editors. The Skull of Lepidosauria. Volume 20,. Ithaca, NY: Society for the Study of Amphibians and Reptiles; 2008. pp. 1–348.
71. ElShafie SJ. Body size and species richness changes in Glyptosaurinae (Squamata:

Anguidae) through climatic transitions of the North American Cenozoic. Master's Thesis. Department of Earth & Atmospheric Sciences: University of Nebraska, Lincoln. University of Nebraska, Lincoln. 2014.

72. Meszoely CAM. North American fossil anguid lizards. Bull Museum Comp Zool. 1970;139: 87–150.

73. Gilmore CW. Fossil Lizards of North America. Mem Natl Acad Sci. 1928;22: v–201.

74. Sullivan RM. The taxonomy, chronostratigraphy and paleobiogeography of glyptosaurine lizards (Glyptosaurinae, Anguidae). Comptes Rendus Palevol. 2019;18: 747–763. doi:10.1016/j.crpv.2019.05.006

75. Scarpetta SG. *Peltosaurus granulosus* (Squamata, Anguidae) from the middle Oligocene of Sharps Corner, South Dakota, and the youngest known chronostratigraphic occurrence of Glyptosaurinae. J Vertebr Paleontol. 2019;39: e1622129. doi:10.1080/02724634.2019.1622129

76. Pianka ER. Evolution of body size: Varanid lizards as a model system. Am Nat. 1995;146: 398–414.

77. Makarieva AM, Gorshkov VG, Li BL. Gigantism, temperature and metabolic rate in terrestrial poikilotherms. Proc R Soc B Biol Sci. 2005;272: 2325–2328. doi:10.1098/rspb.2005.3223

78. Makarieva AM, Gorshkov VG, Li BL. Temperature-associated upper limits to body size in terrestrial poikilotherms. Oikos. 2005;111: 425–436. doi:10.1111/j.1600-0706.2005.14095.x

79. Pough FH. Lizard Energetics and Diet. Ecology. 1973;54: 837–844. doi:10.2307/1935678

80. Wing SL, Harrington GJ, Smith FA, Bloch JI, Boyer DM, Freeman KH. Transient floral

change and rapid global warming at the Paleocene-Eocene boundary. Science (80-). 2005;310: 993–996. doi:10.1126/science.1116913

81. Aureliano T, Ghilardi AM, Guilherme E, Souza-Filho JP, Cavalcanti M, Riff D. Morphometry, bite-force, and paleobiology of the Late Miocene caiman *Purussaurus brasiliensis*. PLoS One. 2015;10: 1–14. doi:10.1371/journal.pone.0117944

82. Molnar RE. Dragons in the dust: the paleobiology of the giant monitor lizard *Megalania*. Indiana University Press; 2004.

83. Conrad JL, Balcarcel AM, Mehling CM. Earliest Example of a Giant Monitor Lizard (*Varanus*, Varanidae, Squamata). PLoS One. 2012;7: e41767. doi:10.1371/journal.pone.0041767

84. Sinervo B, Méndez-de-la-Cruz F, Miles DB, Heulin B, Bastiaans E, Cruz MVS, et al. Erosion of lizard diversity by climate change and altered thermal niches. Science (80-). 2010;328: 894–899. doi:10.1126/science.1184695

85. Gregory KM, McIntosh WC. Paleoclimate and paleoelevation of the Oligocene Pitch-Pinnacle flora, Sawatch Range, Colorado. Geol Soc Am Bull. 1996;108: 545. doi:10.1130/0016-7606(1996)108<0545:PAPOTO>2.3.CO;2

86. Snell KE. Paleoclimate and paleoelevation of the western Cordillera in the United States. University of California, Santa Cruz. 2011.

87. Snell KE, Thrasher BL, Eiler JM, Koch PL, Sloan LC, Tabor NJ. Hot summers in the Bighorn Basin during the early Paleogene. Geology. 2013;41: 55–58. doi:10.1130/G33567.1

88. Huey RB, Slatkin M. Cost and benefits of lizard thermoregulation. Q Rev Biol. 1976;51: 363–384.

89. Wing SL. Eocene and Oligocene floras and vegetation of the Rocky Mountains. Ann Missouri Bot Gard. 1987;74: 748. doi:10.2307/2399449

90. Wing SL, Bown TM, Obradovich JD. Early Eocene biotic and climatic change in interior western North America. Geology. 1991;19: 1189–1192. doi:10.1130/0091-7613(1991)019<1189:EEBACC>2.3.CO;2

91. Stucky RK. Mammalian faunas in North America of Bridgerian to Early Arikareean "Ages" (Eocene and Oligocene). In: Prothero DR, Berggren WA, editors. Eocene-Oligocene Climatic and Biotic Evolution. Princeton, NJ: Princeton University Press; 1992. pp. 464–493.

92. Stamps JA. Rainfall, Moisture and Dry Season Growth Rates in *Anolis aeneus*. Copeia. 1977;1977: 415–419.

93. Wing SL, Greenwood DR. Fossils and fossil climate: The case for equable continental interiors in the Eocene. Philos Trans - R Soc London, B. 1993;341: 243–252. doi:10.1098/rstb.1993.0109

94. Fricke HC, Wing SL. Oxygen isotope and paleobotanical estimates of temperature and d18O-latitude gradients over North America during the early Eocene. Am J Sci. 2004;304: 612–635.

95. Huey RB. Behavioral thermoregulation in lizards: Importance of associated costs. Science (80-). 1974;184: 1001–1003.

96. Lafuma F, Corfe IJ, Clavel J, Di-Poï N. Multiple evolutionary origins and losses of tooth complexity in squamates. Nat Commun. 2021;12: 1–13. doi:10.1038/s41467-021-26285-w

97. Angilletta MJ, Steury TD, Sears MW. Temperature, growth rate, and body size in

ectotherms: Fitting pieces of a life-history puzzle. Integr Comp Biol. 2004;44: 498–509.

98. Kingsolver JG, Huey RB. Size, temperature, and fitness: Three rules. Evol Ecol Res. 2008;10: 251–268.

99. Beck DD. Biology of Gila Monsters and Beaded Lizards. 9th ed. Berkeley: University of California Press; 2005.

100. Angilletta, Jr. MJ, Niewiarowski PH, Dunham AE, Leaché AD, Porter WP. Bergmann's Clines in Ectotherms: Illustrating a Life- History Perspective with Sceloporine Lizards. Am Nat. 2004;164: E168–E183. doi:10.1086/425222

101. Woolley CH, Thompson JR, Wu Y-H (Beckey), Bottjer DJ, Smith ND. Can a fragmented past be trusted? Assessing bias and phylogenetic signal in the squamate fossil record. J Vertebr Paleontol Progr Abstr. 2020; 351–352.

102. Woolley CH, Thompson JR, Wu YH, Bottjer DJ, Smith ND. A biased fossil record can preserve reliable phylogenetic signal. Paleobiology. 2022; 1–16. doi:10.1017/pab.2021.45

103. Bell CJ, Gauthier JA, Bever GS. Covert biases, circularity, and apomorphies: A critical look at the North American Quaternary Herpetofaunal Stability Hypothesis. Quat Int. 2010;217: 30–36. doi:10.1016/j.quaint.2009.08.009

104. Ledesma DT, Scarpetta SG, Bell CJ. Variation in the skulls of *Elgaria* and *Gerrhonotus* (Anguidae, Gerrhonotinae) and implications for phylogenetics and fossil identification. PeerJ. 2021;9: 1–80. doi:10.7717/peerj.11602

105. Scarpetta SG, Ledesma DT, Bell CJ. A new extinct species of alligator lizard (Squamata: *Elgaria*) and an expanded perspective on the osteology and phylogeny of Gerrhonotinae. BMC Ecol Evol. 2021;21: 1–58. doi:10.1186/s12862-021-01912-8

106. The Paleobiology Database. Available: www.paleobiodb.org

107. Estes R. The fossil record and early distribution of lizards. Adv Herpetol Evol Biol. 1983; 365–398.

108. Nydam RL, Fitzpatrick BM. The occurrence of contogenys-like lizards in the late cretaceous and early tertiary of the western interior of the U.S.A. J Vertebr Paleontol. 2009;29: 677–701. doi:10.1671/039.029.0331

109. Estes R. A scincoid lizard from the Cretaceous and Paleocene of Montana. Cambridge, MA; 1969.

110. Bryant LJ. Non-dinosaurian lower vertebrates across the Cretaceous-Tertiary boundary in northeastern Montana. Vol. 134. Berkeley: University of California Press; 1989.

111. Conrad JL. An Eocene shinisaurid (Reptilia, Squamata) from Wyoming, U.S.A. J Vertebr Paleontol. 2006;26: 113–126. doi:10.1671/0272-4634(2006)26[113:AESRSF]2.0.CO;2

112. Tschopp E, Napoli JG, Wencker LCM, Delfino M, Upchurch P. How to Render Species Comparable Taxonomic Units Through Deep Time: A Case Study on Intraspecific Osteological Variability in Extant and Extinct Lacertid Lizards. Syst Biol. 2022;71: 875–900. doi:10.1093/sysbio/syab078

Supporting information

Materials & methods

Data collection

A~~I measured a~~lmost all the 283 fossil lizard specimens listed in S1 Dataset were measured in person in museum collections. A~~I obtained a~~ few measurements were obtained from literature or specimen photos provided by researchers (see “Measurements” below). The fossil dataset was likely biased toward adult individuals because complete anatomical elements are more likely to be preserved from adults. Juveniles are less likely to get preserved as fossils because their bones are more delicate and juveniles are frequently consumed by predators [100]. This preservation bias would not adversely affect the results in this study since it ~~I~~ focuses on maximum body sizes.

~~I also measured a total of~~The 266 extant specimens measured in this study (S2 Dataset) ~~from the crown lizard lineages represented in the fossil dataset. These~~ include~~d seven extant families (Anguidae, Varanidae, Xenosauridae, Shinisauridae, Xantusiidae, Teiidae, Scincidae) plus Iguania, with~~ 86 extant species total and 1-19 individuals sampled per species. ~~I was not able to sample every known species of every group; I only included the~~This dataset only includes extant species for which ~~I was able to find~~ reasonably complete dry skeletonized specimens ~~to measure~~were available. ~~I omitted~~ L~~l~~egless anguids and scincids were omitted because proportions of cranial elements to SVL differ between limbed taxa and serpentiform taxa in extant squamate groups [21] and because all the extinct taxa in ~~my~~ the fossil dataset presented here are inferred to have had limbs based on associated limb material or assignment to extant groups with limbs. ~~I amassed all~~ T~~t~~hese data for this study were amassed from 26 different

natural history museum collections across the United States (see “Institutional Abbreviations” in the main text).

When collecting extant data, I aimed for athe target sample size was of at least n = 20 for each taxonomic group whenever possible because this is considered acceptable for simple bivariate equations when *x* and *y* are highly correlated [65]. The only group for which I could not reach that sample size wasThe sample size was only smaller for Xenosauridae (n = 12; because there were not enough dry skeletonized specimens available. For that family, I had a usable sample size of only 12 (S3 Table), so results for this group should be considered with caution, so body size estimates for fossil xenosaurids in this study may be less reliable than for other taxa. However, the fossil specimens I sampled from that family were all relatively small (estimated SVL < 175 mm, S1 Dataset). Any inaccuracies for the fossil xenosaurid specimens within the margin of error would not change the observed patterns of maximum body size evolution for Paleogene lizards in the Western Interior.

Most described diagnostic characters for lizards are found in cranial bones [101,102]. Cranial bones account for most of the curated and published fossil record of lizards. This record is biased toward dentaries and maxillae, which are easy to identify as belonging to a lizard based on dentition and often provide at least coarse taxonomic identifications (family or higher) [102]. Therefore, we can be reasonably confident in taxonomic assignments for fossil lizard cranial bones based on extant phylogenetic morphological characters (although caution should be taken with historical identifications that may lack fidelity, especially at the genus or species level [103–105]).

I referred Ffossil specimens were referred to crown groups (family-level or higher) based on specimen label identifications as well as taxonomic information from the Paleobiology

Database [106] and current literature. For extinct taxa whose exact placement within or around an extant lizard group remains unresolved, ~~I assigned~~ the taxon was assigned to the closest crown group for the purpose of this study. This was the case for three extinct taxa: *Palaeoscincosaurus* and *Contogenys*, both referred to Scincidae [107–110], and *Provaranosaurus*, referred to Shinisauridae. The latter was based on Smith and Gauthier [56], who asserted that the extinct genus *Provaranosaurus* is closely related to extant *Shinisaurus* and placed it within a group called “*Pan-Shinisaurus*.” ~~I indicated~~ A~~a~~ny such considerations for specific taxa are indicated in the “Notes” column in S1 Dataset.

Measurements

All measurements were taken with digital calipers (Mitutoyo 150 mm) to the nearest 0.1 mm for individual bones and the nearest 1 mm for SVL. For specimens with an SVL measuring > 150 mm, or for specimens that were preserved in a curved position, ~~I used~~ a tape measure was used to measure SVL to the nearest 1 mm. ~~I only used~~Only extant specimens for which ~~I could measure~~ SVL could be measured were included, i.e., dry skeletonized specimens with a complete articulated vertebral series. ~~I also included t~~Two xenosaurid specimens with wet-preserved bodies and dry skeletonized skulls ~~-~~(S2 Dataset) were also included because fewer xenosaurid specimens were available and these two specimens still provided the ~~necessary~~ measurements needed. ~~I measured~~ SVL was measured on skeletons as the length from the ~~premaxilla (~~tip of the snout~~)~~ to the posterior centrum of the second caudal vertebra, which is the approximate position of the middle of the cloaca (vent; see Fig 1). ~~I determined~~ T~~the~~he position of the cloaca on skeletonized specimens was determined by aligning the pelvis of a wet specimen with that of a dry skeletonized congener specimen of comparable size. Whenever a dried skin was included

with a skeletonized specimen, it was used ~~I used it~~ to verify the SVL measurement from the vertebral series.

Skull length was measured as the length from the tip of the snout to the most posterior point of the skull (either the occipital condyle or the posterior tip of the supratemporal process). If a cranial bone had bilateral asymmetry (e.g., if on a parietal, one supratemporal process was longer than the other), the longer side was taken to represent the maximum measurement. ~~I measured~~ E~~e~~ach cranial bone was measured from the most anterior~~most~~ to the most posterior~~most~~ point (e.g., on the dentary, from the anterior tip of the ramus to the posterior tip of the retroarticular process). ~~If a cranial bone had bilateral asymmetry (e.g., if on a parietal, one supratemporal process was longer than the other), I took the longer side to represent the maximum measurement.~~ For long bones, ~~I only measured~~only complete bones with epiphyses included were measured.

When ~~I could not~~it was not possible to access a fossil specimen in person (which was the case for the UMMP specimens and a few specimens measured from figures in literature), ~~I took~~ measurements were taken from digital photographs with a scale bar in standard orientation using the open access software ImageJ (available online at: https://imagej.nih.gov/ij/download.html). Measurements taken from photographs are indicated in the "Notes" column in S1 Dataset. ~~I compared~~ M~~m~~easurements taken both in person and from photographs were compared for several extant specimens to ensure that ~~my~~ the measurements taken from photographs were accurate: all measurements ~~were~~ proved to be within a margin of error of 5% or less (1-3 mm).

~~Measurements of some extant specimens were taken on multiple occasions. In the first phase of data collection, I had not yet decided to measure individual cranial bones. I realized that I needed those measurements during the pandemic, when I could not access the same specimens~~

~~in person. I therefore had to supplement some extant cranial measurements from photos of those extant specimens that I had taken previously. Measurements taken from photographs are indicated in bold font in S2 Dataset. After the pandemic, I was able to reexamine and measure the extant specimens from the FMNH in person and confirmed the accuracy of those measurements as described above.~~

Generating and testing regressions

~~I only generated R~~Regressions were only generated for the anatomical elements represented in ~~my~~ the fossil dataset for each specific lizard group (S1-S7 Tables). For example, ~~I did not find any~~no isolated fossil varanid femora were measured, so ~~I did not generate~~ a regression for varanid femur length to SVL was not included (S2 Table).

~~I tested E~~Each regression was tested for homoskedasticity and~~, or homogenous scatter of residuals around the regression line [65]. To be considered acceptable, a regression had to have a statistically significant probability of homoskedasticity (p(homosked) ≥ 0.05). If a regression passed the homoskedasticity test, I then tested it~~ for normal distribution of residuals (p(homosked) and p(norm) ≥ 0.05~~, a requirement for RMA regressions~~) to validate the assumptions of the regression.~~ The R^2 values of the resulting regressions that passed both tests ranged from 0.69 to 0.98 (p(uncorr.) < 0.001; S1-S7 Tables).~~ These tests were ~~also~~ performed using PAST v.4.03 [68].

For each extant lizard group, ~~I selected~~ one specimen with a head length and SVL close to the average in ~~my~~ the extant dataset was selected for that group and remove~~d it~~ from the dataset prior to calculating the regressions. ~~I then used that~~ That removed specimen was then used to test the accuracy of each regression ~~that I~~ generated from the remaining sample of that extant lizard group (S1-S7 Tables). The ~~subsets~~ samples of extant specimens encompassed the

range of body size for most of the crown groups ~~that I~~ sampled except for Anguidae and Varanidae. The largest taxa in tho~~e~~se families were exceptionally large outliers (*Diploglossus millepunctatus* (Anguidae); *Varanus komodoensis* (Varanidae)). ~~I therefore~~Those outliers were therefore omitted ~~them~~ from the ~~subsets~~ samples used to generate regressions. However, ~~I applied~~ specimens of tho~~e~~se outliers were applied to their respective family regressions as an additional test. The resulting SVL differences were comparable (see Varanidae, S2 Table) or lower (see Anguidae, S1 Table) than the first test specimen, indicating that ~~both sets of~~ those regressions can reliably predict SVL for large outliers in tho~~e~~se families.

~~I also used~~A ~~a~~ large specimen of *Heloderma horridum* (Helodermatidae), the skull of which greatly resembles those of the largest anguid fossils in this dataset (Fig 3E), was used as an additional test specimen for the anguid regressions (S1 Table). Helodermatidae is placed close to Anguidae within the group Anguimorpha in both morphological and molecular phylogenetic analyses [61,62,65,66], so it is reasonable to consider Helodermatidae as an extant morphological and close phylogenetic analogue to large extinct anguid lizards. The anguid regressions proved comparably accurate in predicting the SVL of the largest helodermatid test specimen compared to the smaller extant and extinct anguid test specimens.

If a regression for a needed element failed the test for homoskedasticity or normal distribution of residuals, ~~I tried removing~~ outliers were removed from the dataset for that extant lizard group [67]. The outliers were no larger or smaller than any other taxa included, but their anatomical proportions differed from their congeners. ~~I then estimated~~ SVL was then estimated for the test specimens with and without the outliers removed. If the predicted values for SVL barely changed, ~~I proceeded to generate~~ regressions were generated for that group with the outliers removed. This was the case for groups such as Anguidae and Iguania. However, if the

new SVL estimate was very different, or if removing clear outliers still did not result in either regression passing the tests for both homoskedasticity and normal distribution of residuals, then ~~I did not proceed with that regression~~the regression was removed completely and fossil specimens that would have relied on that regression were omitted from the study. This resulted in the removal of a handful of fossil specimens known from a single element for which a suitable regression could not be generated. For example, ~~I had to omit~~ a few fossil varanid specimens known only from a frontal or parietal were omitted.

~~I calculated~~ M~~m~~inimum and maximum estimates of SVL (95% confidence interval) were calculated for each specimen by subtracting or adding the standard error, respectively, for the slope and interval of the regression used (S1 Dataset, S1-S7 Tables).

If a particular fossil specimen included more than one complete element, ~~I used~~ the regression that offered the lowest standard error of the estimate (SEE) was used. The regression equations in Table 3 and S1-S7 Tables are ranked in this order. For example, one fossil varanid specimen, FMNH PR 2380, included a complete dentary, maxilla, frontal, and parietal (S1 Dataset). The extant varanid dataset did not yield viable regressions for SVL based on frontal or parietal length, ~~so I could not use~~n ~~either~~either of those elements could be used to estimate SVL for ~~this~~ that specimen. The varanid regression for maxilla length to SVL yielded a lower SEE than that for dentary length to SVL (S2 Table), so ~~I used~~ the maxilla length regression was used to estimate SVL for ~~this~~ that specimen (S1 Dataset).

~~I did not generate~~ S~~s~~eparate regressions for males and females were not generated because most extant specimens ~~that I~~ measured did not have a sex indicated. Although female lizards often have shorter SVLs than males [4], there is no reliable way to tell the sex of an individual fossil lizard specimen based on isolated bones, so any fossil lizard dataset must be

treated as an averaged sample across sexes. The same is largely true ~~for fossils when it comes to determining the~~for ontogenetic stages ~~of a specimen~~represented in a fossil dataset. ~~Unless there are many specimens of differing sizes that are all referred to the same taxon, or if other osteological features related to maturation are present (e.g., terminal fusions of the braincase or mandible in crania or epiphyses on long bones; fusion of osteoderms to the dorsal skull [29,122]) it can be difficult to determine whether a particular specimen represents a juvenile, subadult, or adult of the extinct taxon to which it is referred.~~ Adult members of an extinct taxon can be reasonably identified if a large sample of referred individuals that reach a consistent maximum size range is present (e.g., large fossil anguids, S1 Dataset) or if some of those specimens include long bones with fused ~~or unfused~~ epiphyses, but some subadult or juvenile specimens may also be present. ~~The dataset presented here is robust enough to compensate for these uncertainties.~~

~~I did not generate regressions for every cranial or limb element for every taxonomic group sampled. I only generated the regressions that I needed to estimate SVL from the elements that I had in my fossil dataset (S1-S7 Tables). If you wish to estimate SVL for a fossil lizard specimen referred to a family included in this study, but the necessary anatomical element is not represented by the regressions offered here, you can generate the appropriate regressions from the included extant dataset (S2 Dataset) following the methods outlined in this paper (S2 Fig). I do not~~An attempt was made to ~~recommend~~ combine~~ing~~ measurements from different lizard groups to create "generic" lizard regressions to generate SVL estimates for unidentified lizard elements. However, none of the resulting regressions passed the necessary tests ~~because anatomical proportions vary between crown groups~~ (see the failed generic equations in S9~~8~~ Table). Anatomical proportions of the extant data sampled here varied too much between crown

groups ~~These methods only work for fossil lizard elements that can be referred to a specific crown group.~~ to produce successful generic regressions.

Special considerations for Iguania

Within extant Iguania, ~~I sampled~~ a representative range of the body size, morphology, and taxonomic diversity within pleurodontans was sampled because all the fossil iguanian specimens ~~I~~ measured had pleurodont dentition but not all were identified to a specific pleurodontan family. ~~The only acrodontan specimens I found with specific taxonomic identification were ascribed to *Tinosaurus*, but the phylogenetic affinity of this taxon remains dubious (it has been referred to both Chamaeleonidae [123] and Leiolepididae [124]), so I did not include those specimens in the dataset. I measured specimens from several large-bodied genera within Iguanidae (*Conolophus, Ctenosaura, Dipsosaurus, Iguana, Sauromalus*) to capture the upper range of iguanian body size. I also included some members of Phrynosomatidae (*Sceloporus, Urosaurus*) and Liolaemidae (*Liolaemus*) to account for small-bodied iguanians, and Corytophanidae (*Basiliscus*) to add morphological variety for mid-sized iguanians. I did not include~~ *Amblyrhynchus* ~~or~~ and *Crotaphytus* were not included because they were outliers with respect to head length : SVL ratio compared to the rest of the extant iguanian dataset (0.14 for *Amblyrhynchus* and 0.26 for *Crotaphytus* compared to an average of 0.21 for the other genera). ~~Note that the small-bodied iguanian *Urosaurus* also had a head length : SVL ratio of 0.26, but the regressions still passed tests for homoskedasticity and normal distribution of residuals with *Urosaurus* included. I therefore decided to leave *Urosaurus* in the dataset to capture a wider range of iguanian body sizes in those regressions.~~

Special considerations for Shinisauridae

~~I did not find any~~ No c~~c~~omplete dry skeletonized specimens of extant *Shinisaurus* were available to measure. Only ~~I only found~~ one dry skull and four wet preserved specimens of this monotypic family were found. ~~But~~ However, ~~I was able to measure~~ the holotype and only known complete skeleton of an extinct taxon referred to Shinisauridae, *Bahndwivici ammoskius* (FMNH PR 2260, Fig 5A) [111] was available. The *B. ammoskius* holotype skeleton had the same head length : SVL ratio as the average ~~that I measured~~calculated for extant *Shinisaurus* based on wet preserved specimens (0.23 vs. 0.22, n = 4). Therefore, ~~I used~~ ratios of individual cranial elements to SVL from the *Bahndwivici* skeleton were used to estimate SVL for the handful of fossil specimens referred to Shinisauridae (S1 Dataset).

Estimating body mass

The equation provided by Feldman et al. [42] was used for Shinisauridae because an equation for Shinisauridae was not provided and Shinisauridae falls within Anguimorpha. The equation for Fully Legged Anguidae was used from the same reference for the fossil anguid lizards because limb material has been found with many fossil anguid specimens and none have been interpreted as limbless. ~~Estimating body mass for fossils~~

Formatted: Font: 12 pt

Formatted: Indent: First line: 0.5"

~~Meiri [24] did not provide SVL-mass allometric equations for two families represented in this study, Xenosauridae and Shinisauridae. For these families, I used the general equation. I also used the general equation for almost all pleurotondan iguanian fossil specimens because most were not identified to a specific family within Iguania. Only two pleurodontan specimens were identified to a specific family, one to Corytophanidae (*Orithyia oaklandi*, PTRM 19271) and one to Polychrotidae (*Afairiguana avius*, FMNH PR 2379, Fig 5B). No equation was given for the~~

~~former, so the general equation was applied. Meiri did provide an equation for Polychrotidae, so I used that equation to estimate mass for *Afairiguana*.~~

~~Meiri's equation for Anguidae included legged as well as leg-reduced and legless species. Although Meiri provided equations for legged species only for other families, they did not do this for Anguidae, presumably due to lack of sufficient specimens (n = 4). The general mass estimates for anguids were all 49% greater than the anguid equation estimates. The difference was only 1-5% for all other families. Therefore, I listed mass estimates from both the anguid and general equations for anguid specimens in Table 3.~~

Formatted: Indent: First line: 0.5"

D~~Results & d~~iscussion

Taxonomic ~~turnover~~richness

No more than two distinct species were documented for any given genus in the fossil dataset (S1 Dataset). Many fossil specimens could not be identified with certainty past the level of genus (e.g., *Melanosaurus sp.*). There is no way to know exactly how many different species might be represented by these specimens, even if only one diagnostic species is recognized within a given extinct genus (e.g., *Melanosaurus maximus*). Many of the extinct genera in this study that included only one diagnostic species almost certainly included other unidentified species as well. For example, the extinct anguid genus *Melanosaurus* had greater variation in SVL than any individual extant anguid species (standard deviation = 222 vs. 34 ~~at most~~ (*Gerrhonotus liocephalus*); S1 and S2 Datasets). In general, it is much more difficult to discern diagnostic species within a genus than it is to parse genera within a clade based on fossils [112]. In this fossil dataset, 88% of specimens were identified to the level of genus but only 42% were

referred to a species. This may account for some of the discrepancies between the data reported here and the data available in the Paleobiology Database (Table 2).

However, this is unlikely to affect the patterns of taxonomic richness observed here. Even in extant lizards, elevated richness usually reflects a high number of genera with a low number of species per genus, possibly because lizard genera within families are more ecologically distinct from each other than are species within genera [3]. Thus, in lieu of trying to estimate the actual number of extinct species sampled, tracking richness at the level of genera and higher taxonomic groups (S3~~2~~ Fig) is likely to capture a sufficient representation of the taxonomic diversity in this geotemporal system.

~~Even with this advantage, this dataset does not reflect the full diversity or geographic distribution of that diversity within this system. This study only presents body size estimates for fossil lizard specimens that included a complete cranial or limb element (or in rare cases, a complete skeleton). There were many other specimens available that contained only fragmentary material. For example, this study did not recover the majority of described pleurodontan taxa from the Paleogene Western Interior as a result. For a thorough census of Paleogene lizard diversity in the Western Interior, see the work of Krister Smith [54–58].~~

Supporting Captions

S1 Fig. Protocol for estimating snout-vent length of fossil lizards.

S2~~1~~ Fig. Regressions of dentary length to snout-vent length for all extant lizard groups sampled. Shinisauridae is not included here because no regressions were generated for that group (see “Special Considerations for Shinisauridae” in ~~Supplementary Information~~Supporting

Information). See Table 3 for regression equations and statistics. All regressions are natural log transformed using natural log.

Formatted: Widow/Orphan control, Adjust space between Latin and Asian text, Adjust space between Asian text and numbers

S2 Fig. Protocol for estimating body size of fossil lizards. This is the procedure that I recommend for estimating body length of fossil lizard specimens. Additional notes: **1)** Be sure to measure all cranial and limb element lengths in addition to SVL. An ideal sample size will include 20 or more individuals, but even $n \geq 10$ can be useful. **2)** Ideally, the extant test specimen(s) will represent species for which you have multiple individuals sampled. Make sure the test specimen(s) has all measurements available, including limb measurements if any of the fossil specimens from that taxonomic group include limb elements. **3)** Both log and natural log are commonly used in this type of study. **4)** Use reduced major axis linear regressions to account for error in both the dependent and independent variable measurements. **5)** Check the regression for outliers and remove them as needed for the regression to pass both tests. If removing outliers does not work, the regression cannot be used, and the corresponding fossil specimens should be removed from the dataset. Note that if extant specimens are not available for a particular group, and a complete fossil skeleton from that group is included in the dataset, anatomical ratios from that skeleton can be used to estimate body length for incomplete fossil specimens from the same taxonomic group. This approach should be used as a last option because simple ratios do not capture allometric effects. **6)** Calculate the percentage difference between the predicted and actual SVL for each test specimen. Check to see if the actual SVL falls within or near the 95% confidence interval of the estimate. Rank the regressions in order of increasing standard error of the estimate (SEE). **7)** If a specimen includes more than one element, choose the element regression with the lowest SEE.

S3 Fig. Rarefaction curve showing the number of expected crown taxonomic groups for NALMA sample sizes within the fossil lizard dataset. S1 Dataset was subsampled here by North American Land Mammal Age (NALMA) interval. NALMA abbreviations: Pu = Puercan, To = Torrejonian, Ti = Tiffanian, Cf = Clarkforkian, Wa = Wasatchian, Br = Bridgerian, Ui = Uintan, Du = Duchesnean, Ch = Chadronian, Or = Orellan, Wh = Whitneyan, Ar = Arikareean. Individual rarefaction analysis was performed using PAST v4.03 [68].

S4~~3~~ Fig. Rarefaction curve showing the number of expected size groups for NALMA sample sizes within the fossil lizard dataset. To conduct this analysis, ~~I assigned~~ each fossil specimen in S1 Dataset was assigned to one of 11 size groups, each group representing a bin of 100 mm (e.g., 1-99 mm, 100-199 mm, 200-299 mm, etc.; the last group was ≥ 1 m). S1 Dataset wa~~i~~s subsampled here by North American Land Mammal Age (NALMA) interval. The y-axis in this graph shows the total number of size groups recovered in the sample for each NALMA interval. NALMA abbreviations: Pu = Puercan, To = Torrejonian, Ti = Tiffanian, Cf = Clarkforkian, Wa = Wasatchian, Br = Bridgerian, Ui = Uintan, Du = Duchesnean, Ch = Chadronian, Or = Orellan, Wh = Whitneyan, Ar = Arikareean. Individual rarefaction analysis was performed using PAST v4.03 [68].

~~**S4 Fig. Rarefaction curve showing the number of expected crown taxonomic groups for NALMA sample sizes within the fossil lizard dataset.** S1 Dataset is subsampled here by North American Land Mammal Age (NALMA) interval. NALMA abbreviations: Pu = Puercan, To = Torrejonian, Ti = Tiffanian, Cf = Clarkforkian, Wa = Wasatchian, Br = Bridgerian, Ui = Uintan,~~

~~Du = Duchesnean, Ch = Chadronian, Or = Orellan, Wh = Whitneyan, Ar = Arikareean. Individual rarefaction analysis was performed using PAST v4.03 [66].~~

~~**S5 Fig. Maximum SVL in large-bodied vs. small-bodied lizards by NALMA. A)** Maximum SVL in Anguidae vs. Xantusiidae. Function: LN(Xantusiidae Max SVL) = ((-0.236±0.132)*LN(Anguidae Max SVL)) + (5.96±0.865), R^2 = 0.37, p(uncorr.) = 0.39, SEE = 0.230. **B)** Maximum SVL in Anguidae vs. Xenosauridae. Function: LN(Xenosauridae Max SVL) = ((-0.723±0.452)*LN(Anguidae Max SVL)) + (9.09±2.84), R^2 = 0.22, p(uncorr.) = 0.53, SEE = 0.605. Anguidae includes most of the largest body sizes estimated for fossil lizards in this study and Xantusiidae and Xenosauridae include some of the smallest. Only four paired NALMA data points were available for each correlation. All data were transformed using natural log. Regression analysis was performed using PAST v4.03 [68].~~

S5~~6~~ Fig. Global temperature vs. lizard body size by NALMA. A) Global marine mean annual paleotemperature (MAPT) vs. maximum lizard SVL. Function: LN(Global Marine MAPT) = ((1.23±0.401)*LN(Max Lizard SVL)) + (3.70±0.792), R^2 = 0.04, p(uncorr.) = 0.56, SEE = 0.991. **B)** Global marine MAPT vs. mean lizard SVL. LN(Global Marine MAPT) = ((-0.910±0.303)*LN(Mean Lizard SVL)) + (7.06±0.606), R^2 = 0.0004, p(uncorr.) = 0.95, SEE = 0.812. Temperature data are from Zachos et al. [44] and were taken from the NALMA midpoint in ºC. All data were transformed using natural log. Regression analysis was performed using PAST v4.03 [68].

Formatted: Widow/Orphan control, Adjust space between Latin and Asian text, Adjust space between Asian text and numbers

S6 Fig. Mean and maximum snout-vent length in fossil lizards by taxonomic group per NALMA. Dotted line = maximum. Dashed line = mean. Lines do not connect with the single Duchesnean datapoint since it likely does not accurately represent a maximum or mean value for that NALMA. North American Land Mammal Age (NALMA) abbreviations: Pu = Puercan, To = Torrejonian, Ti = Tiffanian, Cf = Clarkforkian, Wa = Wasatchian, Br = Bridgerian, Ui = Uintan, Du = Duchesnean, Ch = Chadronian, Or = Orellan, Wh = Whitneyan, Ar = Arikareean. All measurements were transformed using natural log.

Formatted: Font: Bold

S7 Fig. Mean and maximum body mass in fossil lizards by taxonomic group per NALMA. Dotted line = maximum. Dashed line = mean. Lines do not connect with the single Duchesnean datapoint since it likely does not accurately represent a maximum or mean value for that NALMA. North American Land Mammal Age (NALMA) abbreviations: Pu = Puercan, To = Torrejonian, Ti = Tiffanian, Cf = Clarkforkian, Wa = Wasatchian, Br = Bridgerian, Ui = Uintan, Du = Duchesnean, Ch = Chadronian, Or = Orellan, Wh = Whitneyan, Ar = Arikareean. All measurements were transformed using natural log.

S1 Table. Anguidae regression equations and tests. Sample sizes indicate number of extant anguid specimens that included the element used in the given regression. Regression equations are ranked in order of increasing standard error of the estimate (SEE). Minimum and maximum SVL estimates for test specimens are based on the standard errors for the slope and intercept of the given regression. Equations for body mass from individual bones are based on the relevant equation listed in S8 Table.

S2 Table. Varanidae regression equations and tests. Sample sizes indicate number of extant varanid specimens that included the element used in the given regression. Regression equations are ranked in order of increasing standard error of the estimate (SEE). Minimum and maximum SVL estimates for test specimens are based on the standard errors for the slope and intercept of the given regression. Equations for body mass from individual bones are based on the relevant equation listed in S8 Table.

S3 Table. Xenosauridae regression equations and tests. Sample sizes indicate number of extant xenosaurid specimens that included the element used in the given regression. Regression equations are ranked in order of increasing standard error of the estimate (SEE). Minimum and maximum SVL estimates for test specimens are based on the standard errors for the slope and intercept of the given regression. Equations for body mass from individual bones are based on the relevant equation listed in S8 Table.

S4 Table. Teiidae regression equations and tests. Sample sizes indicate number of extant teiid specimens that included the element used in the given regression. Regression equations are ranked in order of increasing standard error of the estimate (SEE). Minimum and maximum SVL estimates for test specimens are based on the standard errors for the slope and intercept of the given regression. Equations for body mass from individual bones are based on the relevant equation listed in S8 Table.

S5 Table. Scincidae regression equations and tests. Sample sizes indicate number of extant scincid specimens that included the element used in the given regression. Regression equations

are ranked in order of increasing standard error of the estimate (SEE). Minimum and maximum SVL estimates for test specimens are based on the standard errors for the slope and intercept of the given regression. Equations for body mass from individual bones are based on the relevant equation listed in S8 Table.

S6 Table. Xantusiidae regression equations and tests. Sample sizes indicate number of extant xantusiid specimens that included the element used in the given regression. Regression equations are ranked in order of increasing standard error of the estimate (SEE). Minimum and maximum SVL estimates for test specimens are based on the standard errors for the slope and intercept of the given regression. Equations for body mass from individual bones are based on the relevant equation listed in S8 Table.

S7 Table. Iguania regression equations and tests. Sample sizes indicate number of extant iguanian specimens that included the element used in the given regression. Regression equations are ranked in order of increasing standard error of the estimate (SEE). Minimum and maximum SVL estimates for test specimens are based on the standard errors for the slope and intercept of the given regression. Equations for body mass from individual bones are based on the relevant equation listed in S8 Table.

S8 Table. Published equations for estimating lizard body mass from snout-vent length. These equations were used to estimate mass for fossil lizards as listed in S1 Dataset and to generate equations for estimating body mass from individual bones (S1-S7 Tables).

S9~~8~~ Table. Tests of general regression equations. Sample sizes indicate number of extant ~~helodermatid~~ specimens that included the element used in the given regression. Regression equations are ranked in order of increasing standard error of the estimate (SEE). None of these equations passed tests for both homoskedasticity and normal distribution of residuals, thus, none of these equations were used in this study.

S10~~9~~ Table. Data for correlating SVL with temperature. Temperature data ~~are~~ came from Zachos et al. [44] and were taken from the NALMA midpoint in ºC. The Duchesnean wa~~i~~s not included because the maximum SVL estimate for that NALMA was exceptionally low and based on a single data point.

S11 Table. Mean and maximum fossil lizard snout-vent length and body mass by taxonomic group per NALMA. These natural log-transformed data were used to generate the graphs in S6 Fig and S7 Fig.

Formatted: Font: Bold

S1 Dataset. Fossil lizard data. Measurements, locality information, and body size estimates for all fossil lizard specimens sampled. Family-specific mass was calculated from equations listed in S8 Table [24,41,42]. General mass estimate (across all lizard groups) was calculated using the embedded equation from Meiri [24].

S2 Dataset. Extant lizard data. Data for all extant lizard specimens measured. Measurements taken from photographs using ImageJ are indicated in bold font.

Formatted: Widow/Orphan control, Adjust space between Latin and Asian text, Adjust space between Asian text and numbers

February 28, 2023

Dear PLoS One Editorial Board,

Thank you for the opportunity to make these additional revisions to my manuscript. Below is a list of the new changes that I made.

Overall Changes Made:

- I revised the whole manuscript to be in third-person passive voice to make it sound more impersonal.
- I separated the Results and Discussion sections and substantially revised both sections based on the Editor and Reviewer comments, including omitting the section on Competition from the Discussion.

Body Mass Estimations and Equations

- I agree that adding equations for estimating mass of fossil specimens from individual bone measurements is useful. However, I cannot generate novel equations for body mass from individual lengths here because almost none of the extant lizard specimens that I measured included measurements of body mass. Unfortunately, lizard specimens that are dry skeletonized almost never have the original weight recorded before they are processed. Furthermore, measurements of individual cranial and limb bones are not provided in Feldman et al. (2016) or Meiri (2010), both of which were recommended by Reviewer 2 as sources for extant lizard measurements and equations for lizard body mass from SVL. Therefore, I do not have the data necessary to generate novel equations for estimating body mass from individual cranial or limb bones.
  - Instead, I translated the original equations that I provided here for estimating SVL from individual bones (Tables S1-S7) into the equations for estimating mass from SVL provided in Feldman et al. (2016; see S8 Table). The resulting equations have been added to Tables S1-S7. **These translated equations can now be used to estimate lizard body mass from individual cranial and limb bones.**
  - The mass estimates are the same whether you calculate mass from SVL using the equations from Feldman et al. (2016) or the new equations for body mass from individual elements provided here.
- I am still including the original equations I generated for estimating SVL from individual bones because SVL is also an important variable for paleontologists and zoologists.
- Calculating body mass equations for individual fossil species would not be practical because fossil specimens usually cannot be identified to individual species. In addition, even genus-level assignments often change for fossil specimens upon later reexamination.
  - It is safer and more practical to develop family-level equations - that way, the body mass estimate becomes resilient to any specific taxonomic identification changes. Furthermore, family-level equations are based on a larger sample size.

This approach is consistent with the rest of the paper, and with the sources used (Meiri 2010; Meiri et al. 2013; Feldman et al. 2016), which focus on family-level analyses.

New Figures and Tables

- I added the following figures:
    - **Fig 7.** Body mass distribution by taxonomic group for fossil lizards in the Western Interior of North America through the Paleogene, including a line for the overall mean lizard body mass per NALMA.
    - **S6 Fig.** Mean and maximum snout-vent length in fossil lizards by taxonomic group per NALMA.
    - **S7 Fig.** Mean and maximum body mass in fossil lizards by taxonomic group per NALMA.
        - I added commentary about the latter two new figures in the revised Discussion as well.
- I also added a line for the overall mean lizard snout-vent length per NALMA to the SVL distribution graph in Fig 6.
- I added the following tables:
    - **S8 Table.** Published equations for estimating lizard body mass from snout-vent length.
    - **S11 Table.** Mean and maximum fossil lizard snout-vent length and body mass by taxonomic group per NALMA.
- The numbers of all other figures and tables were also updated accordingly.

I made all changes suggested by the Editor and Reviewers, with the following notes…

Additional Editor Comments:

- *Table 2 is a bit broad and I think it would be better a figure showing specimens and /or species sampled by group / time period in comparison with diversity expected as by the palaeodb database*.
    - I still prefer these data presented as a table, and I think it's helpful to have the NALMAs listed here in table format with their age ranges. But I took the suggestion of adding the number of genera and species sampled per taxonomic group per NALMA compared to the numbers expected based on the Paleobiology Database (PBDB). These data have been added to the table. I think the result is more a reflection of how much is missing from the PBDB, but the data are now provided for comparison.
- *Table 3 should be simplified showing slope as parameter (b), confidence interval and intercept + R2 and SEE.*
    - I made these changes, but for the sake of space and simplicity, I listed the standard error value rather than listing the full confidence interval (e.g., - X.XXX to X.XXX).

Commented [SE1]: Add columns to Table 2 for # fossil specimens and # fossil species sampled per family per NALMA. Also add the number of expected species for each family per NALMA based on PBDB. Note in the Table 2 caption that PBDB diversity is provided for comparison, but should be taken as a coarse estimate because there may be some identification error in museum labels as well as PBDB entries.

- *Line 264-269: ok but perhaps this might be due to some outliers or some groups whose body bauplan differ from the "general" lizard one. What if you try exclusion of some groups?*
    - I did try excluding some groups, and the attempted general regressions still did not pass the tests for homoskedasticity and normal distribution of residuals. In any case, now that body mass estimation equations specific to taxonomic group are used in this study, I think a generalized lizard SVL equation is unnecessary.
- *Reduce extensively also the part on sex (line 936-948). You did not have sex to test so all you are saying is quite speculative.*
    - I think the comment made here is an important point to make for paleontological studies, and Reviewer 1 agreed. However, I did shorten this paragraph.

Reviewer 2 Comments:

- *I made a mistake recommending Meiri 2010 in my last review for body mass estimates. I meant Feldman 2016 (which Meiri is on) supplementary appendix 2 (see below). It has all of the regression equations needed for your study including the families missing at the moment. There is an equation for legged anuids and legless anguids. If I'm not mistaken there are a few species that you are not sure if they had limbs or not? In which case you might want to use the Anguimorpha equation, or simply assume that they had limbs if there are no known limbless glyptosaurs. In any case you definitely need to change this.*
    - I used updated equations from Feldman et al. (2016). The equations listed in this reference were only different for Anguidae, Xenosauridae, Iguania, and Anguimorpha; the rest were taken from Meiri (2010), so I didn't need to change those. I used the Anguimorpha equation for Shinisauridae since an equation for Shinisauridae was not provided and Shinisauridae falls within Anguimorpha. I used the equation for Fully Legged Anguidae for the fossil anguid lizards because limb material has been found with many fossil anguid specimens – none have been interpreted as limbless.
- *27 – you say "large dataset" of fossil lizards but I think that you should be specific in saying what percent of Midwest species you studied. You can check this on the paleobiology database I think.*
    - I honestly don't know what Reviewer 2 meant by this comment. Only some of the fossil lizards in my dataset were collected in the Midwest, and I don't know why that matters in the context of this study. If they meant that I should specify how many of the taxa in the fossil dataset still occur in the Western Interior today, I point that out in the Results section (see "Taxonomic Turnover"). I don't think the Introduction is the place for that detail.
- *414-421 – really interesting analysis, you should use family as an additional variable in addition to doing it across all species. Also, how did you try to correlate them? Linear regression model? ANOVA based on each NALMA? What was the predictor and what was the response? This remains very unclear in the text. You should provide a quantitative analysis, perhaps similar to Smith 2018. Or at the very least, a very in-depth qualitative analysis/discussion. Quantitative is always better, so I would recommend that. I would personally recommend using linear regressions with estimated body size of*

Commented [SE2]: Find these equations in Feldman et al 2016 Supp Appendix 2.

species as the response, and temperature per million years as the predictor (check where smith got the data, I think Zachos something). You can also use family as an additional covariate.

- I revised the text to clarify how I set up this regression. However, the additional analyses suggested here were not feasible because the predictor variable (lizard body size) could not be binned per million years due to the way in which the specimens were documented in museum collections (the majority of the specimens are only referred to a NALMA interval and cannot be referred to a more precise time bin). Furthermore, only two lizard families (Anguidae and Varanidae) represent the maximum lizard body size for any given NALMA, and the record of Varanidae is not continuous enough through the Paleogene to set up a correlation (see S6-S7 Fig). The correlation is already mostly a correlation between maximum anguid body size and varanid body size.

Thank you again for the considerable time you, the Editor, and the Reviewers have contributed to reviewing this paper, and for your patience as I incorporated the suggestions into the manuscript. I look forward to hearing back from you.

Best regards,

Sara J. ElShafie, Ph.D.
Alumnus, Department of Integrative Biology and Museum of Paleontology
University of California, Berkeley
selshafie@berkeley.edu
