# Supplementary material for: Body size estimation from isolated fossil bones reveals deep time evolutionary trends in North American lizards
Source: PLoS One. 2024 Jan 5;19(1):e0296318. doi: 10.1371/journal.pone.0296318 (PMC10769094; doi:10.1371/journal.pone.0296318)
Supplement: Attachment — Submitted filename: PONE-D-22-04244_R3_reviewer.pdf [file pone.0296318.s029.pdf]

PLOS ONE

Body size estimation from isolated fossil bones reveals deep time evolutionary trends in North American lizards

--Manuscript Draft--

**Manuscript Number:**	PONE-D-22-04244R3
**Article Type:**	Research Article
**Full Title:**	Body size estimation from isolated fossil bones reveals deep time evolutionary trends in North American lizards
**Short Title:**	Body size estimation from isolated fossil bones reveals deep time evolutionary trends for lizards
**Corresponding Author:**	Sara J ElShafie, PhD<br>UC Berkeley: University of California Berkeley<br>Berkeley, CA UNITED STATES
**Keywords:**	body size; reptiles; lizards; vertebrate paleobiology; size regressions; fossils
**Abstract:**	Lizards play vital roles in extant ecosystems. However, their roles in extinct ecosystems are poorly understood because the fossil record of lizards consists mostly of isolated bones. This makes it difficult to document changes in lizard morphology and body size over time, which is essential for studies of lizard paleoecology and evolution. It is also difficult to compare available fossil lizard data with existing sources of extant lizard data because extant studies rarely measure individual bones. Furthermore, no previous study has regressed measurements of individual bones to body length across crown lizard groups, nor tested those regressions on fossil skeletons. An extensive dataset of individual bone measurements from extant lizards across crown taxonomic groups is here employed to develop novel methods for estimating lizard body size from isolated fossil elements. These methods were applied to a comparably large dataset of fossil lizard specimens from the robust Paleogene record (66-23 Ma) of the Western Interior of North America. This study tests the hypothesis that anatomical proportions have been conserved within higher-level crown lizard groups since the Paleogene and can therefore be used to reconstruct snout-vent length (SVL) for fossil specimens referred to the same groups.<br>Individual bones allowed for predicting SVL of fossil lizard specimens with strong accuracy. The resulting body size estimates for the entire fossil dataset revealed that lizards reached greatest maximum body size in the middle Paleogene, with the largest size class dominated by anguid lizards that exceeded 1 meter in SVL and 1 kg in body mass. Maximum body size decreased to under 400 mm in the late Paleogene. This is the first study to investigate body size evolution across lizard clades over a deep time interval and for a large geographic region. The proposed methods can be used to generate body size regressions and provide estimates of SVL for isolated lizard bones referred to any crown group.
**Order of Authors:**	Sara J ElShafie, PhD
**Response to Reviewers:**	Dear PLoS One Editorial Board,<br><br>Thank you for the opportunity to make these additional revisions to my manuscript. Below is a list of the new changes that I made.<br><br><br>Overall Changes Made:<br><br>•I revised the Introduction to make it more concise.<br>•I heavily reworked the Materials and Methods section to be sufficiently detailed and more cohesive.<br>•I took the text that had been moved to Supporting Information, distilled it, and incorporated it back into the main text.<br>•I added mean values to Table 4 to provide the full data visualized in Fig 6, Fig 7, S6 Fig, and S7 Fig. I also revised the captions for those figures.<br>•I removed all material related specifically to taxonomic turnover. This included removal of the former Table 2 and S3 Fig, which were no longer relevant.

Powered by Editorial Manager® and ProduXion Manager® from Aries Systems Corporation

I made all changes suggested by the Editor and Reviewers, with the following minor exceptions:

Editor comments and edits (lines refer to previous version of the manuscript)

•Line 18: With respect to the Editor's comment, I did not indicate that lizards are more vital to extant ecosystems than pollinating insects or any other group, only they that they are important. I elaborate on this with citations in the Introduction. I maintain that this is a reasonable and appropriate opening sentence for this abstract.

•Lines 32-35: I like the more concise phrasing suggested here. However, the femur was only possible to regress for Anguidae, not the other lizard groups, so I omitted that mention.

•Lines 159-160: I think the justification for the chosen study system is best placed in the Introduction, and does not need to be reiterated in the Materials & Methods section.

•Line 370 – "do you mean correlation r????": I mean the coefficient of determination for the correlation, R2. I think this sentence was confusing as written, so I revised it.

•Lines 395-406: With respect, I do not think that this information is off topic. It helps to explain why we might be seeing the observed patterns even though the maximum body sizes to not correlate with global temperature data. I left this information in the "Climate as a potential driver of body size evolution" section of the Discussion, but I did revise that section to be more cohesive.

•The section Appendix should be part of supporting material [Appendix S1] and needs to be more in line with PlosOne style and nomenclature (https://journals.plos.org/plosone/s/supporting-information).
oI am not sure what the Editor meant by this comment because there was no section in the manuscript labeled "Appendix." If they were referring to the supporting datasets (S1 and S2 Datasets), I see no reason to change the file name because "Dataset" is listed as one of the common item descriptions in the Supporting Information guidelines link provided by the Editor. Those files are datasets, and I would prefer to designate them as such for clarity. Furthermore, I did not see any instructions in the linked guidelines as to a required format for dataset files. If the Editor would like to request specific changes to the formatting of S1 and S2 Datasets with explanations for the needed changes, I would be happy to make those changes.

Reviewer #3 comments and edits (lines refer to previous version of the manuscript)

•Lines 234-238: Log was not written out as "logarithm" in the PLoS One publication that the Editor recommended as a formatting reference (Field et al. 2013), and "log" is the more common term used, so I did not see the need to write it out here.

•Lines 267-272: I agree that the explanation of how I derived new equations for estimating body mass was not very clear as written. I revised it to be more coherent. However, I decided to stick with the existing arrangement of explaining the equations that had already been published and then explaining how I derived new equations because I had to use the existing published equations to generate the new equations.

•Line 419: Correct, mass cannot be zero, but the mass listed here is 1kg, with a natural log of 0.00.

•I would suggest starting the data collection section by saying the total number of collected specimens and the number of genera they represent.
oI understand what the reviewer is getting at – it would be confusing to only discuss the fossil data but not the extant data before presenting a table that offers both fossil and extant data. However, this is just an artefact of the formatting for the submitted manuscript. Since the tables will be separated from the text in the final layout, this will no longer be an issue. I think the text flows better with the details for the fossil data

Powered by Editorial Manager® and ProduXion Manager® from Aries Systems Corporation

	presented together in one paragraph, followed by the details for the extant data in the next paragraph. But I also revised the entire Data Collection section to improve its clarity.<br><br>•Rows 227-229: I suggest putting this sentence in line with rows 232-235 sayings that although bivariate regression is frequently used, you chose to utilise RMA instead, and then discuss the variables you employed.<br>oRMA is a form of bivariate linear regression, not an alternative to it. I added the word “bivariate” after RMA to clarify this for anyone who is not familiar with RMA regressions.<br><br>•It is not very clear from the main manuscript that the fossil dataset includes also juveniles, in my opinion.<br>oThere was a brief mention of this in the Methods, and the SI included commentary on why juveniles were not included in the extant dataset and why they were likely not represented in the fossil dataset. I revised that commentary and moved it back to the Methods section in the main text.<br><br><br><br>Thank you again for the additional time that you, the Editor, and the Reviewers have contributed to reviewing this paper, and for your patience as I incorporated the suggestions into the manuscript. I look forward to hearing back from you.
**Additional Information:**	
**Question**	**Response**
**Financial Disclosure**<br><br>Enter a financial disclosure statement that describes the sources of funding for the work included in this submission. Review the submission guidelines for detailed requirements. View published research articles from *PLOS ONE* for specific examples.<br><br>This statement is required for submission and **will appear in the published article** if the submission is accepted. Please make sure it is accurate.	This work was supported by the Anthony Barnosky Graduate Student Research Fund (UCMP – ucmp.berkeley.edu), the Doris O. and Samuel P. Welles Research Fund (UCMP), the Department of Integrative Biology at the University of California Berkeley (ib.berkeley.edu), the Mary R. Dawson Award and the Albert E. Wood Award (Society for Vertebrate Paleontology – vertpaleo.org), the Charles A. and June R.P. Ross Research Award (Geological Society of America – www.geosociety.org), the Evolving Earth Foundation Graduate Student Research Grant (www.evolvingearth.org), the Burke Museum Vertebrate Paleontology Collections Study Grant (www.burkemuseum.org), the Sigma Xi Grant in Aid of Research (www.sigmaxi.org), the Friends of the Nebraska State Museum (friendsofthemuseum.org), the Nebraska Geological Society (www.nebraskageologicalsociety.org), the Department of Earth and Atmospheric Sciences at the University of Nebraska Lincoln (eas.unl.edu), the Sakana Foundation, and the Uplands Foundation. The funders had no role in study design, data collection and analysis, decision to publish, or preparation of the manuscript.

**Unfunded studies**<br>Enter: *The author(s) received no specific funding for this work.*<br><br>**Funded studies**<br>Enter a statement with the following details:<br>• Initials of the authors who received each award<br>• Grant numbers awarded to each author<br>• The full name of each funder<br>• URL of each funder website<br>• Did the sponsors or funders play any role in the study design, data collection and analysis, decision to publish, or preparation of the manuscript?<br>• **NO** - Include this sentence at the end of your statement: *The funders had no role in study design, data collection and analysis, decision to publish, or preparation of the manuscript.*<br>• **YES** - Specify the role(s) played.<br><br>* typeset	
**Competing Interests**<br><br>Use the instructions below to enter a competing interest statement for this submission. On behalf of all authors, disclose any competing interests that could be perceived to bias this work—acknowledging all financial support and any other relevant financial or non-financial competing interests.<br><br>This statement is required for submission and **will appear in the published article** if the submission is accepted. Please make sure it is accurate and that any funding sources listed in your Funding Information later in the submission form are also declared in your Financial Disclosure statement.<br><br>View published research articles from *PLOS ONE* for specific examples.	The author has declared that no competing interests exist.

Powered by Editorial Manager® and ProduXion Manager® from Aries Systems Corporation

**NO authors have competing interests**<br><br>Enter: *The authors have declared that no competing interests exist.*<br><br>**Authors with competing interests**<br><br>Enter competing interest details beginning with this statement:<br><br>*I have read the journal's policy and the authors of this manuscript have the following competing interests: [insert competing interests here]*<br><br>* typeset	
**Ethics Statement**<br><br>Enter an ethics statement for this submission. This statement is required if the study involved:<br><br>• Human participants<br>• Human specimens or tissue<br>• Vertebrate animals or cephalopods<br>• Vertebrate embryos or tissues<br>• Field research<br><br>Write "N/A" if the submission does not require an ethics statement.<br><br>General guidance is provided below. Consult the submission guidelines for detailed instructions. **Make sure that all information entered here is included in the Methods section of the manuscript.**	N/A

**Format for specific study types**<br><br>**Human Subject Research (involving human participants and/or tissue)**<br>• Give the name of the institutional review board or ethics committee that approved the study<br>• Include the approval number and/or a statement indicating approval of this research<br>• Indicate the form of consent obtained (written/oral) or the reason that consent was not obtained (e.g. the data were analyzed anonymously)<br><br>**Animal Research (involving vertebrate animals, embryos or tissues)**<br>• Provide the name of the Institutional Animal Care and Use Committee (IACUC) or other relevant ethics board that reviewed the study protocol, and indicate whether they approved this research or granted a formal waiver of ethical approval<br>• Include an approval number if one was obtained<br>• If the study involved *non-human primates*, add *additional details* about animal welfare and steps taken to ameliorate suffering<br>• If anesthesia, euthanasia, or any kind of animal sacrifice is part of the study, include briefly which substances and/or methods were applied<br><br>**Field Research**<br><br>Include the following details if this study involves the collection of plant, animal, or other materials from a natural setting:<br>• Field permit number<br>• Name of the institution or relevant body that granted permission	
**Data Availability**<br><br>Authors are required to make all data underlying the findings described fully available, without restriction, and from the time of publication. PLOS allows rare exceptions to address legal and ethical concerns. See the PLOS Data Policy and FAQ for detailed information.	Yes - all data are fully available without restriction

A Data Availability Statement describing where the data can be found is required at submission. Your answers to this question constitute the Data Availability Statement and **will be published in the article**, if accepted.<br><br>**Important:** Stating 'data available on request from the author' is not sufficient. If your data are only available upon request, select 'No' for the first question and explain your exceptional situation in the text box.<br><br>Do the authors confirm that all data underlying the findings described in their manuscript are fully available without restriction?	
**Describe where the data may be found in full sentences. If you are copying our sample text, replace any instances of XXX with the appropriate details.**<br><br>• If the data are **held or will be held in a public repository**, include URLs, accession numbers or DOIs. If this information will only be available after acceptance, indicate this by ticking the box below. For example: *All XXX files are available from the XXX database (accession number(s) XXX, XXX.*).<br>• If the data are all contained **within the manuscript and/or Supporting Information files**, enter the following: *All relevant data are within the manuscript and its Supporting Information files.*<br>• If neither of these applies but you are able to provide **details of access elsewhere**, with or without limitations, please do so. For example:<br><br>*Data cannot be shared publicly because of [XXX]. Data are available from the XXX Institutional Data Access / Ethics Committee (contact via XXX) for researchers who meet the criteria for access to confidential data.*<br><br>*The data underlying the results presented in the study are available from (include the name of the third party*	All relevant data are within the manuscript and its Supporting Information files.

and contact information or URL).<br>• This text is appropriate if the data are owned by a third party and authors do not have permission to share the data.<br><br>* typeset	
Additional data availability information:	

Powered by Editorial Manager® and ProduXion Manager® from Aries Systems Corporation

Manuscript
Click here to access/download;Manuscript;Manuscript.docx

Body size estimation from isolated fossil bones reveals deep time evolutionary trends in North American lizards

Sara J. ElShafie

Department of Integrative Biology and Museum of Paleontology, University of California, Berkeley, California, United States of America

E-mail: selshafie@berkeley.edu

Abstract

Lizards play vital roles in extant ecosystems. However, their roles in extinct ecosystems are poorly understood because the fossil record of lizards consists mostly of isolated bones. This makes it difficult to document changes in lizard morphology and body size over time, which is essential for studies of lizard paleoecology and evolution. It is also difficult to compare available fossil lizard data with existing sources of extant lizard data because extant studies rarely measure individual bones. Furthermore, no previous study has regressed measurements of individual bones to body length across crown lizard groups, nor tested those regressions on fossil skeletons. An extensive dataset of individual bone measurements from extant lizards across crown taxonomic groups is here employed to develop novel methods for estimating lizard body size from isolated fossil elements. These methods were applied to a comparably large dataset of fossil lizard specimens from the robust Paleogene record (66-23 Ma) of the Western Interior of North America. This study tests the hypothesis that anatomical proportions have been conserved within higher-level crown lizard groups since the Paleogene and can therefore be used to reconstruct snout-vent length (SVL) for fossil specimens referred to the same groups.

Individual bones allowed for predicting SVL of fossil lizard specimens with strong accuracy. The resulting body size estimates for the entire fossil dataset revealed that lizards reached greatest maximum body size in the middle Paleogene, with the largest size class dominated by anguid lizards that exceeded 1 meter in SVL and 1 kg in body mass. Maximum body size decreased to under 400 mm in the late Paleogene. This is the first study to investigate body size evolution across lizard clades over a deep time interval and for a large geographic region. The proposed methods can be used to generate body size regressions and provide estimates of SVL for isolated lizard bones referred to any crown group.

Introduction

Body size influences every aspect of a vertebrate's physiology and life history [1–4]. It is therefore essential to estimate body size for fossil vertebrate taxa in order to understand the ecological and evolutionary context of any vertebrate group [5–10]. Lizards are a particularly important group of vertebrates to investigate in both extant and extinct ecosystems because they exhibit high taxonomic and morphological diversity and occupy a wide range of ecological roles [4,11–14]. For example, lizards contribute significant tetrapod species richness in some environments, especially deserts; can maintain high population densities in small areas; and have high dispersal abilities [15–19]. Lizards also regulate populations of insects, rodents, and other prey on which they feed, and they are an important food source for many animals [4,12,13,20].

The lizard fossil record consists mostly of isolated elements that can be difficult to identify to the level of genus or even family. Therefore, body size for fossil lizards must be inferred primarily from isolated bones [21]. To further complicate matters, anatomical datasets of extant lizards rarely include measurements of individual cranial or limb bones for comparison. Most relevant previous studies only recorded measurements of head length and snout-vent length (SVL) across extant lizard groups [3,4,11,12,19,22–26]. Hence, no prior study has reconstructed body size in lizards on evolutionary time scales across higher-level taxonomic groups. This study presents an extensive reference dataset of individual anatomical element measurements taken across several crown group lizard lineages and develops methods for using those data to estimate body size in fossil lizards.

It is not unambiguously clear which variable is the best proxy for body size in fossil vertebrates. Some previous studies estimated mass as a proxy for body size in fossil

crocodyliforms using scaling relationships in extant taxa between body mass and femoral length, femoral circumference [27], and head width [28], since these anatomical measurements are commonly available in the crocodyliform fossil record. Body mass has also been estimated for dinosaurs [7] and fossil mammals [6,29,30] using similar methods. However, body mass is only weakly correlated with most individual bones in many fossil vertebrates; thus, such methods can be difficult to apply across a fossil record of varied isolated bones [6].

For fossil reptiles, body length is easier to estimate from individual bones than body mass because length correlates tightly with other anatomical measurements. Body length has been shown to correlate with femur length ([27]), vertebral element width ([31]), head length ([32]), head width ([28]), and specific cranial bones such as dentary length [9,33] in extant lizards, snakes, and crocodylians ($R^2 \geq 0.78$ in all cited examples). Mandible length has also been used as a direct proxy for body size in fossil lizards [21,34].

SVL, measured from the tip of the snout to the cloacal opening at the base of the tail (Fig 1), is often the preferred measure of body length in extant lizard studies [22,33]. This is because many lizards can autotomize and even regenerate their tails [35–39], thus lizard tail measurements are highly variable. Total body length is also difficult to reconstruct in lizards because the tail does not always possesses its original length in an extant specimen and is often disarticulated in dry skeletonized specimens. Measurements of SVL are more feasible to obtain from wet preserved or dry skeletonized extant lizard specimens. Maximum SVL strongly correlates with mean adult SVL and SVL at sexual maturity in extant lizards [3,40], so maximum SVL is a good metric of mean adult body size for a population of lizards sampled from the fossil record. Furthermore, once SVL is estimated, lizard body mass can be calculated from family-specific SVL-to-mass equations [24,41,42].

Fig 1. Anatomical measurements. Measurements are indicated by arrows. Snout-vent length (SVL) was measured from the tip of the snout at the premaxilla to the posterior edge of the centrum on the second caudal vertebra, which is the approximate position of the middle of the cloaca (vent). Other measurement acronyms: DL = dentary length, FeL = femur length, FrL = frontal length, HL = head length, HuL = humerus length, MnL = mandible length, MxL = maxilla length, PaL = parietal length, TiL = tibia length. Scale bar = 1 cm. Specimen pictured is *Gerrhonotus infernalis* (Anguidae; FMNH 22452).

For this study, lizards were sampled from the Paleogene record (66-23 Ma) in the Western Interior of North America, specifically, from localities concentrated in the United States (Fig 2). The Paleogene is an interesting period to study because it spans significant warming and cooling events [43,44] and chronicles the radiations of both mammals [45–55] and later squamates [56–60] following the End-Cretaceous Mass Extinction. This timeframe was used here as a study system because it has a prolonged and robust fossil record in the Western Interior, preserving many diverse and ecologically important extant lizard clades that still occur in the area. These include anguids and pleurodontan iguanians (e.g., iguanids and dactyloids), as well as clades that ~~were extirpated~~ from the region during the Paleogene, such as varanids and shinisaurids. All the sampled Paleogene specimens, many identified to the level of genus or even species, were stored in natural history museum collections in the United States.

Fig 2. Map showing all localities for fossil lizard data from across the Western Interior of North America through the Paleogene. Featured area is highlighted in inset map. Data points

represent only specimens for which the locality could be georeferenced. Some taxonomic groups occurring with others in a particular locality may not be visible. Base maps were made with Natural Earth and are reprinted from Natural Earth under a CC Public Domain license, original copyright 2009. All other data contained were collected by the author. Maps were generated using QGIS-LTR 3.22.6 (download.qgis.org). The figure created is under the CC-BY 4.0 license, as determined by the authors of QGIS.

This study tested the hypothesis that proportions of individual bone lengths to SVL are conserved within each of eight crown group lizard lineages (exclusive of snakes) represented in the Paleogene fossil record of the Western Interior, and that these proportions can therefore be used to predict SVLs for fossil lizard specimens. For each crown group investigated, individual cranial and limb bones were regressed onto SVL, and the resulting equations were validated using complete skeletons of both extant and fossil lizards. The equations were then applied to reconstruct body size for crown group lizards sampled from across the U.S. Western Interior through the Paleogene. This study provides the first opportunity to investigate modes of body size evolution across lizard diversity over a prolonged geologic interval and spanning a continental interior.

Institutional abbreviations

AMNH, American Museum of Natural History, New York, NY; **CAS**, California Academy of Sciences, San Francisco, CA; **CJB**, Christopher J. Bell, University of Texas at Austin, Austin, TX; **CM**, Carnegie Museum of Natural History, Pittsburgh, PA; **DMNH**, Denver Museum of Natural History (Denver Museum of Nature and Science), Denver, CO; **FLMNH**,

Florida Museum of Natural History, Gainesville, FL; **FMNH**, Field Museum of Natural History, Chicago, IL; **FOBU**, Fossil Butte National Monument, Kemmerer, WY; **GCVP**, Georgia College Vertebrate Paleontology, Milledgeville, GA; **LACM**, Natural History Museum of Los Angeles County, Los Angeles, CA; **MCZ**, Museum of Comparative Zoology, Cambridge, MA; **MSU**, Michigan State University, Lansing, MI; **MVZ**, Museum of Vertebrate Zoology, Berkeley, CA; **NMMNH**, New Mexico Museum of Natural History, Albuquerque, NM; **PTRM**, Pioneer Trails Regional Museum, Bowman, ND; **RAM**, Raymond M. Alfe Museum of Paleontology, Claremont, CA; **SMM**, Science Museum of Minnesota, Saint Paul, MN; **TxVP**, Texas Vertebrate Paleontology Collection, The University of Texas, Austin, TX; **UCM**, University of Colorado Museum, Boulder, CO; **UCMP**, University of California Museum of Paleontology, Berkeley, CA; **UMMP**, University of Michigan Museum of Paleontology, Ann Arbor, MI; **UNSM**, University of Nebraska State Museum, Lincoln, NE; **USNM**, United States National Museum (Smithsonian National Museum of Natural History), Washington, DC; **UW**, University of Wyoming Geological Museum, Laramie, WY; **UWBM**, University of Washington Burke Museum, Seattle, WA; **YPM**, Yale Peabody Museum, New Haven, CT.

Materials & methods

Data collection

The data for this study were ~~amassed~~ from 26 different natural history museum collections across the United States (see "Institutional Abbreviations"). These include anatomical measurements collected from a total of 283 fossil lizard specimens, with 44 recognized genera and at least 33 recognized species, from the Paleogene record of the U.S. Western Interior (Table 1, S1 Dataset). Only fossil lizard specimens with complete cranial bones or associated limb

bones were sampled (e.g., Fig 3), for the following reasons: 1) the lizard fossil record is mostly comprised of cranial bones and, fortunately, most extant phylogenetic morphological characters for lizards are found in cranial bones [61,62], making it possible to determine at least coarse taxonomic identifications (family or higher) for fossil lizard specimens from cranial material [62]; 2) historical fossil identification may lack fidelity at the genus or species level [63–65]; 3) cranial and limb bones can be used to estimate SVL in lizards [9,33]. A few measured specimens included complete skulls (Fig 3A) or even skeletons (Figs 4 and 5). All fossil specimens were referred to crown groups (family-level or higher) based on specimen label identifications as well as taxonomic information from the Paleobiology Database [66] and current literature.

Table 1. Summary of datasets. The extant species totals include subspecies. Fossil species totals do not include specimens that could not be identified to a specific genus or species (e.g., Anguidae indet.). For example, all three fossil teiid specimens sampled were identified as only “Teiidae indet.”

Taxonomic group	# Fossil specimens sampled	# Fossil genera sampled	# Fossil species sampled	# Extant specimens sampled	# Extant genera sampled	# Extant species sampled
**Anguidae**	218	16	17	66	4	17
**Varanidae**	6	1	1	47	1	17
**Teiidae**	3	0	0	33	5	11
**Scincidae**	4	3	1	28	8	19
**Xantusiidae**	19	2	2	20	1	3
**Xenosauridae**	7	4	3	17	1	3

**Iguania**	19	8	6	55	9	16
**Shinisauridae**	7	3	3	N/A	N/A	N/A
**Total**	**283**	**37**	**33**	**266**	**29**	**86**

Fig 3. Paleogene anguimorph lizards from the Western Interior of North America and extant morphological analogues. (A) *Peltosaurus granulosus*† (Anguidae) skull in right lateral view, and B) right humerus in medial view (AMNH 42913), indicated by arrow, Orellan (33.9-31.8 Ma). (C) *Gerrhonotus infernalis* (Anguidae) skull and right mandible in right lateral view and (D) left humerus in medial view (FMNH 22452), extant. A-D are oriented with anterior to the right. (E) *Heloderma suspectum* (Helodermatidae) skull and left mandible in left lateral view, and (F) right humerus in medial view (UCMP 117512), extant. (G) *Helodermoides tuberculatus*† skull, mandibles, and cervical osteoderms in right lateral view, reflected for continuity (USNM V 13869), Chadronian (37.0-33.9 Ma). (H) *Helodermoides tuberculatus*† (Anguidae) left mandible in lateral view, and (I) right humerus in medial view, reflected for continuity (UNSM 4511), Chadronian. E-I are oriented with anterior to the left. Scale bar = 1 cm. USNM V 13869 (G) image courtesy of the Smithsonian Institution.

Fig 4. Paleogene varanids from the Western Interior of North America. (A) *Saniwa ensidens*† skeleton (FMNH PR 2378), Wasatchian (54.9-50.5 Ma). Scale bar = 10 cm. (B) *Saniwa sp.*† dentaries, vertebrae, and fragments (DMNH EPV.34588), Bridgerian (50.5-46.2 Ma). Scale bar = 3 cm.

Fig 5. Other complete Paleogene lizard skeletons from the Western Interior of North America. (A) *Bahndwivici ammoskius*† (Shinisauridae) skeleton (FMNH PR 2260), Wasatchian (54.9-50.5 Ma). Scale bar = 3 cm. (B) *Afairiguana avius*† (Iguania; Polychrotidae) skeleton (FMNH PR 2379), Wasatchian. Scale bar = 3 cm.

To compare the fossil lizard specimens with extant congeners, 266 extant specimens were also measured (S2 Dataset), comprising 30 genera and 86 species (Table 1) from the same crown group lineages represented in the fossil dataset. These included seven extant families: Anguidae (e.g., alligator lizards and galliwasps), Scincidae (skinks), Shinisauridae (crocodile lizards), Teiidae (e.g., whip-tail lizards), Varanidae (monitor lizards), Xantusiidae (night lizards), and Xenosauridae (knob-scaled lizards). Legless anguids and scincids were omitted because proportions of cranial elements to SVL differ between limbed taxa and serpentiform taxa in extant squamate groups [21] and because all the extinct taxa in the fossil dataset presented here are inferred to have had limbs based on associated limb material or assignment to extant groups with limbs. The pleurodontan iguanian families represented in the fossil dataset (e.g., Iguanidae and Polychrotidae (Fig 5B)) were treated as a collective group, "Iguania," in both datasets. Phylogenetic relationships among iguanian families are poorly resolved, but the group "Iguania" is supported by both morphological and molecular analyses [67–70]. In order to compare individual cranial and limb measurements with SVL measurements, extant data collection in this study focused on dry skeletonized specimens with reasonably complete skulls and vertebral series (Fig 1, S2 Dataset). A total sample size of at least n = 17 was collected for each of the eight major taxonomic groups (it was difficult to find complete specimens of Xenosauridae, so that sample size was the lowest), with 1-21 individuals sampled per species.

Extant species were selected for sampling to encompass the full range of extant body size diversity within a given lineage as much as possible, with the exception of extreme outliers. However, some outlier specimens were used to further test the predictive power of regressions for groups that included exceptionally large individuals in the fossil dataset, Anguidae and Varanidae.

All measurements were taken with digital calipers (Mitutoyo 150 mm) to the nearest 0.1 mm for individual bones and the nearest 1 mm for SVL. For specimens with an SVL measuring > 150 mm, or for specimens that were preserved in a curved position, a tape measure was used to measure SVL to the nearest 1 mm. SVL was measured on skeletons as the length from the tip of the snout to the posterior centrum of the second caudal vertebra, which is the approximate position of the middle of the cloaca (vent; see Fig 1). The position of the cloaca on skeletonized specimens was determined by aligning the pelvis of a wet specimen with that of a dry skeletonized congener specimen of comparable size. Whenever a dried skin was included with a skeletonized specimen, it was used to verify the SVL measurement from the vertebral series. Skull length was measured as the length from the tip of the snout to the most posterior point of the skull (either the occipital condyle or the posterior tip of the supratemporal process; Fig 1). If a cranial bone had bilateral asymmetry (e.g., if on a parietal, one supratemporal process was longer than the other), the longer side was taken to represent the maximum measurement. Each cranial bone was measured from the most anterior to the most posterior point (e.g., on the dentary, from the anterior tip of the ramus to the posterior tip of the retroarticular process). For long bones, only complete bones with epiphyses included were measured.

When it was not possible to access a fossil specimen in person (which was the case for the UMMP specimens and a few specimens measured from figures in literature), measurements

were taken from digital photographs with a scale bar in standard orientation using the open access software ImageJ (available online at: https://imagej.nih.gov/ij/download.html). Measurements taken from photographs are indicated in the "Notes" column in S1 Dataset.

No permits were required for the described study, which complied with all relevant regulations.

Generation and testing of regressions for body length

This study regressed anatomical elements (the independent variables) – including head length, individual cranial bone length (dentary, mandible, maxilla, frontal, parietal), and limb bone length (humerus, femur, tibia) – to SVL for each extant lizard group in the dataset presented (S1 Fig, S1-S7 Tables). Regressions were only generated for the anatomical elements available for each lizard group in the fossil dataset (S1-S7 Tables). For example, no complete and isolated fossil varanid femora were found in collections, so a regression for varanid femur length to SVL was not generated (S2 Table).

Reduced major axis (RMA) bivariate linear regressions were used because they accommodate error in both the dependent and independent variable measurements [71]. Before the analyses, all the measurements were transformed using the natural log (LN). This method is commonly applied in analyses of fossil vertebrate body size, especially to minimize the effects that any outliers might have on regression coefficients [7,9,29,49,52,53,72–75]. Tests of natural log compared to base 10 log regressions also produced the same SVL predictions for large test specimens in this study, so natural log was used in all regressions for consistency. All regression analyses were performed using PAST v.4.03 [71].

To test the assumption that anatomical proportions are conserved between extant and extinct members within any particular lizard lineage represented in this study, the SVL values predicted from individual bones were compared with those measured from complete skeletons of extant taxa from the same lineage and, when available, fossil congeners as well (S1-S7 Tables). The fossil test specimens included three of the few available complete fossil lizard skeletons that were measured: a large-bodied varanid (*Saniwa ensidens,* FMNH PR 2378, SVL 425 mm which is smaller than the largest extant varanid measured in this study (*V. exanthematicus*, 770 mm; Fig 4A), a small-bodied anguid (*cf. Parophisaurus pawneensis,* YPM VP 060609, SVL 138 mm), and a very small-bodied iguanian (*Afairiguana avius*, FMNH PR 2379, SVL 46 mm; Fig 5B). For each SVL regression tested, the 95% confidence interval for the SVL estimate of each test specimen was calculated by adding or subtracting the standard error of the regression (S1-S7 Tables).

No complete dry skeletonized specimens of extant *Shinisaurus* were available to measure. Only one dry skull and four wet preserved specimens of this monotypic family were found in collections. Fortuitously, however, the holotype and only known complete skeleton of an extinct taxon referred to Shinisauridae, *Bahndwivici ammoskius* (FMNH PR 2260, SVL 154 mm; Fig 5A) [76] had the same head length-: SVL ratio as the average calculated for extant *Shinisaurus* based on wet preserved specimens (0.23 vs. 0.22, n = 4; S2 Dataset). Therefore, ratios of individual cranial elements to SVL from the *Bahndwivici* skeleton were used to estimate SVL for the handful of fossil specimens referred to Shinisauridae (S1 Dataset).

Estimations and new equations for body mass

It has been shown that body mass scales linearly with SVL in extant lizards with limbs [15,24]. This relationship allowed researchers to previously develop allometric equations useful for estimating lizard body mass based on SVL at the family level (S8 Table) [24,41,42] as well as a general SVL-to-mass equation for all lizards (RMA: Body Mass in grams = (((3.088±0.067)*SVL) – (4.852±0.128))/1000, R^2 = 0.946) [24]. Body mass for each fossil specimen in the dataset presented here (S1 Dataset) was calculated using the published family-level equations (S8 Table), as well as the published general RMA equation for comparison.

This study also generated new equations for estimating lizard body mass directly from individual elements (S1-S7 Tables). This was done by substituting an equation from this study for estimating SVL from a specific element for a given family (e.g., head length-to-SVL for Anguidae, S1 Table) into the published equation for estimating lizard body mass from SVL for that same family (S8 Table), producing a new equation for calculating mass directly from that specific element (e.g., head length-to-mass for Anguidae, S1 Table). Thus, when applied to the fossil lizard specimens in S1 Dataset, the new element-to-mass equations produced the same body mass estimates as those obtained from the previously published equations (S1 Dataset, S8 Table).

Analysis of body size evolution

Maximum and mean SVL and mass were used as proxies for understanding patterns of lizard body size evolution through the Paleogene in the Western Interior of North America. The novel equations generated in this study for estimating body length and the published equations referenced in S8 Table for calculating body mass were applied to all fossil lizard specimens in S1 Dataset. When estimating body length or mass for a fossil specimen that included more than one

complete cranial or limb element, the corresponding regression that offered the lowest SEE was used (S1-S7 Tables). All analyses were conducted at the level of crown-group assignments [63–65]. Fossil data were grouped by North American Land Mammal Ages (NALMAs), which compose a relative chronology used to divide the Paleogene based on taxonomic turnover in the mammal fossil record [77,78].

Rarefaction was used to analyze sample sizes though the Paleogene in this study system. S1 Dataset was subsampled by NALMA interval to determine the expected size range per NALMA based on sample size. Each fossil specimen in S1 Dataset was assigned to one of 11 size groups for SVL, each group representing a bin of 100 mm (e.g., 1-99 mm, 100-199 mm, 200-299 mm, etc.; the last group was SVL ≥ 1 m). Individual rarefaction analysis was performed using PAST v4.03 [71].

Results

Regression equations for lizard body size

Results investigating the relationships between different skeletal elements and body size across the lizard crown groups represented in this study indicated that the most consistently reliable cranial measurements for estimating SVL were head length (standard error of the estimate (SEE) ≤ 0.0997, Anguidae and Iguania) and maxilla length (SEE ≤ 0.1331, Anguidae, Varanidae, Scincidae, Xantusiidae, Iguania; Fig 6, Table 2, S1-S7 Tables). Regressions based on limb measurements (femur, humerus, or tibia length) also had strong predictive power for Anguidae and Varanidae, the only two groups with fossil limb material available in the fossil dataset (SEE ≤ 0.0815). Greater variation in rankings was observed among lizard groups for regressions based on dentary, frontal, and parietal length (SEE 0.0530 – 0.1571). General

equations based on the entire extant dataset (S2 Dataset) proved unsuccessful because none passed tests for both homoskedasticity and normal distribution of residuals (S9 Table).

Table 2. Regression functions and associated statistics for snout-vent length estimation from individual bones. All functions were transformed using the natural log (LN). Standard error for slope and intercept terms are listed after the slope and intercept in the equations. Functions are listed in order of increasing standard error of the estimate (SEE) for each group. If a particular fossil specimen included more than one complete cranial or limb measurement, the applicable regression that offered the lowest SEE was used. P(uncorrelated) < 0.001 for all functions.

**Group**	**Predictor**	***n***	**Slope *b***	**Standard error of *b***	**Intercept**	**Standard error of intercept**	R^2	**SEE**
Anguidae	Skull Length	46	0.946	0.0417	1.71	0.129	0.91	0.0740
	Femur Length	43	0.888	0.0410	2.46	0.101	0.91	0.0742
	Mandible Length	43	0.921	0.0424	1.82	0.129	0.91	0.0760
	Humerus Length	41	0.889	0.0451	2.57	0.106	0.90	0.0815
	Parietal Length	24	1.03	0.0658	2.29	0.148	0.91	0.0853
	Maxilla Length	22	1.02	0.0803	2.40	0.170	0.88	0.0992
	Frontal Length	22	1.27	0.111	2.13	0.216	0.85	0.120
	Dentary Length	24	0.898	0.0816	2.43	0.195	0.82	0.121
Varanidae	Tibia Length	27	0.953	0.0282	2.43	0.103	0.98	0.0695
	Maxilla Length	25	1.13	0.0423	2.04	0.145	0.97	0.0837
	Dentary Length	23	1.13	0.0845	1.86	0.305	0.88	0.157
Xe	Dentary Length	12	0.36	0.164	0.921	0.450	0.85	0.0530

	Parietal Length	12	1.03	0.154	2.17	0.369	0.78	0.0661
	Frontal Length	12	1.05	0.185	2.16	0.435	0.69	0.0787
Teiidae	Dentary Length	26	0.677	0.0686	2.70	0.165	0.75	0.0995
Scincidae	Maxilla Length	27	1.06	0.0352	2.20	0.0850	0.97	0.115
	Dentary Length	27	1.12	0.0426	1.74	0.115	0.96	0.133
Xantusiidae	Maxilla Length	20	0.698	0.0659	2.82	0.0944	0.88	0.119
	Dentary Length	20	0.787	0.0869	2.58	0.134	0.89	0.124
Iguania	Skull Length	53	1.15	0.0248	1.06	0.0838	0.98	0.0997
	Maxilla Length	55	1.07	0.0303	2.07	0.0824	0.96	0.133
	Dentary Length	55	1.22	0.0402	1.38	0.118	0.94	0.155

Fig 6. Regressions of dentary length to snout-vent length for all extant lizard groups sampled. Shinisauridae is not included here because no regressions were generated for that group. See Table 3 for regression equations and statistics. All regressions are transformed using the natural log (LN).

The group-level regressions generated for body length in this study predicted actual SVL with comparable accuracy between the extant and fossil test specimens (Fig 6, Table 2, S1-S7 Tables). The actual SVL for each extant and fossil test specimen fell within or close to the 95% confidence interval for the estimated SVL (S1-S7 Tables). The percentage difference between predicted and actual SVL of test specimens ranged from 1% (varanid tibia length vs. SVL, SEE = 0.0695; S2 Table) to 36% (anguid frontal length vs. SVL, SEE = 0.120; S1 Table).

When large-bodied outliers were used to test the predictive power of the SVL regressions, the resulting percentage differences between actual and estimated SVL were comparable (see Varanidae, S2 Table) or lower (see Anguidae, S1 Table) than the other test specimens, indicating that those regressions can reliably estimate SVL for large individuals in those families. A large specimen of *Heloderma horridum* (Helodermatidae), the skull of which greatly resembles those of the largest anguid fossils in this dataset (Fig 3E), was used as an additional test specimen for the anguid regressions (S1 Table). Helodermatidae is placed close to Anguidae within the group Anguimorpha in both morphological and molecular phylogenetic analyses [67–70], so it is reasonable to consider Helodermatidae as an extant morphological and close phylogenetic analogue to large extinct anguid lizards. The anguid regressions proved comparably accurate in predicting the SVL of the largest helodermatid test specimen compared to the smaller extant and extinct anguid test specimens.

The family-specific SVL-to-mass equations [24,41,42] and the general SVL-to-mass equation [24] produced similar mass estimates for fossil specimens, with a percentage difference of 1% (Teiidae and Xantusiidae) to 16% (Xenosauridae). The novel element-to-mass equations generated in this study were derived from the published family-specific SVL-to-mass equations, thus, the two sets of equations produced the same mass estimates for fossil specimens.

Body size in fossil lizards through the Paleogene

Maximum lizard SVL remained below 300 mm through the early and middle Paleocene (Puercan (66.0-63.8 Ma) – Tiffanian (60.9-56.2 Ma), Fig 7, Table 3, S1 Dataset, S2 Fig, S10 Table). This upper SVL limit increased to almost 500 mm around the Paleocene-Eocene transition (Clarkforkian (56.2-54.9 Ma)). By the Wasatchian (54.9-50.5 Ma), maximum SVL

reached 750 mm, and surpassed 1 m by the Bridgerian (50.5-46.2 Ma; Table 3). Lizard body mass followed a similar pattern, with lizards exceeding 1 kg (LN(Mass in kg) = 0.00) by the Clarkforkian (Fig 8, Table 3, S1 Dataset, S3 Fig, S10 Table).

Fig 7. Body length distribution by taxonomic group for fossil lizards in the Western Interior of North America through the Paleogene. Body length is measured as snout-vent length (SVL) in mm. All measurements were transformed using natural log (LN). Data points marked with an asterisk (*) were measured from complete fossil skeletons. All others were estimated from individual cranial or limb elements using regressions (see S1-S7 Tables). Dashed line indicates mean lizard SVL per NALMA (see also S5 Fig). Sample sizes are per North American Land Mammal Age (NALMA). Data points are spread laterally within each NALMA for visibility. NALMA abbreviations: Pu = Puercan, To = Torrejonian, Ti = Tiffanian, Cf = Clarkforkian, Wa = Wasatchian, Br = Bridgerian, Ui = Uintan, Du = Duchesnean, Ch = Chadronian, Or = Orellan, Wh = Whitneyan, Ar = Arikareean.

Table 3. Maximum and mean fossil lizard body size estimates per NALMA interval. NALMA age ranges are from Barnosky et al. [78]. Mean values are not listed for the Duchesnean because n = 1 for that NALMA.

**NALMA**	**Age Range (Ma)**	**Crown Group**	**Binomial**	**Specimen**	**Max SVL Estimate (mm)**	**Mean SVL Estimate (mm)**	**Max Mass Estimate (kg)**	**Mean Mass Estimate (kg)**

Puercan	66.0-63.8	Anguidae	*cf. Odaxosaurus piger*	UCM 34991	91	83	0.311	0.276
Torrejonian	63.8-60.9	Varanidae	cf. Varanidae indet.	UCMP 2671	191	113	0.615	0.373
Tiffanian	60.9-56.2	Anguidae	*Melanosaurus sp.*	UCM 98615	256	133	0.885	0.451
Clarkforkian	56.2-54.9	Anguidae	*Melanosaurus maximus*	UMMP 74618	487	269	1.69	0.932
Wasatchian	54.9-50.5	Anguidae	*Melanosaurus sp.*	UCMP 154536	788	232	2.74	0.793
Bridgerian	50.5-46.2	Anguidae	*Glyptosaurus sylvestris*	USNM 12590	1056	408	3.67	1.41
Uintan	46.2-39.7	Varanidae	*Saniwa ensidens*	FMNH UC 1719	540	391	1.74	1.28
Duchesnean	39.7-37.0	Anguidae	Anguidae indet.	CM 42469	116	N/A	0.399	N/A
Chadronian	37.0-33.9	Anguidae	*Helodermoides sp.*	UW 11057	1296	459	4.51	1.59
Orellan	33.9-31.8	Anguidae	*Peltosaurus sp.*	UCM 20877	360	176	1.25	0.604
Whitneyan	31.8-29.5	Anguidae	*Peltosaurus sp.*	SMM P81.8.71	224	194	0.775	0.669
Arikareean	29.5-23.0	Anguidae	*Peltosaurus sp.*	UNSM 81001	351	262	1.22	0.906

Fig 8. Body mass distribution by taxonomic group for fossil lizards in the Western Interior of North America through the Paleogene. Mass estimates were calculated from snout-vent length (SVL) using the published equations listed in S8 Table. All measurements (in kg) were transformed using natural log (LN). Data points marked with an asterisk (*) used SVLs

measured from complete fossil skeletons. All others used SVLs estimated from individual cranial or limb element lengths using regressions (see S1-S7 Tables). Dashed line indicates mean lizard body mass per NALMA (see also S6 Fig). Sample sizes are per North American Land Mammal Age (NALMA). Data points are spread laterally within each NALMA for visibility. NALMA abbreviations: Pu = Puercan, To = Torrejonian, Ti = Tiffanian, Cf = Clarkforkian, Wa = Wasatchian, Br = Bridgerian, Ui = Uintan, Du = Duchesnean, Ch = Chadronian, Or = Orellan, Wh = Whitneyan, Ar = Arikareean.

The late Eocene record (Chadronian, 37.0-33.9 Ma) featured several large individuals, including the largest body size estimated in this study (1.3 m, 4.5 kg, *Helodermoides sp.*, Table 3). However, by the early Oligocene (Orellan, 33.9-31.8 Ma), maximum body size dropped considerably (360 mm, 1.25 kg, *Peltosaurus sp.*) and remained below that limit through the late Paleogene.

Discussion

Strength of body size regression equations

This study found skull length to be the strongest cranial element predictor of SVL in Anguidae and Iguania, in agreement with previous studies [12,21,22]. Limb bones were also strong predictors of SVL in Anguidae (femur and humerus length) and Varanidae (tibia length), as was previously demonstrated in crocodylians [27]. These elements were not available for the other lizard crown groups investigated in this study. Among those (Scincidae, Shinisauridae, Teiidae, Xantusiidae, Xenosauridae), maxilla length provided the most consistently accurate SVL estimate compared to SVL measurements from test specimens (S1-S2 Tables, S5-S7

Tables), followed by dentary length (S1-S7 Tables). The body mass equations could not be tested for accuracy in the same way because body mass data were not available for the extant test specimens. In terms of deviation from the general equation for all lizards, the body mass estimations from equations for Anguimorpha (used for Shinisauridae), Iguania, Teiidae, Varanidae, and Xantusiidae were the closest to the general estimation, with a percentage difference of 1-5% (S1 Dataset, S8 Table).

Attempts to derive general equations for calculating SVL from individual bones proved unsuccessful in this study (S9 Table). This might be because cranial element morphology and proportions often vary between closely related lizard groups [79]. Meiri's [24] general equation for predicting mass from SVL for lizards (see S1 Dataset) was likely more viable because the relationship between SVL and mass appears to be more consistent than the relationship between head length or any individual element to SVL across lizard taxonomic groups.

Separate regressions for males and females were not generated because most extant specimens sampled did not have a sex indicated. However, it was not an issue for this study because any fossil lizard dataset must be treated as an averaged sample across sexes. Although female lizards often have shorter SVLs than males [4], there is no reliable way to tell the sex of an individual fossil lizard specimen based on isolated bones.

The same is largely true for ontogenetic stages represented in a fossil dataset. Adult members of an extinct taxon can be reasonably identified if a large sample of referred individuals that reach a consistent maximum size range is present (e.g., large fossil anguids, S1 Dataset) or if some of those specimens include long bones with fused epiphyses. Complete anatomical elements from adult individuals are also more likely to get preserved as fossils. Juveniles are less likely to get preserved as fossils because they are frequently consumed by predators and their

bones are more delicate [80]. This preservation bias toward adult specimens would not affect the resulting patterns of maximum body size for any NALMA with a decent sample size, even if some subadult or juvenile specimens were present in the fossil dataset. For these reasons, and to avoid skewing the regression equations with outliers, juvenile specimens were excluded from the extant dataset.

Body size evolution among Paleogene lizard groups

Anguids are known to be the most abundant lizards in the Paleogene fossil record of the Western Interior [81–83] and that was reflected in their dominance of the fossil dataset presented here (S1 Dataset). Anguids also accounted for the largest individuals in the dataset and most of the maximum sizes in each NALMA, with the exception of a few varanids (Figs 7-8, S2-S3 Figs, Table 3, S10 Table). Among the eight lizard crown groups sampled, only those two reached SVLs over 200 mm and masses over 1 kg. Interestingly, the largest extant limbed anguid today, *Diploglossus millepunctatus* (maximum SVL 280 mm), does not approach the size of the largest anguids from the Eocene, nor do the largest lizards occurring in North America today (*Heloderma horridum,* maximum SVL 520 mm [84], Fig 3E; *Iguana iguana,* maximum SVL 580 mm [4]). Conversely, Varanidae includes both the largest extant lizard (*Varanus komodoensis,* which exceeds the adult size of the largest Eocene anguid at around 1.5 m SVL [4,85]) and the largest known terrestrial lizard in the fossil record (*Varanus priscus*, known from Pleistocene deposits in Australia, with SVL at least 3 m [86,87]), but varanids are now restricted to the Eastern Hemisphere [13].

The other lizard groups in this geotemporal system (Iguania, Scincidae, Shinisauridae, Teiidae, Xantusiidae, Xenosauridae) did not experience an increase in body size range (Figs 7-8,

S10 Table). Small-bodied lizards dominated the North American Paleogene lizard record. The maximum and mean body sizes for most lizard groups did not exceed 200 mm in SVL (LN(SVL) = 5.30, S2 Fig, S10 Table) or 1 kg in body mass (LN(Mass) = 0.00, S3 Fig, S10 Table).

The observed maximum body size for the middle Eocene (540 mm, 1.74 kg, *Saniwa ensidens* (Varanidae), Figs 6-7, Table 3, S10 Table) was much lower than that of the early or late Eocene. Fewer outcrops are available from this interval in the U.S. Western Interior, so this result was likely an artifact of poor sampling (n = 4 for the Uintan (46.2-39.7 Ma), n = 1 for the Duchesnean (39.7-37.0 Ma), Fig 7). Rarefaction analysis (S4 Fig) demonstrated that the number of expected size groups recovered in a sample from this fossil dataset again peaked and leveled off around n = 50, supporting the assumption that the NALMAs with small sample sizes in this study did not capture the full range of lizard body size that was likely present during those intervals.

The decrease in maximum body size in the early Oligocene cannot be attributed to low sample size because the Orellan (33.9-31.8 Ma) was very well sampled (n = 64) compared to other NALMAs (Figs 6-7, S4 Fig). In fact, sample size did not correlate strongly with maximum estimated SVL across the entire dataset (R^2 = 0.23, p(uncorr.) = 0.14 for sample size *n* vs. LN(Max SVL)). The observed late Paleogene reductions in maximum body size likely represent the extirpation or extinction of the largest Paleogene lizard taxa in the region [88,89]. Even in extant lizards, extinction risk increases with body size [3]. Among the eight lizard groups documented in this study from the Paleogene record of this region, only Anguidae, Scincidae, Teiidae, and Xantusiidae still occur in the area. Xenosaurids and the iguanians included in this study that occurred in the Western Interior during the Paleogene have since been ~~extirpated~~ from the region; varanids and shinisaurids no longer occur anywhere in North America [4,11,13,85].

In general, the observed patterns may reflect some taphonomic or preservation bias (especially with respect to smaller bones), gaps in the rock record, and selective sampling by fossil collection crews. However, rarefaction analysis (S4 Fig) indicated that this study adequately sampled the upper range of body size diversity represented overall in this fossil record, especially from thoroughly documented areas like the Bighorn and Green River Basins (S1 Dataset; for further discussion, see the censuses of microvertebrate localities in this system by Smith [56–60]). Preservation bias toward complete elements from adult individuals may have mitigated other sources of bias when reconstructing maximum body size through geologic intervals.

Climate as a potential driver of body size evolution

Environmental temperature can influence body size evolution in reptiles [3,9,31,90–92]. Metabolic rate increases with temperature and scales with body size in all poikilothermic vertebrates [1,93,94], including lizards [15,95]. Changes in environmental temperature are known to correlate globally with changes in maximum body size in extant lizards (exclusive of snakes [25]). Increased temperatures around the Paleocene-Eocene Thermal Maximum (PETM, 56 Ma; [44,96]) could have elevated metabolism and released a physiological constraint on maximum size for some lizard taxa. Gigantism has been documented in other fossil reptiles in conjunction with elevated temperatures, including a boid snake (*Titanoboa*, total length 13 m, 60-58 Ma, Colombia [31]), a caimanine crocodyliform (*Purussaurus,* total length 12.5 m, 8 Ma, northern South America [97]), and a varanid lizard (*Varanus priscus,* total length up to 5.7 m, around 50 ka, Australia [86,87]).

To investigate this possibility, RMA linear regression was applied to test whether maximum and mean fossil lizard SVL predicted global temperature per NALMA (S5 Fig, S10 Table). Global temperature data were obtained from Zachos et al. [43,44], a widely used dataset of Cenozoic paleotemperatures derived from marine oxygen isotopes (e.g., [10,49]). Here, fossil lizard body size showed no relationship to global temperature through the Paleogene. It is possible that the relationship observed today is not detectable across scales of millions of years, perhaps due to taphonomic or sampling bias in the fossil record. It might also be more pertinent to compare these data with terrestrial paleotemperature proxies from the same geotemporal system sampled in this study. This could be a fruitful area for continued research to build on the data presented here.

Alternatively, body size evolution patterns observed here could reflect indirect effects of climate [98]. Temperatures were warmer during both the summer and winter months around the PETM, and most precipitation came during the summer [99–101]. This would have allowed lizards to be active and growing during a longer portion of the year [98,102] and could also have led to greater food availability from increased productivity, especially in the summer months [103–105]. Previous studies found elevated growth rates in extant tropical lizards occupying areas that received greater precipitation [106]. Attaining large body size in a closed tropical forest system with an equable climate, like that present in the Western Interior in the early Eocene [103,104,107,108], would also have allowed lizards to spend less time and energy on active thermoregulation [1,109]. Large body size sometimes corresponds with thermoconforming behavior and nocturnal activity in extant lizards [3,12,102], and these strategies could have reduced competition with diurnal organisms. Herbivory can also lead to large body size in lizards [3,4,95]. This has been inferred for at least one giant fossil lizard [9]. However, the dentition of

the largest lizards in this study were more indicative of omnivorous or insectivorous (Anguidae, Fig 3A) and carnivorous diets (Varanidae, Fig 4B; see [21,110]), rather than herbivory.

Lizards can maintain smaller body sizes in warm climates as a result of behavioral thermoregulation [109], competition, predation, or specializations related to resource zones of small taxa [92,111]. This might explain why not all lizard groups in this fossil dataset exhibited an increase in body size. These results may reflect an ecological hierarchy in the Paleogene communities of the Western Interior similar to extant ecosystems in which large-bodied lizards are less abundant than small-bodied lizards [3].

Conclusions

This study generated novel equations for estimating fossil lizard body size from isolated anatomical elements, with skull length, limb bone length, and maxilla length providing the most accurate estimates for SVL. Body mass can also be calculated from individual bones based on these equations. Application of these equations to the Paleogene record of lizards in the Western Interior of North America offers the first survey and reconstruction of body size across crown lizard groups through a prolonged geologic interval and across a large continental interior region. The findings indicate that lizard body size peaked in the early Eocene in this system. Only two lizard groups, Anguidae and Varanidae, reached large body sizes exceeding 1 m in SVL and 1 kg in mass. Body size range did not change considerably for other groups. Maximum lizard body size decreased across the Western Interior in the early Oligocene. The methods presented here can be applied to other lizard clades to generate reasonably accurate body size estimates for fossil taxa and to study patterns of body size evolution across higher taxonomic groups.

Acknowledgments

The author wishes to thank K. Padian, J. McGuire, A. Barnosky, and I. Wang for their mentorship and helpful comments on this work, as well as J. Head, who mentored the research that started this project. The following individuals provided assistance and access to museum collections: M. Norell, C. Mehling, J. Meng, D. Kizirian, L. Vonnahme, M. Arnold (AMNH); J. Vindum, L. Scheinberg, E. Ely (CAS); M. Lamanna, J. Padial, A. Henrici, S. Rogers (CM); T. Lyson, L. Ivy, K. MacKenzie (DMNH); J. Bloch, R. Hulbert, J. Bourque (FLMNH); P. Makovicky, L. Grande, A. Resetar, W. Simpson, A. Stroupe, J. Mata (FMNH); A. Aase (FOBU); N. Smith, S. McLeod, V. Rhue (LACM); J.Rosado (MCZ); J. McGuire, C. Spencer (MVZ); T. Williamson (NMMNH); A. Farke, G. Santos (RAM); A. Hastings, E. Whiting (SMM); C. Bell, M. Brown (TMM); T. Culver, N. Ridgwell (UCM); P. Holroyd, C. Marshall (UCMP); J. Wilson, A. Rountrey (UMMP); R. Hunt, Jr., P. Freeman, G. Corner, T. Labedz (UNSM); H. Dieter-Sues, M. Carrano, M. Brett-Surman, A. Millhouse (USNM); L. Vietti (UW); C. Sidor, R. Eng, M. Rivin (UWBM); J. Gauthier, C. Norris, D. Brinkman (YPM). The author thanks the following for helpful discussions: C. Badgley, C. Bell, B-A. Bhullar, J. Bloch, W. Clemens[†], C. Crumly, J. Gauthier, W. Gearty, P. Gingerich, L. Grande, A. Gunderson, P. Holroyd, E. Holt, R. Huey, R. Hunt, Jr., M. Kemp, D. Meyer, D. Miles, J. McGuire, J. Parham, H. Petermann, R. Secord, M. Stocker, R. Sullivan, D. Watkins, L. Weaver, S. Werning, E. Whiting, C. Williams, the MVZ Herp Group, the entire UCMP community. C. Bell also provided access to obscure literature. D. Meyer provided measurements of an unpublished specimen. G. Brown (UNSM) prepared a new *Peltosaurus* specimen (UNSM 12102) containing critical limb material. F. Vasconcellos provided the photo in Fig 3E. B. Chelemedos and C. Wong assisted with data collection. E. Whiting provided instruction on using QGIS to make maps. J. Lipps helped to design a visually

accessible color palette for Fig 2. The author thanks C. Meloro, S. Scarpetta, and two anonymous reviewers for their constructive comments which greatly helped to improve the manuscript.

References

1. Peters RH. The Ecological Implications of Body Size. 1st ed. Beck E, Birks HJB, Connor EF, editors. New York, NY: Cambridge University Press; 1983.

2. Schmidt-Nielsen K. Scaling: Why is Animal Size So Important? Cambridge: Cambridge University Press; 1984.

3. Meiri S. Evolution and ecology of lizard body sizes. Glob Ecol Biogeogr. 2008;17: 724–734. doi:10.1111/j.1466-8238.2008.00414.x

4. Meiri S. Traits of lizards of the world: Variation around a successful evolutionary design. Glob Ecol Biogeogr. 2018;27: 1168–1172. doi:10.1111/geb.12773

5. Damuth J. Problems in estimating body masses of archaic ungulates using dental measurements. Body Size Mamm Paleobiol Estim Biol Implic. 1990;229: tables 16.9-16.10.

6. Figueirido B, Pérez-Claros JA, Hunt RM, Palmqvist P. Body mass estimation in amphicyonid carnivoran mammals: A multiple regression approach from the skull and skeleton. Acta Palaeontol Pol. 2011;56: 225–246. doi:10.4202/app.2010.0005

7. Campione NE, Evans DC. A universal scaling relationship between body mass and proximal limb bone dimensions in quadrupedal terrestrial tetrapods. BMC Biol. 2012;10. doi:10.1186/1741-7007-10-60

8. Thomas Goodwin H, Bullock KM. Estimates of body mass for fossil giant ground squirrels, genus *Paenemarmota*. J Mammal. 2012;93: 1169–1177. doi:10.1644/11-MAMM-A-312.3

9. Head JJ, Gunnell GF, Holroyd PA, Howard Hutchison J, Ciochon RL. Giant lizards occupied herbivorous mammalian ecospace during the Paleogene greenhouse in Southeast

Asia. Proc R Soc B Biol Sci. 2013;280. doi:10.1098/rspb.2013.0665

10. Godoy PL, Benson RBJ, Bronzati M, Butler RJ. The multi-peak adaptive landscape of crocodylomorph body size evolution. BMC Evol Biol. 2019;19: 1–29. doi:10.1186/s12862-019-1466-4

11. Vidan E, Novosolov M, Bauer AM, Herrera FC, Chirio L, Campos Nogueira C, et al. The global biogeography of lizard functional groups. J Biogeogr. 2019;00: 1–12. doi:10.1111/jbi.13667

12. Pianka ER, Vitt LJ, Pelegrin N, Fitzgerald DB, Winemiller KO. Toward a Periodic Table of Niches, or Exploring the Lizard Niche Hypervolume. Am Nat. 2017;190: 000–000. doi:10.1086/693781

13. Pianka ER, Vitt LJ. Lizards: Windows to the Evolution of Diversity. Berkeley: University of California Press; 2003.

14. Vitt LJ, Pianka ER, Cooper WE, Schwenk K. History and the global ecology of squamate reptiles. Am Nat. 2003;162: 44–60. doi:10.1086/375172

15. Pough FH. The advantages of ectothermy for tetrapods. Am Nat. 1980;115: 92–112. doi:10.1086/283547

16. Muñoz MM, Bodensteiner BL. Janzen’s Hypothesis meets the Bogert Effect: Connecting climate variation, thermoregulatory behavior, and rates of physiological evolution. Integr Org Biol. 2019;1: 1–12. doi:10.1093/iob/oby002

17. Muñoz MM, Losos JB. Thermoregulatory behavior simultaneously promotes and forestalls evolution in a tropical lizard. Am Nat. 2018;191: E15–E26. doi:10.1086/694779

18. Muñoz MM, Stimola MA, Algar AC, Conover A, Rodriguez AJ, Landestoy MA, et al. Evolutionary stasis and lability in thermal physiology in a group of tropical lizards. Proc R

Soc B Biol Sci. 2014;281: 20132433. doi:10.1098/rspb.2013.2433

19. Roll U, Feldman A, Novosolov M, Allison A, Bauer AM, Bernard R, et al. The global distribution of tetrapods reveals a need for targeted reptile conservation. Nat Ecol Evol. 2017;1: 1677–1682. doi:10.1038/s41559-017-0332-2

20. Vitt E.R. Pianka, W.E. Cooper, Jr., and K. Schwenk LJ. History and the global ecology of squamate reptiles. Am Nat. 2003;162: 44–60.

21. Herrera-Flores JA, Stubbs TL, Benton MJ. Ecomorphological diversification of squamates in the Cretaceous. R Soc Open Sci. 2021;8: 1–11. doi:10.1098/rsos.201961

22. Metzger KA, Herrel A. Correlations between lizard cranial shape and diet: A quantitative, phylogenetically informed analysis. Biol J Linn Soc. 2005;86: 433–466. doi:10.1111/j.1095-8312.2005.00546.x

23. Olalla-Tárraga MÁ, Rodríguez MÁ, Hawkins BA. Broad-scale patterns of body size in squamate reptiles of Europe and North America. J Biogeogr. 2006;33: 781–793. doi:10.1111/j.1365-2699.2006.01435.x

24. Meiri S. Length-weight allometries in lizards. J Zool. 2010;281: 218–226. doi:10.1111/j.1469-7998.2010.00696.x

25. Slavenko A, Feldman A, Allison A, Bauer AM, Böhm M, Chirio L, et al. Global patterns of body size evolution in squamate reptiles are not driven by climate. Glob Ecol Biogeogr. 2019;28: 471–483. doi:10.1111/geb.12868

26. Villa A, Delfino M. A comparative atlas of the skull osteology of European lizards (Reptilia: Squamata). Zool J Linn Soc. 2019; 1–100. doi:10.1093/zoolinnean/zlz035

27. Farlow JO, Hurlburt GR, Elsey RM, Britton ARC, Langston W. Femoral dimensions and body size of *Alligator mississippiensis*: Estimating the size of extinct mesoeucrocodylians.

J Vertebr Paleontol. 2005;25: 354–369. doi:10.1671/0272-4634(2005)025[0354:FDABSO]2.0.CO;2

28. O'Brien HD, Lynch LM, Vliet KA, Brueggen J, Erickson GM, Gignac PM. Crocodylian Head Width Allometry and Phylogenetic Prediction of Body Size in Extinct Crocodyliforms. Integr Org Biol. 2019;1. doi:10.1093/iob/obz006

29. Egi N. Body mass estimates in extinct mammals from limb bone dimensions: The case of North American Hyaenodontids. Palaeontology. 2001;44: 497–528. doi:10.1111/1475-4983.00189

30. Valkenburgh B Van. Skeletal and dental predictors of body mass in carnivores. Body size in Mammalian Paleobiology: Estimation and biological implications. 1990. pp. 181–205.

31. Head JJ, Bloch JI, Hastings AK, Bourque JR, Cadena EA, Herrera FA, et al. Giant boid snake from the Palaeocene neotropics reveals hotter past equatorial temperatures. Nature. 2009;457: 715–718. doi:10.1038/nature07671

32. Sereno PC, Larsson HCE, Sidor CA, Gado B. The giant crocodyliform *Sarcosuchus* from the cretaceous of Africa. Science (80-). 2001;294: 1516–1519. doi:10.1126/science.1066521

33. Bochaton C, Kemp ME. Reconstructing the body sizes of Quaternary lizards using *Pholidoscelis Fitzinger*, 1843, and Anolis Daudin, 1802, as case studies. J Vertebr Paleontol. 2017;37: 9 pp. doi:10.1080/02724634.2017.1239626

34. Herrera-Flores JA, Elsler A, Stubbs TL, Benton MJ. Slow and fast evolutionary rates in the history of lepidosaurs. Palaeontology. 2022;65: 1–13. doi:10.1111/pala.12579

35. Arnold EN. Evolutionary aspects of tail shedding in lizards and their relatives. J Nat Hist. 1984;18: 127–169. doi:10.1080/00222938400770131

36. Bateman PW, Fleming PA. To cut a long tail short: A review of lizard caudal autotomy studies carried out over the last 20 years. J Zool. 2009;277: 1–14. doi:10.1111/j.1469-7998.2008.00484.x

37. Gilbert EAB, Payne SL, Vickaryous MK. The anatomy and histology of caudal autotomy and regeneration in lizards. Physiol Biochem Zool. 2013;86: 631–644. doi:10.1086/673889

38. Sanggaard KW, Danielsen CC, Wogensen L, Vinding MS, Rydtoft LM, Mortensen MB, et al. Unique Structural Features Facilitate Lizard Tail Autotomy. PLoS One. 2012;7. doi:10.1371/journal.pone.0051803

39. Zani PA. Patterns of caudal-anatomy evolution in lizards. J Zool. 1996;240: 201–230.

40. Greer AE. Distribution of Maximum Snout-Vent Length among Species of Scincid Lizards. J Herpetol. 2001;35: 383–395.

41. Meiri S, Bauer AM, Chirio L, Colli GR, Das I, Doan TM, et al. Are lizards feeling the heat?: a tale of ecology and evolution under two temperatures. Glob Ecol Biogeogr. 2013;22: 834–845.

42. Feldman A, Sabath N, Pyron RA, Mayrose I, Meiri S. Body sizes and diversification rates of lizards, snakes, amphisbaenians and the tuatara. Glob Ecol Biogeogr. 2016;25: 187–197. doi:10.1111/geb.12398

43. Zachos J, Pagani M, Sloan L, Thomas E, Billups K. Trends, rhythms and aberrations in global climate 65 Ma to present. Science (80-). 2001;292: 686–693.

44. Zachos JC, Dickens GR, Zeebe RE. An early Cenozoic perspective on greenhouse warming and carbon-cycle dynamics. Nature. 2008;451: 279–283. doi:10.1038/nature06588

45. Woodburne MO, Gunnell GF, Stucky RK. Climate directly influences Eocene mammal faunal dynamics in North America. Proc Natl Acad Sci. 2009;106: 13399–13403. doi:10.1073/pnas.0906802106

46. Gunnell GF, Zonneveld JP, Bartels WS. Stratigraphy, mammalian paleontology, paleoecology, and age correlation of the Wasatch Formation, Fossil Butte National Monument, Wyoming. J Paleontol. 2016;90: 981–1011. doi:10.1017/jpa.2016.100

47. Saarinen JJ, Boyer AG, Brown JH, Costa DP, Ernest M, Evans AR, et al. Patterns of maximum body size evolution in Cenozoic land mammals: eco-evolutionary processes and abiotic forcing. Proc R Soc B Biol Sci. 2014;281: 1–10.

48. Rose KD. Composition and Species Diversity in Paleocene and Eocene Mammal Assemblages: An Empirical Study. J Vertebr Paleontol. 1981;1: 367–388.

49. Lovegrove BG, Mowoe MO. The evolution of mammal body sizes: Responses to Cenozoic climate change in North American mammals. J Evol Biol. 2013;26: 1317–1329. doi:10.1111/jeb.12138

50. Lyson TR, Miller IM, Bercovici AD, Weissenburger K, Fuentes AJ, Clyde WC, et al. Exceptional continental record of biotic recovery after the Cretaceous−Paleogene mass extinction. Science (80-). 2019;366: 977–983. doi:10.1126/science.aay2268

51. Alroy J. Constant extinction, constrained diversification, and uncoordinated stasis in North American mammals. Palaeogeogr Palaeoclimatol Palaeoecol. 1996;127: 285–311.

52. Alroy J. Cope’s Rule and the Dynamics of Body Mass Evolution in North American Fossil Mammals. Science (80-). 1998;280: 731–734.

53. Alroy J. New Methods for Quantifying Macroevolutionary Patterns and Processes. Paleobiology. 2000;26: 707–733.

54. Alroy J. Speciation and extinction in the fossil record of North American mammals. In: Butlin RK, Brindle JR, Schluter D, editors. Speciation and Patterns of Diversity. Cambridge University Press; 2009. pp. 301–323.

55. Huang S, Eronen JT, Janis CM, Saarinen JJ, Silvestro D, Fritz SA. Mammal body size evolution in North America and Europe over 20 Myr: Similar trends generated by different processes. Proc R Soc B Biol Sci. 2017;284: 1–9. doi:10.1098/rspb.2016.2361

56. Smith KT, Gauthier JA. Early Eocene lizards of the Wasatch Formation near Bitter Creek, Wyoming: Diversity and paleoenvironment during an interval of global warming. Bull Peabody Museum Nat Hist. 2013;54: 135–230. doi:10.3374/014.054.0205

57. Smith KT. A diverse new assemblage of Late Eocene squamates (Reptilia) from the Chadron Formation of North Dakota, U.S.A. Palaeontol Electron. 2006;9: 1–44.

58. Smith KT. A new lizard assemblage from the earliest Eocene (zone Wa0) of the Bighorn Basin, Wyoming, USA: Biogeography during the warmest interval of the Cenozoic. Journal of Systematic Palaeontology. 2009. doi:10.1017/S1477201909002752

59. Smith KT. The evolution of mid-latitude faunas during the Eocene: Late Eocene lizards of the Medicine Pole Hills reconsidered. Bull Peabody Museum Nat Hist. 2011;52: 3–105.

60. Smith KT. The long-term history of dispersal among lizards in the early Eocene: New evidence from a microvertebrate assemblage in the Bighorn Basin of Wyoming, USA. Palaeontology. 2011;54: 1243–1270. doi:10.1111/j.1475-4983.2011.01107.x

61. Woolley CH, Thompson JR, Wu Y-H (Beckey), Bottjer DJ, Smith ND. Can a fragmented past be trusted? Assessing bias and phylogenetic signal in the squamate fossil record. J Vertebr Paleontol Progr Abstr. 2020; 351–352.

62. Woolley CH, Thompson JR, Wu YH, Bottjer DJ, Smith ND. A biased fossil record can

preserve reliable phylogenetic signal. Paleobiology. 2022; 1–16. doi:10.1017/pab.2021.45

63. Bell CJ, Gauthier JA, Bever GS. Covert biases, circularity, and apomorphies: A critical look at the North American Quaternary Herpetofaunal Stability Hypothesis. Quat Int. 2010;217: 30–36. doi:10.1016/j.quaint.2009.08.009

64. Ledesma DT, Scarpetta SG, Bell CJ. Variation in the skulls of *Elgaria* and *Gerrhonotus* (Anguidae, Gerrhonotinae) and implications for phylogenetics and fossil identification. PeerJ. 2021;9: 1–80. doi:10.7717/peerj.11602

65. Scarpetta SG, Ledesma DT, Bell CJ. A new extinct species of alligator lizard (Squamata: *Elgaria*) and an expanded perspective on the osteology and phylogeny of Gerrhonotinae. BMC Ecol Evol. 2021;21: 1–58. doi:10.1186/s12862-021-01912-8

66. The Paleobiology Database. Available: www.paleobiodb.org

67. Estes R, De Queiroz K, Gauthier J. Phylogenetic Relationships within Squamata. In: Estes R, Pregill G, editors. Phylogenetic Relationships of the Lizard Families. Palo Alto, CA: Stanford University Press; 1988. pp. 119–281.

68. Frost DR, Etheridge R. A phylogenetic analysis and taxonomy of iguanian lizards. Univ Kansas Museum Nat Hist Misc Publ. 1989;81: 1–65.

69. Gauthier JA, Kearney M, Maisano JA, Rieppel O, Behlke ADB. Assembling the Squamate Tree of Life : Perspectives from the Phenotype and the Fossil Record Assembling the Squamate Tree of Life : Perspectives from the Phenotype and the Fossil Record. Bull Peabody Museum Nat Hist. 2012;53: 3–308. doi:10.3374/014.053.0101

70. Wiens JJ, Hutter CR, Mulcahy DG, Noonan BP, Townsend TM, Sites JW, et al. Resolving the phylogeny of lizards and snakes (Squamata) with extensive sampling of genes and species. Biol Lett Lett. 2012;8: 1043–1046. doi:10.5061/dryad.g1gd8

71. Hammer Ø, Harper DAT, Ryan PD. PAST: Palaeontological Statistics software package for education and data analysis. Palaeontol Electron. 2001;4: 1–9.

72. Gingerich PD. Arithmetic or geometric normality of biological variation: An empirical test of theory. J Theor Biol. 2000;204: 201–221. doi:10.1006/jtbi.2000.2008

73. Sokal R, Rohlf F. Biometry: The principles and practice of statistics in biological science. San Francisco: W. H. Freeman and Company; 1969.

74. Field DJ, Lynner C, Brown C, Darroch SAF. Skeletal correlates for body mass estimation in modern and fossil flying birds. PLoS One. 2013;8: 1–13. doi:10.1371/journal.pone.0082000

75. Alberdi MT, Prado JL, Ortiz-Jaureguizar E. Patterns of body size changes in fossil and living Equini. Biol J Linn Soc. 1995;54: 349–370.

76. Conrad JL. An Eocene shinisaurid (Reptilia, Squamata) from Wyoming, U.S.A. J Vertebr Paleontol. 2006;26: 113–126. doi:10.1671/0272-4634(2006)26[113:AESRSF]2.0.CO;2

77. Woodburne MO, editor. Late Cretaceous and Cenozoic Mammals of North America: Biostratigraphy and Geochronology. New York: Columbia University Press; 2004.

78. Barnosky AD, Holmes M, Kirchholtes R, Lindsey E, Maguire KC, Poust AW, et al. Prelude to the Anthropocene: Two new North American Land Mammal Ages (NALMAs). Anthr Rev. 2014;1: 225–242. doi:10.1177/2053019614547433

79. Evans SE. The Skull of Lizards and Tuatara. Volume 20,. In: Gans C, Gaunt AS, Adler K, editors. The Skull of Lepidosauria. Volume 20,. Ithaca, NY: Society for the Study of Amphibians and Reptiles; 2008. pp. 1–348.

80. Angilletta, Jr. MJ, Niewiarowski PH, Dunham AE, Leaché AD, Porter WP. Bergmann's Clines in Ectotherms: Illustrating a Life- History Perspective with Sceloporine Lizards.

Am Nat. 2004;164: E168–E183. doi:10.1086/425222

81. ElShafie SJ. Body size and species richness changes in Glyptosaurinae (Squamata: Anguidae) through climatic transitions of the North American Cenozoic. Master's Thesis. Department of Earth & Atmospheric Sciences: University of Nebraska, Lincoln. University of Nebraska, Lincoln. 2014.

82. Meszoely CAM. North American fossil anguid lizards. Bull Museum Comp Zool. 1970;139: 87–150.

83. Gilmore CW. Fossil Lizards of North America. Mem Natl Acad Sci. 1928;22: v–201.

84. Beck DD. Biology of Gila Monsters and Beaded Lizards. 9th ed. Berkeley: University of California Press; 2005.

85. Pianka ER. Evolution of body size: Varanid lizards as a model system. Am Nat. 1995;146: 398–414.

86. Molnar RE. Dragons in the dust: the paleobiology of the giant monitor lizard *Megalania*. Indiana University Press; 2004.

87. Conrad JL, Balcarcel AM, Mehling CM. Earliest Example of a Giant Monitor Lizard (*Varanus*, Varanidae, Squamata). PLoS One. 2012;7: e41767. doi:10.1371/journal.pone.0041767

88. Sullivan RM. The taxonomy, chronostratigraphy and paleobiogeography of glyptosaurine lizards (Glyptosaurinae, Anguidae). Comptes Rendus Palevol. 2019;18: 747–763. doi:10.1016/j.crpv.2019.05.006

89. Scarpetta SG. *Peltosaurus granulosus* (Squamata, Anguidae) from the middle Oligocene of Sharps Corner, South Dakota, and the youngest known chronostratigraphic occurrence of Glyptosaurinae. J Vertebr Paleontol. 2019;39: e1622129.

doi:10.1080/02724634.2019.1622129

90. Pincheira-Donoso D, Meiri S. An Intercontinental Analysis of Climate-Driven Body Size Clines in Reptiles: No Support for Patterns, No Signals of Processes. doi:10.1007/s11692-013-9232-9

91. Piantoni C, Navas CA, Ibargüengoytía NR. A real tale of Godzilla: Impact of climate warming on the growth of a lizard. Biol J Linn Soc. 2019;126: 768–782. doi:10.1093/biolinnean/bly216

92. Kingsolver JG, Huey RB. Size, temperature, and fitness: Three rules. Evol Ecol Res. 2008;10: 251–268.

93. Makarieva AM, Gorshkov VG, Li BL. Gigantism, temperature and metabolic rate in terrestrial poikilotherms. Proc R Soc B Biol Sci. 2005;272: 2325–2328. doi:10.1098/rspb.2005.3223

94. Makarieva AM, Gorshkov VG, Li BL. Temperature-associated upper limits to body size in terrestrial poikilotherms. Oikos. 2005;111: 425–436. doi:10.1111/j.1600-0706.2005.14095.x

95. Pough FH. Lizard Energetics and Diet. Ecology. 1973;54: 837–844. doi:10.2307/1935678

96. Wing SL, Harrington GJ, Smith FA, Bloch JI, Boyer DM, Freeman KH. Transient floral change and rapid global warming at the Paleocene-Eocene boundary. Science (80-). 2005;310: 993–996. doi:10.1126/science.1116913

97. Aureliano T, Ghilardi AM, Guilherme E, Souza-Filho JP, Cavalcanti M, Riff D. Morphometry, bite-force, and paleobiology of the Late Miocene caiman *Purussaurus brasiliensis*. PLoS One. 2015;10: 1–14. doi:10.1371/journal.pone.0117944

98. Sinervo B, Méndez-de-la-Cruz F, Miles DB, Heulin B, Bastiaans E, Cruz MVS, et al.

Erosion of lizard diversity by climate change and altered thermal niches. Science (80-). 2010;328: 894–899. doi:10.1126/science.1184695

99. Gregory KM, McIntosh WC. Paleoclimate and paleoelevation of the Oligocene Pitch-Pinnacle flora, Sawatch Range, Colorado. Geol Soc Am Bull. 1996;108: 545. doi:10.1130/0016-7606(1996)108<0545:PAPOTO>2.3.CO;2

100. Snell KE. Paleoclimate and paleoelevation of the western Cordillera in the United States. University of California, Santa Cruz. 2011.

101. Snell KE, Thrasher BL, Eiler JM, Koch PL, Sloan LC, Tabor NJ. Hot summers in the Bighorn Basin during the early Paleogene. Geology. 2013;41: 55–58. doi:10.1130/G33567.1

102. Huey RB, Slatkin M. Cost and benefits of lizard thermoregulation. Q Rev Biol. 1976;51: 363–384.

103. Wing SL. Eocene and Oligocene floras and vegetation of the Rocky Mountains. Ann Missouri Bot Gard. 1987;74: 748. doi:10.2307/2399449

104. Wing SL, Bown TM, Obradovich JD. Early Eocene biotic and climatic change in interior western North America. Geology. 1991;19: 1189–1192. doi:10.1130/0091-7613(1991)019<1189:EEBACC>2.3.CO;2

105. Stucky RK. Mammalian faunas in North America of Bridgerian to Early Arikareean "Ages" (Eocene and Oligocene). In: Prothero DR, Berggren WA, editors. Eocene-Oligocene Climatic and Biotic Evolution. Princeton, NJ: Princeton University Press; 1992. pp. 464–493.

106. Stamps JA. Rainfall, Moisture and Dry Season Growth Rates in *Anolis aeneus*. Copeia. 1977;1977: 415–419.

107. Wing SL, Greenwood DR. Fossils and fossil climate: The case for equable continental interiors in the Eocene. Philos Trans - R Soc London, B. 1993;341: 243–252. doi:10.1098/rstb.1993.0109

108. Fricke HC, Wing SL. Oxygen isotope and paleobotanical estimates of temperature and d18O-latitude gradients over North America during the early Eocene. Am J Sci. 2004;304: 612–635.

109. Huey RB. Behavioral thermoregulation in lizards: Importance of associated costs. Science (80-). 1974;184: 1001–1003.

110. Lafuma F, Corfe IJ, Clavel J, Di-Poï N. Multiple evolutionary origins and losses of tooth complexity in squamates. Nat Commun. 2021;12: 1–13. doi:10.1038/s41467-021-26285-w

111. Angilletta MJ, Steury TD, Sears MW. Temperature, growth rate, and body size in ectotherms: Fitting pieces of a life-history puzzle. Integr Comp Biol. 2004;44: 498–509.

Supporting information

S1 Fig. Protocol for estimating snout-vent length of fossil lizards.

S2 Fig. Mean and maximum body length in fossil lizards by taxonomic group per NALMA. Body length is measured as snout-vent length (SVL) in mm. All measurements were transformed using natural log (LN). Dotted line = maximum. Dashed line = mean. Lines do not connect with the single Duchesnean datapoint since it likely does not accurately represent a maximum or mean value for that NALMA. North American Land Mammal Age (NALMA) abbreviations: Pu = Puercan, To = Torrejonian, Ti = Tiffanian, Cf = Clarkforkian, Wa = Wasatchian, Br = Bridgerian, Ui = Uintan, Du = Duchesnean, Ch = Chadronian, Or = Orellan, Wh = Whitneyan, Ar = Arikareean.

S3 Fig. Mean and maximum body mass in fossil lizards by taxonomic group per NALMA. Mass estimates were calculated from snout-vent length (SVL) using the published equations listed in S8 Table. All measurements (in kg) were transformed using natural log (LN). Dotted line = maximum. Dashed line = mean. Lines do not connect with the single Duchesnean datapoint since it likely does not accurately represent a maximum or mean value for that NALMA. North American Land Mammal Age (NALMA) abbreviations: Pu = Puercan, To = Torrejonian, Ti = Tiffanian, Cf = Clarkforkian, Wa = Wasatchian, Br = Bridgerian, Ui = Uintan, Du = Duchesnean, Ch = Chadronian, Or = Orellan, Wh = Whitneyan, Ar = Arikareean.

S4 Fig. Rarefaction curve showing the number of expected size groups for NALMA sample sizes within the fossil lizard dataset. To conduct this analysis, each fossil specimen in S1

Dataset was assigned to one of 11 size groups, each group representing a bin of 100 mm (e.g., 1-99 mm, 100-199 mm, 200-299 mm, etc.; the last group was ≥ 1 m). S1 Dataset was subsampled here by North American Land Mammal Age (NALMA) interval. The y-axis in this graph shows the total number of size groups recovered in the sample for each NALMA interval. NALMA abbreviations: Pu = Puercan, To = Torrejonian, Ti = Tiffanian, Cf = Clarkforkian, Wa = Wasatchian, Br = Bridgerian, Ui = Uintan, Du = Duchesnean, Ch = Chadronian, Or = Orellan, Wh = Whitneyan, Ar = Arikareean. Individual rarefaction analysis was performed using PAST v4.03 [71].

S5 Fig. Global temperature vs. lizard body size by NALMA. A) Global marine mean annual paleotemperature (MAPT) vs. maximum lizard SVL. Function: LN(Global Marine MAPT) = ((1.23±0.401)*LN(Max Lizard SVL)) + (3.70±0.792), R^2 = 0.04, p(uncorr.) = 0.56, SEE = 0.991. **B)** Global marine MAPT vs. mean lizard SVL. LN(Global Marine MAPT) = ((-0.910±0.303)*LN(Mean Lizard SVL)) + (7.06±0.606), R^2 = 0.0004, p(uncorr.) = 0.95, SEE = 0.812. Temperature data are from Zachos et al. [44] and were taken from the NALMA midpoint in ºC. All data were transformed using natural log. Regression analysis was performed using PAST v4.03 [71].

S1 Table. Anguidae regression equations and tests. Sample sizes indicate number of extant anguid specimens that included the element used in the given regression. Regression equations are ranked in order of increasing standard error of the estimate (SEE). Minimum and maximum SVL estimates for test specimens are based on the standard errors for the slope and intercept of

the given regression. Equations for body mass from individual bones are based on the relevant equation listed in S8 Table.

S2 Table. Varanidae regression equations and tests. Sample sizes indicate number of extant varanid specimens that included the element used in the given regression. Regression equations are ranked in order of increasing standard error of the estimate (SEE). Minimum and maximum SVL estimates for test specimens are based on the standard errors for the slope and intercept of the given regression. Equations for body mass from individual bones are based on the relevant equation listed in S8 Table.

S3 Table. Xenosauridae regression equations and tests. Sample sizes indicate number of extant xenosaurid specimens that included the element used in the given regression. Regression equations are ranked in order of increasing standard error of the estimate (SEE). Minimum and maximum SVL estimates for test specimens are based on the standard errors for the slope and intercept of the given regression. Equations for body mass from individual bones are based on the relevant equation listed in S8 Table.

S4 Table. Teiidae regression equations and tests. Sample sizes indicate number of extant teiid specimens that included the element used in the given regression. Regression equations are ranked in order of increasing standard error of the estimate (SEE). Minimum and maximum SVL estimates for test specimens are based on the standard errors for the slope and intercept of the given regression. Equations for body mass from individual bones are based on the relevant equation listed in S8 Table.

S5 Table. Scincidae regression equations and tests. Sample sizes indicate number of extant scincid specimens that included the element used in the given regression. Regression equations are ranked in order of increasing standard error of the estimate (SEE). Minimum and maximum SVL estimates for test specimens are based on the standard errors for the slope and intercept of the given regression. Equations for body mass from individual bones are based on the relevant equation listed in S8 Table.

S6 Table. Xantusiidae regression equations and tests. Sample sizes indicate number of extant xantusiid specimens that included the element used in the given regression. Regression equations are ranked in order of increasing standard error of the estimate (SEE). Minimum and maximum SVL estimates for test specimens are based on the standard errors for the slope and intercept of the given regression. Equations for body mass from individual bones are based on the relevant equation listed in S8 Table.

S7 Table. Iguania regression equations and tests. Sample sizes indicate number of extant iguanian specimens that included the element used in the given regression. Regression equations are ranked in order of increasing standard error of the estimate (SEE). Minimum and maximum SVL estimates for test specimens are based on the standard errors for the slope and intercept of the given regression. Equations for body mass from individual bones are based on the relevant equation listed in S8 Table.

S8 Table. Published equations for estimating lizard body mass from snout-vent length. These equations were used to estimate mass for fossil lizards as listed in S1 Dataset and to generate equations for estimating body mass from individual bones (S1-S7 Tables).

S9 Table. Tests of general regression equations. Sample sizes indicate number of extant specimens that included the element used in the given regression. Regression equations are ranked in order of increasing standard error of the estimate (SEE). None of these equations passed tests for both homoskedasticity and normal distribution of residuals, thus, none of these equations were used in this study.

S10 Table. Mean and maximum fossil lizard snout-vent length and body mass by taxonomic group per NALMA. These natural log-transformed data were used to generate the graphs in S2 Fig and S3 Fig.

S11 Table. Data for correlating SVL with temperature. Temperature data came from Zachos et al. [44] and were taken from the NALMA midpoint in ºC. The Duchesnean was not included because the maximum SVL estimate for that NALMA was exceptionally low and based on a single data point.

S1 Dataset. Fossil lizard data. Measurements, locality information, and body size estimates for all fossil lizard specimens sampled. Family-specific mass was calculated from equations listed in S8 Table [24,41,42]. General mass estimate (across all lizard groups) was calculated using the embedded equation from Meiri [24].

S2 Dataset. Extant lizard data. Data for all extant lizard specimens measured.

Figure 1

Click here to access/download;Figure;Fig_1.tif

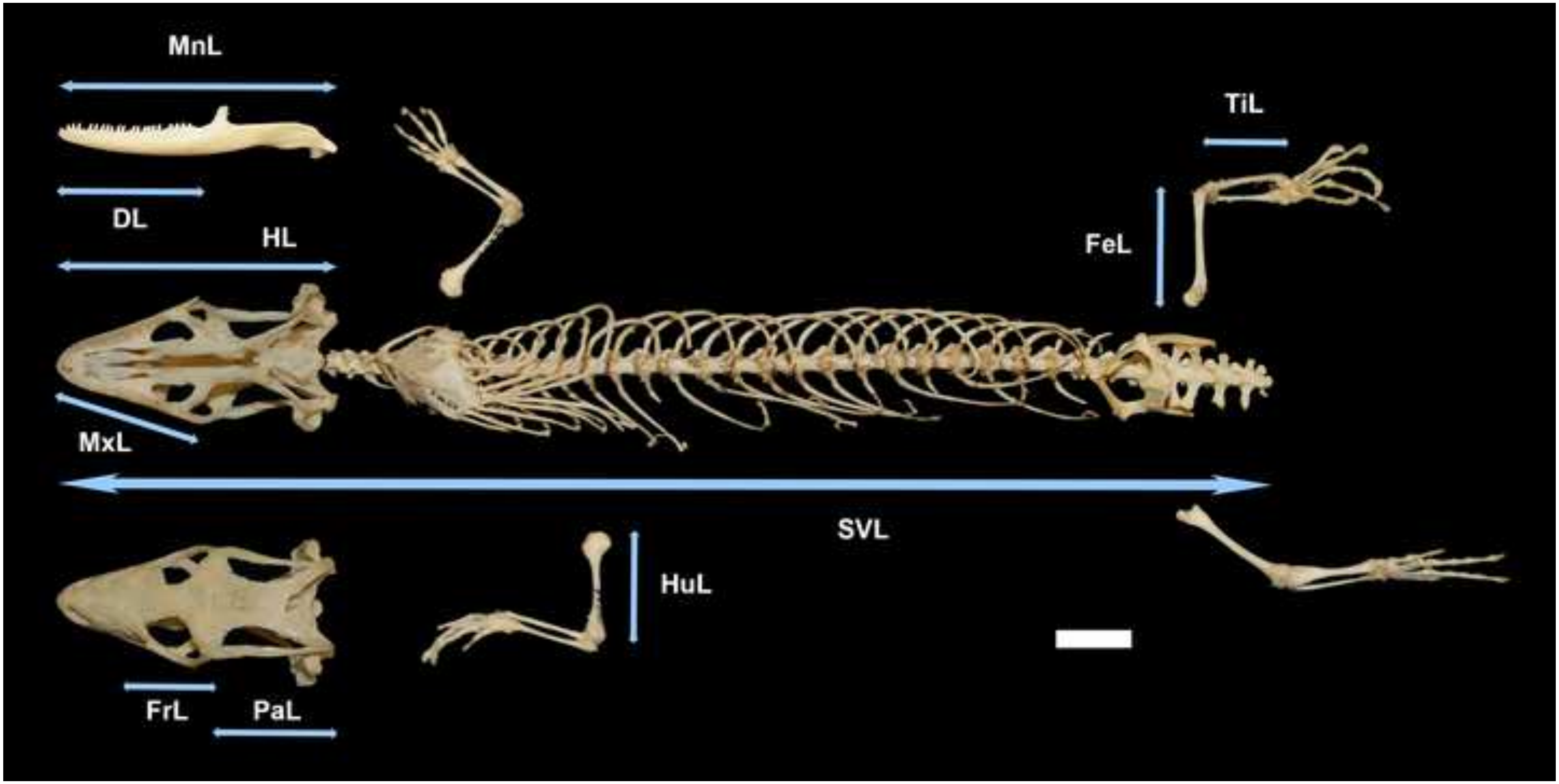

Figure 2

Click here to access/download;Figure;Fig_2.tif

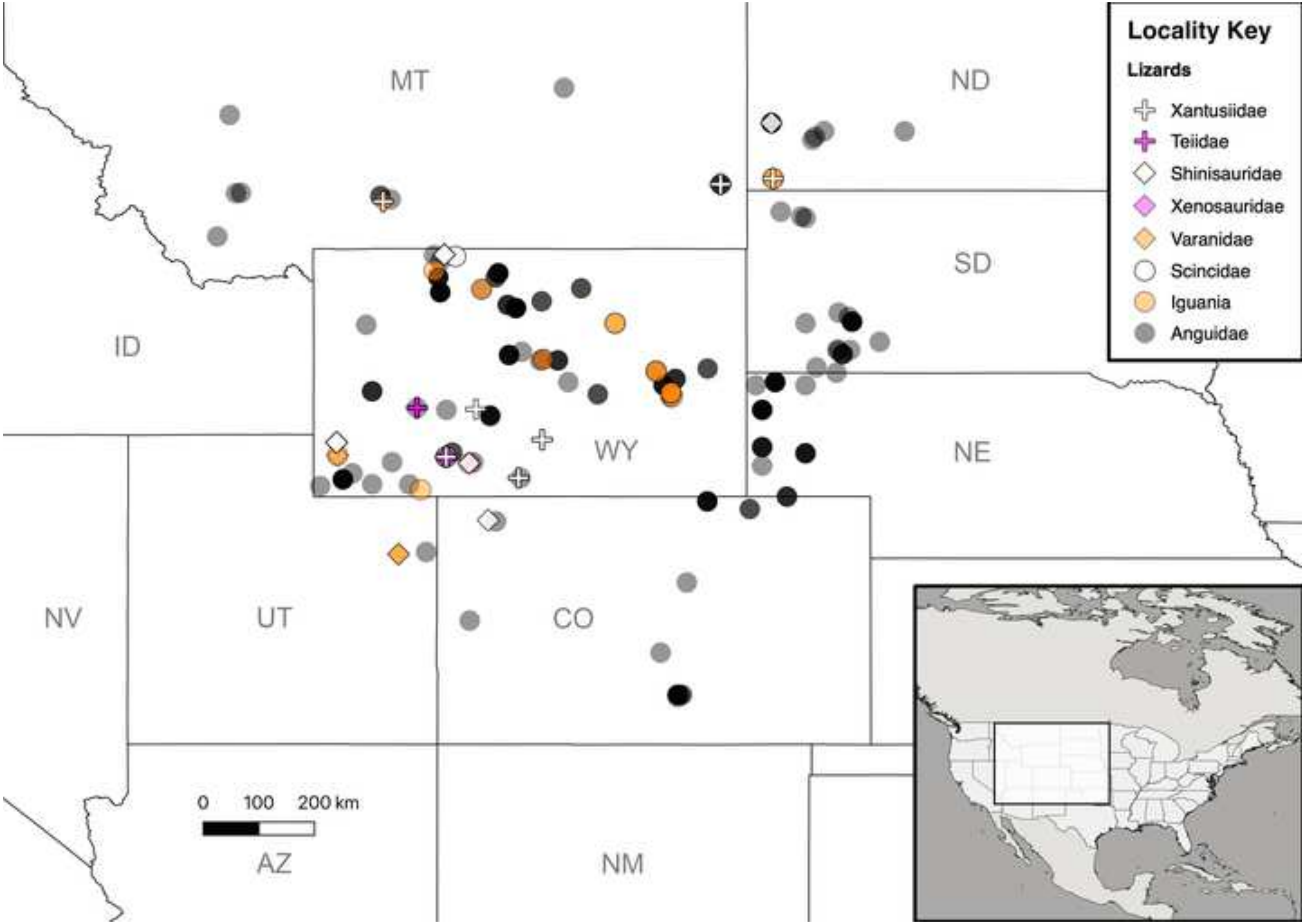

Figure 3

Click here to access/download;Figure;Fig_3.tif

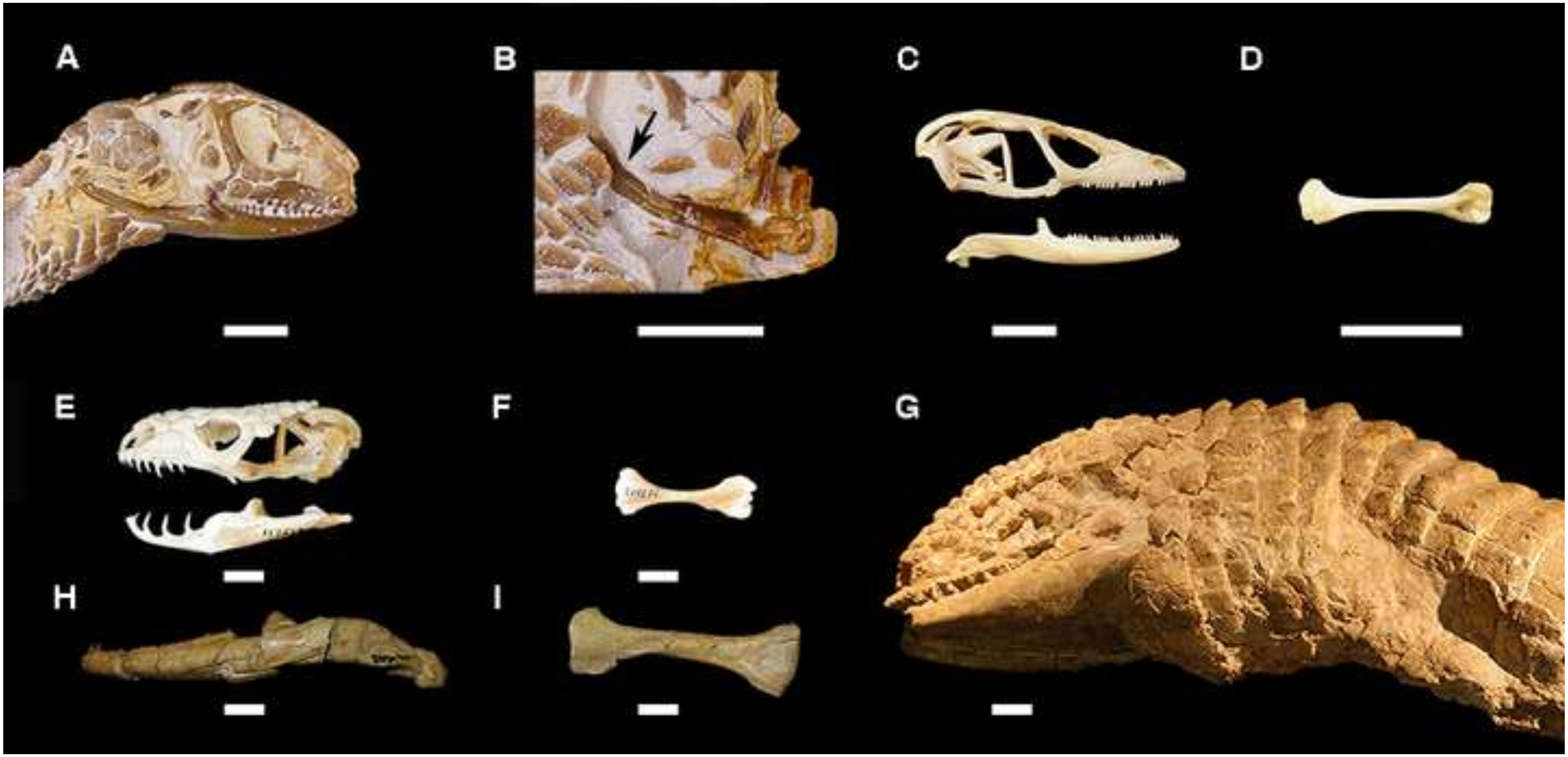

Figure 4

Click here to access/download;Figure;Fig_4.tif

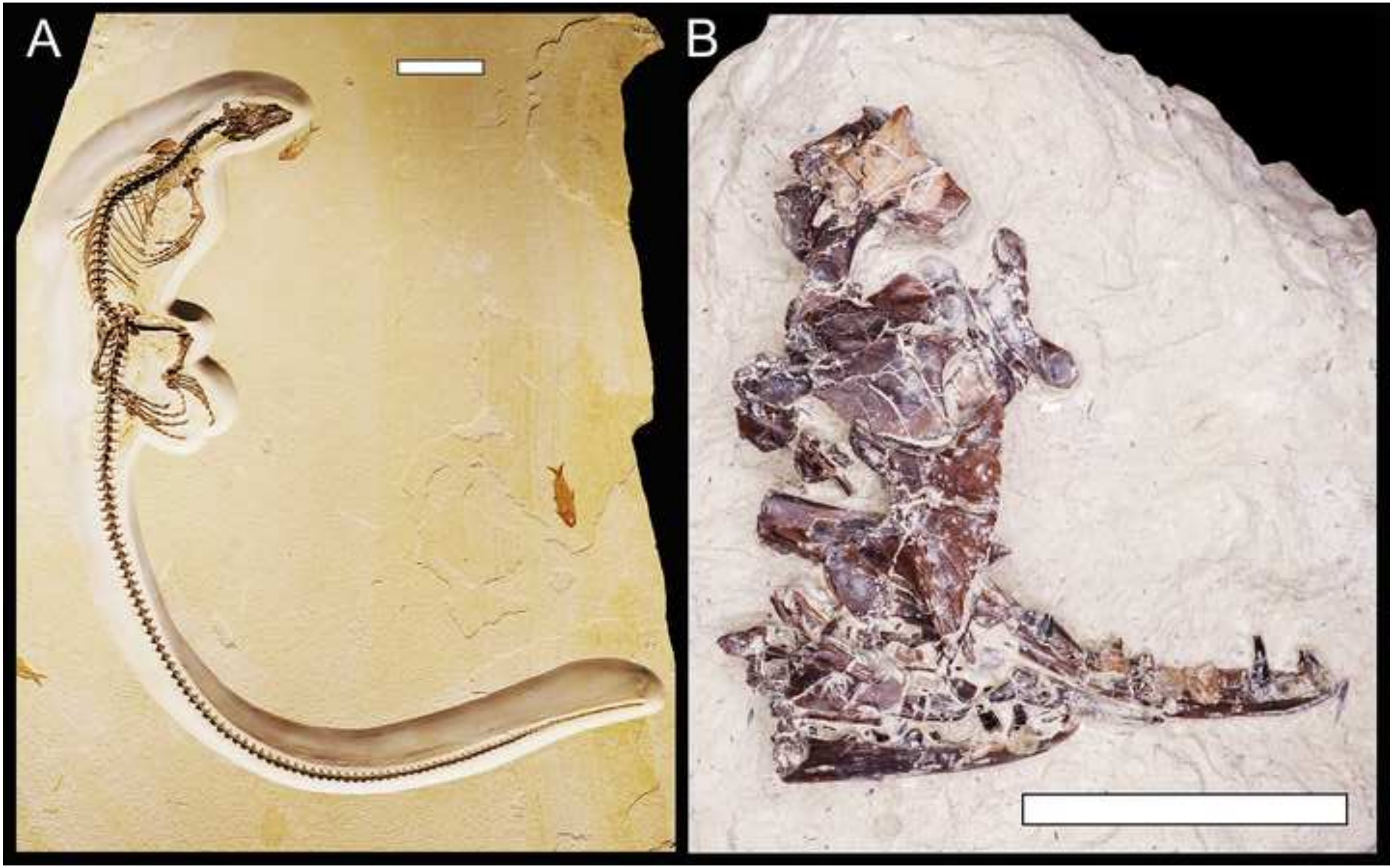

Figure 5

Click here to access/download;Figure;Fig_5.tif

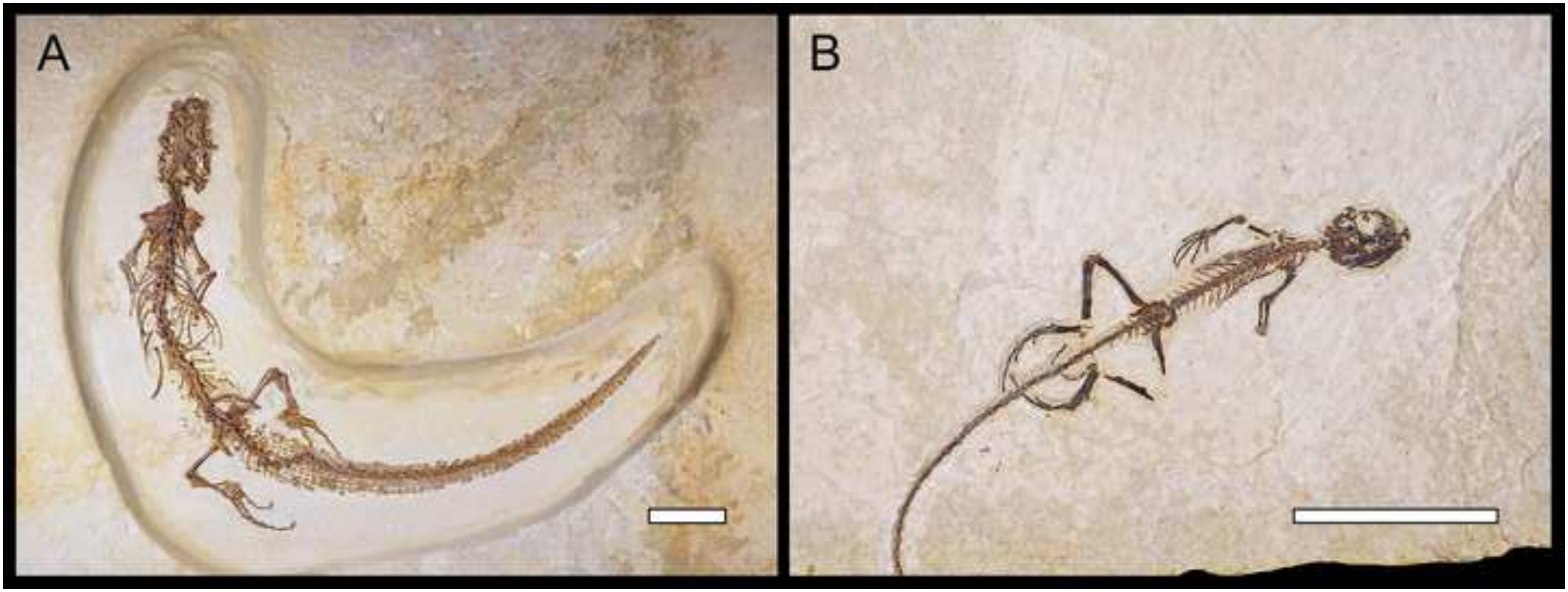

Figure 6

Click here to access/download;Figure;Fig_6.tif

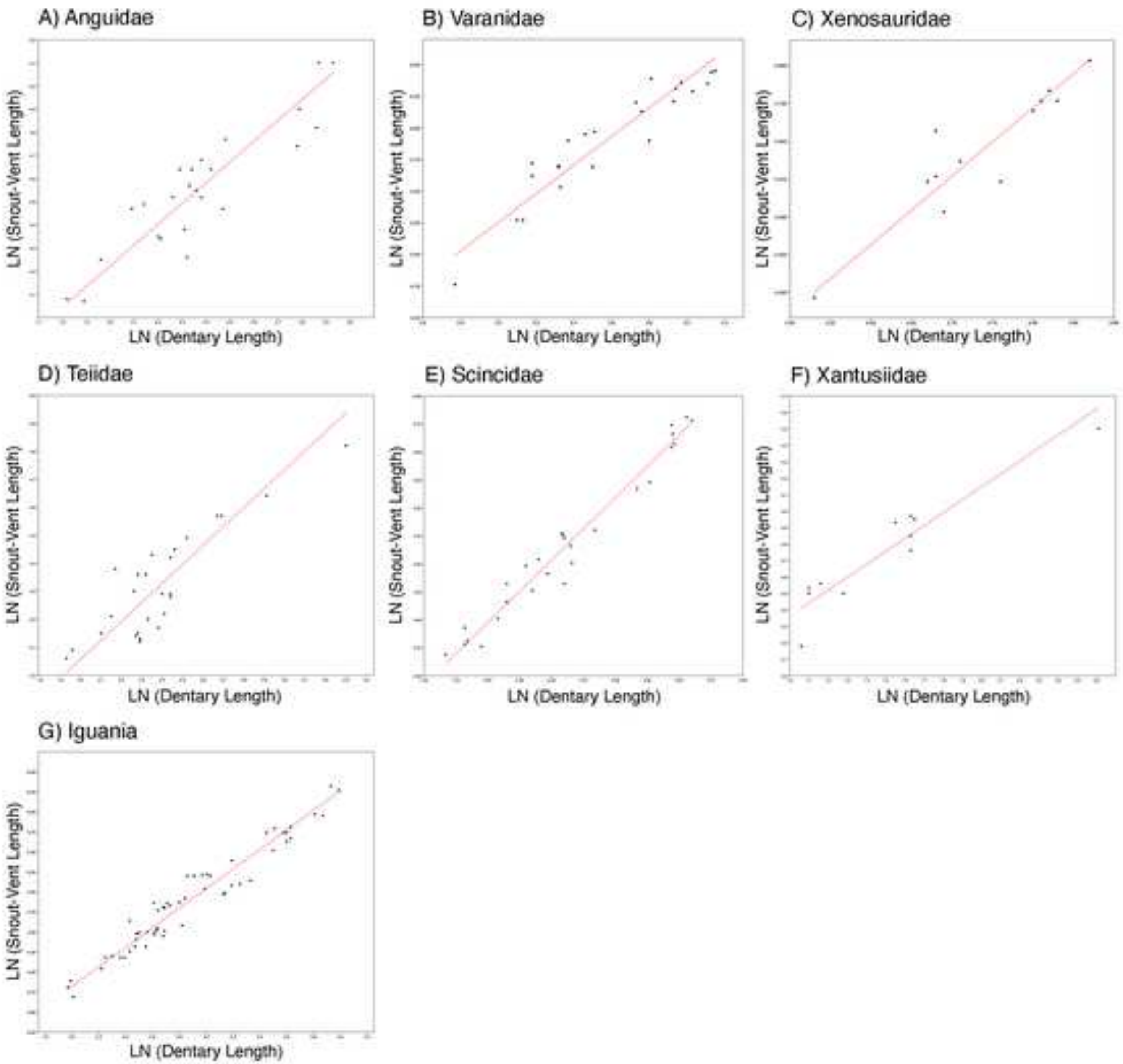

Figure 7

Click here to access/download;Figure;Fig_7.tif

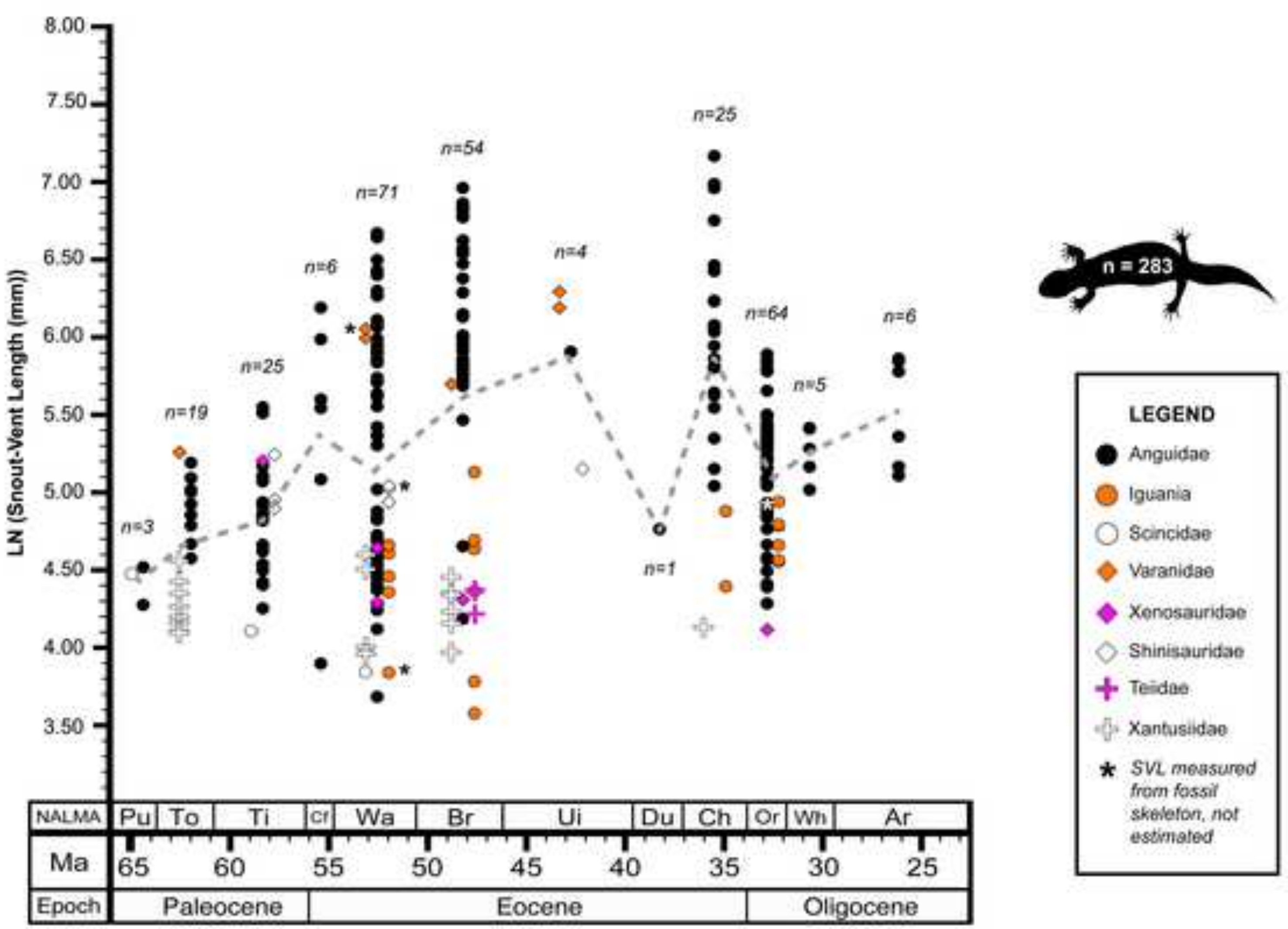

Figure 8

Click here to access/download;Figure;Fig_8.tif

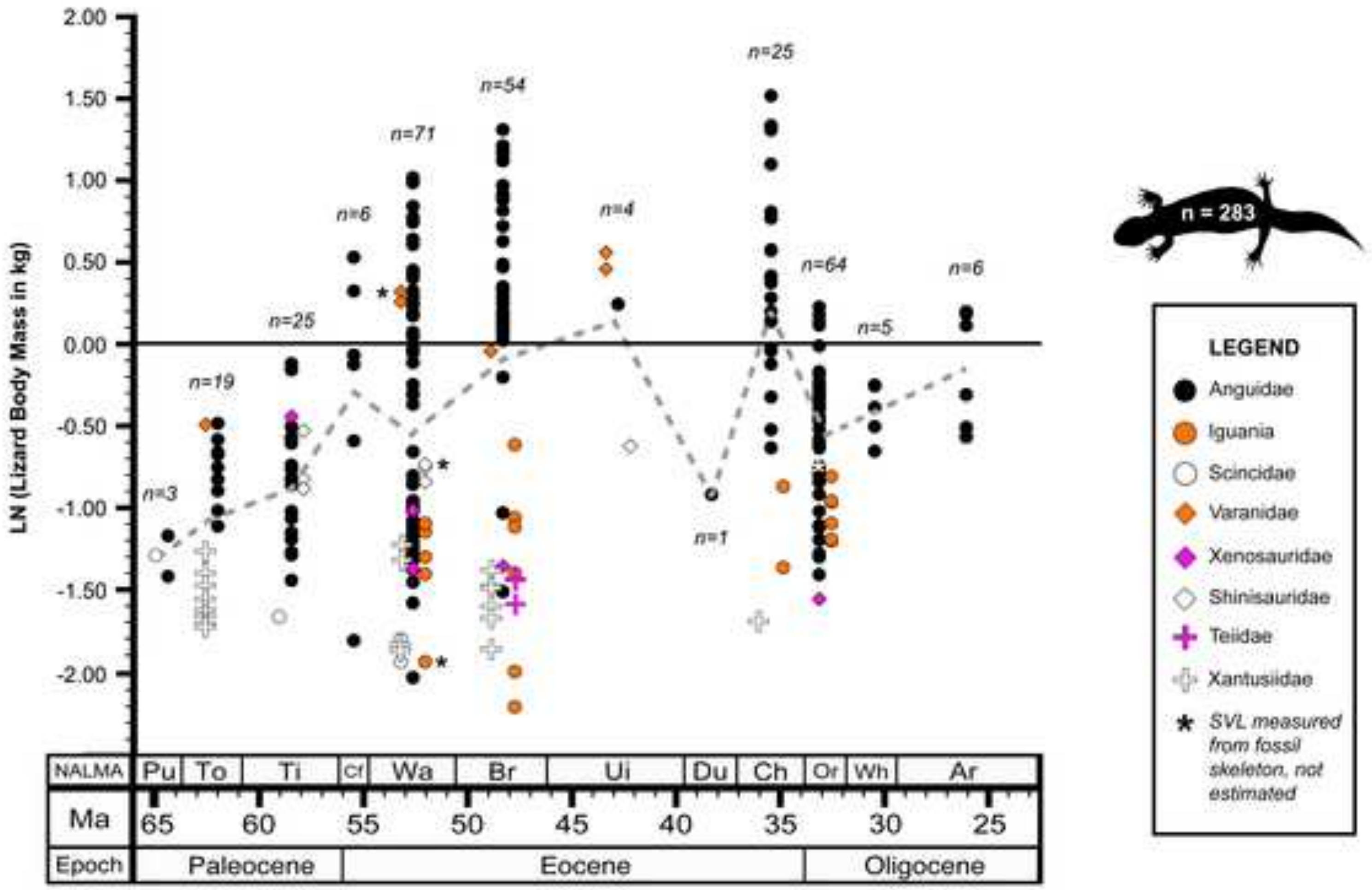

S1 Figure

Click here to access/download
Supporting Information
S1_Fig.tif

S2 Figure

Click here to access/download
Supporting Information
S2_Fig.tif

S3 Figure

Click here to access/download
Supporting Information
S3_Fig.tif

S4 Figure

Click here to access/download
Supporting Information
S4_Fig.tif

S5 Figure

Click here to access/download
Supporting Information
S5_Fig.tif

Supporting Tables

Click here to access/download
Supporting Information
S1-S11_Tables.xlsx

Click here to access/download
Supporting Information
S1-S2_Datasets.xlsx

Body size estimation from isolated fossil bones reveals deep time evolutionary trends ~~for~~ in North American lizards

Sara J. ElShafie

Department of Integrative Biology and Museum of Paleontology, University of California, Berkeley, California, United States of America

E-mail: selshafie@berkeley.edu

Abstract

Lizards play vital roles in extant ecosystems. However, their roles in extinct ecosystems are poorly understood because the fossil record of lizards consists mostly of isolated bones. This makes it difficult to document changes in lizard morphology and body size over time, which is essential for studies of lizard paleoecology and evolution. It is also difficult to compare available fossil lizard data with existing sources of extant lizard data~~sets~~ because extant studies rarely measure individual bones. Furthermore, no previous study has regressed measurements of individual bones to body length across crown lizard groups, nor tested those regressions on fossil skeletons. An extensive dataset of ~~extant lizard~~individual bone measurements from extant lizards~~,~~ across crown taxonomic groups~~,~~ is ~~provided~~ here employed to develop novel ~~. This database is used to derive new~~ methods~~,~~ ~~including individual bones, from which methods~~ for estimating lizard body size from isolated fossil elements~~ were derived~~. These methods were applied to a comparably large dataset of fossil lizard specimens from the robust Paleogene record (66-23 M~~y~~a) of the Western Interior of North America. This study tests the hypothesis that anatomical proportions have been conserved within higher-level crown lizard groups since the Paleogene and can therefore be used to reconstruct snout-vent length (SVL) for fossil specimens referred to the same groups.

Individual bones allowed for predicting SVL of fossil lizard specimens with strong accuracy. ~~Individual bones were regressed to SVL in each extant lizard group and the regressions were tested using measurements from complete extant specimens as well as some rare fossil skeletons. The regressions predicted actual SVL for both the extant and fossil test specimens with similar accuracy.~~ The resulting body size estimates for the entire fossil dataset revealed that lizards reached greatest maximum body size ~~for this study period~~ in the middle Paleogene, with

the largest size class dominated by anguid lizards that exceeded 1 meter in SVL and 1 kg in body mass. Maximum body size decreased to under 400 mm in the late Paleogene. This is the first study to investigate body size evolution across lizard clades over a deep time interval and for a large geographic region. The proposed methods can be used to generate body size regressions and provide estimates of SVL for isolated ~~fossil~~ lizard bones~~s~~ referred to any crown group.

Introduction

Body size influences every aspect of a vertebrate’s physiology and life history [1–4]. It is therefore essential to estimate body size for fossil vertebrate taxa in order to understand the ecological and evolutionary context of any vertebrate group [5–10]. Lizards are a particularly important group of vertebrates to investigate in both extant and extinct ecosystems because they exhibit high taxonomic and morphological diversity and occupy ~~myriad~~ a wide range of ecological roles [4,11–14]. For example, lizards ~~make up the majority of~~contribute significant tetrapod species richness ~~s~~ in some ~~harsh~~ environments, especially deserts; can maintain high population densities in small areas; and have high dispersal abilities [15–19]. Lizards also regulate populations of insects, rodents, and other prey on which they feed, and they are an important food source for many animals [4,12,13,20].

The lizard fossil record consists mostly of isolated elements that can be difficult to identify to the level of genus or even family. Therefore, body size for fossil lizards must be inferred primarily from isolated bones [21]. To further complicate matters, anatomical datasets of extant lizards rarely include measurements of individual cranial or limb bones for comparison. Most relevant previous studies only recorded measurements of head length and snout-vent length (SVL) across extant lizard groups [3,4,11,12,19,22–26]. Hence, no prior study has reconstructed

body size in lizards on evolutionary time scales across higher-level taxonomic groups. This ~~new~~ study ~~aims to~~ ~~addresses this need by~~ presents~~ing~~ an extensive reference dataset of individual anatomical element measurements taken across several crown group lizard lineages and ~~detailing~~ develops methods for using those data to estimate body size in fossil lizards. ~~These methods are used to reconstruct body size in lizard lineages over a deep time interval and across a continental interior.~~

It is not unambiguously clear which variable is the best proxy for body size in fossil vertebrates. Some previous studies estimated mass as a proxy for body size in fossil crocodyliforms using scaling relationships in extant taxa between body mass and femoral length, femoral ~~and~~ circumference [27], and head width [28], since these anatomical measurements are commonly available in the crocodyliform fossil record. Body mass has also been estimated for dinosaurs [7] and fossil mammals [6,29,30] using similar methods.

However, body mass is only weakly correlated with most individual bones in many fossil vertebrates; thus, such methods can be difficult to apply across a fossil record of varied isolated bones [6].

For fossil reptiles, body length is easier to estimate from individual bones than body mass because length correlates tightly with other anatomical measurements. B~~Reptile b~~ody length has been shown to correlate with ~~specific cranial bones (e.g., R^2 = 0.89 for dentary length in acrodontan lizards [9,31]; $R^2 \geq$ 0.78 for various cranial elements in the lizard genera *Anolis* and *Pholidoscelis* [31]), head length (e.g., $R^2 \geq$ 0.97 for extant crocodylians [32]), head width (e.g., $R^2 \geq$ 0.85 for extant crocodylians [28]),~~ femur length (~~e.g., $R^2 \geq$ 0.99 for extant crocodylians~~ [27]), ~~and~~ vertebral element width (~~e.g., $R^2 \geq$ 0.81 for extant boine snakes~~ [31]), head length ([32]), head width ([28]), and specific cranial bones such as dentary length [9,33] in extant

lizards, snakes, and crocodylians ($R^2 \geq 0.78$ in all cited examples). ~~;~~. ~~while M~~m~~M~~andible length has also ~~also~~ been used as a direct proxy for body size in fossil lizards~~, in particular~~ [21,34].

~~Snout-vent length (~~SVL~~)~~, measured from the tip of the snout to the cloacal opening at the base of the tail (~~Fig 1~~Fig 1), is often the preferred measure of body length in extant lizard studies [22,33]. This is because many lizards can autotomize and even regenerate their tails [35–39], thus lizard tail measurements are highly variable. Total body length is also difficult to reconstruct in lizards because the tail ~~rarely~~ does not always possesses its original length in an~~y~~ extant specimen and is often disarticulated in dry skeletonized specimens. Measurements of SVL are more feasible to obtain from wet preserved or dry skeletonized extant lizard specimens. Maximum SVL strongly correlates with mean adult SVL and SVL at sexual maturity in extant lizards [3,40], so maximum SVL is a good metric of mean adult body size for a population of lizards sampled from the fossil record. Furthermore, once SVL is estimated, lizard body mass can be calculated from family-specific SVL-to-mass equations [24,41,42].

Fig 1. Anatomical measurements. Measurements are indicated by arrows. Snout-vent length (SVL) was measured from the tip of the snout at the premaxilla to the posterior edge of the centrum on the second caudal vertebra, which is the approximate position of the middle of the cloaca (vent). Other measurement acronyms: DL = dentary length, FeL = femur length, FrL = frontal length, HL = head length, HuL = humerus length, MnL = mandible length, MxL = maxilla length, PaL = parietal length, TiL = tibia length. Scale bar = 1 cm. Specimen pictured is *Gerrhonotus infernalis* (Anguidae; FMNH 22452).

Formatted: Indent: First line: 0"

Formatted: Indent: First line: 0.5"

~~**Fig 1. Measurement of snout-vent length (SVL).** SVL was measured from the tip of the snout at the premaxilla to the posterior edge of the centrum on the second caudal vertebra, which is the approximate position of the middle of the cloaca (vent). SVL measurement is indicated by the blue arrow. Scale bar = 1 cm. Specimen pictured is *Gerrhonotus infernalis* (Anguidae; FMNH 22452).~~

For this study, lizards were sampled from the Paleogene record (~~23~~ 66-23 M~~y~~a) in the Western Interior of North America, specifically, from localities concentrated in the United States (~~Fig 2~~Fig 2). The Paleogene is an interesting period to study because it spans significant warming and cooling events [43,44] and chronicles the radiations of both mammals [45–55] and later squamates [56–60] following the End-Cretaceous Mass Extinction. This timeframe was used here as a study system because it has a prolonged and robust fossil record in the Western Interior, preserving many diverse and ecologically important extant lizard clades that still occur in the area. These include anguids and pleurodontan iguanians (e.g., iguanids and dactyloids), as well as clades that were extirpated from the region during the Paleogene, such as varanids and shinisaurids. All the sampled Paleogene specimens, many identified to the level of genus or even species, were stored in n~~N~~atural history museum collections in the United States. ~~include many Paleogene lizard specimens identified to the level of family or even genus.~~

Formatted: Indent: First line: 0"

~~**Fig 2. Map showing all localities for fossil lizard data from across the Western Interior of North America through the Paleogene.** Featured area is highlighted in inset map. Data points represent only specimens for which the locality could be georeferenced. Some taxonomic groups occurring with others in a particular locality may not be visible. Base maps were made with~~

~~Natural Earth and are reprinted from Natural Earth under a CC Public Domain license, original copyright 2009. All other data contained were collected by the author. Maps were generated using QGIS-LTR 3.22.6 (download.qgis.org). The figure created is under the CC-BY 4.0 license, as determined by the authors of QGIS.~~

Formatted: Indent: First line: 0.5"

Fig 2. Map showing all localities for fossil lizard data from across the Western Interior of North America through the Paleogene. Featured area is highlighted in inset map. Data points represent only specimens for which the locality could be georeferenced. Some taxonomic groups occurring with others in a particular locality may not be visible. Base maps were made with Natural Earth and are reprinted from Natural Earth under a CC Public Domain license, original copyright 2009. All other data contained were collected by the author. Maps were generated using QGIS-LTR 3.22.6 (download.qgis.org). The figure created is under the CC-BY 4.0 license, as determined by the authors of QGIS.

Formatted: Indent: First line: 0"

This study tested the hypothesis that proportions of individual bone lengths to SVL are conserved within each of eight crown group lizard lineages (exclusive of snakes) represented in the Paleogene fossil record of the Western Interior, and that these proportions can therefore be used to predict SVLs ~~within crown squamate groups (exclusive of snakes)~~ for fossil lizard specimens~~ identified to those same groups~~. ~~This hypothesis was tested using eight taxonomic lizard groups (seven families + Iguania – the latter encompassing the iguanian families, not all of which have clear taxonomic phylogenetic relationships [61,62]) that are represented in the Paleogene fossil record and still exist today.~~ For each crown group ~~represented~~investigated,

~~regressions of~~ individual cranial and limb bones were regressed onto SVL ~~were generated~~, and the resulting equations were validated using~~.~~ ~~Both~~ complete skeletons of both extant and fossil lizards ~~skeletons were used as test specimens to validate the regressions~~. The equations were then ~~used~~ applied to reconstruct body size for crown group lizards sampled from across the U.S. Western Interior through the Paleogene. ~~This is the first study to reconstruct lizard body size evolution in different crown groups over a prolonged geologic interval and across a continental interior.~~ This study ~~, using an extensive dataset,~~ provides the first opportunity to investigate modes of body size evolution ~~and resource zone partitioning in~~ across lizard diversity~~s~~ over a prolonged geologic interval and spanning a continental interior.e ~~results have broad implications for modes of body size evolution and resource zone partitioning in lizards. In addition to applications for fossil research, the extensive dataset and body size estimation methods presented here can be of great use to functional morphologists and herpetologists.~~

Formatted: Not Strikethrough

Institutional abbreviations

AMNH, American Museum of Natural History, New York, NY; **CAS**, California Academy of Sciences, San Francisco, CA; **CJB**, Christopher J. Bell, University of Texas at Austin, Austin, TX; **CM**, Carnegie Museum of Natural History, Pittsburgh, PA; **DMNH**, Denver Museum of Natural History (Denver Museum of Nature and Science), Denver, CO; **FLMNH**, Florida Museum of Natural History, Gainesville, FL; **FMNH**, Field Museum of Natural History, Chicago, IL; **FOBU**, Fossil Butte National Monument, Kemmerer, WY; **GCVP**, Georgia College Vertebrate Paleontology, Milledgeville, GA; **LACM**, Natural History Museum of Los Angeles County, Los Angeles, CA; **MCZ**, Museum of Comparative Zoology, Cambridge, MA; **MSU**, Michigan State University, Lansing, MI; **MVZ**, Museum of Vertebrate Zoology,

Berkeley, CA; **NMMNH**, New Mexico Museum of Natural History, Albuquerque, NM; **PTRM**, Pioneer Trails Regional Museum, Bowman, ND; **RAM**, Raymond M. Alfe Museum of Paleontology, Claremont, CA; **SMM**, Science Museum of Minnesota, Saint Paul, MN; **TxVP**, Texas Vertebrate Paleontology Collection, The University of Texas, Austin, TX; **UCM**, University of Colorado Museum, Boulder, CO; **UCMP**, University of California Museum of Paleontology, Berkeley, CA; **UMMP**, University of Michigan Museum of Paleontology, Ann Arbor, MI; **UNSM**, University of Nebraska State Museum, Lincoln, NE; **USNM**, United States National Museum (Smithsonian National Museum of Natural History), Washington, DC; **UW**, University of Wyoming Geological Museum, Laramie, WY; **UWBM**, University of Washington Burke Museum, Seattle, WA; **YPM**, Yale Peabody Museum, New Haven, CT.

Materials & methods

Data collection

The data for this study were amassed from 26 different natural history museum collections across the United States (see “Institutional Abbreviations”). These include a~~A~~natomical measurements ~~were~~ collected from a total of 283 fossil lizard specimens, ~~including~~ with 44 recognized genera and at least 33 recognized species, from the Paleogene record of the U.S. Western Interior (~~Table 1~~Table 1, S1 Dataset). Only fossil lizard specimens with complete cranial bones or associated limb bones were sampled (e.g., Fig 3), for the following reasons: 1) the lizard fossil record is mostly comprised of cranial bones and, fortunately, most extant phylogenetic morphological characters for lizards are found in cranial bones [61,62], making it possible to determine at least coarse taxonomic identifications (family or higher) for fossil lizard specimens from cranial material [62]; 2) historical fossil identification may lack fidelity at the

genus or species level [63–65]; 3) cranial and limb bones can be used to estimate SVL in lizards [9,33]. Not all specimens that were attributed to a genus could be attributed to a specific species. Only specimens that included complete cranial or limb elements (e.g., Fig 3) were sampled. A few measured specimens included complete skulls (Fig 3Fig 3A) or even skeletons (Figs 4 and 5). All The fossil specimens were grouped by North American Land Mammal Age (NALMA), which compose a relative chronology used to divide the Paleogene based on taxonomic turnover in the mammal fossil record [63]. Currently recognized aAge ranges for these intervals are listed in Table 2 based on Barnosky et al. [64]. fossil specimens were referred to crown groups (family-level or higher) based on specimen label identifications as well as taxonomic information from the Paleobiology Database [66] and current literature.

Formatted: Indent: First line: 0.5"

Table 1. Summary of datasets. The extant species totals include subspecies. Fossil species totals do not include specimens that could not be identified to a specific genus or species (e.g., Anguidae indet., *Glyptosaurus sp.*). For example, all three fossil teiid specimens sampled were identified as only “Teiidae indet.”

**Taxonomic group**				**# Extant specimens sampled**	**# Extant genera sampled**	**# Extant species sampled**
**Anguidae**				66	4	17
**Varanidae**				47	1	17
**Teiidae**				33	5	11
**Scincidae**				28	8	19

~~**Xantusiidae**~~				~~20~~	~~1~~	~~3~~
~~**Xenosauridae**~~				~~17~~	~~1~~	~~3~~
~~**Iguania**~~				~~55~~	~~9~~	~~16~~
~~**Shinisauridae**~~				~~N/A~~	~~N/A~~	~~N/A~~
~~**Total**~~				~~**266**~~	~~**29**~~	~~**86**~~

Table 1. Summary of datasets. The extant species totals include subspecies. Fossil species totals do not include specimens that could not be identified to a specific genus or species (e.g., Anguidae indet.). For example, all three fossil teiid specimens sampled were identified as only "Teiidae indet."

**Taxonomic group**	**# Fossil specimens sampled**	**# Fossil genera sampled**	**# Fossil species sampled**	**# Extant specimens sampled**	**# Extant genera sampled**	**# Extant species sampled**
**Anguidae**	218	16	17	66	4	17
**Varanidae**	6	1	1	47	1	17
**Teiidae**	3	0	0	33	5	11
**Scincidae**	4	3	1	28	8	19
**Xantusiidae**	19	2	2	20	1	3
**Xenosauridae**	7	4	3	17	1	3
**Iguania**	19	8	6	55	9	16
**Shinisauridae**	7	3	3	N/A	N/A	N/A
**Total**	**283**	**37**	**33**	**266**	**29**	**86**

Fig 3. Paleogene anguimorph lizards from the Western Interior of North America and extant morphological analogues. (A) *Peltosaurus granulosus*† (Anguidae) skull in right lateral view, and B) right humerus in medial view (AMNH 42913), indicated by arrow, Orellan (33.9-31.8 Ma). (C) *Gerrhonotus infernalis* (Anguidae) skull and right mandible in right lateral view and (D) left humerus in medial view (FMNH 22452), extant. A-D are oriented with anterior to the right. (E) *Heloderma suspectum* (Helodermatidae) skull and left mandible in left lateral view, and (F) right humerus in medial view (UCMP 117512), extant. (G) *Helodermoides tuberculatus*† skull, mandibles, and cervical osteoderms in right lateral view, reflected for continuity (USNM V 13869), Chadronian (37.0-33.9 Ma). (H) *Helodermoides tuberculatus*† (Anguidae) left mandible in lateral view, and (I) right humerus in medial view, reflected for continuity (UNSM 4511), Chadronian. E-I are oriented with anterior to the left. Scale bar = 1 cm. USNM V 13869 (G) image courtesy of the Smithsonian Institution.

Formatted: Font:

Fig 4. Paleogene varanids from the Western Interior of North America. (A) *Saniwa ensidens*† skeleton (FMNH PR 2378), Wasatchian (54.9-50.5 Ma). Scale bar = 10 cm. (B) *Saniwa sp.*† dentaries, vertebrae, and fragments (DMNH EPV.34588), Bridgerian (50.5-46.2 Ma). Scale bar = 3 cm.

Fig 5. Other complete Paleogene lizard skeletons from the Western Interior of North America. (A) *Bahndwivici ammoskius*† (Shinisauridae) skeleton (FMNH PR 2260), Wasatchian (54.9-50.5 Ma). Scale bar = 3 cm. (B) *Afairiguana avius*† (Iguania; Polychrotidae) skeleton (FMNH PR 2379), Wasatchian. Scale bar = 3 cm.

Fig 3. Paleogene anguimorph lizards from the Western Interior of North America and extant morphological analogues. (A) *Peltosaurus granulosus*‡ (Anguidae) skull in right lateral view, and B) right humerus in medial view (AMNH 42913), indicated by arrow, Orellan. (C) *Gerrhonotus infernalis* (Anguidae) skull and right mandible in right lateral view and (D) left humerus in medial view (FMNH 22452), extant. A-D are oriented with anterior to the right. (E) *Heloderma suspectum* (Helodermatidae) skull and left mandible in left lateral view, and (F) right humerus in medial view (UCMP 117512), extant. (G) *Helodermoides tuberculatus*‡ skull, mandibles, and cervical osteoderms in right lateral view, reflected for continuity (USNM V 13869), Chadronian. (H) *Helodermoides tuberculatus*‡ (Anguidae) left mandible in lateral view, and (I) right humerus in medial view, reflected for continuity (UNSM 4511), Chadronian. E-I are oriented with anterior to the left. Scale bar = 1 cm. USNM V 13869 (G) image courtesy of the Smithsonian Institution.

Fig 4. Paleogene varanids from the Western Interior of North America. (A) *Saniwa ensidens*‡ skeleton (FMNH PR 2378), Wasatchian. Scale bar = 10 cm. (B) *Saniwa sp.*‡ dentaries, vertebrae, and fragments (DMNH EPV.34588), Bridgerian. Scale bar = 3 cm.

Fig 5. Other complete Paleogene lizard skeletons from the Western Interior of North America. (A) *Bahndwivici ammoskius*‡ (Shinisauridae) skeleton (FMNH PR 2260), Wasatchian. Scale bar = 3 cm. (B) *Afairiguana avius*‡ (Iguania; Polychrotidae) skeleton (FMNH PR 2379), Wasatchian. Scale bar = 3 cm.

To compare the fossil lizard specimens with extant congeners, 266 extant specimens were also measured (S2 Dataset), comprising 30 genera and 86 species (Table 1) from the same crown group lineages represented in the fossil dataset. These included seven extant families: Anguidae (e.g., alligator lizards and galliwasps), Scincidae (skinks), Shinisauridae (crocodile lizards), Teiidae (e.g., whip-tail lizards), Varanidae (monitor lizards), Xantusiidae (night lizards), and Xenosauridae (knob-scaled lizards). Legless anguids and scincids were omitted because proportions of cranial elements to SVL differ between limbed taxa and serpentiform taxa in extant squamate groups [21] and because all the extinct taxa in the fossil dataset presented here are inferred to have had limbs based on associated limb material or assignment to extant groups with limbs. The pleurodontan iguanian families represented in the fossil dataset (e.g., Iguanidae and Polychrotidae (Fig 5B)) were treated as a collective group, “Iguania,” in both datasets. Phylogenetic relationships among iguanian families are poorly resolved, but the group “Iguania” is supported by both morphological and molecular analyses [67–70]. In order to compare individual cranial and limb measurements with SVL measurements, extant data collection in this study focused on dry skeletonized specimens with reasonably complete skulls and vertebral series (Fig 1, S2 Dataset). A total sample size of at least n = 17 was collected for each of the eight major taxonomic groups (it was difficult to find complete specimens of Xenosauridae, so that sample size was the lowest), with 1-21 individuals sampled per species.

Extant species were selected for sampling to encompass the full range of extant body size diversity within a given lineage as much as possible, with the exception of extreme outliers. However, some outlier specimens were used to further test the predictive power of regressions for groups that included exceptionally large individuals in the fossil dataset, Anguidae and Varanidae.

All measurements were taken with digital calipers (Mitutoyo 150 mm) to the nearest 0.1 mm for individual bones and the nearest 1 mm for SVL. For specimens with an SVL measuring > 150 mm, or for specimens that were preserved in a curved position, a tape measure was used to measure SVL to the nearest 1 mm. SVL was measured on skeletons as the length from the tip of the snout to the posterior centrum of the second caudal vertebra, which is the approximate position of the middle of the cloaca (vent; see Fig 1). The position of the cloaca on skeletonized specimens was determined by aligning the pelvis of a wet specimen with that of a dry skeletonized congener specimen of comparable size. Whenever a dried skin was included with a skeletonized specimen, it was used to verify the SVL measurement from the vertebral series. Skull length was measured as the length from the tip of the snout to the most posterior point of the skull (either the occipital condyle or the posterior tip of the supratemporal process; Fig 1). If a cranial bone had bilateral asymmetry (e.g., if on a parietal, one supratemporal process was longer than the other), the longer side was taken to represent the maximum measurement. Each cranial bone was measured from the most anterior to the most posterior point (e.g., on the dentary, from the anterior tip of the ramus to the posterior tip of the retroarticular process). For long bones, only complete bones with epiphyses included were measured.

When it was not possible to access a fossil specimen in person (which was the case for the UMMP specimens and a few specimens measured from figures in literature), measurements were taken from digital photographs with a scale bar in standard orientation using the open access software ImageJ (available online at: https://imagej.nih.gov/ij/download.html). Measurements taken from photographs are indicated in the "Notes" column in S1 Dataset.

~~**Table 2. Lizard crown group diversity by NALMA interval.** Expected diversity based on the Paleobiology Database (PBDB; www.paleobiodb.org) is provided for comparison but should be~~

taken as a coarse estimate because there may be some identification error in PBDB entries. Entries that were deemed inaccurate based on literature review were disregarded. Some data are also missing from the PBDB (e.g., no specific varanid genera or species are listed for the Paleogene within the United States). Iguanian specimens are identified by specific family rather than as "Iguania" in the PBDB, so comparison for that group was not possible. This table does not represent the full taxonomic diversity of Paleogene lizards represented in the U.S. Western Interior fossil record; it only represents groups for which complete fossil cranial or limb bones were measured. Fossil genera and species totals per family per NALMA do not include specimens that could not be identified to a specific genus or species, respectively (e.g., Anguidae indet.; *Glyptosaurus* sp.). Hence, some genera totals are zero. Specimens tentatively referred to a specific genus or species (e.g., *c.f. Glyptosaurus*) are counted toward that genus and/or species. NALMA age ranges are from Barnosky et al. [72].

**Epoch**	**NALMA (Mya)**	**Lizard crown groups sampled**	**# of recognized genera sampled / expected from PBDB**	**# of recognized species sampled / expected from PBDB**
Paleocene	Puercan (66.0 – 63.8)	Anguidae	1 / 2	1 / 2
		Scincidae	1 / 0	1 / 0
	Torrejonian (63.8 – 60.9)	Anguidae	2 / 4	2 / 5
		Varanidae	1 / 0	0 / 0
		Xantusiidae	1 / 1	1 / 1
	Tiffanian (60.9 – 56.2)	Anguidae	3 / 4	3 / 5
		Scincidae	1 / 0	0 / 0
		Shinisauridae	1 / 0	1 / 0

		Xenosauridae	1 / 0	1 / 0
	Clarkforkian (56.2 – 54.9)	Anguidae	3 / 0	3 / 0
Eocene	Wasatchian (54.9 – 50.5)	Anguidae	10 / 10	8 / 14
		Iguania	4 / 0	3 / 0
		Scincidae	1 / 0	0 / 0
		Shinisauridae	2 / 1	2 / 1
		Varanidae	1 / 0	1 / 0
		Xantusiidae	1 / 2	0 / 4
		Xenosauridae	2 / 0	1 / 0
	Bridgerian (50.5 – 46.2)	Anguidae	4 / 8	2 / 7
		Iguania	1 / 0	0 / 0
		Teiidae	0 / 0	0 / 0
		Varanidae	1 / 0	0 / 0
		Xantusiidae	2 / 1	1 / 1
		Xenosauridae	1 / 0	0 / 0
	Uintan (46.2 – 39.7)	Anguidae	1 / 4	0 / 6
		Shinisauridae	1 / 0	0 / 0
		Varanidae	1 / 0	1 / 0
	Duchesnean (39.7 – 37.0)	Anguidae	0 / 0	0 / 0
	Chadronian (37.0 – 33.9)	Anguidae	3 / 2	3 / 2
		Iguania	2 / 0	2 / 0

Formatted: English (United States)
Formatted: English (United States)
Formatted: English (United States)
Formatted: English (United States)
Formatted: English (United States)
Formatted: English (United States)
Formatted: English (United States)
Formatted: English (United States)
Formatted: English (United States)
Formatted: English (United States)
Formatted: English (United States)
Formatted: English (United States)
Formatted: English (United States)
Formatted: English (United States)
Formatted: English (United States)
Formatted: English (United States)
Formatted: English (United States)
Formatted: English (United States)
Formatted: English (United States)
Formatted: English (United States)
Formatted: English (United States)
Formatted: English (United States)

		Xantusiidae	1 / 0	1 / 0
Oligocene	Orellan (33.9 - 31.8)	Anguidae	2 / 1	2 / 1
		Iguania	1 / 0	1 / 0
		Xenosauridae	1 / 1	1 / 1
	Whitneyan (31.8 - 29.5)	Anguidae	1 / 0	1 / 0
	Arikareean (29.5 - 23.0)	Anguidae	1 / 1	1 / 1

To compare the fossil lizard specimens with extant congeners, 266 extant specimens were measured (S2 Dataset), comprising 30 genera and 86 species (Table 1) from the same crown group lineages that were represented in the fossil dataset. These included seven extant families: Anguidae (e.g., alligator lizards and galliwasps), Scincidae (skinks), Shinisauridae (crocodile lizards), Teiidae (e.g., whip-tail lizards), Varanidae (monitor lizards), Xantusiidae (night lizards), and Xenosauridae (knob-scaled lizards). There were also several pleurodontan iguanian families represented in the fossil dataset (e.g., Iguanidae and Polychrotidae), so these were treated as a collective group, “Iguania,” in both datasets. Phylogenetic relationships among iguanian families are poorly resolved, but the group “Iguania” is supported by both morphological and molecular analyses [61,62,65,66]. Extant data collection in this study focused on dry skeletonized specimens to obtain measurements of individual bones as well as SVL from the same specimens. Juvenile specimens were excluded from the extant dataset. Further details on data collection procedures and considerations are provided in Supporting Information.

No permits were required for the described study, which complied with all relevant regulations.

Generation~~ng~~ and testing of regressions for body length

~~Researchers commonly use bivariate linear regressions to estimate SVL for fossil vertebrates using anatomical measurements from specimens of the most inclusive phylogenetic group [72].~~ This study regressed anatomical elements (the independent variables) – including ~~skull~~ head length, individual cranial bone length (dentary, mandible, maxilla, frontal, parietal), and limb bone length (humerus, femur, tibia) – to SVL for ~~the~~ each extant lizard group~~s~~ in the dataset presented (~~Table 3, S1~~S1 Fig,~~ S2 Fig,~~ S1-S7 Tables). Regressions were only generated for the anatomical elements available for each lizard group in the fossil dataset (S1-S7 Tables). For example, no complete and isolated fossil varanid femora were found in collections, so a regression for varanid femur length to SVL was not generated (S2 Table).

Reduced major axis (RMA) bivariate linear regressions were used because ~~RMA~~ they accommodate~~s~~ error in both the dependent and independent variable measurements [71]. Before the analyses, a~~A~~ll the measurements were transformed using the natural log (LN). This method ~~,~~ ~~which~~ is commonly applied in analyses of fossil vertebrate body size, especially to minimize the effects that any outliers might have on regression coefficients [7,9,29,49,52,53,72–75]. Tests of natural log compared to base 10 log regressions also produced the same SVL predictions for large test specimens in this study, so natural log was used in all regressions for consistency. All regression analyses were performed using PAST v.4.03 [71].

Formatted: Indent: First line: 0.5"

Table 3. Regression functions and associated statistics for SVL estimation from individual bones. All functions were transformed using natural log (LN). Standard error for slope and intercept terms are listed after the slope and intercept in the equations. SEE = Standard error of the estimate. Functions are listed in order of increasing SEE for each group. P(uncorrelated) < 0.001 for all functions.

**Group**	**Predictor**	***n***	**Slope *b***	**Standard error of *b***	**Intercept**	**Standard error of intercept**	***R*²**	**SEE**
Anguidae	Skull Length	46	0.946	0.0417	1.71	0.129	0.91	0.0740
	Femur Length	43	0.888	0.0410	2.46	0.101	0.91	0.0742
	Mandible Length	43	0.921	0.0424	1.82	0.129	0.91	0.0760
	Humerus Length	41	0.889	0.0451	2.57	0.106	0.90	0.0815
	Parietal Length	24	1.03	0.0658	2.29	0.148	0.91	0.0853
	Maxilla Length	22	1.02	0.0803	2.40	0.170	0.88	0.0992
	Frontal Length	22	1.27	0.111	2.13	0.216	0.85	0.120
	Dentary Length	24	0.898	0.0816	2.43	0.195	0.82	0.121
Varanidae	Tibia Length	27	0.953	0.0282	2.43	0.103	0.98	0.0695
	Maxilla Length	25	1.13	0.0423	2.04	0.145	0.97	0.0837
	Dentary Length	23	1.13	0.0845	1.86	0.305	0.88	0.157
Xenosauridae	Dentary Length	12	0.36	0.164	0.921	0.450	0.85	0.0530
	Parietal Length	12	1.03	0.154	2.17	0.369	0.78	0.0661
	Frontal Length	12	1.05	0.185	2.16	0.435	0.69	0.0787
Teiidae	Dentary Length	26	0.677	0.0686	2.70	0.165	0.75	0.0995
Scincidae	Maxilla Length	27	1.06	0.0352	2.20	0.0850	0.97	0.115
	Dentary Length	27	1.12	0.0426	1.74	0.115	0.96	0.133

Xantusiidae	Maxilla Length	20	0.698	0.0659	2.82	0.0944	0.88	0.119
	Dentary Length	20	0.787	0.0869	2.58	0.134	0.89	0.124
Iguania	Skull Length	53	1.15	0.0248	1.06	0.0838	0.98	0.0997
	Maxilla Length	55	1.07	0.0303	2.07	0.0824	0.96	0.133
	Dentary Length	55	1.22	0.0402	1.38	0.118	0.94	0.155

To test the assumption that anatomical proportions are conserved between extant and extinct members of thewithin any particular lizard lineages represented in this study, the SVL values predicted from individual bones were compared with those measured from complete skeletons the regressions were tested using complete skeletons of both extant and extinct taxa from the same lineage and, when available, fossil congeners as well . (S1-S7 Tables). The fossil test specimens included three of the few available complete fossil lizard skeletons that were measured: a large-bodied varanid (*Saniwa ensidens*, FMNH PR 2378, SVL 425 mm which is smaller than the largest extant varanid measured in this study (*V. exanthematicus*, 770 mm); Fig 4Fig 4A), a small-bodied anguid (*cf.* *Parophisaurus pawneensis*, YPM VP 060609, SVL 138 mm; not yet published at the time of this publication and thus not pictured here), and a very small-bodied polychrotid iguanian (*Afairiguana avius*, FMNH PR 2379, SVL 46 mm; Fig 5Fig 5B). For each SVL regression tested, the 95% confidence interval for the SVL estimate of each test specimen was calculated by adding or subtracting the standard error of the regression (S1-S7 Tables).

No complete dry skeletonized specimens of extant *Shinisaurus* were available to measure. Only one dry skull and four wet preserved specimens of this monotypic family were found in collections. Fortuitously, however, the holotype and only known complete skeleton of

Formatted: Font: Italic

an extinct taxon referred to Shinisauridae, *Bahndwivici ammoskius* (FMNH PR 2260, SVL 154 mm; Fig 5A) [76] had the same head length : SVL ratio as the average calculated for extant *Shinisaurus* based on wet preserved specimens (0.23 vs. 0.22, n = 4; S2 Dataset). Therefore, ratios of individual cranial elements to SVL from the *Bahndwivici* skeleton were used to estimate SVL for the handful of fossil specimens referred to Shinisauridae (S1 Dataset).

~~The only known complete skeleton of a fossil shinisaurid, *Bahndwivici ammoskius* (FMNH PR 2260, SVL 154 mm; Fig 5A) was also measured and used to estimate SVL for the other fossil shinisaurid specimens based on anatomical ratios because complete dry skeletonized extant specimens for Shinisauridae were not available. See Supporting Information for more details on materials and methods.~~

Formatted: Indent: First line: 0.5"

Estimations and new equations for~~ing~~ body mass ~~for fossils~~

It has been shown that b~~B~~ody mass scales linearly with SVL in extant lizards with limbs [15,24]. This relationship allowed ~~Other~~ researchers to previously develop allometric equations useful for estimating lizard body mass based on SVL at the~~ed~~ family ~~-~~level ~~SVL-to-mass allometric equations for extant lizards~~ (S8 Table) [24,41,42] as well as a general SVL-to-mass equation~~s~~ for all lizards (RMA: Body Mass in grams~~kg~~ = (((3.088±0.067)*SVL) – (4.852±0.128))/1000, R^2 = 0.946) [24]. Body mass for each fossil specimen in the dataset presented here (S1 Dataset) was calculated using ~~both~~ the published family-level equations (S8 Table), ~~and~~ as well as the published general RMA equation~~s~~ ~~from these published studies~~for comparison ~~for comparison~~.

This study also generated n~~N~~ew equations ~~were also generated~~ for estimating lizard body mass directly from individual ~~bones~~ elements (S1-S7 Tables). This was done by ~~combining~~

substituting an equation from this study for estimating SVL from a specific element for a given family (e.g., head length-to-SVL for Anguidae, S1 Table) into the published equation~~s generated in this study for estimating SVL from individual bones (S1-S7 Tables) with the equations published elsewhere~~ for estimating lizard body mass from SVL for that same family (S8 Table), producing a new ~~. The resulting~~ equation~~s~~ for calculating mass directly from that specific element (e.g., head length-to-mass for Anguidae, S1 Table). Thus, when applied to the fossil lizard specimens in S1 Dataset, the new element-to-mass equations produced the same body mass estimates~~s for the fossil lizards as the estimates~~as those obtained from the previously published equations (S1 Dataset, S8 Table).

Analysis of body size evolution

Maximum and mean SVL and mass were used as proxies for understanding patterns of lizard body size evolution through the Paleogene in the Western Interior of North America. The novel equations generated in this study for estimating body length and the published equations referenced in S8 Table for calculating body mass were applied to all fossil lizard specimens in S1 Dataset. When estimating body length or mass for a fossil specimen that included more than one complete cranial or limb element, the corresponding regression that offered the lowest SEE was used (S1-S7 Tables). All analyses were conducted at the level of crown-group assignments [63–65]. Fossil data were grouped by North American Land Mammal Ages (NALMAs), which compose a relative chronology used to divide the Paleogene based on taxonomic turnover in the mammal fossil record [77,78].

Rarefaction was used to analyze sample sizes though the Paleogene in this study system. S1 Dataset was subsampled by NALMA interval to determine the expected size range per

Formatted: PLOS One Level 2 Heading, Indent: First line: 0", Line spacing: single

Formatted: Indent: First line: 0.5", Widow/Orphan control, Adjust space between Latin and Asian text, Adjust space between Asian text and numbers

Formatted: Not Highlight

NALMA based on sample size. Each fossil specimen in S1 Dataset was assigned to one of 11 size groups for SVL, each group representing a bin of 100 mm (e.g., 1-99 mm, 100-199 mm, 200-299 mm, etc.; the last group was SVL ≥ 1 m). Individual rarefaction analysis was performed using PAST v4.03 [71].

Formatted: Font: Not Bold

Results

Estimating Regression equations for lizard body size of fossil lizards

Results investigating the relationships between different skeletal elements and body size across the lizard crown groups represented in this study indicated that the mostSkull and mandible length consistently provided consistently reliable regressions cranial measurements for estimating SVL were head length (standard error of the estimate ((SEE) ≤≤ 0.0997, Anguidae and Iguania997) and maxilla length (SEE ≤ 0.1331, Anguidae, Varanidae, Scincidae, Xantusiidae, Iguania;) across relevant taxonomic groups represented in this study (Table 3, Fig 6, Table 2, S1-S7 Tables).. These measurements are known to correlate with SVL widely among extant lizards [12,21,22]. Regressions based on limb measurements (femur, humerus, or tibia length) also were also consistently accurate across groupshad strong predictive power for Anguidae and Varanidae, the only two groups with fossil limb material available in the fossil dataset (SEE ≤ 0.0815). Greater variation in rankings was observed among lizard groups for rRegressions based on dentary, maxilla, frontal, and parietal length had the greatest variation in rankings among lizard groups (SEE 0.0530 – 0.1571). General equations based on the entire extant dataset (S2 Dataset) proved unsuccessful because none passed tests for both homoskedasticity and normal distribution of residuals (S9 Table).

Table 2. Regression functions and associated statistics for snout-vent length estimation from individual bones. All functions were transformed using the natural log (LN). Standard error for slope and intercept terms are listed after the slope and intercept in the equations. Functions are listed in order of increasing standard error of the estimate (SEE) for each group. If a particular fossil specimen included more than one complete cranial or limb measurement, the applicable regression that offered the lowest SEE was used. P(uncorrelated) < 0.001 for all functions.

**Group**	**Predictor**	***n***	**Slope *b***	**Standard error of *b***	**Intercept**	**Standard error of intercept**	R^2	**SEE**
Anguidae	Skull Length	46	0.946	0.0417	1.71	0.129	0.91	0.0740
	Femur Length	43	0.888	0.0410	2.46	0.101	0.91	0.0742
	Mandible Length	43	0.921	0.0424	1.82	0.129	0.91	0.0760
	Humerus Length	41	0.889	0.0451	2.57	0.106	0.90	0.0815
	Parietal Length	24	1.03	0.0658	2.29	0.148	0.91	0.0853
	Maxilla Length	22	1.02	0.0803	2.40	0.170	0.88	0.0992
	Frontal Length	22	1.27	0.111	2.13	0.216	0.85	0.120
	Dentary Length	24	0.898	0.0816	2.43	0.195	0.82	0.121
Varanidae	Tibia Length	27	0.953	0.0282	2.43	0.103	0.98	0.0695
	Maxilla Length	25	1.13	0.0423	2.04	0.145	0.97	0.0837
	Dentary Length	23	1.13	0.0845	1.86	0.305	0.88	0.157
Xenosauridae	Dentary Length	12	0.36	0.164	0.921	0.450	0.85	0.0530
	Parietal Length	12	1.03	0.154	2.17	0.369	0.78	0.0661
	Frontal Length	12	1.05	0.185	2.16	0.435	0.69	0.0787

Teiidae	Dentary Length	26	0.677	0.0686	2.70	0.165	0.75	0.0995
Scincidae	Maxilla Length	27	1.06	0.0352	2.20	0.0850	0.97	0.115
	Dentary Length	27	1.12	0.0426	1.74	0.115	0.96	0.133
Xantusiidae	Maxilla Length	20	0.698	0.0659	2.82	0.0944	0.88	0.119
	Dentary Length	20	0.787	0.0869	2.58	0.134	0.89	0.124
Iguania	Skull Length	53	1.15	0.0248	1.06	0.0838	0.98	0.0997
	Maxilla Length	55	1.07	0.0303	2.07	0.0824	0.96	0.133
	Dentary Length	55	1.22	0.0402	1.38	0.118	0.94	0.155

Fig 6. Regressions of dentary length to snout-vent length for all extant lizard groups sampled. Shinisauridae is not included here because no regressions were generated for that group. See Table 3 for regression equations and statistics. All regressions are transformed using the natural log (LN).

Formatted: Indent: First line: 0"

~~Cranial element morphology often varies between closely related lizard groups, more so than proportions of head or limb length to SVL [77]. This may be the reason why attempts to calculate general regression equations for this study proved unsuccessful: none of the general equations passed tests for both homoskedasticity and normal distribution of residuals (S9 Table). Meiri's [24] general equation for predicting mass from SVL for lizards (see S1 Dataset) was more viable likely because the relationship between SVL and mass appears to be more consistent than the relationship between head length or any individual element to SVL across lizard taxonomic groups.~~

The group-level regressions generated for body length in this study predicted actual SVL with comparable accuracy between the extant and fossil test specimens (Fig 6, Table 2, S1-S7 Tables). The actual SVL for each extant and fossil test specimen fell within or close to the 95% confidence interval for the estimated SVL (S1-S7 Tables). The percentage difference between predicted and actual SVL of test specimens ranged from 1% (varanid tibia length vs. SVL, SEE = 0.0695; S2 Table) to 36% (anguid frontal length vs. SVL, SEE = 0.120; S1 Table). ~~Clearly, some anatomical elements provide more accurate estimates than others for different lizard groups.~~

When large-bodied outliers were used to test the predictive power of the SVL regressions, the resulting percentage differences between actual and estimated SVL were comparable (see Varanidae, S2 Table) or lower (see Anguidae, S1 Table) than the other test specimens, indicating that those regressions can reliably estimate SVL for large individuals in those families. A large specimen of *Heloderma horridum* (Helodermatidae), the skull of which greatly resembles those of the largest anguid fossils in this dataset (Fig 3E), was used as an additional test specimen for the anguid regressions (S1 Table). Helodermatidae is placed close to Anguidae within the group Anguimorpha in both morphological and molecular phylogenetic analyses [67–70], so it is reasonable to consider Helodermatidae as an extant morphological and close phylogenetic analogue to large extinct anguid lizards. The anguid regressions proved comparably accurate in predicting the SVL of the largest helodermatid test specimen compared to the smaller extant and extinct anguid test specimens.

The family-specific SVL-to-mass equations [24,41,42] and the general SVL-to-mass equation [24] produced similar mass estimates for fossil specimens, with a percentage difference of 1% (Teiidae and Xantusiidae) to 16% (Xenosauridae). The novel element-to-mass equations

Formatted: Indent: First line: 0.5"

generated in this study were derived from the published family-specific SVL-to-mass equations, thus, the two sets of equations produced the same mass estimates for fossil specimens.

Taxonomic turnover

Anguids are known to be the most abundant lizards in the Paleogene fossil record of the Western Interior [80–82] and that was reflected in this study by their unrivaled taxonomic richness, which included at least 15 genera (Figs 6-7, Table 2, S1 Dataset). The results presented here also indicated that taxonomic diversity was greatest in the early Eocene and decreased in the early Oligocene (Figs 6-7, Table 2). Rarefaction analysis indicated that the number of crown groups recovered within a given NALMA peaked around a sample size of 50 specimens (S3 Fig). The Orellan sample included 64 specimens (Fig 6, S1 Dataset) and yet recovered only three crown groups (Table 2, S3 Fig), supporting the interpretation of a decline in diversity in the late Paleogene.

Fig 6. Snout-vent length distribution by taxonomic group for fossil lizards in the Western Interior of North America through the Paleogene. Sample sizes are per North American Land Mammal Age (NALMA). Data points are arbitrarily spread within each NALMA for visibility. NALMA abbreviations: Pu = Puercan, To = Torrejonian, Ti = Tiffanian, Cf = Clarkforkian, Wa = Wasatchian, Br = Bridgerian, Ui = Uintan, Du = Duchesnean, Ch = Chadronian, Or = Orellan, Wh = Whitneyan, Ar = Arikareean. Data points marked with an asterisk (*) were measured from complete fossil skeletons. All others were estimated from individual cranial or limb elements using regressions (see S1-S7 Tables). All measurements are transformed using natural log.

Formatted: Font: Not Bold

Formatted: Highlight

Formatted: Highlight

Formatted: Indent: First line: 0"

Formatted: Highlight

Formatted: Highlight

Fig 7. Body mass distribution by taxonomic group for fossil lizards in the Western Interior of North America through the Paleogene. Sample sizes are per North American Land Mammal Age (NALMA). Data points are arbitrarily spread within each NALMA for visibility. NALMA abbreviations: Pu = Puercan, To = Torrejonian, Ti = Tiffanian, Cf = Clarkforkian, Wa = Wasatchian, Br = Bridgerian, Ui = Uintan, Du = Duchesnean, Ch = Chadronian, Or = Orellan, Wh = Whitneyan, Ar = Arikareean. Masses were calculated with snout-vent length using the published equations listed in S8 Table. Data points marked with an asterisk (*) used SVLs measured from complete fossil skeletons. All others used SVLs estimated from individual cranial or limb element lengths using regressions (see S1-S7 Tables). All measurements are transformed using natural log.

The observed patterns may reflect some taphonomic or preservation bias (especially with respect to smaller bones), gaps in the rock record, and selective sampling by fossil collection crews. However, rarefaction analysis (S3 Fig) indicated that this study adequately sampled the upper range of body size diversity represented in this fossil record, especially from thoroughly documented areas like the Bighorn and Green River Basins (S1 Dataset; for further discussion, see the censuses of microvertebrate localities in this system by Smith [56–60]). Preservation bias toward complete elements from adult individuals may have mitigated other sources of bias when reconstructing maximum body size through geologic intervals.

The observed late Paleogene reductions in maximum body size represent the extinction of larger taxa [79,80]. Even in extant lizards, extinction risk increases with body size [3]. Among the eight lizard groups documented in this study from the Paleogene record of this region, only Anguidae, Scincidae, Teiidae, and Xantusiidae still occur in the area. Xenosaurids and the

~~iguanians included in this study that occurred in the Western Interior during the Paleogene have since been extirpated from the region; varanids and shinisaurids no longer occur anywhere in North America [4,11,13,81]. For further discussion, see Supporting Information.~~

Body size ~~evolution~~ in fossil lizards through the Paleogene

Maximum lizard SVL remained below 300 mm through the early and middle Paleocene (Puercan (66.0-63.8 Ma) – Tiffanian (60.9-56.2 Ma), ~~Fig 6~~Fig 7, ~~Table 4~~Table 3, S1 Dataset, S2 Fig, S10 Table). This upper SVL limit increased to almost 500 mm ~~and maximum body mass exceeded 1 kg~~ around the Paleocene-Eocene transition (Clarkforkian (56.2-54.9 Ma)~~; Fig 7~~). By the Wasatchian (54.9-50.5 Ma), maximum SVL reached 750 mm, and surpassed 1 m by the Bridgerian (50.5-46.2 Ma; ~~Table 4~~Table 3). Lizard body mass followed a similar pattern, with lizards exceeding 1 kg (LN(Mass in kg) = 0.00) by the Clarkforkian (Fig 8, Table 3, S1 Dataset, S3 Fig, S10 Table).

Fig 7. Body length distribution by taxonomic group for fossil lizards in the Western Interior of North America through the Paleogene. Body length is measured as snout-vent length (SVL) in mm. All measurements were transformed using natural log (LN). Data points marked with an asterisk (*) were measured from complete fossil skeletons. All others were estimated from individual cranial or limb elements using regressions (see S1-S7 Tables). Dashed line indicates mean lizard SVL per NALMA (see also S5 Fig). Sample sizes are per North American Land Mammal Age (NALMA). Data points are spread laterally within each NALMA for visibility. NALMA abbreviations: Pu = Puercan, To = Torrejonian, Ti = Tiffanian, Cf =

Clarkforkian, Wa = Wasatchian, Br = Bridgerian, Ui = Uintan, Du = Duchesnean, Ch = Chadronian, Or = Orellan, Wh = Whitneyan, Ar = Arikareean.

Table 3. Maximum and mean fossil lizard body size estimates per NALMA interval. NALMA age ranges are from Barnosky et al. [78]. Mean values are not listed for the Duchesnean because n = 1 for that NALMA.

Formatted: Indent: First line: 0"

**NALMA**	**Age Range (Ma)**	**Crown Group**	**Binomial**	**Specimen**	**Max SVL Estimate (mm)**	**Mean SVL Estimate (mm)**	**Max Mass Estimate (kg)**	**Mean Mass Estimate (kg)**
Puercan	66.0-63.8	Anguidae	*cf. Odaxosaurus piger*	UCM 34991	91	83	0.311	0.276
Torrejonian	63.8-60.9	Varanidae	cf. Varanidae indet.	UCMP 2671	191	113	0.615	0.373
Tiffanian	60.9-56.2	Anguidae	*Melanosaurus sp.*	UCM 98615	256	133	0.885	0.451
Clarkforkian	56.2-54.9	Anguidae	*Melanosaurus maximus*	UMMP 74618	487	269	1.69	0.932
Wasatchian	54.9-50.5	Anguidae	*Melanosaurus sp.*	UCMP 154536	788	232	2.74	0.793
Bridgerian	50.5-46.2	Anguidae	*Glyptosaurus sylvestris*	USNM 12590	1056	408	3.67	1.41
Uintan	46.2-39.7	Varanidae	*Saniwa ensidens*	FMNH UC 1719	540	391	1.74	1.28
Duchesnean	39.7-37.0	Anguidae	Anguidae indet.	CM 42469	116	N/A	0.399	N/A

Chadronian	37.0-33.9	Anguidae	*Helodermoides sp.*	UW 11057	1296	459	4.51	1.59
Orellan	33.9-31.8	Anguidae	*Peltosaurus sp.*	UCM 20877	360	176	1.25	0.604
Whitneyan	31.8-29.5	Anguidae	*Peltosaurus sp.*	SMM P81.8.71	224	194	0.775	0.669
Arikareean	29.5-23.0	Anguidae	*Peltosaurus sp.*	UNSM 81001	351	262	1.22	0.906

Formatted: Indent: First line: 0"

Fig 8. Body mass distribution by taxonomic group for fossil lizards in the Western Interior of North America through the Paleogene. Mass estimates were calculated from snout-vent length (SVL) using the published equations listed in S8 Table. All measurements (in kg) were transformed using natural log (LN). Data points marked with an asterisk (*) used SVLs measured from complete fossil skeletons. All others used SVLs estimated from individual cranial or limb element lengths using regressions (see S1-S7 Tables). Dashed line indicates mean lizard body mass per NALMA (see also S6 Fig). Sample sizes are per North American Land Mammal Age (NALMA). Data points are spread laterally within each NALMA for visibility. NALMA abbreviations: Pu = Puercan, To = Torrejonian, Ti = Tiffanian, Cf = Clarkforkian, Wa = Wasatchian, Br = Bridgerian, Ui = Uintan, Du = Duchesnean, Ch = Chadronian, Or = Orellan, Wh = Whitneyan, Ar = Arikareean.

~~**Table 4. Maximum lizard body size estimates per NALMA interval.** NALMA age ranges are from Barnosky et al. [79].~~

NALMA	Age Range (Mya)	Crown Group	Binomial	Specimen	Max SVL Estimate (mm)	Mass Estimate (kg)
Puercan	66.0-63.8	Anguidae	*cf. Odaxosaurus piger*	UCM 34991	91	0.311
Torrejonian	63.8-60.9	Varanidae	cf. Varanidae indet.	UCMP 2671	191	0.615
Tiffanian	60.9-56.2	Anguidae	*Melanosaurus sp.*	UCM 98615	256	0.885
Clarkforkian	56.2-54.9	Anguidae	*Melanosaurus maximus*	UMMP 74618	487	1.69
Wasatchian	54.9-50.5	Anguidae	*Melanosaurus sp.*	UCMP 154536	788	2.74
Bridgerian	50.5-46.2	Anguidae	*Glyptosaurus sylvestris*	USNM 12590	1056	3.67
Uintan	46.2-39.7	Varanidae	*Saniwa ensidens*	FMNH UC 1719	540	1.74
Duchesnean	39.7-37.0	Anguidae	Anguidae indet.	CM 42469	116	0.399
Chadronian	37.0-33.9	Anguidae	*Helodermoides sp.*	UW 11057	1296	4.51
Orellan	33.9-31.8	Anguidae	*Peltosaurus sp.*	UCM 20877	360	1.25
Whitneyan	31.8-29.5	Anguidae	*Peltosaurus sp.*	SMM P81.8.71	224	0.775
Arikareean	29.5-23.0	Anguidae	*Peltosaurus sp.*	UNSM 81001	351	1.22

Fig 7. Body mass distribution by taxonomic group for fossil lizards in the Western Interior of North America through the Paleogene. Sample sizes are per North American Land Mammal Age (NALMA). Data points are arbitrarily spread within each NALMA for visibility. NALMA abbreviations: Pu = Puercan, To = Torrejonian, Ti = Tiffanian, Cf = Clarkforkian, Wa = Wasatchian, Br = Bridgerian, Ui = Uintan, Du = Duchesnean, Ch = Chadronian, Or = Orellan, Wh = Whitneyan, Ar = Arikareean. Masses were calculated with snout-vent length using the published equations listed in S8 Table. Data points marked with an asterisk (*) used SVLs measured from complete fossil skeletons. All others used SVLs estimated from individual cranial or limb element lengths using regressions (see S1-S7 Tables). All measurements are transformed using natural log.

The pattern for the middle Eocene (Uintan – Duchesnean) was dubious. The observed maximum body size for the middle Eocene (540 mm, 1.74 kg, *Saniwa ensidens*, Figs 6-7, Table 4) was much lower than the early or late Eocene. Fewer outcrops are available from this interval in the U.S. Western Interior, so this result was likely an artifact of poor sampling (n = 4 for the Uintan, n = 1 for the Duchesnean, Fig 6). Rarefaction analysis (S4 Fig) demonstrated that the number of expected size groups recovered in a sample from this fossil dataset again peaked and leveled off around n = 50, supporting the assumption that the NALMAs with small sample sizes in this study did not capture the full range of lizard body size that was likely present during those intervals.

The sample size was higher for the late Eocene (n = 24 for the Chadronian, Fig 6). The late Eocene record (Chadronian, 37.0-33.9 Ma) record includedfeatured several large individuals, including the largest body size estimated in this study (1.3 m, 4.5 kg, *Helodermoides sp.*, Table

~~4~~Table 3). However, b~~B~~y the early Oligocene (Orellan, 33.9-31.8 Ma), maximum ~~SVL~~ body size dropped considerably ~~to 360 mm~~ (360 mm, 1.25 kg, *Peltosaurus sp.*) and remained below that limit through the late Paleogene. ~~The Orellan was very well sampled (n = 64) compared to other NALMAs, and consequently, sample size did not correlate strongly with maximum estimated SVL across the dataset (R^2 = 0.23, p(uncorr.) = 0.14). Together with rarefaction analysis (S4 Fig), these results indicate that the observed decrease in maximum lizard SVL in the late Paleogene is likely valid.~~

Discussion

Strength of body size regression equations

This study found skull length to be the strongest cranial element predictor of SVL in Anguidae and Iguania, in agreement with previous studies [12,21,22]. Limb bones were also strong predictors of SVL in Anguidae (femur and humerus length) and Varanidae (tibia length), as was previously demonstrated in crocodylians [27]. These elements were not available for the other lizard crown groups investigated in this study. Among those (Scincidae, Shinisauridae, Teiidae, Xantusiidae, Xenosauridae), maxilla length provided the most consistently accurate SVL estimate compared to SVL measurements from test specimens (S1-S2 Tables, S5-S7 Tables), followed by dentary length (S1-S7 Tables). The body mass equations could not be tested for accuracy in the same way because body mass data were not available for the extant test specimens. In terms of deviation from the general equation for all lizards, the body mass estimations from equations for Anguimorpha (used for Shinisauridae), Iguania, Teiidae, Varanidae, and Xantusiidae were the closest to the general estimation, with a percentage difference of 1-5% (S1 Dataset, S8 Table).

Attempts to derive general equations for calculating SVL from individual bones proved unsuccessful in this study (S9 Table). This might be because cranial element morphology and proportions often vary between closely related lizard groups [79]. Meiri's [24] general equation for predicting mass from SVL for lizards (see S1 Dataset) was likely more viable because the relationship between SVL and mass appears to be more consistent than the relationship between head length or any individual element to SVL across lizard taxonomic groups.

Separate regressions for males and females were not generated because most extant specimens sampled did not have a sex indicated. However, it was not an issue for this study because any fossil lizard dataset must be treated as an averaged sample across sexes. Although female lizards often have shorter SVLs than males [4], there is no reliable way to tell the sex of an individual fossil lizard specimen based on isolated bones.

The same is largely true for ontogenetic stages represented in a fossil dataset. Adult members of an extinct taxon can be reasonably identified if a large sample of referred individuals that reach a consistent maximum size range is present (e.g., large fossil anguids, S1 Dataset) or if some of those specimens include long bones with fused epiphyses. Complete anatomical elements from adult individuals are also more likely to get preserved as fossils. Juveniles are less likely to get preserved as fossils because they are frequently consumed by predators and their bones are more delicate [80]. This preservation bias toward adult specimens would not affect the resulting patterns of maximum body size for any NALMA with a decent sample size, even if some subadult or juvenile specimens were present in the fossil dataset. For these reasons, and to avoid skewing the regression equations with outliers, juvenile specimens were excluded from the extant dataset.

Body size evolution among Paleogene lizard groups

Anguids are known to be the most abundant lizards in the Paleogene fossil record of the Western Interior [81–83] and that was reflected in their dominance of the fossil dataset presented here (S1 Dataset). Anguids also accounted for the largest individuals in the dataset and most of the maximum sizes in each NALMA, with the exception of a few varanids (Figs 7-8, S2-S3 Figs, Table 3, S10 Table). Among the eight lizard crown groups sampled, only those two reached SVLs over 200 mm and masses over 1 kg. Interestingly, the largest extant limbed anguid today, *Diploglossus millepunctatus* (maximum SVL 280 mm), does not approach the size of the largest anguids from the Eocene, nor do the largest lizards occurring in North America today (*Heloderma horridum*, maximum SVL 520 mm [84], Fig 3E; *Iguana iguana*, maximum SVL 580 mm [4]). Conversely, Varanidae includes both the largest extant lizard (*Varanus komodoensis*, which exceeds the adult size of the largest Eocene anguid at around 1.5 m SVL [4,85]) and the largest known terrestrial lizard in the fossil record (*Varanus priscus*, known from Pleistocene deposits in Australia, with SVL at least 3 m [86,87]), but varanids are now restricted to the Eastern Hemisphere [13].

The other lizard groups in this geotemporal system (Iguania, Scincidae, Shinisauridae, Teiidae, Xantusiidae, Xenosauridae) did not experience an increase in body size range (Figs 7-8, S10 Table). Small-bodied lizards dominated the North American Paleogene lizard record. The maximum and mean body sizes for most lizard groups did not exceed 200 mm in SVL (LN(SVL) = 5.30, S2 Fig, S10 Table) or 1 kg in body mass (LN(Mass) = 0.00, S3 Fig, S10 Table).

The observed maximum body size for the middle Eocene (540 mm, 1.74 kg, *Saniwa ensidens* (Varanidae), Figs 6-7, Table 3, S10 Table) was much lower than that of the early or late Eocene. Fewer outcrops are available from this interval in the U.S. Western Interior, so this

result was likely an artifact of poor sampling (n = 4 for the Uintan (46.2-39.7 Ma), n = 1 for the Duchesnean (39.7-37.0 Ma), Fig 7). Rarefaction analysis (S4 Fig) demonstrated that the number of expected size groups recovered in a sample from this fossil dataset again peaked and leveled off around n = 50, supporting the assumption that the NALMAs with small sample sizes in this study did not capture the full range of lizard body size that was likely present during those intervals.

The observed patterns may reflect some taphonomic or preservation bias (especially with respect to smaller bones), gaps in the rock record, and selective sampling by fossil collection crews. However, rarefaction analysis (S3 Fig) indicated that this study adequately sampled the upper range of body size diversity represented in this fossil record, especially from thoroughly documented areas like the Bighorn and Green River Basins (S1 Dataset; for further discussion, see the censuses of microvertebrate localities in this system by Smith [56–60]). Preservation bias toward complete elements from adult individuals may have mitigated other sources of bias when reconstructing maximum body size through geologic intervals.

The decrease in maximum body size in the early Oligocene cannot be attributed to low sample size because the Orellan (33.9-31.8 Ma) was very well sampled (n = 64) compared to other NALMAs (Figs 6-7, S4 Fig). In fact, sample size did not correlate strongly with maximum estimated SVL across the entire dataset (R^2 = 0.23, p(uncorr.) = 0.14 for sample size *n* vs. LN(Max SVL)). The observed late Paleogene reductions in maximum body size likely represent the extirpation or extinction of the largest Paleogene lizard taxa in the region [88,89]. Even in extant lizards, extinction risk increases with body size [3]. Among the eight lizard groups documented in this study from the Paleogene record of this region, only Anguidae, Scincidae, Teiidae, and Xantusiidae still occur in the area. Xenosaurids and the iguanians included in this

study that occurred in the Western Interior during the Paleogene have since been extirpated from the region; varanids and shinisaurids no longer occur anywhere in North America [4,11,13,85].

In general, the observed patterns may reflect some taphonomic or preservation bias (especially with respect to smaller bones), gaps in the rock record, and selective sampling by fossil collection crews. However, rarefaction analysis (S4 Fig) indicated that this study adequately sampled the upper range of body size diversity represented overall in this fossil record, especially from thoroughly documented areas like the Bighorn and Green River Basins (S1 Dataset; for further discussion, see the censuses of microvertebrate localities in this system by Smith [56–60]). Preservation bias toward complete elements from adult individuals may have mitigated other sources of bias when reconstructing maximum body size through geologic intervals.

Climate as a potential driver of ~~Paleogene lizard~~ body size evolution

Environmental temperature can influence body size evolution in reptiles [3,9,31,90–92]. Metabolic rate increases with temperature and scales with body size in all poikilothermic vertebrates [1,93,94], including lizards [15,95]. Changes in environmental temperature are known to correlate globally with changes in maximum body size in extant lizards (exclusive of snakes [25]). Increased temperatures around the Paleocene-Eocene Thermal Maximum (PETM, 56 M~~y~~a; [44,96]) could have elevated metabolism and released a physiological constraint on maximum size for some lizard taxa. Gigantism has been documented in other fossil reptiles in conjunction with elevated temperatures, including a boid snake (*Titanoboa*, total length 13 m, 60-58 M~~y~~a, Colombia [31]), a caimanine crocodyliform (*Purussaurus*, total length 12.5 m, 8

M~~y~~a, northern South America [97]), and a varanid lizard (*Varanus priscus*, total length up to 5.7 m, around 50 k~~y~~a, Australia [86,87]).

To investigate this possibility, RMA linear regression was applied to test whether maximum and mean fossil lizard SVL predicted global temperature per NALMA (~~S5 Fig~~S5 Fig, S10 Table). Global temperature data were obtained from Zachos et al. [43,44], a widely used dataset of Cenozoic paleotemperatures derived from marine oxygen isotopes (e.g., [10,49]). Here, fossil lizard body size showed no relationship to global temperature through the Paleogene. It is possible that the relationship observed today is not detectable across scales of millions of years, perhaps due to t~~.~~ aphonomic or sampling bias in the fossil record. It might also be more pertinent to compare these data with terrestrial paleotemperature proxies from the same geotemporal system sampled in this study. This could be a fruitful area for continued research to build on the data presented here.

Alternatively, body size evolution patterns observed here could reflect indirect effects of climate [98]. Temperatures were warmer during both the summer and winter months around the PETM, and most precipitation came during the summer [99–101]. This would have allowed lizards to be active and growing during a longer portion of the year [98,102] and could also have led to greater food availability from increased productivity, especially in the summer months [103–105]. Previous studies found elevated growth rates in extant tropical lizards occupying areas that received greater precipitation [106]. Attaining large body size in a closed tropical forest system with an equable climate, like that present in the Western Interior in the early Eocene [103,104,107,108], would also have allowed lizards to spend less time and energy on active thermoregulation [1,109]. Large body size sometimes corresponds with thermoconforming

behavior and nocturnal activity in extant lizards [3,12,102], and these strategies could have reduced competition with diurnal organisms.

Herbivory can also lead to large body size in lizards [3,4,95]. This has been inferred for at least one giant fossil lizard [9]. However, the dentition of the largest lizards in this study were more indicative of omnivorous or insectivorous (Anguidae, ~~Fig 3~~Fig 3A) and carnivorous diets (Varanidae, ~~Fig 4~~Fig 4B; see [21,110]), rather than herbivory. ~~It may be impossible to determine the specific mechanisms of gigantism in lizards in this system, but the results presented here elucidate clear patterns of body size evolution.~~

> **Formatted:** Indent: First line: 0"

Lizards can maintain smaller body sizes in warm climates as a result of behavioral thermoregulation [109], competition, predation, or specializations related to resource zones of small taxa [92,111]. This might explain why not all lizard groups in this fossil dataset exhibited an increase in body size. These results may reflect an ecological hierarchy in the Paleogene communities of the Western Interior similar to extant ecosystems in which large-bodied lizards are less abundant than small-bodied lizards [3].

> **Formatted:** Normal, Indent: First line: 0.5", Line spacing: Double

~~Cranial element morphology often varies between closely related lizard groups, more so than proportions of head or limb length to SVL [77]. This may be the reason why attempts to calculate general regression equations for this study proved unsuccessful: none of the general equations passed tests for both homoskedasticity and normal distribution of residuals (S9 Table). Meiri's [24] general equation for predicting mass from SVL for lizards (see S1 Dataset) was more viable likely because the relationship between SVL and mass appears to be more consistent than the relationship between head length or any individual element to SVL across lizard taxonomic groups.~~

Body size evolution among taxonomic groups

Anguids are known to be the most abundant lizards in the Paleogene fossil record of the Western Interior [107–109] and that was reflected in this study by their unrivaled taxonomic richness, which included at least 15 genera (Figs 6-7, Table 2, S1 Dataset). Despite increased environmental temperatures in the early Eocene, most of the lizards in this geotemporal system did not experience an increase in body size range. Small-bodied lizards dominated the North American Paleogene lizard record. The maximum and mean body sizes for most lizard groups did not exceed 200 mm in SVL (LN(SVL) = 5.30, S6 Fig, S11 Table) or 1 kg in body mass (LN(Mass) = 0.00, S7 Fig, S11 Table). Maximum body size in extant lizards can stay comparatively low in warm climates due to behavioral thermoregulation [104], competition, predation, or specializations related to resource zones of small taxa [85,110]. The results presented here may reflect an ecological hierarchy in the Paleogene communities of the Western Interior similar to extant ecosystems in which large-bodied lizards are less abundant [3].

Among the eight lizard crown groups sampled, only two reached SVLs over 200 mm and masses over 1kg: Anguidae and Varanidae (Figs 6-7, S6-S7 Figs, Table 4). Interestingly, the largest extant limbed anguid today, *Diploglossus millepunctatus* (maximum SVL 280 mm), does not approach the size of the largest anguids from the Eocene, nor do the largest lizards occurring in North America today (*Heloderma horridum*, maximum SVL 520 mm [111], Fig 3E; *Iguana iguana*, maximum SVL 580 mm [4]). Conversely, Varanidae includes both the largest extant lizard (*Varanus komodoensis*, which exceeds the adult size of the largest Eocene anguid at around 1.5 m SVL [4,82]) and the largest known terrestrial lizard in the fossil record (*Varanus priscus*, known from Pleistocene deposits in Australia, with SVL at least 3 m [91,92]), but varanids are now restricted to the Eastern Hemisphere [13].

Conclusions

This study generated novel equations for estimating fossil lizard body size from isolated anatomical elements, with skull length, limb bone length, and maxilla length providing the most accurate estimates for SVL. Body mass can also be calculated from individual bones based on these equations. ~~This study~~Application of these equations to the Paleogene record of lizards in the Western Interior of North America offers the first survey and reconstruction of body size across crown lizard groups through a prolonged geologic interval and across a large continental interior region. The findings indicate that lizard body size peaked in the early Eocene in this system. Only two lizard groups, Anguidae and Varanidae, reached large body sizes exceeding 1 m in SVL and 1 kg in mass. Body size range did not change considerably for other groups. ~~Both~~ M~~m~~aximum lizard body size ~~and taxonomic diversity~~ decreased ~~for lizards~~ across the Western Interior in the early Oligocene. The methods presented here can be applied to other lizard clades to generate reasonably accurate body size estimates for fossil taxa and to study patterns of body size evolution across higher taxonomic groups.

Formatted: Highlight

Formatted: Highlight

Acknowledgments

The author wishes to thank K. Padian, J. McGuire, A. Barnosky, and I. Wang for their mentorship and helpful comments on this work, as well as J. Head, who mentored the research that started this project. The following individuals provided assistance and access to museum collections: M. Norell, C. Mehling, J. Meng, D. Kizirian, L. Vonnahme, M. Arnold (AMNH); J. Vindum, L. Scheinberg, E. Ely (CAS); M. Lamanna, J. Padial, A. Henrici, S. Rogers (CM); T. Lyson, L. Ivy, K. MacKenzie (DMNH); J. Bloch, R. Hulbert, J. Bourque (FLMNH); P. Makovicky, L. Grande, A. Resetar, W. Simpson, A. Stroupe, J. Mata (FMNH); A. Aase (FOBU); N. Smith, S. McLeod, V. Rhue (LACM); J.Rosado (MCZ); J. McGuire, C. Spencer (MVZ); T. Williamson (NMMNH); A. Farke, G. Santos (RAM); A. Hastings, E. Whiting (SMM); C. Bell, M. Brown (TMM); T. Culver, N. Ridgwell (UCM); P. Holroyd, C. Marshall (UCMP); J. Wilson, A. Rountrey (UMMP); R. Hunt, Jr., P. Freeman, G. Corner, T. Labedz (UNSM); H. Dieter-Sues, M. Carrano, M. Brett-Surman, A. Millhouse (USNM); L. Vietti (UW); C. Sidor, R. Eng, M. Rivin (UWBM); J. Gauthier, C. Norris, D. Brinkman (YPM). The author thanks the following for helpful discussions: C. Badgley, C. Bell, B-A. Bhullar, J. Bloch, W. Clemens†, C. Crumly, J. Gauthier, W. Gearty, P. Gingerich, L. Grande, A. Gunderson, P. Holroyd, E. Holt, R. Huey, R. Hunt, Jr., M. Kemp, D. Meyer, D. Miles, J. McGuire, J. Parham, H. Petermann, R. Secord, M. Stocker, R. Sullivan, D. Watkins, L. Weaver, S. Werning, E. Whiting, C. Williams, the MVZ Herp Group, the entire UCMP community. C. Bell also provided access to obscure literature. D. Meyer provided measurements of an unpublished specimen. G. Brown (UNSM) prepared a new *Peltosaurus* specimen (UNSM 12102) containing critical limb material. F. Vasconcellos provided the photo in ~~Fig 3~~Fig 3E. B. Chelemedos and C. Wong assisted with data collection. E. Whiting provided instruction on using QGIS to make maps. J. Lipps helped to design a visually

accessible color palette for ~~Fig 2~~Fig 2. The author thanks C. Meloro, S. Scarpetta, and ~~an~~ two anonymous reviewers for their constructive comments which greatly helped to improve the manuscript.

References

1. Peters RH. The Ecological Implications of Body Size. 1st ed. Beck E, Birks HJB, Connor EF, editors. New York, NY: Cambridge University Press; 1983.
2. Schmidt-Nielsen K. Scaling: Why is Animal Size So Important? Cambridge: Cambridge University Press; 1984.
3. Meiri S. Evolution and ecology of lizard body sizes. Glob Ecol Biogeogr. 2008;17: 724–734. doi:10.1111/j.1466-8238.2008.00414.x
4. Meiri S. Traits of lizards of the world: Variation around a successful evolutionary design. Glob Ecol Biogeogr. 2018;27: 1168–1172. doi:10.1111/geb.12773
5. Damuth J. Problems in estimating body masses of archaic ungulates using dental measurements. Body Size Mamm Paleobiol Estim Biol Implic. 1990;229: tables 16.9-16.10.
6. Figueirido B, Pérez-Claros JA, Hunt RM, Palmqvist P. Body mass estimation in amphicyonid carnivoran mammals: A multiple regression approach from the skull and skeleton. Acta Palaeontol Pol. 2011;56: 225–246. doi:10.4202/app.2010.0005
7. Campione NE, Evans DC. A universal scaling relationship between body mass and proximal limb bone dimensions in quadrupedal terrestrial tetrapods. BMC Biol. 2012;10. doi:10.1186/1741-7007-10-60
8. Thomas Goodwin H, Bullock KM. Estimates of body mass for fossil giant ground squirrels, genus *Paenemarmota*. J Mammal. 2012;93: 1169–1177. doi:10.1644/11-MAMM-A-312.3
9. Head JJ, Gunnell GF, Holroyd PA, Howard Hutchison J, Ciochon RL. Giant lizards occupied herbivorous mammalian ecospace during the Paleogene greenhouse in Southeast

Asia. Proc R Soc B Biol Sci. 2013;280. doi:10.1098/rspb.2013.0665

10. Godoy PL, Benson RBJ, Bronzati M, Butler RJ. The multi-peak adaptive landscape of crocodylomorph body size evolution. BMC Evol Biol. 2019;19: 1–29. doi:10.1186/s12862-019-1466-4

11. Vidan E, Novosolov M, Bauer AM, Herrera FC, Chirio L, Campos Nogueira C, et al. The global biogeography of lizard functional groups. J Biogeogr. 2019;00: 1–12. doi:10.1111/jbi.13667

12. Pianka ER, Vitt LJ, Pelegrin N, Fitzgerald DB, Winemiller KO. Toward a Periodic Table of Niches, or Exploring the Lizard Niche Hypervolume. Am Nat. 2017;190: 000–000. doi:10.1086/693781

13. Pianka ER, Vitt LJ. Lizards: Windows to the Evolution of Diversity. Berkeley: University of California Press; 2003.

14. Vitt LJ, Pianka ER, Cooper WE, Schwenk K. History and the global ecology of squamate reptiles. Am Nat. 2003;162: 44–60. doi:10.1086/375172

15. Pough FH. The advantages of ectothermy for tetrapods. Am Nat. 1980;115: 92–112. doi:10.1086/283547

16. Muñoz MM, Bodensteiner BL. Janzen’s Hypothesis meets the Bogert Effect: Connecting climate variation, thermoregulatory behavior, and rates of physiological evolution. Integr Org Biol. 2019;1: 1–12. doi:10.1093/iob/oby002

17. Muñoz MM, Losos JB. Thermoregulatory behavior simultaneously promotes and forestalls evolution in a tropical lizard. Am Nat. 2018;191: E15–E26. doi:10.1086/694779

18. Muñoz MM, Stimola MA, Algar AC, Conover A, Rodriguez AJ, Landestoy MA, et al. Evolutionary stasis and lability in thermal physiology in a group of tropical lizards. Proc R

Soc B Biol Sci. 2014;281: 20132433. doi:10.1098/rspb.2013.2433

19. Roll U, Feldman A, Novosolov M, Allison A, Bauer AM, Bernard R, et al. The global distribution of tetrapods reveals a need for targeted reptile conservation. Nat Ecol Evol. 2017;1: 1677–1682. doi:10.1038/s41559-017-0332-2

20. Vitt E.R. Pianka, W.E. Cooper, Jr., and K. Schwenk LJ. History and the global ecology of squamate reptiles. Am Nat. 2003;162: 44–60.

21. Herrera-Flores JA, Stubbs TL, Benton MJ. Ecomorphological diversification of squamates in the Cretaceous. R Soc Open Sci. 2021;8: 1–11. doi:10.1098/rsos.201961

22. Metzger KA, Herrel A. Correlations between lizard cranial shape and diet: A quantitative, phylogenetically informed analysis. Biol J Linn Soc. 2005;86: 433–466. doi:10.1111/j.1095-8312.2005.00546.x

23. Olalla-Tárraga MÁ, Rodríguez MÁ, Hawkins BA. Broad-scale patterns of body size in squamate reptiles of Europe and North America. J Biogeogr. 2006;33: 781–793. doi:10.1111/j.1365-2699.2006.01435.x

24. Meiri S. Length-weight allometries in lizards. J Zool. 2010;281: 218–226. doi:10.1111/j.1469-7998.2010.00696.x

25. Slavenko A, Feldman A, Allison A, Bauer AM, Böhm M, Chirio L, et al. Global patterns of body size evolution in squamate reptiles are not driven by climate. Glob Ecol Biogeogr. 2019;28: 471–483. doi:10.1111/geb.12868

26. Villa A, Delfino M. A comparative atlas of the skull osteology of European lizards (Reptilia: Squamata). Zool J Linn Soc. 2019; 1–100. doi:10.1093/zoolinnean/zlz035

27. Farlow JO, Hurlburt GR, Elsey RM, Britton ARC, Langston W. Femoral dimensions and body size of *Alligator mississippiensis*: Estimating the size of extinct mesoeucrocodylians.

J Vertebr Paleontol. 2005;25: 354–369. doi:10.1671/0272-4634(2005)025[0354:FDABSO]2.0.CO;2

28. O'Brien HD, Lynch LM, Vliet KA, Brueggen J, Erickson GM, Gignac PM. Crocodylian Head Width Allometry and Phylogenetic Prediction of Body Size in Extinct Crocodyliforms. Integr Org Biol. 2019;1. doi:10.1093/iob/obz006

29. Egi N. Body mass estimates in extinct mammals from limb bone dimensions: The case of North American Hyaenodontids. Palaeontology. 2001;44: 497–528. doi:10.1111/1475-4983.00189

30. Valkenburgh B Van. Skeletal and dental predictors of body mass in carnivores. Body size in Mammalian Paleobiology: Estimation and biological implications. 1990. pp. 181–205.

31. Head JJ, Bloch JI, Hastings AK, Bourque JR, Cadena EA, Herrera FA, et al. Giant boid snake from the Palaeocene neotropics reveals hotter past equatorial temperatures. Nature. 2009;457: 715–718. doi:10.1038/nature07671

32. Sereno PC, Larsson HCE, Sidor CA, Gado B. The giant crocodyliform *Sarcosuchus* from the cretaceous of Africa. Science (80-). 2001;294: 1516–1519. doi:10.1126/science.1066521

33. Bochaton C, Kemp ME. Reconstructing the body sizes of Quaternary lizards using *Pholidoscelis Fitzinger*, 1843, and Anolis Daudin, 1802, as case studies. J Vertebr Paleontol. 2017;37: 9 pp. doi:10.1080/02724634.2017.1239626

34. Herrera-Flores JA, Elsler A, Stubbs TL, Benton MJ. Slow and fast evolutionary rates in the history of lepidosaurs. Palaeontology. 2022;65: 1–13. doi:10.1111/pala.12579

35. Arnold EN. Evolutionary aspects of tail shedding in lizards and their relatives. J Nat Hist. 1984;18: 127–169. doi:10.1080/00222938400770131

36. Bateman PW, Fleming PA. To cut a long tail short: A review of lizard caudal autotomy studies carried out over the last 20 years. J Zool. 2009;277: 1–14. doi:10.1111/j.1469-7998.2008.00484.x

37. Gilbert EAB, Payne SL, Vickaryous MK. The anatomy and histology of caudal autotomy and regeneration in lizards. Physiol Biochem Zool. 2013;86: 631–644. doi:10.1086/673889

38. Sanggaard KW, Danielsen CC, Wogensen L, Vinding MS, Rydtoft LM, Mortensen MB, et al. Unique Structural Features Facilitate Lizard Tail Autotomy. PLoS One. 2012;7. doi:10.1371/journal.pone.0051803

39. Zani PA. Patterns of caudal-anatomy evolution in lizards. J Zool. 1996;240: 201–230.

40. Greer AE. Distribution of Maximum Snout-Vent Length among Species of Scincid Lizards. J Herpetol. 2001;35: 383–395.

41. Meiri S, Bauer AM, Chirio L, Colli GR, Das I, Doan TM, et al. Are lizards feeling the heat?: a tale of ecology and evolution under two temperatures. Glob Ecol Biogeogr. 2013;22: 834–845.

42. Feldman A, Sabath N, Pyron RA, Mayrose I, Meiri S. Body sizes and diversification rates of lizards, snakes, amphisbaenians and the tuatara. Glob Ecol Biogeogr. 2016;25: 187–197. doi:10.1111/geb.12398

43. Zachos J, Pagani M, Sloan L, Thomas E, Billups K. Trends, rhythms and aberrations in global climate 65 Ma to present. Science (80-). 2001;292: 686–693.

44. Zachos JC, Dickens GR, Zeebe RE. An early Cenozoic perspective on greenhouse warming and carbon-cycle dynamics. Nature. 2008;451: 279–283. doi:10.1038/nature06588

45. Woodburne MO, Gunnell GF, Stucky RK. Climate directly influences Eocene mammal faunal dynamics in North America. Proc Natl Acad Sci. 2009;106: 13399–13403. doi:10.1073/pnas.0906802106

46. Gunnell GF, Zonneveld JP, Bartels WS. Stratigraphy, mammalian paleontology, paleoecology, and age correlation of the Wasatch Formation, Fossil Butte National Monument, Wyoming. J Paleontol. 2016;90: 981–1011. doi:10.1017/jpa.2016.100

47. Saarinen JJ, Boyer AG, Brown JH, Costa DP, Ernest M, Evans AR, et al. Patterns of maximum body size evolution in Cenozoic land mammals: eco-evolutionary processes and abiotic forcing. Proc R Soc B Biol Sci. 2014;281: 1–10.

48. Rose KD. Composition and Species Diversity in Paleocene and Eocene Mammal Assemblages: An Empirical Study. J Vertebr Paleontol. 1981;1: 367–388.

49. Lovegrove BG, Mowoe MO. The evolution of mammal body sizes: Responses to Cenozoic climate change in North American mammals. J Evol Biol. 2013;26: 1317–1329. doi:10.1111/jeb.12138

50. Lyson TR, Miller IM, Bercovici AD, Weissenburger K, Fuentes AJ, Clyde WC, et al. Exceptional continental record of biotic recovery after the Cretaceous−Paleogene mass extinction. Science (80-). 2019;366: 977–983. doi:10.1126/science.aay2268

51. Alroy J. Constant extinction, constrained diversification, and uncoordinated stasis in North American mammals. Palaeogeogr Palaeoclimatol Palaeoecol. 1996;127: 285–311.

52. Alroy J. Cope's Rule and the Dynamics of Body Mass Evolution in North American Fossil Mammals. Science (80-). 1998;280: 731–734.

53. Alroy J. New Methods for Quantifying Macroevolutionary Patterns and Processes. Paleobiology. 2000;26: 707–733.

54. Alroy J. Speciation and extinction in the fossil record of North American mammals. In: Butlin RK, Brindle JR, Schluter D, editors. Speciation and Patterns of Diversity. Cambridge University Press; 2009. pp. 301–323.

55. Huang S, Eronen JT, Janis CM, Saarinen JJ, Silvestro D, Fritz SA. Mammal body size evolution in North America and Europe over 20 Myr: Similar trends generated by different processes. Proc R Soc B Biol Sci. 2017;284: 1–9. doi:10.1098/rspb.2016.2361

56. Smith KT, Gauthier JA. Early Eocene lizards of the Wasatch Formation near Bitter Creek, Wyoming: Diversity and paleoenvironment during an interval of global warming. Bull Peabody Museum Nat Hist. 2013;54: 135–230. doi:10.3374/014.054.0205

57. Smith KT. A diverse new assemblage of Late Eocene squamates (Reptilia) from the Chadron Formation of North Dakota, U.S.A. Palaeontol Electron. 2006;9: 1–44.

58. Smith KT. A new lizard assemblage from the earliest Eocene (zone Wa0) of the Bighorn Basin, Wyoming, USA: Biogeography during the warmest interval of the Cenozoic. Journal of Systematic Palaeontology. 2009. doi:10.1017/S1477201909002752

59. Smith KT. The evolution of mid-latitude faunas during the Eocene: Late Eocene lizards of the Medicine Pole Hills reconsidered. Bull Peabody Museum Nat Hist. 2011;52: 3–105.

60. Smith KT. The long-term history of dispersal among lizards in the early Eocene: New evidence from a microvertebrate assemblage in the Bighorn Basin of Wyoming, USA. Palaeontology. 2011;54: 1243–1270. doi:10.1111/j.1475-4983.2011.01107.x

61. Woolley CH, Thompson JR, Wu Y-H (Beckey), Bottjer DJ, Smith ND. Can a fragmented past be trusted? Assessing bias and phylogenetic signal in the squamate fossil record. J Vertebr Paleontol Progr Abstr. 2020; 351–352.

62. Woolley CH, Thompson JR, Wu YH, Bottjer DJ, Smith ND. A biased fossil record can

preserve reliable phylogenetic signal. Paleobiology. 2022; 1–16. doi:10.1017/pab.2021.45

63. Bell CJ, Gauthier JA, Bever GS. Covert biases, circularity, and apomorphies: A critical look at the North American Quaternary Herpetofaunal Stability Hypothesis. Quat Int. 2010;217: 30–36. doi:10.1016/j.quaint.2009.08.009

64. Ledesma DT, Scarpetta SG, Bell CJ. Variation in the skulls of *Elgaria* and *Gerrhonotus* (Anguidae, Gerrhonotinae) and implications for phylogenetics and fossil identification. PeerJ. 2021;9: 1–80. doi:10.7717/peerj.11602

65. Scarpetta SG, Ledesma DT, Bell CJ. A new extinct species of alligator lizard (Squamata: *Elgaria*) and an expanded perspective on the osteology and phylogeny of Gerrhonotinae. BMC Ecol Evol. 2021;21: 1–58. doi:10.1186/s12862-021-01912-8

66. The Paleobiology Database. Available: www.paleobiodb.org

67. Estes R, De Queiroz K, Gauthier J. Phylogenetic Relationships within Squamata. In: Estes R, Pregill G, editors. Phylogenetic Relationships of the Lizard Families. Palo Alto, CA: Stanford University Press; 1988. pp. 119–281.

68. Frost DR, Etheridge R. A phylogenetic analysis and taxonomy of iguanian lizards. Univ Kansas Museum Nat Hist Misc Publ. 1989;81: 1–65.

69. Gauthier JA, Kearney M, Maisano JA, Rieppel O, Behlke ADB. Assembling the Squamate Tree of Life : Perspectives from the Phenotype and the Fossil Record Assembling the Squamate Tree of Life : Perspectives from the Phenotype and the Fossil Record. Bull Peabody Museum Nat Hist. 2012;53: 3–308. doi:10.3374/014.053.0101

70. Wiens JJ, Hutter CR, Mulcahy DG, Noonan BP, Townsend TM, Sites JW, et al. Resolving the phylogeny of lizards and snakes (Squamata) with extensive sampling of genes and species. Biol Lett Lett. 2012;8: 1043–1046. doi:10.5061/dryad.g1gd8

71. Hammer Ø, Harper DAT, Ryan PD. PAST: Palaeontological Statistics software package for education and data analysis. Palaeontol Electron. 2001;4: 1–9.

72. Gingerich PD. Arithmetic or geometric normality of biological variation: An empirical test of theory. J Theor Biol. 2000;204: 201–221. doi:10.1006/jtbi.2000.2008

73. Sokal R, Rohlf F. Biometry: The principles and practice of statistics in biological science. San Francisco: W. H. Freeman and Company; 1969.

74. Field DJ, Lynner C, Brown C, Darroch SAF. Skeletal correlates for body mass estimation in modern and fossil flying birds. PLoS One. 2013;8: 1–13. doi:10.1371/journal.pone.0082000

75. Alberdi MT, Prado JL, Ortiz-Jaureguizar E. Patterns of body size changes in fossil and living Equini. Biol J Linn Soc. 1995;54: 349–370.

76. Conrad JL. An Eocene shinisaurid (Reptilia, Squamata) from Wyoming, U.S.A. J Vertebr Paleontol. 2006;26: 113–126. doi:10.1671/0272-4634(2006)26[113:AESRSF]2.0.CO;2

77. Woodburne MO, editor. Late Cretaceous and Cenozoic Mammals of North America: Biostratigraphy and Geochronology. New York: Columbia University Press; 2004.

78. Barnosky AD, Holmes M, Kirchholtes R, Lindsey E, Maguire KC, Poust AW, et al. Prelude to the Anthropocene: Two new North American Land Mammal Ages (NALMAs). Anthr Rev. 2014;1: 225–242. doi:10.1177/2053019614547433

79. Evans SE. The Skull of Lizards and Tuatara. Volume 20,. In: Gans C, Gaunt AS, Adler K, editors. The Skull of Lepidosauria. Volume 20,. Ithaca, NY: Society for the Study of Amphibians and Reptiles; 2008. pp. 1–348.

80. Angilletta, Jr. MJ, Niewiarowski PH, Dunham AE, Leaché AD, Porter WP. Bergmann's Clines in Ectotherms: Illustrating a Life- History Perspective with Sceloporine Lizards.

Am Nat. 2004;164: E168–E183. doi:10.1086/425222

81. ElShafie SJ. Body size and species richness changes in Glyptosaurinae (Squamata: Anguidae) through climatic transitions of the North American Cenozoic. Master's Thesis. Department of Earth & Atmospheric Sciences: University of Nebraska, Lincoln. University of Nebraska, Lincoln. 2014.

82. Meszoely CAM. North American fossil anguid lizards. Bull Museum Comp Zool. 1970;139: 87–150.

83. Gilmore CW. Fossil Lizards of North America. Mem Natl Acad Sci. 1928;22: v–201.

84. Beck DD. Biology of Gila Monsters and Beaded Lizards. 9th ed. Berkeley: University of California Press; 2005.

85. Pianka ER. Evolution of body size: Varanid lizards as a model system. Am Nat. 1995;146: 398–414.

86. Molnar RE. Dragons in the dust: the paleobiology of the giant monitor lizard *Megalania*. Indiana University Press; 2004.

87. Conrad JL, Balcarcel AM, Mehling CM. Earliest Example of a Giant Monitor Lizard (*Varanus*, Varanidae, Squamata). PLoS One. 2012;7: e41767. doi:10.1371/journal.pone.0041767

88. Sullivan RM. The taxonomy, chronostratigraphy and paleobiogeography of glyptosaurine lizards (Glyptosaurinae, Anguidae). Comptes Rendus Palevol. 2019;18: 747–763. doi:10.1016/j.crpv.2019.05.006

89. Scarpetta SG. *Peltosaurus granulosus* (Squamata, Anguidae) from the middle Oligocene of Sharps Corner, South Dakota, and the youngest known chronostratigraphic occurrence of Glyptosaurinae. J Vertebr Paleontol. 2019;39: e1622129.

doi:10.1080/02724634.2019.1622129

90. Pincheira-Donoso D, Meiri S. An Intercontinental Analysis of Climate-Driven Body Size Clines in Reptiles: No Support for Patterns, No Signals of Processes. doi:10.1007/s11692-013-9232-9

91. Piantoni C, Navas CA, Ibargüengoytía NR. A real tale of Godzilla: Impact of climate warming on the growth of a lizard. Biol J Linn Soc. 2019;126: 768–782. doi:10.1093/biolinnean/bly216

92. Kingsolver JG, Huey RB. Size, temperature, and fitness: Three rules. Evol Ecol Res. 2008;10: 251–268.

93. Makarieva AM, Gorshkov VG, Li BL. Gigantism, temperature and metabolic rate in terrestrial poikilotherms. Proc R Soc B Biol Sci. 2005;272: 2325–2328. doi:10.1098/rspb.2005.3223

94. Makarieva AM, Gorshkov VG, Li BL. Temperature-associated upper limits to body size in terrestrial poikilotherms. Oikos. 2005;111: 425–436. doi:10.1111/j.1600-0706.2005.14095.x

95. Pough FH. Lizard Energetics and Diet. Ecology. 1973;54: 837–844. doi:10.2307/1935678

96. Wing SL, Harrington GJ, Smith FA, Bloch JI, Boyer DM, Freeman KH. Transient floral change and rapid global warming at the Paleocene-Eocene boundary. Science (80-). 2005;310: 993–996. doi:10.1126/science.1116913

97. Aureliano T, Ghilardi AM, Guilherme E, Souza-Filho JP, Cavalcanti M, Riff D. Morphometry, bite-force, and paleobiology of the Late Miocene caiman *Purussaurus brasiliensis*. PLoS One. 2015;10: 1–14. doi:10.1371/journal.pone.0117944

98. Sinervo B, Méndez-de-la-Cruz F, Miles DB, Heulin B, Bastiaans E, Cruz MVS, et al.

Erosion of lizard diversity by climate change and altered thermal niches. Science (80-). 2010;328: 894–899. doi:10.1126/science.1184695

99. Gregory KM, McIntosh WC. Paleoclimate and paleoelevation of the Oligocene Pitch-Pinnacle flora, Sawatch Range, Colorado. Geol Soc Am Bull. 1996;108: 545. doi:10.1130/0016-7606(1996)108<0545:PAPOTO>2.3.CO;2

100. Snell KE. Paleoclimate and paleoelevation of the western Cordillera in the United States. University of California, Santa Cruz. 2011.

101. Snell KE, Thrasher BL, Eiler JM, Koch PL, Sloan LC, Tabor NJ. Hot summers in the Bighorn Basin during the early Paleogene. Geology. 2013;41: 55–58. doi:10.1130/G33567.1

102. Huey RB, Slatkin M. Cost and benefits of lizard thermoregulation. Q Rev Biol. 1976;51: 363–384.

103. Wing SL. Eocene and Oligocene floras and vegetation of the Rocky Mountains. Ann Missouri Bot Gard. 1987;74: 748. doi:10.2307/2399449

104. Wing SL, Bown TM, Obradovich JD. Early Eocene biotic and climatic change in interior western North America. Geology. 1991;19: 1189–1192. doi:10.1130/0091-7613(1991)019<1189:EEBACC>2.3.CO;2

105. Stucky RK. Mammalian faunas in North America of Bridgerian to Early Arikareean “Ages” (Eocene and Oligocene). In: Prothero DR, Berggren WA, editors. Eocene-Oligocene Climatic and Biotic Evolution. Princeton, NJ: Princeton University Press; 1992. pp. 464–493.

106. Stamps JA. Rainfall, Moisture and Dry Season Growth Rates in *Anolis aeneus*. Copeia. 1977;1977: 415–419.

107. Wing SL, Greenwood DR. Fossils and fossil climate: The case for equable continental interiors in the Eocene. Philos Trans - R Soc London, B. 1993;341: 243–252. doi:10.1098/rstb.1993.0109

108. Fricke HC, Wing SL. Oxygen isotope and paleobotanical estimates of temperature and d18O-latitude gradients over North America during the early Eocene. Am J Sci. 2004;304: 612–635.

109. Huey RB. Behavioral thermoregulation in lizards: Importance of associated costs. Science (80-). 1974;184: 1001–1003.

110. Lafuma F, Corfe IJ, Clavel J, Di-Poï N. Multiple evolutionary origins and losses of tooth complexity in squamates. Nat Commun. 2021;12: 1–13. doi:10.1038/s41467-021-26285-w

111. Angilletta MJ, Steury TD, Sears MW. Temperature, growth rate, and body size in ectotherms: Fitting pieces of a life-history puzzle. Integr Comp Biol. 2004;44: 498–509.

Supporting information

Materials & methods

Data collection

Almost all the 283 fossil lizard specimens listed in S1 Dataset were measured in person in museum collections. A few measurements were obtained from literature or specimen photos provided by researchers (see “Measurements” below). The fossil dataset was likely biased toward adult individuals because complete anatomical elements are more likely to be preserved from adults. Juveniles are less likely to get preserved as fossils because their bones are more delicate and juveniles are frequently consumed by predators [100]. This preservation bias would not adversely affect the results in this study since it focuses on maximum body sizes.

The 266 extant specimens measured in this study (S2 Dataset) include 86 extant species total and 1-19 individuals sampled per species. This dataset only includes extant species for which reasonably complete dry skeletonized specimens were available. Legless anguids and scincids were omitted because proportions of cranial elements to SVL differ between limbed taxa and serpentiform taxa in extant squamate groups [21] and because all the extinct taxa in the fossil dataset presented here are inferred to have had limbs based on associated limb material or assignment to extant groups with limbs. These data for this study were amassed from 26 different natural history museum collections across the United States (see “Institutional Abbreviations” in the main text).

When collecting extant data, the target sample size was at least n = 20 for each taxonomic group. The sample size was only smaller for Xenosauridae (n = 12; S3 Table), so results for this group should be considered with caution.

Most described diagnostic characters for lizards are found in cranial bones [101,102]. Cranial bones account for most of the curated and published fossil record of lizards. This record is biased toward dentaries and maxillae, which are easy to identify as belonging to a lizard based on dentition and often provide at least coarse taxonomic identifications (family or higher) [102]. Therefore, we can be reasonably confident in taxonomic assignments for fossil lizard cranial bones based on extant phylogenetic morphological characters (although caution should be taken with historical identifications that may lack fidelity, especially at the genus or species level [103–105]).

Fossil specimens were referred to crown groups (family-level or higher) based on specimen label identifications as well as taxonomic information from the Paleobiology Database [106] and current literature. For extinct taxa whose exact placement within or around an extant lizard group remains unresolved, the taxon was assigned to the closest crown group for the purpose of this study. This was the case for three extinct taxa: *Palaeoscincosaurus* and *Contogenys*, both referred to Scincidae [107–110], and *Provaranosaurus*, referred to Shinisauridae. The latter was based on Smith and Gauthier [56], who asserted that the extinct genus *Provaranosaurus* is closely related to extant *Shinisaurus* and placed it within a group called "*Pan-Shinisaurus*." Any such considerations for specific taxa are indicated in the "Notes" column in S1 Dataset.

Measurements

All measurements were taken with digital calipers (Mitutoyo 150 mm) to the nearest 0.1 mm for individual bones and the nearest 1 mm for SVL. For specimens with an SVL measuring > 150 mm, or for specimens that were preserved in a curved position, a tape measure was used to measure SVL to the nearest 1 mm. Only extant specimens for which SVL could be measured

were included, i.e., dry skeletonized specimens with a complete articulated vertebral series. Two xenosaurid specimens with wet-preserved bodies and dry skeletonized skulls (S2 Dataset) were also included because fewer xenosaurid specimens were available and these two specimens still provided the measurements needed. SVL was measured on skeletons as the length from the tip of the snout to the posterior centrum of the second caudal vertebra, which is the approximate position of the middle of the cloaca (vent; see Fig 1). The position of the cloaca on skeletonized specimens was determined by aligning the pelvis of a wet specimen with that of a dry skeletonized congener specimen of comparable size. Whenever a dried skin was included with a skeletonized specimen, it was used to verify the SVL measurement from the vertebral series.

Skull length was measured as the length from the tip of the snout to the most posterior point of the skull (either the occipital condyle or the posterior tip of the supratemporal process). If a cranial bone had bilateral asymmetry (e.g., if on a parietal, one supratemporal process was longer than the other), the longer side was taken to represent the maximum measurement. Each cranial bone was measured from the most anterior to the most posterior point (e.g., on the dentary, from the anterior tip of the ramus to the posterior tip of the retroarticular process). For long bones, only complete bones with epiphyses included were measured.

When it was not possible to access a fossil specimen in person (which was the case for the UMMP specimens and a few specimens measured from figures in literature), measurements were taken from digital photographs with a scale bar in standard orientation using the open access software ImageJ (available online at: https://imagej.nih.gov/ij/download.html). Measurements taken from photographs are indicated in the “Notes” column in S1 Dataset. Measurements taken both in person and from photographs were compared for several extant

specimens to ensure that the measurements taken from photographs were accurate: all measurements proved to be within a margin of error of 5% or less (1-3 mm).

Generating and testing regressions

Regressions were only generated for the anatomical elements represented in the fossil dataset for each specific lizard group (S1-S7 Tables). For example, no isolated fossil varanid femora were measured, so a regression for varanid femur length to SVL was not included (S2 Table).

Each regression was tested for homoskedasticity and for normal distribution of residuals (p(homosked) and p(norm) ≥ 0.05) to validate the assumptions of the regression. These tests were performed using PAST v.4.03 [68].

For each extant lizard group, one specimen with a head length and SVL close to the average in the extant dataset was selected for that group and remove from the dataset prior to calculating the regressions. That removed specimen was then used to test the accuracy of each regression generated from the remaining sample of that extant lizard group (S1-S7 Tables). The samples of extant specimens encompassed the range of body size for most of the crown groups sampled except for Anguidae and Varanidae. The largest taxa in those families were exceptionally large outliers (*Diploglossus millepunctatus* (Anguidae); *Varanus komodoensis* (Varanidae)). Those outliers were therefore omitted from the samples used to generate regressions. However, specimens of those outliers were applied to their respective family regressions as an additional test. The resulting SVL differences were comparable (see Varanidae, S2 Table) or lower (see Anguidae, S1 Table) than the first test specimen, indicating that those regressions can reliably predict SVL for large outliers in those families.

A large specimen of *Heloderma horridum* (Helodermatidae), the skull of which greatly resembles those of the largest anguid fossils in this dataset (Fig 3E), was used as an additional test specimen for the anguid regressions (S1 Table). Helodermatidae is placed close to Anguidae within the group Anguimorpha in both morphological and molecular phylogenetic analyses [61,62,65,66], so it is reasonable to consider Helodermatidae as an extant morphological and close phylogenetic analogue to large extinct anguid lizards. The anguid regressions proved comparably accurate in predicting the SVL of the largest helodermatid test specimen compared to the smaller extant and extinct anguid test specimens.

If a regression for a needed element failed the test for homoskedasticity or normal distribution of residuals, outliers were removed from the dataset for that extant lizard group [67]. The outliers were no larger or smaller than any other taxa included, but their anatomical proportions differed from their congeners. SVL was then estimated for the test specimens with and without the outliers removed. If the predicted values for SVL barely changed, regressions were generated for that group with the outliers removed. This was the case for groups such as Anguidae and Iguania. However, if the new SVL estimate was very different, or if removing clear outliers still did not result in either regression passing the tests for both homoskedasticity and normal distribution of residuals, then the regression was removed completely and fossil specimens that would have relied on that regression were omitted from the study. This resulted in the removal of a handful of fossil specimens known from a single element for which a suitable regression could not be generated. For example, a few fossil varanid specimens known only from a frontal or parietal were omitted.

Minimum and maximum estimates of SVL (95% confidence interval) were calculated for each specimen by subtracting or adding the standard error, respectively, for the slope and interval of the regression used (S1 Dataset, S1-S7 Tables).

If a particular fossil specimen included more than one complete element, the regression that offered the lowest standard error of the estimate (SEE) was used. The regression equations in Table 3 and S1-S7 Tables are ranked in this order. For example, one fossil varanid specimen, FMNH PR 2380, included a complete dentary, maxilla, frontal, and parietal (S1 Dataset). The extant varanid dataset did not yield viable regressions for SVL based on frontal or parietal length, neither of those elements could be used to estimate SVL for that specimen. The varanid regression for maxilla length to SVL yielded a lower SEE than that for dentary length to SVL (S2 Table), so the maxilla length regression was used to estimate SVL for that specimen (S1 Dataset).

Separate regressions for males and females were not generated because most extant specimens measured did not have a sex indicated. Although female lizards often have shorter SVLs than males [4], there is no reliable way to tell the sex of an individual fossil lizard specimen based on isolated bones, so any fossil lizard dataset must be treated as an averaged sample across sexes. The same is largely true for ontogenetic stages represented in a fossil dataset. Adult members of an extinct taxon can be reasonably identified if a large sample of referred individuals that reach a consistent maximum size range is present (e.g., large fossil anguids, S1 Dataset) or if some of those specimens include long bones with fused epiphyses, but some subadult or juvenile specimens may also be present.

An attempt was made to combine measurements from different lizard groups to create "generic" lizard regressions to generate SVL estimates for unidentified lizard elements.

However, none of the resulting regressions passed the necessary tests (see the failed generic equations in S9 Table). Anatomical proportions of the extant data sampled here varied too much between crown groups to produce successful generic regressions.

Special considerations for Iguania

Within extant Iguania, a representative range of the body size, morphology, and taxonomic diversity within pleurodontans was sampled because all the fossil iguanian specimens measured had pleurodont dentition but not all were identified to a specific pleurodontan family. *Amblyrhynchus* and *Crotaphytus* were not included because they were outliers with respect to head length : SVL ratio compared to the rest of the extant iguanian dataset (0.14 for *Amblyrhynchus* and 0.26 for *Crotaphytus* compared to an average of 0.21 for the other genera).

Special considerations for Shinisauridae

No complete dry skeletonized specimens of extant *Shinisaurus* were available to measure. Only one dry skull and four wet preserved specimens of this monotypic family were found. However, the holotype and only known complete skeleton of an extinct taxon referred to Shinisauridae, *Bahndwivici ammoskius* (FMNH PR 2260, Fig 5A) [111] was available. The *B. ammoskius* holotype skeleton had the same head length : SVL ratio as the average calculated for extant *Shinisaurus* based on wet preserved specimens (0.23 vs. 0.22, n = 4). Therefore, ratios of individual cranial elements to SVL from the *Bahndwivici* skeleton were used to estimate SVL for the handful of fossil specimens referred to Shinisauridae (S1 Dataset).

Estimating body mass

The equation provided by Feldman et al. **[42]** was used for Shinisauridae because an equation for Shinisauridae was not provided and Shinisauridae falls within Anguimorpha. The equation for Fully Legged Anguidae was used from the same reference for the fossil anguid

lizards because limb material has been found with many fossil anguid specimens and none have been interpreted as limbless.

Discussion

Taxonomic richness

No more than two distinct species were documented for any given genus in the fossil dataset (S1 Dataset). Many fossil specimens could not be identified with certainty past the level of genus (e.g., *Melanosaurus sp*.). There is no way to know exactly how many different species might be represented by these specimens, even if only one diagnostic species is recognized within a given extinct genus (e.g., *Melanosaurus maximus*). Many of the extinct genera in this study that included only one diagnostic species almost certainly included other unidentified species as well. For example, the extinct anguid genus *Melanosaurus* had greater variation in SVL than any individual extant anguid species (standard deviation = 222 vs. 34 (*Gerrhonotus liocephalus*); S1 and S2 Datasets). In general, it is much more difficult to discern diagnostic species within a genus than it is to parse genera within a clade based on fossils [112]. In this fossil dataset, 88% of specimens were identified to the level of genus but only 42% were referred to a species. This may account for some of the discrepancies between the data reported here and the data available in the Paleobiology Database (Table 2).

However, this is unlikely to affect the patterns of taxonomic richness observed here. Even in extant lizards, elevated richness usually reflects a high number of genera with a low number of species per genus, possibly because lizard genera within families are more ecologically distinct from each other than are species within genera [3]. Thus, in lieu of trying to estimate the actual number of extinct species sampled, tracking richness at the level of genera and higher taxonomic

Formatted: Font: 11 pt

Commented [SE1]: SD of 222? Are these values correct (size corrected)?

groups (S3 Fig) is likely to capture a sufficient representation of the taxonomic diversity in this geotemporal system.

Supporting Captions

S1 FigS1 Fig. Protocol for estimating snout-vent length of fossil lizards.

S2 Fig. Regressions of dentary length to snout-vent length for all extant lizard groups sampled. Shinisauridae is not included here because no regressions were generated for that group (see "Special Considerations for Shinisauridae" in Supporting Information). See Table 3 for regression equations and statistics. All regressions are transformed using natural log.

S3 Fig. Rarefaction curve showing the number of expected crown taxonomic groups for NALMA sample sizes within the fossil lizard dataset. S1 Dataset was subsampled here by North American Land Mammal Age (NALMA) interval. NALMA abbreviations: Pu = Puercan, To = Torrejonian, Ti = Tiffanian, Cf = Clarkforkian, Wa = Wasatchian, Br = Bridgerian, Ui = Uintan, Du = Duchesnean, Ch = Chadronian, Or = Orellan, Wh = Whitneyan, Ar = Arikareean. Individual rarefaction analysis was performed using PAST v4.03 [71].

S2 Fig. Mean and maximum body length in fossil lizards by taxonomic group per NALMA. Body length is measured as snout-vent length (SVL) in mm. All measurements were transformed using natural log (LN). Dotted line = maximum. Dashed line = mean. Lines do not connect with the single Duchesnean datapoint since it likely does not accurately represent a maximum or mean value for that NALMA. North American Land Mammal Age (NALMA) abbreviations: Pu =

Puercan, To = Torrejonian, Ti = Tiffanian, Cf = Clarkforkian, Wa = Wasatchian, Br = Bridgerian, Ui = Uintan, Du = Duchesnean, Ch = Chadronian, Or = Orellan, Wh = Whitneyan, Ar = Arikareean.

S3 Fig. Mean and maximum body mass in fossil lizards by taxonomic group per NALMA. Mass estimates were calculated from snout-vent length (SVL) using the published equations listed in S8 Table. All measurements (in kg) were transformed using natural log (LN). Dotted line = maximum. Dashed line = mean. Lines do not connect with the single Duchesnean datapoint since it likely does not accurately represent a maximum or mean value for that NALMA. North American Land Mammal Age (NALMA) abbreviations: Pu = Puercan, To = Torrejonian, Ti = Tiffanian, Cf = Clarkforkian, Wa = Wasatchian, Br = Bridgerian, Ui = Uintan, Du = Duchesnean, Ch = Chadronian, Or = Orellan, Wh = Whitneyan, Ar = Arikareean.

~~S4 Fig~~S4 Fig. Rarefaction curve showing the number of expected size groups for NALMA sample sizes within the fossil lizard dataset. To conduct this analysis, each fossil specimen in S1 Dataset was assigned to one of 11 size groups, each group representing a bin of 100 mm (e.g., 1-99 mm, 100-199 mm, 200-299 mm, etc.; the last group was ≥ 1 m). S1 Dataset was subsampled here by North American Land Mammal Age (NALMA) interval. The y-axis in this graph shows the total number of size groups recovered in the sample for each NALMA interval. NALMA abbreviations: Pu = Puercan, To = Torrejonian, Ti = Tiffanian, Cf = Clarkforkian, Wa = Wasatchian, Br = Bridgerian, Ui = Uintan, Du = Duchesnean, Ch = Chadronian, Or = Orellan, Wh = Whitneyan, Ar = Arikareean. Individual rarefaction analysis was performed using PAST v4.03 [71].

S5 FigS5 Fig. Global temperature vs. lizard body size by NALMA. A) Global marine mean annual paleotemperature (MAPT) vs. maximum lizard SVL. Function: LN(Global Marine MAPT) = ((1.23±0.401)*LN(Max Lizard SVL)) + (3.70±0.792), R^2 = 0.04, p(uncorr.) = 0.56, SEE = 0.991. **B)** Global marine MAPT vs. mean lizard SVL. LN(Global Marine MAPT) = ((-0.910±0.303)*LN(Mean Lizard SVL)) + (7.06±0.606), R^2 = 0.0004, p(uncorr.) = 0.95, SEE = 0.812. Temperature data are from Zachos et al. [44] and were taken from the NALMA midpoint in ºC. All data were transformed using natural log. Regression analysis was performed using PAST v4.03 [71].

Formatted: Font: Italic

Formatted: Font: Italic

S6 Fig. Mean and maximum snout-vent length in fossil lizards by taxonomic group per NALMA. Dotted line = maximum. Dashed line = mean. Lines do not connect with the single Duchesnean datapoint since it likely does not accurately represent a maximum or mean value for that NALMA. All measurements were transformed using natural log.North American Land Mammal Age (NALMA) abbreviations: Pu = Puercan, To = Torrejonian, Ti = Tiffanian, Cf = Clarkforkian, Wa = Wasatchian, Br = Bridgerian, Ui = Uintan, Du = Duchesnean, Ch = Chadronian, Or = Orellan, Wh = Whitneyan, Ar = Arikareean. All measurements were transformed using natural log.

S7 Fig. Mean and maximum body mass in fossil lizards by taxonomic group per NALMA. Dotted line = maximum. Dashed line = mean. Lines do not connect with the single Duchesnean datapoint since it likely does not accurately represent a maximum or mean value for that NALMA. North American Land Mammal Age (NALMA) abbreviations: Pu = Puercan, To =

~~Torrejonian, Ti = Tiffanian, Cf = Clarkforkian, Wa = Wasatchian, Br = Bridgerian, Ui = Uintan, Du = Duchesnean, Ch = Chadronian, Or = Orellan, Wh = Whitneyan, Ar = Arikareean. All measurements were transformed using natural log.~~

S1 Table. Anguidae regression equations and tests. Sample sizes indicate number of extant anguid specimens that included the element used in the given regression. Regression equations are ranked in order of increasing standard error of the estimate (SEE). Minimum and maximum SVL estimates for test specimens are based on the standard errors for the slope and intercept of the given regression. Equations for body mass from individual bones are based on the relevant equation listed in S8 Table.

S2 Table. Varanidae regression equations and tests. Sample sizes indicate number of extant varanid specimens that included the element used in the given regression. Regression equations are ranked in order of increasing standard error of the estimate (SEE). Minimum and maximum SVL estimates for test specimens are based on the standard errors for the slope and intercept of the given regression. Equations for body mass from individual bones are based on the relevant equation listed in S8 Table.

S3 Table. Xenosauridae regression equations and tests. Sample sizes indicate number of extant xenosaurid specimens that included the element used in the given regression. Regression equations are ranked in order of increasing standard error of the estimate (SEE). Minimum and maximum SVL estimates for test specimens are based on the standard errors for the slope and

intercept of the given regression. Equations for body mass from individual bones are based on the relevant equation listed in S8 Table.

S4 Table. Teiidae regression equations and tests. Sample sizes indicate number of extant teiid specimens that included the element used in the given regression. Regression equations are ranked in order of increasing standard error of the estimate (SEE). Minimum and maximum SVL estimates for test specimens are based on the standard errors for the slope and intercept of the given regression. Equations for body mass from individual bones are based on the relevant equation listed in S8 Table.

S5 Table. Scincidae regression equations and tests. Sample sizes indicate number of extant scincid specimens that included the element used in the given regression. Regression equations are ranked in order of increasing standard error of the estimate (SEE). Minimum and maximum SVL estimates for test specimens are based on the standard errors for the slope and intercept of the given regression. Equations for body mass from individual bones are based on the relevant equation listed in S8 Table.

S6 Table. Xantusiidae regression equations and tests. Sample sizes indicate number of extant xantusiid specimens that included the element used in the given regression. Regression equations are ranked in order of increasing standard error of the estimate (SEE). Minimum and maximum SVL estimates for test specimens are based on the standard errors for the slope and intercept of the given regression. Equations for body mass from individual bones are based on the relevant equation listed in S8 Table.

S7 Table. Iguania regression equations and tests. Sample sizes indicate number of extant iguanian specimens that included the element used in the given regression. Regression equations are ranked in order of increasing standard error of the estimate (SEE). Minimum and maximum SVL estimates for test specimens are based on the standard errors for the slope and intercept of the given regression. Equations for body mass from individual bones are based on the relevant equation listed in S8 Table.

S8 Table. Published equations for estimating lizard body mass from snout-vent length. These equations were used to estimate mass for fossil lizards as listed in S1 Dataset and to generate equations for estimating body mass from individual bones (S1-S7 Tables).

S9 Table. Tests of general regression equations. Sample sizes indicate number of extant specimens that included the element used in the given regression. Regression equations are ranked in order of increasing standard error of the estimate (SEE). None of these equations passed tests for both homoskedasticity and normal distribution of residuals, thus, none of these equations were used in this study.

S10 Table. Mean and maximum fossil lizard snout-vent length and body mass by taxonomic group per NALMA. These natural log-transformed data were used to generate the graphs in S2 Fig and S3 Fig.

S11~~0~~ Table. Data for correlating SVL with temperature. Temperature data came from Zachos et al. [44] and were taken from the NALMA midpoint in ºC. The Duchesnean was not included because the maximum SVL estimate for that NALMA was exceptionally low and based on a single data point.

~~**S11 Table. Mean and maximum fossil lizard snout-vent length and body mass by taxonomic group per NALMA.** These natural log-transformed data were used to generate the graphs in S6 Fig and S7 Fig.~~

S1 Dataset. Fossil lizard data. Measurements, locality information, and body size estimates for all fossil lizard specimens sampled. Family-specific mass was calculated from equations listed in S8 Table [24,41,42]. General mass estimate (across all lizard groups) was calculated using the embedded equation from Meiri [24].

S2 Dataset. Extant lizard data. Data for all extant lizard specimens measured. ~~Measurements taken from photographs using ImageJ are indicated in bold font.~~

October 6, 2023

Dear PLoS One Editorial Board,

Thank you for the opportunity to make these additional revisions to my manuscript. Below is a list of the new changes that I made.

Overall Changes Made:

- I revised the Introduction to make it more concise.
- I heavily reworked the Materials and Methods section to be sufficiently detailed and more cohesive.
- I took the text that had been moved to Supporting Information, distilled it, and incorporated it back into the main text.
- I added mean values to Table 4 to provide the full data visualized in Fig 6, Fig 7, S6 Fig, and S7 Fig. I also revised the captions for those figures.
- I removed all material related specifically to taxonomic turnover. This included removal of the former Table 2 and S3 Fig, which were no longer relevant.

I made all changes suggested by the Editor and Reviewers, with the following minor exceptions:

Editor comments and edits (lines refer to previous version of the manuscript)

- Line 18: With respect to the Editor's comment, I did not indicate that lizards are more vital to extant ecosystems than pollinating insects or any other group, only they that they are important. I elaborate on this with citations in the Introduction. I maintain that this is a reasonable and appropriate opening sentence for this abstract.

- Lines 32-35: I like the more concise phrasing suggested here. However, the femur was only possible to regress for Anguidae, not the other lizard groups, so I omitted that mention.

- Lines 159-160: I think the justification for the chosen study system is best placed in the Introduction, and does not need to be reiterated in the Materials & Methods section.

- Line 370 – "*do you mean correlation r????*": I mean the coefficient of determination for the correlation, R^2. I think this sentence was confusing as written, so I revised it.

- Lines 395-406: With respect, I do not think that this information is off topic. It helps to explain why we might be seeing the observed patterns even though the maximum body sizes to not correlate with global temperature data. I left this information in the "Climate as a potential driver of body size evolution" section of the Discussion, but I did revise that section to be more cohesive.

- *The section Appendix should be part of supporting material [Appendix S1] and needs to be more in line with PlosOne style and nomenclature (https://journals.plos.org/plosone/s/supporting-information).*
    - I am not sure what the Editor meant by this comment because there was no section in the manuscript labeled "Appendix." If they were referring to the supporting datasets (S1 and S2 Datasets), I see no reason to change the file name because "Dataset" is listed as one of the common item descriptions in the Supporting Information guidelines link provided by the Editor. Those files are datasets, and I would prefer to designate them as such for clarity. Furthermore, I did not see any instructions in the linked guidelines as to a required format for dataset files. If the Editor would like to request specific changes to the formatting of S1 and S2 Datasets with explanations for the needed changes, I would be happy to make those changes.

Reviewer #3 comments and edits (lines refer to previous version of the manuscript)

- Lines 234-238: Log was not written out as "logarithm" in the PLoS One publication that the Editor recommended as a formatting reference (Field et al. 2013), and "log" is the more common term used, so I did not see the need to write it out here.

- Lines 267-272: I agree that the explanation of how I derived new equations for estimating body mass was not very clear as written. I revised it to be more coherent. However, I decided to stick with the existing arrangement of explaining the equations that had already been published and then explaining how I derived new equations because I had to use the existing published equations to generate the new equations.

- Line 419: Correct, mass cannot be zero, but the mass listed here is 1kg, with a natural log of 0.00.

- *I would suggest starting the data collection section by saying the total number of collected specimens and the number of genera they represent.*
    - I understand what the reviewer is getting at – it would be confusing to only discuss the fossil data but not the extant data before presenting a table that offers both fossil and extant data. However, this is just an artefact of the formatting for the submitted manuscript. Since the tables will be separated from the text in the final layout, this will no longer be an issue. I think the text flows better with the details for the fossil data presented together in one paragraph, followed by the details for the extant data in the next paragraph. But I also revised the entire Data Collection section to improve its clarity.

- *Rows 227-229: I suggest putting this sentence in line with rows 232-235 sayings that although bivariate regression is frequently used, you chose to utilise RMA instead, and then discuss the variables you employed.*

    - RMA is a form of bivariate linear regression, not an alternative to it. I added the word “bivariate” after RMA to clarify this for anyone who is not familiar with RMA regressions.

- *It is not very clear from the main manuscript that the fossil dataset includes also juveniles, in my opinion.*
    - There was a brief mention of this in the Methods, and the SI included commentary on why juveniles were not included in the extant dataset and why they were likely not represented in the fossil dataset. I revised that commentary and moved it back to the Methods section in the main text.

Thank you again for the additional time that you, the Editor, and the Reviewers have contributed to reviewing this paper, and for your patience as I incorporated the suggestions into the manuscript. I look forward to hearing back from you.

Best regards,

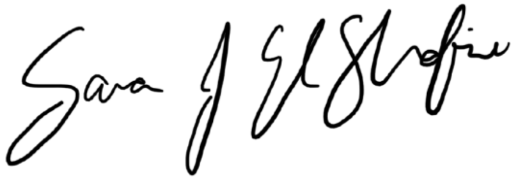

Sara J. ElShafie, Ph.D.
Alumnus, Department of Integrative Biology and Museum of Paleontology
University of California, Berkeley
selshafie@berkeley.edu
