# Supplementary material for: Body size estimation from isolated fossil bones reveals deep time evolutionary trends in North American lizards
Source: PLoS One. 2024 Jan 5;19(1):e0296318. doi: 10.1371/journal.pone.0296318 (PMC10769094; doi:10.1371/journal.pone.0296318)
Supplement: Attachment — Submitted filename: Review_PONE-D-22-04244_V1.docx [file pone.0296318.s020.docx]

Dear author,

I enjoyed reading this paper and I think that it is an excellent contribution to the field, and it is appropriate for this journal. The manuscript is well written and presents useful data and methodology for squamate paleontologists and biologists, which I imagine will also be useful to researchers studying other vertebrate taxa. The results specific to North American fossil lizards are very interesting and will no doubt impact broader thinking on lizard body size evolution during the Paleogene. I have a number of comments, most of which are relatively minor (phrasing, precision or accuracy of certain terms), and a handful of which will require more thought (adding in background for the focal clades in the introduction and caveats to the identifications of a few fossils in the dataset, potentially making a flowchart or workflow figure for “Protocol for estimating fossil lizard body size,” changes to discussion of glyptosaurine diet). Overall though I think that the paper is in great shape and I am looking forward to seeing it published.

Comments and suggestions:

Line 42: you might consider a citation after “taxonomic precision” or at least clarify what you mean- species level precision? Genus level? Family level?

Line 78: In general, I would use “extinct taxa,” “extinct species,” “extinct species known from fossils,” “extinct taxa known from fossils” or even just “fossils” instead of “fossil taxa,” and this term should be removed (in my opinion) here and everywhere else in the manuscript it is used. Fossil taxa is an imprecise/catch-all term, because it could refer to extant species that are known from fossils and living organisms, extinct species known only from fossils (which I think is what people generally mean), or extinct taxa above the species level known only from fossils. Probably missing other potential meanings.

Would “extinct species” or “extinct species known from fossils” work here?

Line 92: Framing of this study with respect to “crown lizard groups” (here and elsewhere)– do you mean crown group lizards broadly (anything that is part of crown Squamata that is not a snake) or only crown groups of crown groups (ie no stem iguanians or stem anguids)? Specific to this paper, it is not clear whether glyptosaurines are in crown Anguidae (see Gauthier et al. 2012 and Conrad et al. 2011). Moreover, saying “crown lizard groups” is in and of itself a little funny given that lizards are a grade and not a clade, although I understand what you mean if the intention is crown squamates excluding snakes. I am pretty sure you mean crown squamates that are lizards, but I would appreciate some clarification.

Something to add that I think would help this confusion and that would improve the introduction generally (and perhaps make your paper stand out more to herpetologists not as familiar with the fossil record) would be to add more background for each of the lizard groups that you are studying. Obviously, the individual groups are not the focus of this paper and so it would not be appropriate to lead with this, but I think moving into some additional background of your study system towards the end of the introduction would be useful. Even a brief mention would be nice (“I sample lizards from the Paleogene record (23-66 Mya) in the Western Interior of the United States. This includes diverse and ecologically important extant clades that are still found in the Western Interior, such as pleurodontan iguanians (e.g., spiny lizards and collared lizards), and clades that were extirpated during the Paleogene, such as varanid lizards). But I think you should put more, especially since you have these great figures illustrating the different taxa, but then no background about the taxa.

Listing out all of the families on line 179-180 cleared this up for me more or less (but glyptosaurines are still potential stem members of an extant family), so I still think early clarification and additional background would help.

Line 92: “This fossil record is extensively sampled”

I think this claim seems is a reach as currently phrased- maybe “There are many known fossil lizard specimens represented in…”

Also, well sampled in total specimens maybe, but unevenly sampled through time and per lineage. You have many specimens from each clade during the Eocene but mostly glyptosaurines from the Oligocene and Paleocene. Here and elsewhere (I have some other comments about this) I think you need to be more explicit that you have an exceptional sample of glyptosaurines throughout most of the dataset, but other clades are not as well sampled in some NALMAs and some NALMAs are poorly sampled generally (this latter point you did draw attention to).

Line 99: “Since the Paleogene” I think implies Paleogene through the present, i.e., also including the Quaternary and the Neogene, but you didn’t look at those fossils. Maybe rephrase to “I hypothesize that proportions of individual bone lengths to SVL are conserved within each lizard lineage and can therefore be used to predict SVLs for fossil lizard specimens identified to those same groups,” or something like that.

Line 108-109: Yes!!!!

Line 138-139: Completely agree that NALMAs are useful for establishing time (both relative and absolute), but they by definition compose a relative time scale that has varying degrees of external age control. I would change “which is the conventional time bin” to “which compose a relative chronology,” or some other phrasing that does not equate NALMAs directly to absolute time.

Figure 2: I love this figure!

Line 169: You call *Afairiguana avius* part of ‘Iguanidae’ here but use the family designation elsewhere (ie line 269, *Afairiguana avius* is called a ‘polychrotid’). I am agnostic as to what terms are used as long as they are consistent. *Afairiguana avius* is either part of Iguanidae and Polychrotinae *or* Pleurodonta and Polchrotidae; it can’t be both Iguanidae and Polychrotidae.

Line 182: I suggest “extinct taxa” instead of “fossil taxa”.

Line 184: “Pleurodontan” is a better term than “pleurodont iguanian.” The former references a specific clade but the latter also makes an implication about tooth morphology in iguanians that is not true (some acrodontans have pleurodont teeth on the premaxilla and even the maxilla/dentary, and with stem taxa it gets even messier. I think some folks don’t like ‘Pleurodonta’ or ‘Acrodonta’ either but those clade terms are fine in my opinion)

Line 192: I would add a word, “Most described diagnostic characters”

Line 193: Most of the published fossil record? Most of the collected fossil record? I think the most common lizard fossils in collections could be osteoderms (you can open any given vial and find >100 body osteoderms), which are not diagnostic at the level you are talking about.

I think it’s more that cranial fossils, particularly tooth-bearing bones, are easy to identify as belonging to a lizard and so those fossils are often picked from matrix and diagnostic characters are then described for them, not that we know with certainty that they are the best possible bones for identifying the extinct taxa (although I agree that cranial bones broadly are often the most useful). You might emphasize that the dentary and maxilla in particular have been frequently identified from collected fossil material and thus published/labelled in museum collections. I would also cite/look at Woolley et al. 2022 (A biased fossil record can preserve reliable phylogenetic signal) for this discussion.

Lines 193-194: See comments on supplementary material, I have reservations about the fossil “gerrhonotines.” More generally, I think you should mention caveats to historical identifications of fossil lizards (Bell et al. 2010, Covert biases, circularity, and apomorphies: A critical look at the North American Quaternary Herpetofaunal Stability Hypothesis; written about the Quaternary obviously but applies here too)

Line 197: “extinct taxa” instead of “fossil taxa”.

Line 196-202: Good that this information is somewhere, but you should mention here how many extinct taxa have uncertain placements in the main text.

Lines 212-213: Since Helodermatids are anguimorphs not anguids, you either need a new section for this group or the title of the “Special Considerations for Fossil Anguids” section should be amended.

Lines 214-220: I think generally the measurements you took are straightforward. But some of them, especially those for individual bones, are potentially ambiguous and I think it would be good to provide clarification on the lengths that you measured. For example, for the dentary did you measure from the anteriormost point (the ramus) to the posteriormost point (probably the coronoid or surangular process), or did you measure a line that is perpendicular to the anteroposterior axis of the dentary (this is assuming a measurement in dorsal view). Is each measurement always the maximum possible length? What did you do if there was bilateral asymmetry (for example, one supratemporal process of the parietal was longer than the other one).

Line 230: “accommodates error in both the dependent and independent variable measurements.” Is a citation needed here?

Line 265: I’d say “extinct” instead of “fossil”

Line 314-316: Thank you for drawing attention to this limitation!! Important to point this out (for this study but for lizard paleontology in general)

Line 317-319: Size is one factor used to determine ontogeny, but there are others as well (terminal fusions of the braincase or mandible, shape of the parietal or supraoccipital, fusion of osteoderms to the skull, presence of osteodermal crust on frontal/parietal or high degree of rugose texturing or sculpturing) (Bhullar 2012; A Phylogenetic Approach to Ontogeny and Heterochrony in the Fossil Record: Cranial Evolution and Development in Anguimorphan Lizards (Reptilia: Squamata)).

Line 329-330: I would say “extinct members” or even more precisely “extinct members known from fossils” instead of “fossil members.” Similar to “fossil taxa,” “fossil members” could refer to extinct species known from fossils, or extant species that are known from both fossils and living organisms.

Line 335: *Peltosaurus* is a glyptosaurine of course, I think you need to mention that here.

Line 344: “nearly equal” instead of “equal”

Line 354-355: I would put “*Celestus*” as the genus outside of the parentheses for both of these; *Comptus* and *Siderolamprus* are not currently recognized to my knowledge

Line 381-384, 386-387: What about the other ratios?

Line 392: Change “had” to “have”

Line 400: “fossil taxon” to “extinct taxon”

Lines 408-410: I might be missing something, but how did you calibrate this?

Line 413: Potentially due to the venom apparatus or pretty apprehension strategy of helodermatids?

Line 418: “more so than proportions of head or limb length to SVL”

Is there a citation to support this?

Lines 437-438: This is an unrealistic assumption, it is fair to make it here but I would change “might” to something more conclusive.

Line 444-447: I am likely missing something but I am not following this line of reasoning

Line 455: “I did not have time to collect a representative sample of extant Acrodontan diversity” Fair enough and appropriate to put in the PhD document, but for the paper I would delete this. You might also mention the uncertain taxonomic allocation of those acrodontans, which could affect your analyses. Illaria Paparella’s 2021 dissertation suggests that *Tinosaurus* is a chameleon and Krister Smith in a 2011 paper (On the phylogenetic affinity of the extinct acrodontan lizard tinosaurus) posited that *Tinosaurus* may be closely related to *Leiolepis*; those are very differently shaped lizards.

Line 456-459: You use the family-level names for the major pleurodontan clades here, so maybe stick with that?

Line 460: In what way were those two taxa outliers? I am not surprised about *Amblyrhynchus* but I am about *Crotaphytus*

Line 467: Use pleurodontan

Line 473: *Bahndwivici ammoskius*- included in the crown group of what? Because extant Shinisauridae is monotypic, is the crown group not then restricted to *Shinisaurus*? Seems to me that *Bahndwivici ammoskius* is the sister of the crown group

Line 489: “Protocol for estimating fossil lizard body size”

I really like this! It could be even more accessible as a table or a flowchart. Actually, having this section as a flowchart in the methods would be nice and perhaps it would be more appropriate to include this information there, and then a summary here? Or a summary there and the flow chart here?

Line 563-564: Is this a table caption or a footnote?

Change “and it very small” to “and it is very small”

I would change this phrasing to “I did not list the single specimen from the Duchesnean because the max SVL and mass estimates for that specimen are substantially lower than all other maxima in the dataset” or something. Could also be worth putting how much smaller that fossil is

Line 570: Change “The rarefaction analysis in S1 Fig shows” to “The rarefaction analysis (S1 Fig) demonstrates”

Line 583: “taxonomic diversity was greatest in the early Eocene”

As you point out later this study is not comprehensive with respect to published Paleogene lizard diversity. You did perform rarefaction analysis so at the level of the 7 major clades you sampled this may be true, but I would be more explicit about that (ie, we don’t know about species or genus level diversity for the clades you sampled based on the data you presented).

Line 584: “decreased along with maximum body size range in the early Oligocene”

Just wanted to say that on the whole this is a very interesting result!

Line 597: I think instead of “Old World” you could say “eastern hemisphere”, but up to you.

Line 598: Most abundant in terms of known specimens, known species richness, or true species richness?

I would probably say known species richness. Fossil pleurodontans can be very difficult to identify and high species richness could be impossible to detect from available fossil (dentaries and maxillae), but even with those difficulties many Paleogene fossil pleurodontans have been successfully identified and extinct species described (several of which you include but only one or two specimens of each when many more are known).

Line 604-605: Tschopp et al 2022 (How to Render Species Comparable Taxonomic Units Through Deep Time: A Case Study on Intraspecific Osteological Variability in Extant and Extinct Lacertid Lizards) would be a good paper to cite here, and also to potentially flesh out discussion of the meaning of genera and species in the fossil record

Line 620: “xantusiids, and iguanians have since been extirpated from the Western Interior”

There are many extant, native pleurodontan iguanians in the modern western interior of the US (phrynosomatids, crotaphytids, even green anoles are found in the study area in the modern day!). Perhaps you meant that the specific iguanians that occurred there during the Paleogene have since been extirpated? Xantusiids are no doubt most abundant adjacent to the Pacific coast, but they do occur far inland in Arizona and Utah well-within your study area. Correct this statement for both groups

For reference, there are maps of all extant North American lizards in Scarpetta et al. 2020 (Evolution of North American lizards) or you could just look at iNaturalist

Line 626: Clarify what you mean by most complete- most known taxa, most specimens published, most specimens in museum collections clearly identified to genera, some combination of things, etc

Also, there many fossil lizards have been described since 1982 (ref 59).. is this still true?

Lines 663-664: The morphospace analyses from Herrera-Flores et al. found a complete overlap between durophagy and herbivory (and even included insects in the category you mentioned) so I do not think this reference would support durophagy for the teeth of glyptosaurines, or at the very least, it would suggest durophagy and/or herbivory.

Line 680: Is there a published citation for this claim? It sounds reasonable but is something that I would prefer to be backed up by published research

Line 680-682: I agree that this is probably true, but I am not sure you should make that claim here when you have not done any dental microwear or other analyses of glyptosaurines (and no one else has either to my knowledge)

Line 691-692: Again, not sure about this claim or that you could call it a consensus. The studies by Gauthier (1982) and Sullivan (1979) are very nice works but the glyptosaurine dietary inferences are based on brief comparison to earlier work (Hotton 1955, which was about pleurodontans specifically, and Pough 1973) and the mention of large Australian skinks. I think a better reference to think about for building these arguments would be Melstrom (2017) which you cite several lines later.

I think generally you need to be much more tentative about the potential diet of glyptosaurines.

Line 696: Flared tricuspid teeth are sometimes characteristic of herbivory in iguanids in particular, not tricuspid teeth in general or even iguanians in general. Many pleurodontans that have flared tricuspid teeth are not herbivores (e.g., *Leiocephalus*, some tropidurids, some corytophanids)

In my opinion you need to emphasize for all diet discussions that, while it makes a lot of sense, attribution of a durophagous diet to glyptosaurines is at this point speculative

Line 707-708: Is there anything to suggest that glyptosaurines were not semiaquatic (at least some of them)? At least several large, extant durophagous lizards are semiaquatic (*Draecana*) or comfortable in water (*Varanus niloticus*). I don’t mean to suggest that glyptosaurines are semiaquatic, but if you are pursuing a discussion/hypothesis of durophagy I think it’s something you have to address.

Line 716: Change “insectivorous mammals in the” to “insectivorous mammals known from the”

Line 719: Change “any mid-sized glyptosaurines consuming mostly snails” to “any mid-sized glyptosaurines that potentially consumed snails”

Line 733: Change “was likely” to “may have been”

Line 744: What does having osteoderms have to do with diet? Remove that part of sentence unless you discuss this specifically

Line 748-750: That is so cool!

Line 764-Line 772: This discussion of *Varanus komodoensis* needs work. *Varanus komodoensis* is armored with ostederms, although the morphology of the “chain-mail” is no doubt very different from glyptosaurines (Maisano et al. 2019; The Cephalic Osteoderms of Varanus komodoensis as Revealed by High-Resolution X-ray Computed Tomography). The authors of that study speculated based on the lack of osteoderms in juvenile *Varanus komodoensis* that the osteoderms of adult *Varanus komodoensis* protected individuals from confrontations with other *Varanus*. Do we know anything about osteoderm morphology in juvenile glyptosaurines? Protection from predation seems like a reasonable hypothesis, but I would cite more literature here and provide additional discussion of glyptosaurine osteoderms in that context (e.g., crocs, English 2018, Evolution of Functional Morphology of Osteoderms Across Crocodylomorpha; lizards, Williams et al. 2021, A review of the osteoderms of lizards (Reptilia: Squamata)

Line 802-805: Well put, this is an important consideration for the study and glad to see you leading with it here.

Line 806: See above comment. I am not sure that I would describe the sample as representative. For example you included several pleurodontan iguanians, but most described (much less known) pleurodontan fossils from the Paleogene western interior were not included, given your study design. The sample is skewed towards glyptosaurines (186 out of 284 fossils), which is ok, but you should be clear about that

Line 807: Agreed.

Line 841: “which appear to have” to “which may have.” Also, this sentence just lists most of the possible diets for lizards as paths to gigantism, so I am not sure that it really needs to be said. Sounds more like we don’t know the association between diet and gigantism in glyptosaurines.

Line 844: “fossil taxa” to potentially “extinct taxa”

Supplemental material:

Specimens SMM P76.28.64, SMM P76.28.69, SMM P76.28.73, SMM P77.7.158.3. I doubt the accuracy of the allocation of these middle Paleocene fossils to Gerrhonotinae. There are well-known issues with the morphological diagnoses of many if not most fossil gerrhonotines especially the early Cenozoic or Cretaceous fossils (see Good 1988; The phylogenetic position of fossils assigned to the Gerrhonotinae (Squamata: Anguidae)). Moreover, these are fossil dentaries; it is not impossible to differentiate the dentaries of gerrhonotines from other anguids, but those characters will generally pertain to crown members of Gerrhonotinae (see Scarpetta et al. 2021, A new extinct species of alligator lizard (Squamata: Elgaria) and an expanded perspective on the osteology and phylogeny of Gerrhonotinae), not characters that could unambiguously differentiate gerrhonotines from diploglossines or vice versa. So without any other material to make a secondary association, I think that considering these fossils to be gerrhonotines would be in error.

Specimens YPM VP 16778, PU 1932, UCMP 47740. Similarly, it is in my opinion very unlikely that these fossils are referable to the current construction of the clade *Gerrhonotus*, much less Gerrhonotinae in general (see above). Those fossils were likely assigned to *Gerrhonotus* when that genus still contained *Elgaria* based on characters common to those two genera (which together constitute a paraphyletic assemblage of alligator lizards). Again, these are fossil dentaries, and I don’t think that dentaries of *Elgaria* and *Gerrhonotus* are easily differentiated, and it can be difficult to differentiate gerrhonotine dentaries from other anguids (Ledesma et al. 2021, Variation in the skulls of Elgaria and Gerrhonotus (Anguidae, Gerrhonotinae) and implications for phylogenetics and fossil identification; Scarpetta et al. 2021).

I don’t think this changes your analysis at all, but you should put a note about this somewhere and/or potentially change the labels in the supplementary material to Anguidae indet.
