# Supplementary material for: Body size estimation from isolated fossil bones reveals deep time evolutionary trends in North American lizards
Source: PLoS One. 2024 Jan 5;19(1):e0296318. doi: 10.1371/journal.pone.0296318 (PMC10769094; doi:10.1371/journal.pone.0296318)
Supplement: Attachment — Submitted filename: Response to Reviewers.docx [file pone.0296318.s021.docx]

Dear PLOS One Editorial Board,

Thank you for the opportunity to make these revisions to my manuscript. Below is a list of the changes that I made.

In response to the Journal Requirements:

1. I reformatted the title page in exact accordance with the PLOS One formatting requirements. I also double-checked that the rest of the manuscript is formatted correctly.
2. Specimen numbers and complete repository information, including museum/institution abbreviations and geographic information, are included in S1 and S2 Datasets. The list of institutional abbreviations and full institution names are listed in the manuscript under the heading “Institutional Abbreviations.” No permits were required for this study, and I added the relevant language to the end of the “Data Collection” section under “Materials & Methods.”
3. The map in Figure 1 (previously Fig 1) was obtained from Natural Earth, which makes all its content freely available under a Public Domain CC license. According to the text on this [Terms of Use page](https://www.naturalearthdata.com/about/terms-of-use/), no permission is required to use Natural Earth maps for any purpose, including commercial use, so the permission form was unnecessary. I added the necessary attribution to the Fig 2 caption as indicated in the Editor’s comments. I also embellished the map in Fig 2.

In response to the Additional Editor Comments:

- This manuscript was indeed a chapter of my dissertation. I had a feeling that the manuscript might be a bit too long, but I was not sure which material to omit, so I appreciate all the constructive suggestions about which material to rework and/or move to a separate file. I have now moved a considerable amount of the Materials & Methods content to a new file of Supplementary Information.
- I made the writing style of the manuscript sound more impersonal (e.g., removing all use of the word “my”) while being careful to not use passive voice too often.
- I revised Table 1 and added the number of fossil species sampled per taxonomic group.
- I added a new table with regression function equations and statistics (slope, intercept, confidence intervals, SEE, etc.; see Table 3).
- I updated the regression equation methods according to the following suggestions:
  - I used the anguid regressions to estimate SVL for large-bodied fossil anguid specimens, not just the small- to medium-bodied ones. I no longer use any “reference” anguid specimens to estimate head length for large-bodied fossil anguids.
    - I omitted the Helodermatidae data and regressions accordingly. I only used the largest helodermatid specimen as an additional test specimen for the anguid regressions since it is the closest morphological analogue to large glyptosaurines in my dataset. The anguid regressions proved comparably accurate in predicting the SVL of the largest helodermatid test specimen compared to the smaller extant and extinct anguid test specimens.
    - I omitted the former S8 Table (Helodermatidae Regressions), S9 Table (Anguid Anatomical Ratios), and S10 Table (Anguid Reference Specimens), as they were no longer relevant.
  - I ensured that no juveniles were included in any of the regression datasets.
  - I used natural log to transform all the regressions for consistency.
    - Natural log is commonly used to transform data in studies of body size, especially for vertebrates (e.g., Head et al 2013). Although some of the studies the editor recommended use log base 10 (e.g., Figuerido et al. 2011), several of the papers the editor recommended use natural log (Alroy 1998, 2000; Egi 2003). I also tried applying both natural log and log_10_ equations on some of the largest test specimens examined in this study (e.g., extant anguid equations applied to *Gerrhonotus*) and the resulting SVL predictions were exactly equal. Therefore, I used natural log throughout the revised manuscript. I provide this explanation in the revised methods section (Line 322).
  - I ranked the regression equations in order of preference according to the standard error of the estimate, as in Egi (2001). I updated the “Protocol for Estimating Fossil Lizard Body Size” accordingly. I also updated all body size/mass distribution and rarefaction figures accordingly.
- I tried calculating general regression equations for all the anatomical elements I was using in order to perhaps better accommodate the largest fossil specimens. However, none of the general equations passed tests for both normality and homoskedasticity, so I was not able to use any of them. I address this in Results & Discussion.
- I restructured the Results & Discussion section in the style of Egi (2001), with a new discussion of the accuracy of the regression methods in terms of SEE, followed by a distilled discussion of lizard body size estimates.
- I omitted the discussion of ecological and dietary adaptations in fossil lizards in the Discussion section, since the editor felt that the subject did not fit because it was not statistically quantified. I now agree that this subject was not essential to the focus of the paper.
- Instead, I now comment more on patterns of body size diversification through time, possible sources of bias in the fossil record, and competition and temperature (using data from Zachos et al. 2008) as possible drivers of body size evolution in lizards. I include correlation graphs and statistics as additional supplementary figures to augment this discussion.
  - These new figures (S5 and S6 Figs), as well as an updated version of S1 Fig (previously Fig 5) are presented as composite figures with their default formatting retained. I did not fully format these figures because I do not yet know if you will want to keep these figures. If you do think they are worth retaining, I can fully format them to make the numbers on the axes more visible (see S3 and S4 Figs, which I fully formatted because you mentioned them favorably in your comments).
- I added a figure illustrating how I measured SVL from skeletons (the second caudal vertebra is the approximate position of the middle of the cloaca; see the new Fig 1) and cited it in the Methods and Supplementary Information.
- I added symbols to Fig 2 indicating the exact location of the fossil localities sampled. I even created a legend to categorize the localities by the lizard family sampled. Locality names, coordinates, and ages are provided with the fossil data in S1 Dataset.
- I revised the Introduction to omit any repeated information.

In response to the line items under Additional Editor Comments:

- I distilled the Introduction and removed the repetitive material about previous studies of fossil lizard body size.
- *Line 98-99: how many families are you analyzing among extant and fossil species? Are you testing that regression slopes will not change between families? Make this question a bit clearer. Are the fossil crown lizard completely extinct taxonomic groups or can they be linked to extant families in some way?*
  - I added additional context to help clarify the hypothesis, including the following language: “I test this hypothesis using eight taxonomic lizard groups (seven families + Iguania, which encompasses the polytomy of iguanian families) that are represented in the Paleogene fossil record and still exist today.”
- *In Table 1 you can avoid putting Ln and just mention in the caption that all measurements were natural log transformed. If for some you did not do it, you need to explain why. It will make more sense to generate another table. You need to arrange this info in a better way so that for each taxonomic group we can understand how many anatomical elements were regressed [e.g. each anatomical element can be a separate column].*
  - I updated Table 1 accordingly.
- *Line 178-202 looks more of supporting information not needed at this stage. Try to make condense the crucial points in a small sentence and transfer these details in the Supplementary.*
  - I shortened this section and moved the details to the new Supplementary Information file.
- *Line 209-213: If you included both skeletonized and wet SVL estimates in the same dataset did you make sure that they correspond and correlates well in specimens where you can verify this? Using X-ray of wet-specimens you can measure the SVL based on the skeleton in a subsample [e.g. 10 random individuals] and verify if it correlates well with measurements taken from external body.*
  - I do not have the funding available to CT-scan a sample of wet specimens. But I carefully aligned the pelvis of a wet specimen from each of several different families with those of a dry skeletonized specimen of comparable size from each of the same families to figure out where the cloaca would be positioned on the skeleton. This is how I determined that the approximate middle of the cloaca corresponded to the posterior centrum of the second caudal vertebra. I added this clarification to the text, and I moved that paragraph to the Supplementary Information.
- *Line 217-22-: this can be another source of error because depending on the photos you might get biased measurements. Can you assure us that this is not the case? Do you have species where this can be verified (e.g. species averages SVL from wet measured individuals correspond well with those from photos of website?)?*
  - I did go back and measure some of these specimens again and confirmed that the measurements I had taken from photographs were accurate with a margin of error of about 5% or less (1-3 mm). I am therefore confident in my measurements. I moved this detail to the Supplementary Information and added the additional explanation. I had also forgotten to point out that some of my fossil specimens (those from UMMP) were also measured from photographs. I now mention this in the same section in the Supplementary Information.
- *Line 312 onward: this is fine although it will be more re-assuring to test if your slope do not differ between sexed individuals in a model: body_size~bone_L+sex+bone_L*sex. Same should be done for juveniles whose data points might alter sensibly the slope of only adult regression. If that is an improvement on one side, on the other it might be an additional source of error so be careful.*
  - I cannot perform this test because I do not have sex data for enough of the extant specimens that I measured (sex was not indicated for most of them). I eliminated all juvenile specimens from my datasets, so the latter suggestion need not apply.
- *Line 806-808: this sentence is kind of suggesting us that your study is not that important. It is good that you acknowledge previous work on diversity pattern but I presume your work is a way forward. You need to re-phrase this work and make consideration about why your study is important! Also in the next section you are making a lot of justifications for your choice…this is not necessary. You need to re-calibrate your writing here.*
  - I moved all of the content that was under “Considerations” to the relevant parts of the Materials & Methods section or to the Supplementary Information.

In response to Reviewer 1’s comments:

- For the fossil lizard specimens that had been previously referred to Gerrhonotinae, I updated the identification to “Anguidae indet.” and cited the references that this reviewer suggested (Ledesma et al. 2021, Scarpetta et al. 2021).
- I changed the terms “fossil taxa” and “fossil members” to “extinct taxa” and “extinct members” throughout the manuscript.
- I followed every individual suggestion outlined in the additional notes, with the following exception:
  - “Line 354-355: I would put “*Celestus*” as the genus outside of the parentheses for both of these; *Comptus* and *Siderolamprus* are not currently recognized to my knowledge”
    - I double-checked the Reptile Database: *Celestus* is now the currently recognized genus over *Comptus* for *C. c. crusculus*, but *Siderolamprus (Celestus) enneagrammus* is still currently valid.

In response to Reviewer 2’s comments:

- I updated the SVL distribution figure (see the new Fig 6) according to the new methods employed here and with all measurements transformed using natural log.
- I added clarification on the number of families, genera, and species analyzed in the main text under “Data Collection.”
- I recalculated lizard body mass using the family-specific and general equations from Meiri (2010). See the updated Methods section and Supplementary Information for details.
- I commented on preservation bias in the fossil record in the new Results & Discussion section and in the Supplementary Information under “Data Collection.” I also clarified that the omitted outliers were exceptionally large in the section on “Testing and applying regressions.”
- I added new data and discussion about body size changes in the context of temperature changes through the Paleogene.
- All body size figures are now natural log transformed.
- I turned the “Protocol for estimating body size of fossil lizards” into a flowchart. It is now a supplemental figure.
- I followed every individual suggestion outlined in the reviewer’s notes, with the following minor exceptions:
- “Abstract – should be one paragraph”
  - I don't think this matters, as I have seen plenty of abstracts with multiple paragraphs. I felt that breaking up the abstract into two paragraphs here signals the start of a new idea and makes it easier to read.
- “206 – cite”
  - Rather than providing a citation, I added an explanation of the method I used to determine the position of the cloaca on a dry skeletonized lizard specimen.

Thank you again and I look forward to hearing back from you.

Best regards,

Sara J. ElShafie, Ph.D.

Alumnus, Department of Integrative Biology and Museum of Paleontology

University of California, Berkeley

selshafie@berkeley.edu
