# Supplementary material for: Body size estimation from isolated fossil bones reveals deep time evolutionary trends in North American lizards
Source: PLoS One. 2024 Jan 5;19(1):e0296318. doi: 10.1371/journal.pone.0296318 (PMC10769094; doi:10.1371/journal.pone.0296318)
Supplement: Attachment — Submitted filename: Review_PONE-D-22-04244_V2.docx [file pone.0296318.s022.docx]

Dear author,

Thank you for submitting this revision! Almost all changes I requested were implemented, and the same is the case for comments from the editor and other reviewer. The manuscript is in much better shape after being shortened/condensed, having some superfluous sections removed (eg glyptosaurine diet), and having some changes made to the analyses. I have a few minor suggestions, but no major changes to request. Congratulations!

Simon Scarpetta

Minor changes

Line 65 page 4. “The first question to consider here is which variable would be best to use as a measure of body size.”

Phrasing is awkward in my opinion, I would say “It is not unambiguously clear which variable is the best proxy for body size.” Or something like that

Line 70 page 4. Add comma after “fossil vertebrates” and “many” before “fossil vertebrates”

Line 71 page 4. “Body length is often more feasible to estimate for fossil reptiles”

Relative to mammals? Dinosaurs? If the latter, have to rephrase given that dinos are a reptile

Line 101 page 5. “I use this geotemporal system as a paleontological framework”

Understand intent but this is confusing to me- maybe “I use the timeframe as a study system” or something

Line 103 page 5. Use “dactyloids” instead of “polychrotids”

Line 120 page 6. Not sure I would say polytomy, as even among pleurodontans some of the relationships are reasonably clear. So might remove this word and the of after, or clarify that not all the relationships are clear rather than saying polytomy which implies that none of them are.

Line 143 page 7. TMM. I believe they prefer TxVP (Texas Vertebrate Paleontology) now; I would confirm with Chris Sagebiel

Line 166 page 9. “anguid” should really be “anguimorph” because *Heloderma* is not an anguid but is presented as a modern analogue

Line 179 page 9. Delete “(Varanidae)”

Line 184 page 9. Change “Iguanidae; Polychrotidae” to “Iguania; Polychrotidae”

Line 322 page 19. Add likely between words “taxa exceeded”

Line 354 page 20. After “paleocommunities” add “ similar to modern ecosystems”

Line 359 page 21. “rarefaction analysis in S4 Fig would indicate that this study” change to . “rarefaction analysis (Fig S4) may indicate that this study”

Same line. “diversity”. Taxonomic diversity? Body size diversity? Next sentence seems to indicate body size, so please clarify here.

Line 378 page 21. “iguanians” clarify that the iguanians that occurred there then were extirpated, not iguanians in general (still plenty of iguanians in west/central NA!!)

Line 402-404. “abundant group in this study that included large-bodied individuals, negatively correlated with maximum body size in Xantusiidae and Xenosauridae, two of the most common small-bodied groups”

Something is incomplete/missing in this sentence, please clarify

Line 965 page 49. I would use “Leiolepidinae” instead of “Leiolepididae”

Line 979, page 50. Change “But” to “However,”

Line 1027, page 52. Change “recover” to “incorporate” and remove “as a result” on the next line
