# Supplementary material for: Body size estimation from isolated fossil bones reveals deep time evolutionary trends in North American lizards
Source: PLoS One. 2024 Jan 5;19(1):e0296318. doi: 10.1371/journal.pone.0296318 (PMC10769094; doi:10.1371/journal.pone.0296318)
Supplement: Attachment — Submitted filename: Plos review - 2.docx [file pone.0296318.s023.docx]

PLOS ONE review

Overall, the ms has improved greatly and I highly recommend its publication after major revisions. I think that the drivers of body size evolution and taxonomic turnover sections can be expanded and the author should give the space necessary to properly synthesize all the data acquired and put these species in a context of living and extinct squamates. Developing allometric equations is relatively straightforward (though the amount of work done here is impressive!) I think that what would benefit the scientific community most is to better understand your interpretation of the data, and what the next steps should be moving forward in this field. Here are some things to consider adding to the section: drivers of reptile body size evolution (perhaps you can look at studies done by Meiri), maximum body size in squamates in history and how they got so big (Titanoboa, Megalania, mosasaurs), diet (unless all species here were carnivores? Herbivores are usually larger, you can check diet in Lafuma 2021), substrate use and ecology (were all species terrestrial, semi-arboreal, semi-aquatic, could this have affected body size), filling empty niches after the kpg extinction (a huge point that wasn’t mentioned), the PETM. Body size evolution is a rich field with much to dig into and I feel that it needs to be done.

-Jacob Dembitzer

**General comments**

I made a mistake recommending Meiri 2010 in my last review for body mass estimates. I meant Feldman 2016 (which Meiri is on) supplementary appendix 2 (see below). It has all of the regression equations needed for your study including the families missing at the moment. There is an equation for legged anuids and legless anguids. If I’m not mistaken there are a few species that you are not sure if they had limbs or not? In which case you might want to use the Anguimorpha equation, or simply assume that they had limbs if there are no known limbless glyptosaurs. In any case you definitely need to change this.

You should clarify how you measured skull length. Was it the absolute farthest point of the skull to tip of snout (in some cases that’s the jaw)? Was it to the occipital condyles?

**Specific comments**

27 – you say “large dataset” of fossil lizards but I think that you should be specific in saying what percent of Midwest species you studied. You can check this on the paleobiology database I think.

36 – You need to say in the study period or something as it reads a bit confusing. lizards did not reach maximum body size in the Paleogene. Pleistocene Megalania was much larger and Mosasaurs were much larger still.

40 – take out “reasonable”, estimates is good enough

49 – 50 – instead of saying “lizards occupy a wide range of…” which you say in the previous sentence, I would say “lizards (or all squamates?) make up the majority of tetrapods in harsh environments, especially deserts,” and cite Roll et al. 2017.

53 – I think you can take out unfortunately complete fossils are rare. Just say isolated elements are most common (this is true for any group though).

65 – 77 – instead of discussing crocs I would discuss how people have recently been using raw jaw length as a measure of body size across all lepidosaurs, see Herrera-Flores 2022 and 2021. Or discuss both but lepidosaurs are more relevant.

155 – and how many recognized species? Just say not all specimens that were attributed to a genus could be attributed to a specific species.

162 – maybe add a column for genera then? It’s really important that it is clear to everyone what data you collected.

187 – table 2, perhaps you could add in parentheses number of genera in each family in each NALMA

218 – log10 will make better graphs because you can label your axes much more easily (10mm, 100mm, 1000mm). if it takes too much time don’t bother as it’s not essential (though you’re readers will appreciate it).

278 – this section is perhaps the most interesting (and if I’m not mistaken the point of the study). I would expand on it either here or in the discussion I would recommend that you try to provide an overview of species' body mass evolution in squamates and other groups at this time in earth’s history. So maybe mention larger species in other places on earth like Barbaturex morrisoni and at some point, I would add a sentence about what the largest lizards in history were and compare them (mosasaurs and Megalania). You could also show the entire body size distribution of lizards in your study period in a histogram and compare them to the distribution in Feldman 2016. Or maybe just write a sentence on it. If you don’t want to add another figure then write in a sentence or table the mean, median and range for all species in the study period and then different NALMA periods. I would show body size distribution of species for the entire study period in a histogram and in the supplementary material divide it by NALMA. You could potentially do these things with both svl and mass. You can be creative with this, but I think that it should be clear that this is the meat of the research and should be expanded on. In theory you could’ve just written a paper using the allometric equations, but you’re in too deep now (and that’s a good thing).

334 – mention Megalania size

361 – its more likely that there were smaller species in the system than larger ones as larger ones fossilize better

385 – 389 – you need to be more specific; I don’t understand any of this. Why would lizards be smaller in the Paleogene and bigger in the Oligocene for the same reason? This is the interesting part, elaborate!

400 – you can remove the first paragraph of this section, it says the same thing. Or combine the two.

408 – I would add the example of Titanoboa to this section and how it was supposedly able to reach giant size because of the PETM

414-421 – really interesting analysis, you should use family as an additional variable in addition to doing it across all species. Also, how did you try to correlate them? Linear regression model? ANOVA based on each NALMA? What was the predictor and what was the response? This remains very unclear in the text. You should provide a quantitative analysis, perhaps similar to Smith 2018. Or at the very least, a very in-depth qualitative analysis/discussion. Quantitative is always better, so I would recommend that. I would personally recommend using linear regressions with estimated body size of species as the response, and temperature per million years as the predictor (check where smith got the data, I think Zachos something). You can also use family as an additional covariate.

Feldman, A., Sabath, N., Pyron, R.A., Mayrose, I. and Meiri, S., 2016. Body sizes and diversification rates of lizards, snakes, amphisbaenians and the tuatara. *Global Ecology and Biogeography*, *25*(2), pp.187-197.

Roll, U., Feldman, A., Novosolov, M., Allison, A., Bauer, A.M., Bernard, R., Böhm, M., Castro-Herrera, F., Chirio, L., Collen, B. and Colli, G.R., 2017. The global distribution of tetrapods reveals a need for targeted reptile conservation. *Nature ecology & evolution*, *1*(11), pp.1677-1682.

Herrera‐Flores, J.A., Elsler, A., Stubbs, T.L. and Benton, M.J., 2022. Slow and fast evolutionary rates in the history of lepidosaurs. *Palaeontology*, *65*(1), p.e12579.

Herrera‐Flores, J.A., Elsler, A., Stubbs, T.L. and Benton, M.J., 2022. Slow and fast evolutionary rates in the history of lepidosaurs. *Palaeontology*, *65*(1), p.e12579.

Herrera-Flores, J.A., Stubbs, T.L. and Benton, M.J., 2021. Ecomorphological diversification of squamates in the Cretaceous. *Royal Society Open Science*, *8*(3), p.201961.

Smith, F.A., Elliott Smith, R.E., Lyons, S.K. and Payne, J.L., 2018. Body size downgrading of mammals over the late Quaternary. *Science*, *360*(6386), pp.310-313.
