# Supplementary material for: Body size estimation from isolated fossil bones reveals deep time evolutionary trends in North American lizards
Source: PLoS One. 2024 Jan 5;19(1):e0296318. doi: 10.1371/journal.pone.0296318 (PMC10769094; doi:10.1371/journal.pone.0296318)
Supplement: Attachment — Submitted filename: Response to Reviewers.docx [file pone.0296318.s024.docx]

February 28, 2023

Dear PLoS One Editorial Board,

Thank you for the opportunity to make these additional revisions to my manuscript. Below is a list of the new changes that I made.

**Overall Changes Made:**

- I revised the whole manuscript to be in third-person passive voice to make it sound more impersonal.
- I separated the Results and Discussion sections and substantially revised both sections based on the Editor and Reviewer comments, including omitting the section on Competition from the Discussion.

**Body Mass Estimations and Equations**

- I agree that adding equations for estimating mass of fossil specimens from individual bone measurements is useful. However, I cannot generate novel equations for body mass from individual lengths here because almost none of the extant lizard specimens that I measured included measurements of body mass. Unfortunately, lizard specimens that are dry skeletonized almost never have the original weight recorded before they are processed. Furthermore, measurements of individual cranial and limb bones are not provided in Feldman et al. (2016) or Meiri (2010), both of which were recommended by Reviewer 2 as sources for extant lizard measurements and equations for lizard body mass from SVL. Therefore, I do not have the data necessary to generate novel equations for estimating body mass from individual cranial or limb bones.
  - Instead, I translated the original equations that I provided here for estimating SVL from individual bones (Tables S1-S7) into the equations for estimating mass from SVL provided in Feldman et al. (2016; see S8 Table). The resulting equations have been added to Tables S1-S7. **These translated equations can now be used to estimate lizard body mass from individual cranial and limb bones.**
  - The mass estimates are the same whether you calculate mass from SVL using the equations from Feldman et al. (2016) or the new equations for body mass from individual elements provided here.
- I am still including the original equations I generated for estimating SVL from individual bones because SVL is also an important variable for paleontologists and zoologists.
- Calculating body mass equations for individual fossil species would not be practical because fossil specimens usually cannot be identified to individual species. In addition, even genus-level assignments often change for fossil specimens upon later reexamination.
  - It is safer and more practical to develop family-level equations - that way, the body mass estimate becomes resilient to any specific taxonomic identification changes. Furthermore, family-level equations are based on a larger sample size. This approach is consistent with the rest of the paper, and with the sources used (Meiri 2010; Meiri et al. 2013; Feldman et al. 2016), which focus on family-level analyses.

**New Figures and Tables**

- I added the following figures:
  - **Fig 7.** Body mass distribution by taxonomic group for fossil lizards in the Western Interior of North America through the Paleogene, including a line for the overall mean lizard body mass per NALMA.
  - **S6 Fig.** Mean and maximum snout-vent length in fossil lizards by taxonomic group per NALMA.
  - **S7 Fig.** Mean and maximum body mass in fossil lizards by taxonomic group per NALMA.
    - I added commentary about the latter two new figures in the revised Discussion as well.
- I also added a line for the overall mean lizard snout-vent length per NALMA to the SVL distribution graph in Fig 6.
- I added the following tables:
  - **S8 Table.** Published equations for estimating lizard body mass from snout-vent length.
  - **S11 Table.** Mean and maximum fossil lizard snout-vent length and body mass by taxonomic group per NALMA.
- The numbers of all other figures and tables were also updated accordingly.

I made all changes suggested by the Editor and Reviewers, with the following notes…

**Additional Editor Comments:**

- *Table 2 is a bit broad and I think it would be better a figure showing specimens and /or species sampled by group / time period in comparison with diversity expected as by the palaeodb database.*
  - I still prefer these data presented as a table, and I think it’s helpful to have the NALMAs listed here in table format with their age ranges. But I took the suggestion of adding the number of genera and species sampled per taxonomic group per NALMA compared to the numbers expected based on the Paleobiology Database (PBDB). These data have been added to the table. I think the result is more a reflection of how much is missing from the PBDB, but the data are now provided for comparison.
- *Table 3 should be simplified showing slope as parameter (b), confidence interval and intercept + R2 and SEE.*
  - I made these changes, but for the sake of space and simplicity, I listed the standard error value rather than listing the full confidence interval (e.g., - X.XXX to X.XXX).
- *Line 264-269: ok but perhaps this might be due to some outliers or some groups whose body bauplan differ from the "general" lizard one. What if you try exclusion of some groups?*
  - I did try excluding some groups, and the attempted general regressions still did not pass the tests for homoskedasticity and normal distribution of residuals. In any case, now that body mass estimation equations specific to taxonomic group are used in this study, I think a generalized lizard SVL equation is unnecessary.
- *Reduce extensively also the part on sex (line 936-948). You did not have sex to test so all you are saying is quite speculative.*
  - I think the comment made here is an important point to make for paleontological studies, and Reviewer 1 agreed. However, I did shorten this paragraph.

**Reviewer 2 Comments:**

- *I made a mistake recommending Meiri 2010 in my last review for body mass estimates. I meant Feldman 2016 (which Meiri is on) supplementary appendix 2 (see below). It has all of the regression equations needed for your study including the families missing at the moment. There is an equation for legged anuids and legless anguids. If I’m not mistaken there are a few species that you are not sure if they had limbs or not? In which case you might want to use the Anguimorpha equation, or simply assume that they had limbs if there are no known limbless glyptosaurs. In any case you definitely need to change this.*
  - I used updated equations from Feldman et al. (2016). The equations listed in this reference were only different for Anguidae, Xenosauridae, Iguania, and Anguimorpha; the rest were taken from Meiri (2010), so I didn’t need to change those. I used the Anguimorpha equation for Shinisauridae since an equation for Shinisauridae was not provided and Shinisauridae falls within Anguimorpha. I used the equation for Fully Legged Anguidae for the fossil anguid lizards because limb material has been found with many fossil anguid specimens – none have been interpreted as limbless.
- *27 – you say “large dataset” of fossil lizards but I think that you should be specific in saying what percent of Midwest species you studied. You can check this on the paleobiology database I think.*
  - I honestly don’t know what Reviewer 2 meant by this comment. Only some of the fossil lizards in my dataset were collected in the Midwest, and I don’t know why that matters in the context of this study. If they meant that I should specify how many of the taxa in the fossil dataset still occur in the Western Interior today, I point that out in the Results section (see “Taxonomic Turnover”). I don’t think the Introduction is the place for that detail.
- *414-421 – really interesting analysis, you should use family as an additional variable in addition to doing it across all species. Also, how did you try to correlate them? Linear regression model? ANOVA based on each NALMA? What was the predictor and what was the response? This remains very unclear in the text. You should provide a quantitative analysis, perhaps similar to Smith 2018. Or at the very least, a very in-depth qualitative analysis/discussion. Quantitative is always better, so I would recommend that. I would personally recommend using linear regressions with estimated body size of species as the response, and temperature per million years as the predictor (check where smith got the data, I think Zachos something). You can also use family as an additional covariate.*
  - I revised the text to clarify how I set up this regression. However, the additional analyses suggested here were not feasible because the predictor variable (lizard body size) could not be binned per million years due to the way in which the specimens were documented in museum collections (the majority of the specimens are only referred to a NALMA interval and cannot be referred to a more precise time bin). Furthermore, only two lizard families (Anguidae and Varanidae) represent the maximum lizard body size for any given NALMA, and the record of Varanidae is not continuous enough through the Paleogene to set up a correlation (see S6-S7 Fig). The correlation is already mostly a correlation between maximum anguid body size and varanid body size.

Thank you again for the considerable time you, the Editor, and the Reviewers have contributed to reviewing this paper, and for your patience as I incorporated the suggestions into the manuscript. I look forward to hearing back from you.

Best regards,

**Sara J. ElShafie, Ph.D.**

Alumnus, Department of Integrative Biology and Museum of Paleontology

University of California, Berkeley

selshafie@berkeley.edu
