# Supplementary material for: Body size estimation from isolated fossil bones reveals deep time evolutionary trends in North American lizards
Source: PLoS One. 2024 Jan 5;19(1):e0296318. doi: 10.1371/journal.pone.0296318 (PMC10769094; doi:10.1371/journal.pone.0296318)
Supplement: Attachment — Submitted filename: Response to Reviewers.docx [file pone.0296318.s028.docx]

October 6, 2023

Dear PLoS One Editorial Board,

Thank you for the opportunity to make these additional revisions to my manuscript. Below is a list of the new changes that I made.

**Overall Changes Made:**

- I revised the Introduction to make it more concise.
- I heavily reworked the Materials and Methods section to be sufficiently detailed and more cohesive.
- I took the text that had been moved to Supporting Information, distilled it, and incorporated it back into the main text.
- I added mean values to Table 4 to provide the full data visualized in Fig 6, Fig 7, S6 Fig, and S7 Fig. I also revised the captions for those figures.
- I removed all material related specifically to taxonomic turnover. This included removal of the former Table 2 and S3 Fig, which were no longer relevant.

I made all changes suggested by the Editor and Reviewers, with the following minor exceptions:

**Editor comments and edits (lines refer to previous version of the manuscript)**

- Line 18: With respect to the Editor’s comment, I did not indicate that lizards are more vital to extant ecosystems than pollinating insects or any other group, only they that they are important. I elaborate on this with citations in the Introduction. I maintain that this is a reasonable and appropriate opening sentence for this abstract.
- Lines 32-35: I like the more concise phrasing suggested here. However, the femur was only possible to regress for Anguidae, not the other lizard groups, so I omitted that mention.
- Lines 159-160: I think the justification for the chosen study system is best placed in the Introduction, and does not need to be reiterated in the Materials & Methods section.
- Line 370 – “*do you mean correlation r????*”: I mean the coefficient of determination for the correlation, *R^2^*. I think this sentence was confusing as written, so I revised it.
- Lines 395-406: With respect, I do not think that this information is off topic. It helps to explain why we might be seeing the observed patterns even though the maximum body sizes to not correlate with global temperature data. I left this information in the “Climate as a potential driver of body size evolution” section of the Discussion, but I did revise that section to be more cohesive.
- *The section Appendix should be part of supporting material [Appendix S1] and needs to be more in line with PlosOne style and nomenclature (*[*https://journals.plos.org/plosone/s/supporting-information*](https://journals.plos.org/plosone/s/supporting-information)*).*
  - I am not sure what the Editor meant by this comment because there was no section in the manuscript labeled “Appendix.” If they were referring to the supporting datasets (S1 and S2 Datasets), I see no reason to change the file name because “Dataset” is listed as one of the common item descriptions in the Supporting Information guidelines link provided by the Editor. Those files are datasets, and I would prefer to designate them as such for clarity. Furthermore, I did not see any instructions in the linked guidelines as to a required format for dataset files. If the Editor would like to request specific changes to the formatting of S1 and S2 Datasets with explanations for the needed changes, I would be happy to make those changes.

**Reviewer #3 comments and edits (lines refer to previous version of the manuscript)**

- Lines 234-238: Log was not written out as “logarithm” in the PLoS One publication that the Editor recommended as a formatting reference (Field et al. 2013), and “log” is the more common term used, so I did not see the need to write it out here.
- Lines 267-272: I agree that the explanation of how I derived new equations for estimating body mass was not very clear as written. I revised it to be more coherent. However, I decided to stick with the existing arrangement of explaining the equations that had already been published and then explaining how I derived new equations because I had to use the existing published equations to generate the new equations.
- Line 419: Correct, mass cannot be zero, but the mass listed here is 1kg, with a natural log of 0.00.
- *I would suggest starting the data collection section by saying the total number of collected specimens and the number of genera they represent.*
  - I understand what the reviewer is getting at – it would be confusing to only discuss the fossil data but not the extant data before presenting a table that offers both fossil and extant data. However, this is just an artefact of the formatting for the submitted manuscript. Since the tables will be separated from the text in the final layout, this will no longer be an issue. I think the text flows better with the details for the fossil data presented together in one paragraph, followed by the details for the extant data in the next paragraph. But I also revised the entire Data Collection section to improve its clarity.
- *Rows 227-229: I suggest putting this sentence in line with rows 232-235 sayings that although bivariate regression is frequently used, you chose to utilise RMA instead, and then discuss the variables you employed.*
  - RMA is a form of bivariate linear regression, not an alternative to it. I added the word “bivariate” after RMA to clarify this for anyone who is not familiar with RMA regressions.
- *It is not very clear from the main manuscript that the fossil dataset includes also juveniles, in my opinion.*
  - There was a brief mention of this in the Methods, and the SI included commentary on why juveniles were not included in the extant dataset and why they were likely not represented in the fossil dataset. I revised that commentary and moved it back to the Methods section in the main text.

Thank you again for the additional time that you, the Editor, and the Reviewers have contributed to reviewing this paper, and for your patience as I incorporated the suggestions into the manuscript. I look forward to hearing back from you.

Best regards,

**Sara J. ElShafie, Ph.D.**

Alumnus, Department of Integrative Biology and Museum of Paleontology

University of California, Berkeley

selshafie@berkeley.edu
