# Supplementary material for: Body size estimation from isolated fossil bones reveals deep time evolutionary trends in North American lizards
Source: PLoS One. 2024 Jan 5;19(1):e0296318. doi: 10.1371/journal.pone.0296318 (PMC10769094; doi:10.1371/journal.pone.0296318)
Supplement: Attachment — Submitted filename: Response to Reviewers.docx [file pone.0296318.s030.docx]

December 7, 2023

Dear PLoS One Editorial Board,

Thank you for the opportunity to make these additional revisions to my manuscript.

I made all changes suggested by the Editor and Reviewer #3, with the following minor exceptions:

**Editor comments and edits (lines refer to previous version of the manuscript)**

- In line 32 write: Individual bones predict SVL of fossil lizards with high level of accuracy (R > 0.9)
  - I agree with the editor that the sentence, as it was written, needed improvement. I also realized that the sentence was not conveying what I had intended. I revised it accordingly for clarity.
- Line 46: change "roles" with "niches"
  - For the term “niche,” I refer to Van Valen’s 1971 paper in which he regards “niche” as the specific ecological resource space that a species occupies [Van Valen, L. "Adaptive zones and the orders of mammals." *Evolution* (1971): 420-428]. Thus, for the context of the sentence in question, I consider “roles” to be a more appropriate term.
- Figure 7 and 8 are identical so maybe just add an axis to the right showing body mass values that reflect the SVL values or add this into the appendix. It will be good to see a climate Oxygen curve at the bottom of this graph. Nothing fancy but something similar to this Fig. 3: "<https://www.science.org/doi/10.1126/science.1194830>"
  - I agree that it was a good idea to combine the SVL and mass figures since they were essentially the same. I also liked the idea of adding in the Zachos curve. In the interest of keeping the figure from looking too crowded, I accomplished this by reflecting the LN(Mass) scale onto the LN(SVL) scale on the lefthand side, and adding the Zachos Curve scale on the righthand side. I also simplified the Zachos Curve into a single dotted line representing the mean annual paleo temperature (MAPT) for the midpoint of each NALMA interval, as derived from ∂O^18^ isotope proxies. I noted these changes in the new Figure 7 caption.
- Line 507-510: again this must go in the results. Conclude your Methods also mentioning that you will test the hypothesis of climatic influence on global lizard maximum, minimum and average body size using oxygen isotopes as proxy for climate. Again, in the results please report the correlation test (Pearson or Spearman, very easy to be done in PAST) because it is NOT lizard body size to predict climate, but maybe the other way round (biologically makes more sense which is also in line with your supplementary figures....based on what you wrote it seems that you wanted to predict climate from lizard body size). A simple correlation test will be sufficient with climate curve implemented at the bottom of Figure 7.
  - You are correct that I accidentally reversed my dependent and independent variables here – thank you so much for catching that! I corrected the text in the caption for S5 Fig. I also moved the relevant text from the Discussion to the end of both the Methods and the Results sections and revised accordingly. Furthermore, I ran correlations for Global temperature vs. Maximum and mean mass in addition to SVL. I used RMA regression correlation statistics to be consistent with the methods in the rest of the manuscript.

**Reviewer #3 comments and edits (lines refer to previous version of the manuscript)**

- ***Also from Editor:*** Lines 103, 483: “change ‘were extirpated’ to ‘gone extinct’”
  - The term “extirpated” means that the clade no longer occurs in that particular geographic area, but still exists elsewhere. “Extinct” means that the clade no longer exists anywhere. The lines in question refer to clades such as varanids and xenosaurids­­ that no longer occur in the Western Interior of North America but still exist elsewhere, so the term “extirpated” would be more appropriate than “extinct.” However, in case the Editor and Reviewer #3 think that this term would be unfamiliar for most readers, I changed the wording to “locally extinct.”
- Lines 151-152: “It would be great if you could include a table describing the anatomical measurements that were taken.”
  - Table 2 already includes a list of each anatomical measurement taken for each taxonomic group, and Figure 1 visually indicates how each of those measurements were taken. I do not think that adding a table with any further description of the anatomical measurements would contribute value to the paper. On the contrary, it would be redundant and take up space.
- Lines 202-203, 366-367: I’m not sure if the outer parentheses should be replaced by brackets here since brackets are used for citation numbers in PLoS One’s format. I will leave this up to the discretion of the copy editor.

**I also made the following revisions to figures:**

- As suggested by the Editor, I combined Figures 7 and 8 into a single figure (now just Fig 7) that shows data representing both SVL and mass. I also added the Zachos curve representing global marine temperature to this figure.
- I rearranged the layout somewhat in Figures 1 and 3 to make things less cluttered.

Thank you again for the additional time that you, the Editor, and the Reviewers have contributed to reviewing this paper, and for your patience as I incorporated the suggestions into the manuscript. I look forward to hearing back from you.

Best regards,

**Sara J. ElShafie, Ph.D.**

Alumnus, Department of Integrative Biology and Museum of Paleontology

University of California, Berkeley

selshafie@berkeley.edu
